# Unsymmetric N-heterocyclic carbene ligand enabled nickel-catalysed arylation of bulky primary and secondary amines†

**DOI:** 10.1039/d3sc00492a

**Published:** 2023-03-28

**Authors:** Zi-Chao Wang, Yan-Yu Li, Shuo-Qing Zhang, Xin Hong, Shi-Liang Shi

**Affiliations:** a State Key Laboratory of Organometallic Chemistry, Shanghai Institute of Organic Chemistry, University of Chinese Academy of Sciences, Chinese Academy of Sciences 345 Lingling Road Shanghai 200032 China shiliangshi@sioc.ac.cn; b Center of Chemistry for Frontier Technologies, Department of Chemistry, State Key Laboratory of Clean Energy Utilization, Zhejiang University Hangzhou 310027 China hxchem@zju.edu.cn; c Beijing National Laboratory for Molecular Sciences Zhongguancun North First Street NO. 2 Beijing 100190 PR China; d Key Laboratory of Precise Synthesis of Functional Molecules of Zhejiang Province, School of Science, Westlake University 18 Shilongshan Road Hangzhou 310024 Zhejiang Province China

## Abstract

The arylation of sterically hindered amines represents one of the long-standing challenges in synthetic chemistry. Herein, we report a highly efficient Ni-catalysed arylation of sterically hindered primary and secondary amines with aryl chlorides or phenol derivatives enabled by an unsymmetric N-heterocyclic carbene (NHC) ligand. The protocol provides general, efficient, and scalable access to various sterically demanding anilines in excellent yields under mild conditions. A wide range of functional groups and heterocycles are compatible (>50 examples), including those present in biologically relevant molecules. Computational studies suggest that the unsymmetric bulky and flexible NHC ligand was critical to balance the oxidative addition and reductive elimination elementary steps, thus promoting this challenging transformation.

## Abstract 2

A general and efficient Ni-catalysed arylation of bulky primary and secondary amines with aryl chlorides or phenol derivatives is described. DFT studies suggest that unsymmetric bulky and flexible NHC ligand is critical to promote this reaction.

## Graphical abstract

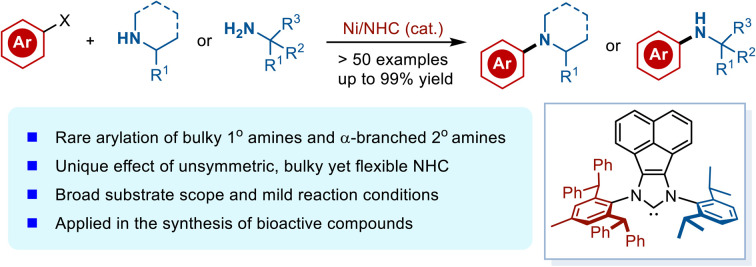

## Introduction

**Fig. 1:**
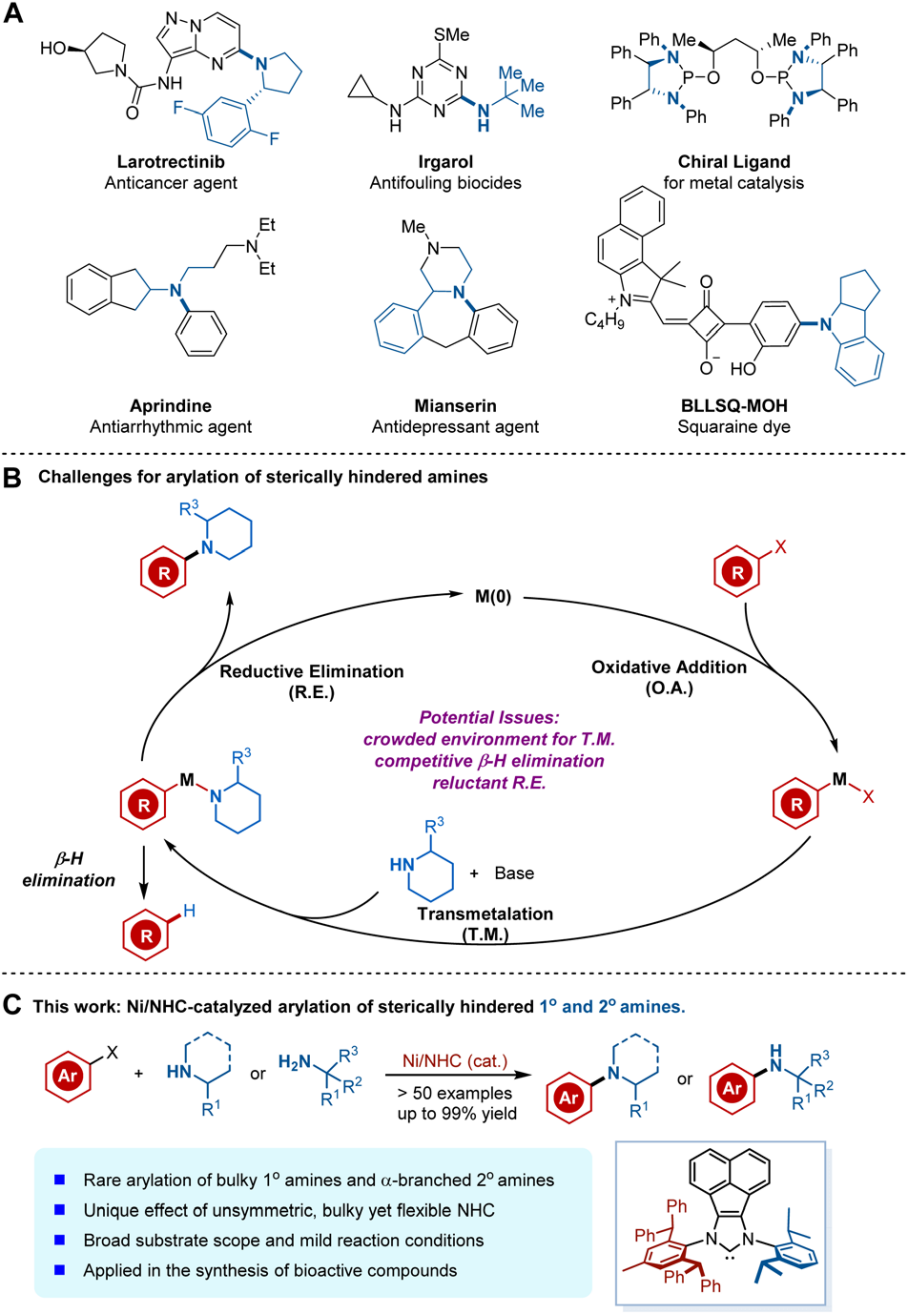
(A) Examples of pharmaceuticals, ligands, and materials possessing bulky aniline motifs. (B) Potential challenges for the arylation of sterically hindered amines. (C) This work.

Transition-metal-catalysed C–N cross-coupling reactions of amines and aryl halides have become some of the most frequently employed and significant transformations in academic research and industrial processes. These amination methods have enabled mild and general access to various *N*-aryl structures ubiquitous in drugs, materials science, and natural products (Fig. 1A).^1–4^ Despite tremendous advances, transition-metal-catalysed arylation of sterically hindered amines remains a challenging but important task. Such transformations are attractive because of the prevalence of bulky anilines in medical agents and their applicability in drug design (Fig. 1A), as sterically demanding substituents can increase a bioactive molecule's lipophilicity or improve its stability against enzymatic degradation.^5^

Despite these attractive features of the coupling of bulky amines, specific factors have impeded the development of such reactions (Fig. 1B). The main challenges stem from the intrinsic poor nucleophilicity of bulky amines as a consequence of steric hindrance, which results in a slower rate of coordination and transmetalation (T. M.) of bulky amines to metals.^6^ Added to this complication is the β-hydride elimination process encountered in most coupling reactions.^7^ In this regard, β-hydride eliminations from the metal-amido complex intermediate often cause the undesired reduced arene as a side product. Accordingly, such challenging yet crucial reactions have attracted chemists to search for more efficient catalytic systems. Nevertheless, very few examples of the Pd or Cu-mediated arylation of bulky amines have been reported, considering the numerous known methods for C–N cross-couplings (Fig. 1B).^6,8^ In these sporadic examples, the design and modification of the ligand architecture represent a fundamental strategy to overcome the key limitations and achieve more efficient cross-coupling.

Recently, more and more attention has been focused on nickel as a catalyst for C–N cross-coupling reactions,^3,9^ not only due to the low cost of the earth-abundant metal but also because of its unique reactivity towards aryl chloride and phenol derivatives.^10^ Despite these advantages, considerable limitations and challenges remain in Ni-catalysed arylation of hindered amines. In addition to challenges associated with transmetalation, Ni-catalysed *N*-arylation also suffers from more reluctant reductive elimination (R. E.) of nickel(ii) amido complexes.^11^ In addition, ancillary ligands developed for Pd catalysts are not fully applicable for Ni catalysts. Therefore, the rational ligand design or strategical application of privileged ligands for Ni-catalyzed *N*-arylation is non-trivial considering the infancy of ligand design for Ni catalysis and the relative lack of general principles.^12,13^ As a consequence, efficient Ni-catalysed arylation of bulky α-branched secondary amines has not been explored, presumably due to the more difficult in balancing the elementary steps and potential deleterious β-H elimination process.

We recently developed an array of bulky yet flexible NHCs^14^ and applied them to a range of Ni-catalysed transformations, including challenging cross-coupling reactions.^15^ We felt that a sterically demanding NHC ligand could promote R. E. At the same time, the conformational flexibility, available through rotatable single bonds on the ligand, may benefit coordination to the metal center, as well as oxidative addition (O. A.) and T. M. processes. As part of our ongoing endeavors in this field, we herein report a highly efficient Ni-catalysed arylation of sterically demanding primary amines and bulky α-branched secondary amines enabled by an unsymmetric bulky yet flexible NHC ligand (Fig. 1C). Our protocol is advantageous in terms of its broad substrate scope, cost effectiveness, simple and mild reaction conditions, and the applicability of aryl chlorides or phenol derivatives when compared to previous Pd- or Cu-based methods.^6,8^ The unique ligand effect of the unsymmetric bulky yet flexible NHC on balancing the elementary catalytic steps was first observed and confirmed by DFT studies, which would inspire further rational ligand design.

## Results and discussion

We began our study by treating commercially available 1,2,3,4-tetrahydroquinaldine (1a) with 4-chlorobenzotrifluoride (2a) in the presence of 2.0 mol% Ni(COD)_2_, 1.2 equiv. *t*-BuOK, and 2.0 mol% imidazolium salts in cyclohexane at room temperature. To our surprise, the use of IPr (L1) and other typical ligands for C–N cross-coupling^3*a*^ failed to afford the desired product 3a (Table 1, entry 1, also see the ESI† for more details). Instead, we observed significant amounts of imine by-product generated from the dehydrogenation of 1a probably through β-H elimination. Interestingly, the bulkier NHC ligands with acenaphthoimidazolylidene skeletons (L2, ANIPr)^16^ or aniline moiety containing benzhydryl groups (L3)^17^ delivered 3a in 26% and 24% yields, respectively (entries 2 and 3). However, a reaction using highly bulky ANIPr* (L4)^18^ resulted in no conversion to the desired product (entry 4). We felt that a suitable sterically hindered but flexible ligand would facilitate this challenging amination process. We thus used our ANIPE ligand (L5) to promote the C–N coupling reaction. Indeed, the yield was improved to 58% (entry 5). However, we found that further improvement in reactivity was difficult since only one of the enantiomers of substrate 1a could react efficiently as a result of a highly selective kinetic resolution by the chiral ligand.^15*h*^ An achiral unsymmetric NHC (L6) with 2,6-diphenethylaniline and 2,6-diisopropylaniline fragments^19^ was then utilized as the ligand. With this change, 3a was formed in a high yield (70%; entry 6). We attribute the low reactivity to inefficient R. E. in L1–L3 and ineffective O. A., T. M., or coordination to nickel for L4, while the unsymmetrical NHC (L6) seems to strike an excellent balance in having favorable steric environments for different stages of the catalytic cycle (*vide infra*).

**Table 1:** Reaction optimization^a^

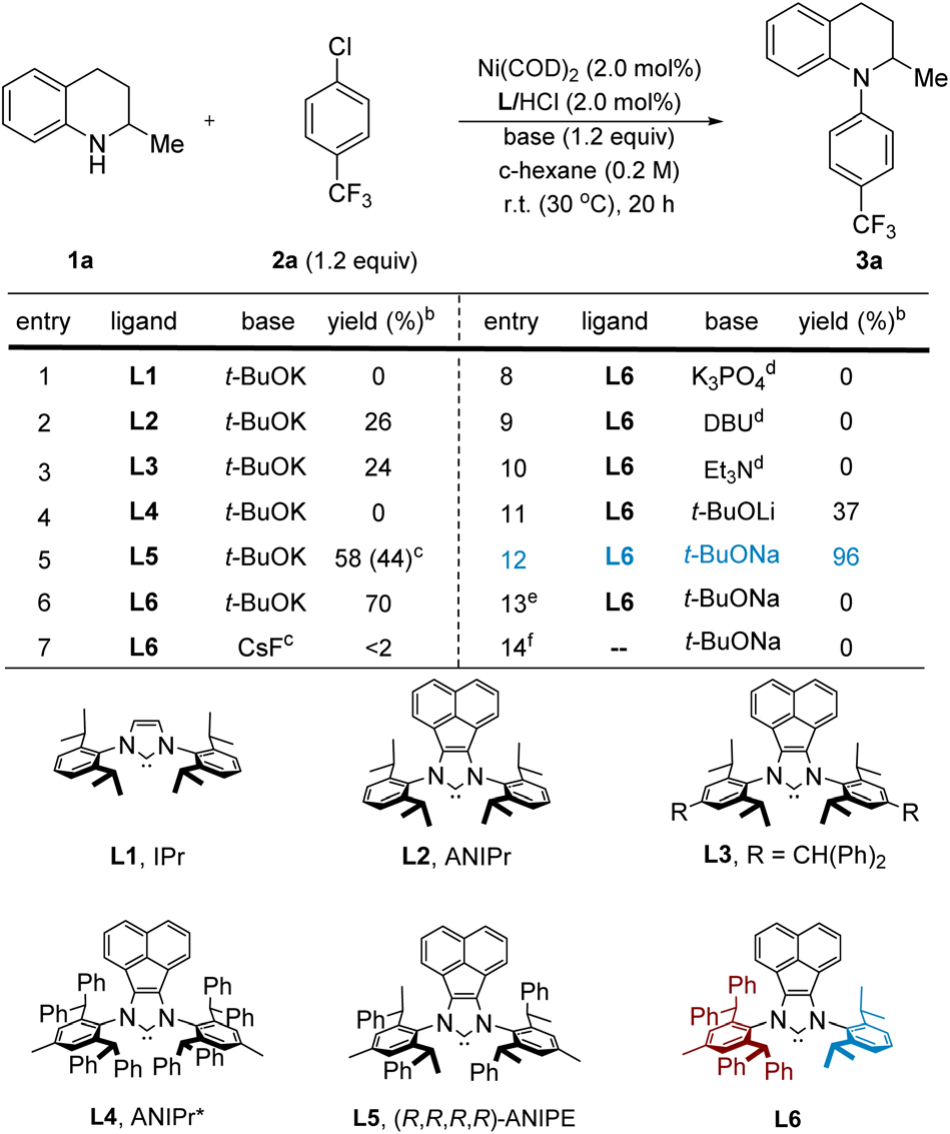

a Reactions were performed on a 0.2 mmol scale.

b Determined by NMR analysis using 1,3,5-trimethyl-benzene as an internal standard.

c Using *t*-BuONa instead of *t*-BuOK.

d 5.0 mol% *t*-BuOK was added.

e Without Ni(COD)_2_.

f Without L6.

To further improve the catalytic efficiency, the effect of the base was then studied (entries 7–12). Organic bases, such as Et_3_N and DBU,^20^ were found to be ineffective in this transformation. The use of weak inorganic bases, including CsF and K_3_PO_4_, delivered little or no 3a. To our delight, an excellent yield (96%) was obtained when *t*-BuONa was used (entry 12), in part because of better solubility in cyclohexane compared to *t*-BuOK (see the ESI† for solvent optimization details). As expected, control experiments revealed that Ni(COD)_2_ and NHC ligands (entries 13 and 14) were both essential for the C–N cross-coupling reaction.

With the optimized reaction conditions, we focused our attention on the substrate scope of this transformation with various hindered primary and secondary amines. As depicted in Table 2, our arylation method showed an excellent efficiency profile. A diverse array of C–N cross-coupling products were prepared in good to excellent yields. A series of bulky cyclic secondary amines, including tetrahydroquinoline (3a and 3b), tetrahydroisoquinoline (3f and 3g), decahydroquinoline (3h), piperidine (3i), and piperazine (3j), all served as competent substrates. Moreover, seven-membered cyclic amines (3d and 3e) could also be arylated efficiently. In addition, acyclic hindered secondary amines (3k)^6*b*^ and diarylamine (3l),^21^ which are non-trivial in Ni-catalyzed cross-coupling reactions due to their steric hindrance, gave the corresponding products in good yields. Notably, the arylation protocol was efficient for a wide variety of bulky α,α,α-trisubstituted primary amines, such as *tert*-butylamine (3m), triphenylmethylamine (3n), amantadine (3o), and *tert*-octylamine (3p).^13^ It is worth mentioning that room temperature was sufficient to carry on the reaction in some cases, highlighting the high efficiency of our protocol.

**Table 2:** Substrate Scope^a^

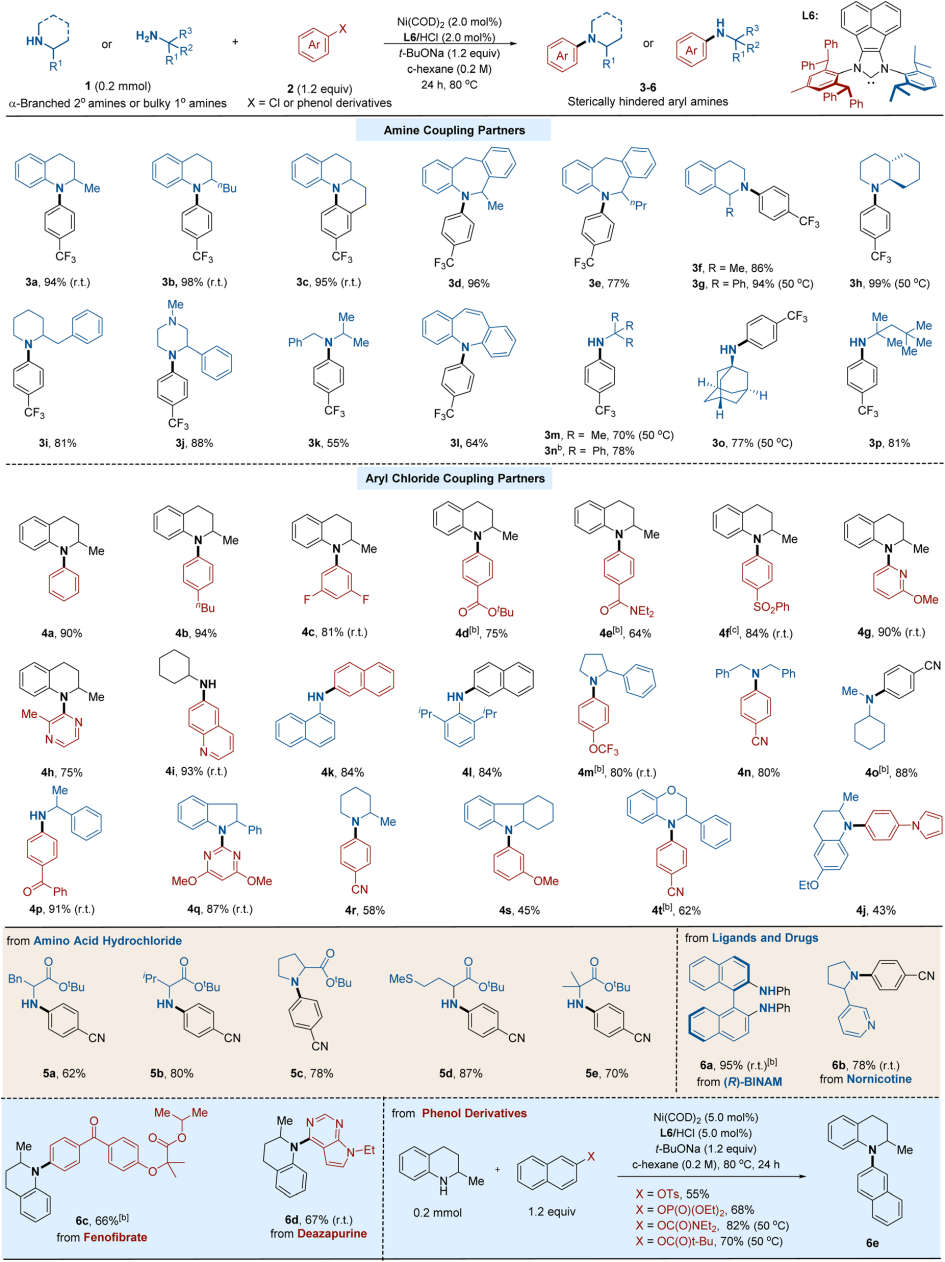

a Yields of isolated products on a 0.2 mmol scale are reported.

b 5.0 mol% Ni(COD)_2_ and L6/HCl were used.

c Cyclohexane (0.1 M).

Encouraged by the above outcomes, we moved on to explore the scope and reactivity of aryl chlorides. As shown in Table 2, a series of aryl chlorides with electron-deficient, electron-neutral, and electron-rich substituents on the aryl group are suitable substrates, affording the corresponding products in good to excellent yields. Diverse functional groups, such as fluoride (4c), ester (4d), amide (4e), sulfonyl (4f), ether (4g and 4s), cyano (4o), and trifluoromethoxy group (4m), could be well accommodated under the reaction conditions. Notably, an *ortho*-substituted aryl chloride was applicable (4h), which is challenging in metal-catalyzed *N*-arylation. Cross-couplings using heteroaryl chlorides containing pyridine (4g), pyrazine (4h), quinolone (4i), pyrrole (4j), and pyrimidine (4q) also performed efficiently. Given the wide presence of aniline and the prevalence of heterocycles in pharmaceutically relevant compounds, the current C–N cross-coupling protocol would provide a robust tool for constructing the skeleton of the biologically active molecule.

With this reliable protocol for both primary and secondary amines, we next applied this method to modify a series of ligands and bioactive compounds. We first extended this reaction to various amino acid derivatives; either primary hindered amines or secondary congeners could be arylated in good yields. Furthermore, complex ligands and drug-relevant molecules, including (*R*)-BINAM, nornicotine, fenofibrate, and deazapurine, readily undergo amination reactions, furnishing arylated products in high yields. Considering the unique reactivity of Ni catalysts towards phenol derivatives, we then turn our attention to these challenging coupling partners. A range of phenol-derived electrophiles, including aryl triflates, aryl phosphates, aryl carbamate, and aryl carbonate, which are ineffective for Ni-bisphosphine conditions,^13^ were found to be suitable substrates under the current conditions, giving moderate to high yields of aminated products.

**Fig. 2:**
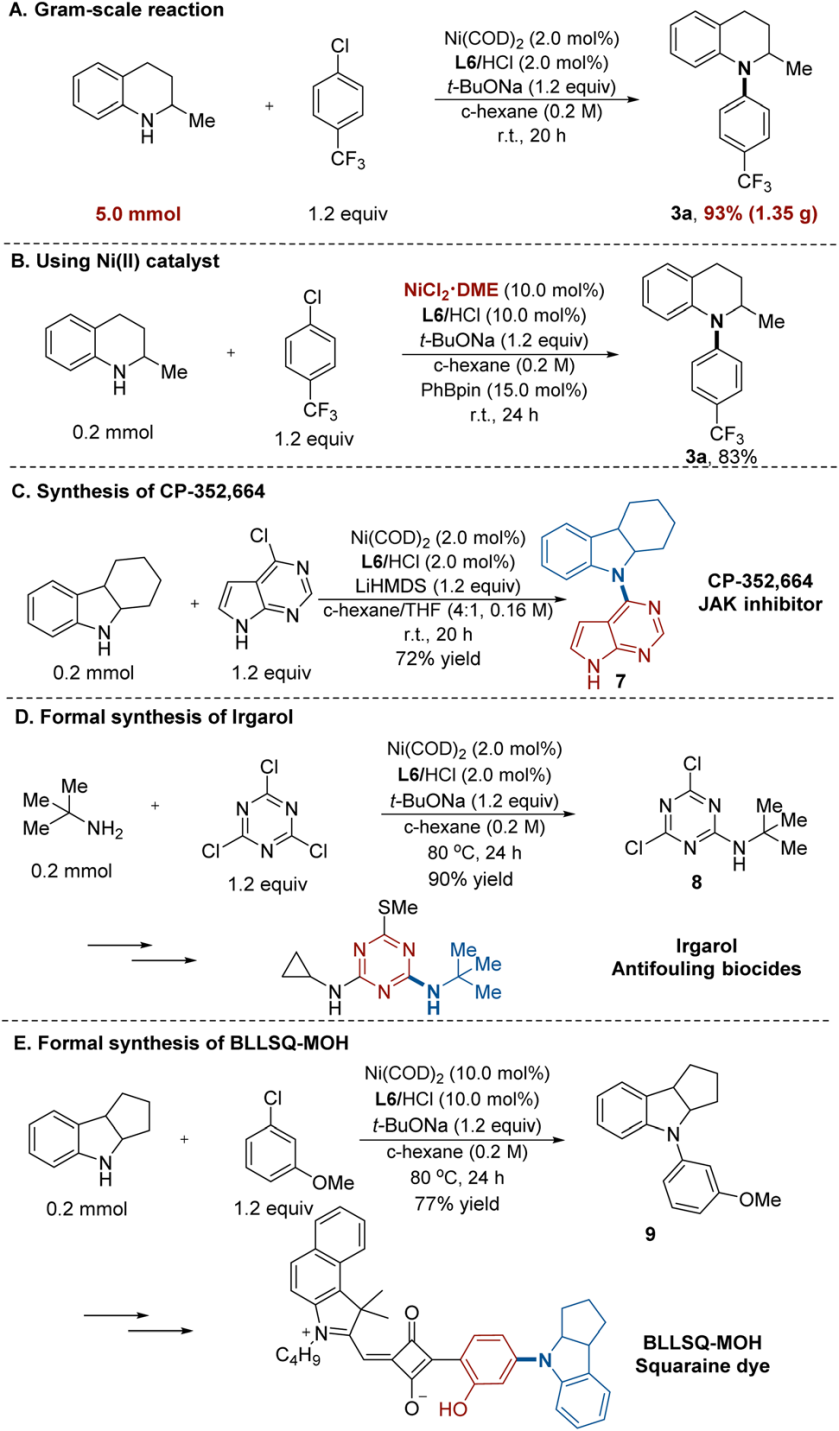
Gram-scale reaction and synthetic applications.

To demonstrate the practicality of our reaction, we conducted a 5.0 mmol–scale reaction (Fig. 2A). On this scale, product 3a was obtained in 93% yield (1.35 g). When a combination of the air-stable precatalyst NiCl_2_·DME and PhBpin was used instead of Ni(COD)_2_, product 3a was obtained with similarly high efficiency (Fig. 2B). To further show the synthetic utility of our protocol, we exemplify three applications in the synthesis of drugs, herbicides, and advanced materials. First, this Ni/NHC-catalysed process was applied to prepare CDP-352, 664 (7),^22^ a potent JAK inhibitor. The desired product was furnished in a single transformation in 72% yield (Fig. 2C). Moreover, the intermediate for irgarol^23^ (8) was prepared in 90% yield using *tert*-butylamine and 1,3,5-trichlorotriazine, without the formation of di- or triamination byproducts (Fig. 2D). In addition, we applied the amination protocol to synthesize the key intermediate (9) for BLLSQ-MOH, a squaraine dye,^24^ which was obtained in 77% yield (Fig. 2E).

**Fig. 3:**
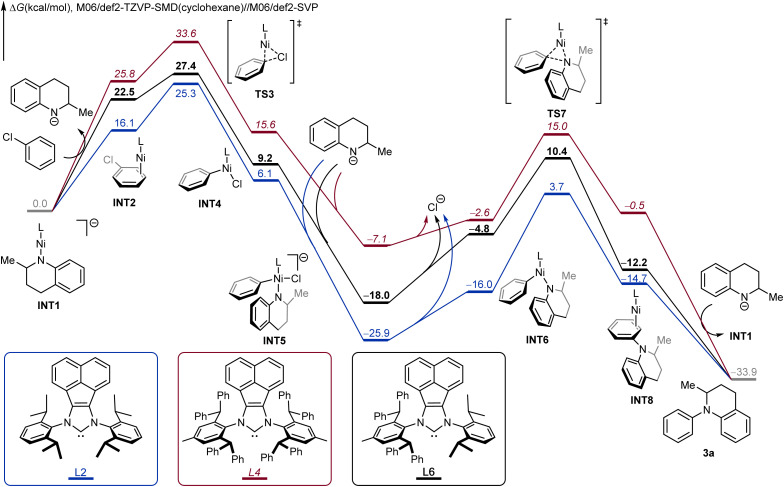
DFT-computed Gibbs free profiles of Ni-catalyzed *N*-arylation between 1,2,3,4-tetrahydroquinaldine and chlorobenzene under various NHC ligands.

We next performed density functional theory (DFT) calculations to investigate the origins of the high reactivity of the unsymmetric NHC ligand L6, using coupling between 1,2,3,4-tetrahydroquinaldine and chlorobenzene as a model. The DFT-computed free energy profiles of the coupling catalytic cycle for L2, L4 and L6 are compared in Fig. 3. From the deprotonated tetrahydroquinoline anion-coordinated complex INT1, the ligand exchange with chlorobenzene leads to the substrate-coordinated intermediate INT2. The subsequent oxidative addition *via*TS3 generates the LNi(phenyl)Cl intermediate INT4, which is further coordinated by the deprotonated tetrahydroquinoline anion to produce INT5. From INT5, the dissociation of the chloride anion leads to the neutral LNi(phenyl)(amino) intermediate INT6. INT6 undergoes three-membered ring C–N reductive elimination through TS7 to produce the product-coordinated complex INT8. The subsequent product liberation regenerates the active intermediate INT1 and releases the observed arylation product 3a. For the optimal unsymmetric NHC ligand L6 (black pathway), the oxidative addition requires an overall barrier of 27.4 kcal mol^−1^ from INT1 to TS3. Due to the stable intermediate INT5, the C–N reductive elimination is rate-determining with an overall barrier of 28.4 kcal mol^−1^.

Comparing the three free energy profiles, the computation well reproduces the trend of ligand reactivities. For L6 (black pathway), the overall reaction barrier is 28.4 kcal mol^−1^ with reductive elimination being the rate-limiting step. For the more sterically demanding ligand L4 (red pathway), the additional steric repulsions promote reductive elimination; the C–N reductive elimination requires an overall barrier of 22.1 kcal mol^−1^ from INT5 to TS7. However, the same steric repulsions also disfavor the oxidative addition, and the barrier for L4 increases to 33.6 kcal mol^−1^ from INT1 to TS3. For the less demanding ligand L2 (blue pathway), the oxidative addition step requires a barrier of 25.3 kcal mol^−1^, which is more efficient than L4; however, the reductive elimination barrier with L2 increases to 29.6 kcal mol^−1^, which is rate-determining for L2 and is 1.2 kcal mol^−1^ higher as compared to the overall reaction barrier of L4. Overall, our computations corroborated the reactivity trend of L6 > L2 > L4, which is in nice agreement with the experimental observations (Table 1). These calculations highlight the important role of tuning the steric repulsions by the unsymmetric NHC ligand, which is able to achieve the desired balance of the contrasting steric effects on oxidative addition and reductive elimination.

## Conclusions

In conclusion, we have developed a highly efficient unprecedented Ni-catalysed *N*-arylation of sterically hindered primary and secondary amines with aryl chlorides or phenol derivatives. This protocol exhibits broad substrate scope and excellent functional group compatibility, proceeds under mild reaction conditions, and is applicable in late-stage functionalization. Computational studies reveal that the unsymmetric, bulky yet flexible NHC ligand was the key to the success of this challenging transformation, which allowed for well-balanced rates for the elementary catalytic steps. We anticipate that this Ni/NHC-catalysed protocol would offer a general and powerful platform for C–N cross-couplings in medicinal research and chemical synthesis. We expect that this intriguing discovery would inspire further design of sterically flexible ligands to promote other challenging metal-catalysed cross-coupling reactions *via* balancing the elementary steps.

## Data availability

General experimental conditions, calculations related to the mechanism elucidation, and characterization of all compounds synthesized are available in the ESI.†

## Author contributions

Z.-C. W. developed the catalytic system and performed the reaction optimization and scope study, Y.-Y. Li and S.-Q. Zhang conducted the DFT calculations under the supervision of X. H., S.-L. S., X. H. and Z.-C. W. wrote the manuscript with inputs from the other authors. S.-L. S. conceived and directed the project.

## Conflicts of interest

There are no conflicts to declare.

## Supplementary Material

SC-014-D3SC00492A-s001
