# Supplementary material for: Unsymmetric N-heterocyclic carbene ligand enabled nickel-catalysed arylation of bulky primary and secondary amines
Source: Chem Sci. 2023 Mar 28;14(16):4390–6. doi: 10.1039/d3sc00492a (PMC10132161; doi:10.1039/d3sc00492a)
Supplement: SC-014-D3SC00492A-s001 [file SC-014-D3SC00492A-s001.pdf]

Electronic Supplementary Material (ESI) for Chemical Science.
This journal is © The Royal Society of Chemistry 2023

Supporting Information

Unsymmetrical *N*-Heterocyclic Carbene Ligand Enabled Nickel-Catalysed Arylation of Bulky Primary and Secondary Amines

Zi-Chao Wang[a], Yan-Yu Li[b], Shuo-Qing Zhang[b], Xin Hong*[b,c,d] and Shi-Liang Shi*[a]

[a] State Key Laboratory of Organometallic Chemistry, Shanghai Institute of Organic Chemistry, University of Chinese Academy of Sciences, Chinese Academy of Sciences, 345 Lingling Road, Shanghai 200032, China

E-mail: shiliangshi@sioc.ac.cn

[b] Department of Chemistry, Zhejiang University, 38 Zheda Road, Hangzhou 310027, China

[c] Beijing National Laboratory for Molecular Sciences, Zhongguancun North First Street NO. 2, Beijing 100190, China

[d] Key Laboratory of Precise Synthesis of Functional Molecules of Zhejiang Province, School of Science, Westlake University, 18 Shilongshan Road, Hangzhou 310024, Zhejiang Province, China

Table of contents

General Information 2

Reaction Optimization 3

Nickel/NHC-Catalyzed Arylation of Sterically Hindered Amines 5

DFT Calculations of the Ni-Catalyzed *N*-Arylation 32

References 147

NMR Spectra 149

General Information

All the reactions were conducted under N_2 atmosphere with a standard dry box. The glassware was dried in an oven (140 °C) or flame-dried. Reaction solvents including anhydrous cyclohexane, tetrahydrofuran (THF), *N*,*N*-dimethylformamide (DMA), toluene, etc., were purchased from Energy Chemical Co. Ltd. or J&K Chemical Co. Ltd. and used as received. $Ni(COD)_2$ was purchased from Strem Chemicals Inc. and stored in the refrigerator at -20 ℃. 1,2,3,4-tetrahydroquinaldine was purchased from Energy Chemical Co. Ltd. and used as received. 4-chlorobenzotrifluoride was purchased from Energy Chemical Co. Ltd. and used as received. All new compounds were characterized by NMR spectroscopy, IR spectroscopy, high-resolution mass spectroscopy, and melting point (if solids). NMR spectra were recorded on an Agilent 400 MHz or 600MHz, Varian 400 MHz, or Bruker 400 MHz spectrometers and were calibrated using residual solvent as an internal reference ($CDCl_3$: 7.26 ppm for ^1H NMR and 77.16 ppm for ^{13}C NMR). Melting points were measured on an SGW X-4 apparatus. All IR spectra were taken on a BRUKER TENSOR 27 FT-IR spectrometer. EI-HRMS spectra were obtained on a Waters Micromass G1540N/GCT Premier, and ESI-HRMS spectra were obtained on a Thermo Fisher Scientific LTQ FT Ultra or Agilent Technologies 6224 TOF LC/MS.

Reaction Optimization

Table 1. Ligand and Base Optimization[a]

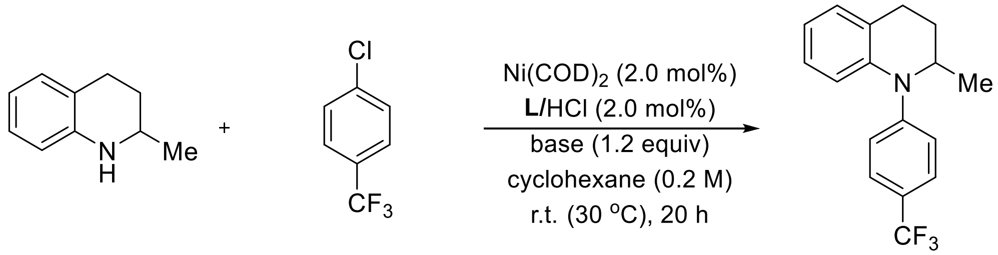

1a **2a** (1.2 equiv) **3a**

Entry	Ligand	Base	Yield (%)[b]	Entry	Ligand	Base	Yield (%)[b]
1	**L1**	*t*-BuOK	0	8	**L6**	CsF[d]	<2%
2	**L2**	*t*-BuOK	26	9	**L6**	K_3PO_4[d]	0
3	**L3**	*t*-BuOK	24	10	**L6**	DBU[d]	0
4	**L4**	*t*-BuOK	0	11	**L6**	Et_3N[d]	0
5	**L5**	*t*-BuOK	58	12	**L6**	*t*-BuOLi	37
6[c]	**L5**	*t*-BuOK	70	**13**	**L6**	***t*-BuONa**	**96**
7	**L6**	*t*-BuOK	70	14[e]	**L6**	*t*-BuONa	0

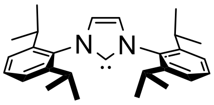

L1, IPr

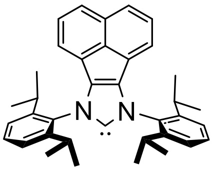

L2

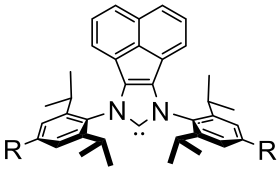

L3, R = CH(Ph)$_2$

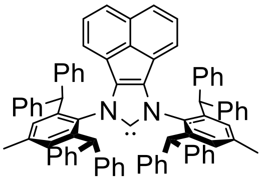

L4

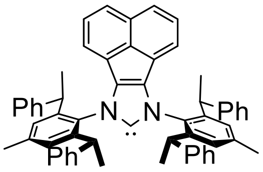

L5, (*R*,*R*,*R*,*R*)-ANIPE

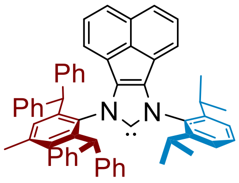

L6

[a] Reactions were performed on a 0.2-mmol scale. [b] Determined by NMR analysis using 1,3,5-trimethyl-benzene as an internal standard. [c] 50 °C. [d] 5.0 mol% *t*-BuOK was added. [e] Without Ni(COD)$_2$ or **L6**.

Table 2. Solvent Screening[a]

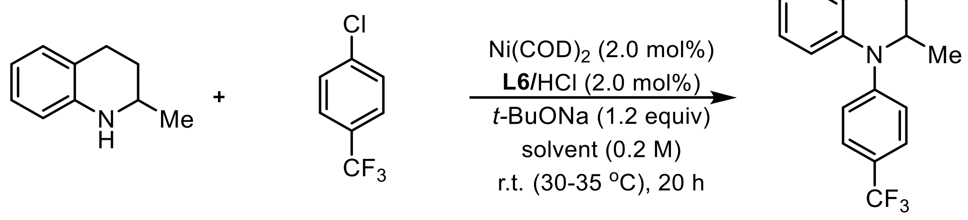

1 **2** (1.2 equiv) **3**

Entry	Solvent	Yield (%)[b]	Entry	Solvent	Yield (%)[b]
1	cyclohexane	96	4	$PhCF_3$	31
2	THF	24	5	DMA	0
3	toluene	39	6	DME	29

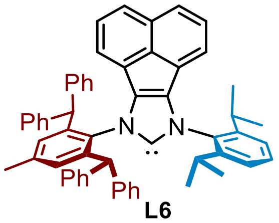

L6

[a] Reactions were performed on a 0.2-mmol scale. [b] Determined by NMR analysis using 1,3,5-trimethyl-benzene as an internal standard.

Table 3. Other Ligands[a]

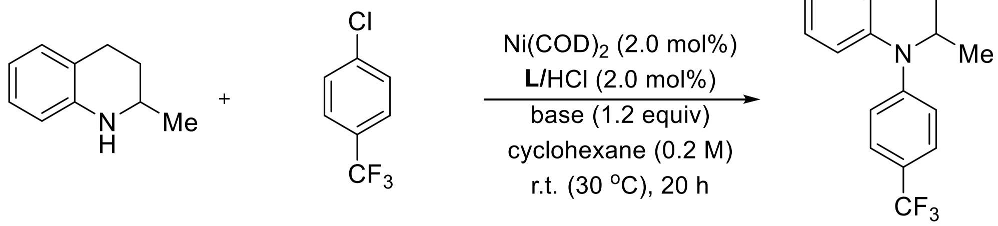

1a **2a** (1.2 equiv) **3a**

Entry	Ligand	Base	Yield (%)[b]	Entry	Ligand	Base	Yield (%)[b]
1	**L7**	*t*-BuOK	0	8	**L9**	*t*-BuOK	0
2	**L8**	*t*-BuOK	0	9	**L10**	*t*-BuOK	0

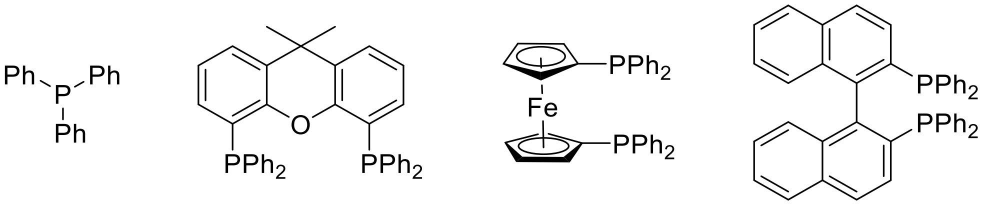

L7 **L8** **L9** **L10**

[a] Reactions were performed on a 0.2-mmol scale. [b] Determined by NMR analysis using 1,3,5-trimethyl-benzene as an internal standard.

Nickel/NHC-Catalyzed Arylation of Sterically Hindered Amines

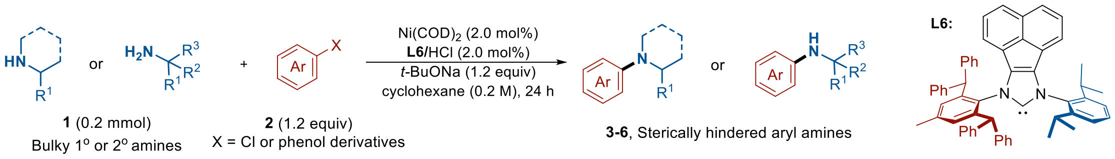

General Procedure: In a nitrogen-filled glove box, an oven-dried screw-cap reaction tube equipped with a magnetic stir bar was charged with $Ni(COD)_2$ (1.1 mg, 4 µmol, 2 mol%), **L6**/HCl (3.3 mg, 4 µmol, 2 mol%), *t*-BuONa (23 mg, 0.24 mmol, 1.2 equiv) and cyclohexane (1.0 ml, 0.2 M). The reaction tube was sealed with a screw-cap septum and stirred at room temperature (r.t.) for 20 min until the color turned brown. The amine (0.2 mmol) and aryl chloride (0.24 mmol, 1.2 equiv) were then added. The reaction tube was wrapped with electrical tape and removed from the glove box. The reaction mixture was stirred within the sealed tube at temperature as indicated for each compound for 24 h, followed by adding a saturated aqueous solution of NaCl (2.0 mL) and EtOAc (2.0 mL). The phases were separated, and the aqueous layer was extracted with EtOAc (2 x 5.0 mL). The combined organic phases were washed with a saturated aqueous solution of NaCl (2 x 10 mL) and then concentrated in vacuo. The crude residue was purified via column chromatography to afford the desired product.

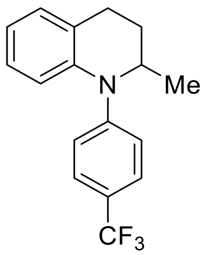

2-methyl-1-(4-(trifluoromethyl)phenyl)-1,2,3,4-tetrahydroquinoline (3a)[1]

Following the General Procedure using 1,2,3,4-tetrahydro-2-methylquinoline (29.4 mg, 0.2 mmol, 1.0 equiv) and 4-chlorobenzotrifluoride (43.3 mg, 0.24 mmol, 1.2 equiv), the reaction mixture was stirred at r.t. for 24 h. The crude product was purified by column chromatography (0-2% EtOAc in hexanes) to provide the title compound as a yellow oil in 94% yield (55 mg). ^{1}H NMR (400 MHz, $CDCl_3$) δ 7.5 (d, *J* = 8.5 Hz, 2H), 7.3 (d, *J* = 8.4 Hz, 2H), 7.1 (d, *J* = 7.3

Hz, 1H), 7.0 (m, 1H), 6.9 (m, 1H), 6.8 (m, 1H), 4.1 (m, 1H), 2.9 (m, 1H), 2.8 (m, 1H), 2.1 (m, 1H), 1.8 (m, 1H), 1.3 (d, J = 6.6 Hz, 3H). ^{13}C NMR (101 MHz, $CDCl_3$) δ 151.2, 141.8, 129.6, 126.6 (q, J = 4.0 Hz), 126.5, 126.1, 124.6 (q, J = 272.7 Hz), 124.0 (q, J = 33.3 Hz), 122.9, 120.1, 118.7, 54.1, 28.5, 23.8, 19.1. ^{19}F NMR (376 MHz, $CDCl_3$) δ -61.2.

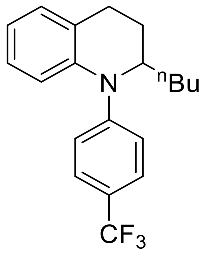

2-butyl-1-(4-(trifluoromethyl)phenyl)-1,2,3,4-tetrahydroquinoline (3b) [1]

Following the General Procedure using 2-butyl-1,2,3,4-tetrahydroquinoline (37.9 mg, 0.2 mmol, 1.0 equiv) and 4-chlorobenzotrifluoride (43.3 mg, 0.24 mmol, 1.2 equiv), the reaction mixture was stirred at r.t. for 24 h. The crude product was purified by column chromatography (0-2% EtOAc in hexanes) to provide the title compound as a yellow oil in 98% yield (65 mg). ^{1}H NMR (400 MHz, $CDCl_3$) δ 7.5 (d, J = 8.6 Hz, 2H), 7.2 (d, J = 8.5 Hz, 2H), 7.1 (d, J = 7.4 Hz, 1H), 7.0 (m, 2H), 6.9 (m, 1H), 3.9 (m, 1H), 2.8 (m, 2H), 2.0 (m, 1H), 1.9 (m, 1H), 1.7 (m, 1H), 1.4 (m, 5H), 0.9 (t, J = 7.2 Hz, 3H). ^{13}C NMR (101 MHz, $CDCl_3$) δ 152.4, 141.2, 129.7, 127.5, 126.4 (q, J = 4.0 Hz), 126.4, 124.7 (q, J = 271.7 Hz), 122.9 (q, J = 32.3 Hz), 121.3, 121.1, 121.1, 59.2, 31.7, 28.9, 25.2, 23.3, 22.9, 14.3. ^{19}F NMR (376 MHz, $CDCl_3$) δ -61.6.

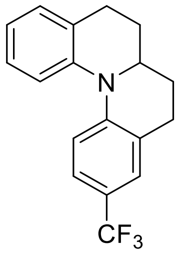

3-(trifluoromethyl)-6,6a,7,8-tetrahydro-5H-quinolino[1,2-a]quinolone (3c)

Following the General Procedure using 2-(2-chloro-5-(trifluoromethyl)phenethyl)-1,2,3,4-tetrahydroquinoline (68.0 mg, 0.2 mmol, 1.0 equiv), the reaction mixture was stirred at r.t. for 24 h. The crude product was purified by column chromatography (0-2% EtOAc in hexanes) to

provide the title compound as a yellow oil in 95% yield (58 mg).

^{1}H NMR (400 MHz, $CDCl_3$) δ 7.3 (m, 3H), 7.2 (m, 1H), 7.1 (d, J = 7.5 Hz, 1H), 7.0 (m, 1H), 6.9 (t, J = 7.4 Hz, 1H), 3.5 (m, 1H), 2.8 (m, 4H), 2.3 (m, 1H), 2.2 (m, 1H), 1.6 (m, 2H). ^{13}C NMR (101 MHz, $CDCl_3$) δ 145.6, 141.7, 129.6, 129.3, 128.8, 126.1, 125.9 (q, J = 4.0 Hz), 124.9 (q, J = 272.7 Hz), 123.5 (q, J = 4.0 Hz), 121.4, 121.0 (q, J = 32.3Hz), 119.3, 117.3, 56.0, 31.0, 30.8, 27.4, 27.2. ^{19}F NMR (376 MHz, $CDCl_3$) δ -61.4. IR (neat, cm^{-1}) 2927, 1736, 1505, 1310, 1111, 751. HRMS (EI) calculated for $C_{18}H_{16}F_3N$ [M]$^+$ m/z 303.1229, found 303.1225.

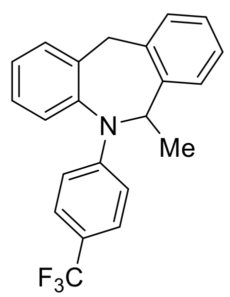

6-methyl-5-(4-(trifluoromethyl)phenyl)-6,11-dihydro-5H-dibenzo[b,e]azepine (3d) [1]

Following the General Procedure using 6-methyl-6,11-dihydro-*5H*-dibenzo[b,e]azepine (41.9 mg, 0.2 mmol, 1.0 equiv) and 4-chlorobenzotrifluoride (43.3 mg, 0.24 mmol, 1.2 equiv), the reaction mixture was stirred at 80 °C for 24 h. The crude product was purified by column chromatography (0-2% EtOAc in hexanes) to provide the title compound as a yellow oil in 96% yield (68 mg).

^{1}H NMR (400 MHz, $CDCl_3$) δ 7.3 (m, 6H), 7.2 (m, 2H), 7.1 (m, 2H), 6.7 (d, J = 8.7 Hz, 2H), 5.4 (m, 1H), 4.1 (d, J = 15.6 Hz, 1H), 3.5 (d, J = 15.7 Hz, 1H), 1.3 (d, J = 7.0 Hz, 3H). ^{13}C NMR (101 MHz, $CDCl_3$) δ 150.9, 141.4, 141.2, 140.7, 134.3, 131.5, 130.8, 130.4, 129.3, 128.2, 127.9, 127.1, 126.9, 126.6 (q, J = 4.0 Hz), 125.1 (q, J = 271.7 Hz), 119.3 (q, J = 33.3 Hz), 112.5, 55.0, 40.1, 25.4. ^{19}F NMR (376 MHz, $CDCl_3$) δ -61.0.

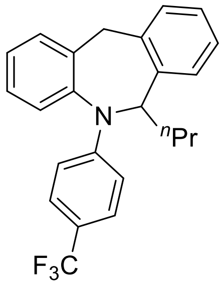

6-isopropyl-5-(4-(trifluoromethyl)phenyl)-6,11-dihydro-5H-dibenzo[b,e]azepine (3e)

Following the General Procedure using 6-propyl-6,11-dihydro-*5H*-dibenzo[b,e]azepine (47.4 mg, 0.2 mmol, 1.0 equiv) and 4-chlorobenzotrifluoride (43.3 mg, 0.24 mmol, 1.2 equiv), the reaction mixture was stirred at 80 ºC for 24 h. The crude product was purified by column chromatography (0-2% EtOAc in hexanes) to provide the title compound as a yellow oil in 77% yield (59 mg).

^{1}H NMR (400 MHz, $CDCl_3$) δ 7.4 (d, J = 7.1 Hz, 1H), 7.3 (m, 5H), 7.2 (m, 2H), 7.1 (m, 2H), 6.7 (d, J = 8.6 Hz, 2H), 5.2 (m, 1H), 4.1 (d, J = 15.8 Hz, 1H), 3.5 (d, J = 15.9 Hz, 1H), 1.6 (m, 1H), 1.5 (m, 3H), 0.9 (t, J = 7.0 Hz, 3H). ^{13}C NMR (101 MHz, $CDCl_3$) δ 151.5, 141.2, 140.7, 140.6, 134.4, 130.8, 130.7, 130.6, 129.8, 128.2, 127.8, 127.1, 126.7, 126.6 (q, J = 4.0 Hz), 125.1 (q, J = 271.7 Hz), 119.1 (q, J = 32.3 Hz), 112.4, 60.1, 41.0, 40.3, 20.2, 13.9. ^{19}F NMR (376 MHz, $CDCl_3$) δ -61.0. IR (neat, cm^{-1}) 2959, 1737, 1616, 1324, 1254, 1110. HRMS (EI) calculated for $C_{24}H_{22}F_3N$ $[M]^+$ m/z 381.1699, found 381.1707.

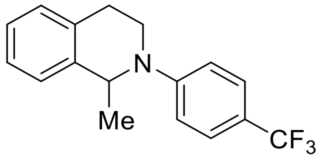

1-methyl-2-(4-(trifluoromethyl)phenyl)-1,2,3,4-tetrahydroisoquinoline (3f)

Following the General Procedure using 1-methyl-1,2,3,4-tetrahydroisoquinoline (29.4 mg, 0.2 mmol, 1.0 equiv) and 4-chlorobenzotrifluoride (43.3 mg, 0.24 mmol, 1.2 equiv), the reaction mixture was stirred at 80 ºC for 24 h. The crude product was purified by column chromatography (0-2% EtOAc in hexanes) to provide the title compound as a yellow oil in 86% yield (58 mg).

^{1}H NMR (400 MHz, $CDCl_3$) δ 7.5 (d, *J* = 8.8 Hz, 2H), 7.2 (m, 4H), 6.9 (d, *J* = 8.8 Hz, 2H), 5.0

(q, J = 6.7 Hz, 1H), 3.7 (m, 1H), 3.6 (m, 1H), 3.0 (m, 2H), 1.5 (d, J = 6.7 Hz, 3H). ^{13}C NMR (101 MHz, $CDCl_3$) δ 151.2, 139.5, 134.4, 128.6, 126.8, 126.8, 126.7 (q, J = 4.0 Hz), 125.2 (q, J = 271.7 Hz), 126.5, 118.2 (q, J = 33.3 Hz), 112.3, 54.1, 41.4, 28.5, 21.3. ^{19}F NMR (376 MHz, $CDCl_3$) δ -60.9. IR (neat, cm^{-1}) 2959, 1708, 1638, 1375, 1154, 885. HRMS (ESI) calculated for $C_{17}H_{17}F_3N$ [M+H]$^+$ m/z 292.1308, found 292.1314.

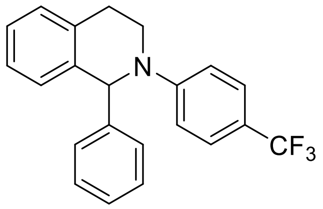

1-phenyl-2-(4-(trifluoromethyl)phenyl)-1,2,3,4-tetrahydroisoquinoline (3g) [1]

Following the General Procedure using 1-phenyl-1,2,3,4-tetrahydroisoquinoline (41.8 mg, 0.2 mmol, 1.0 equiv) and 4-chlorobenzotrifluoride (43.3 mg, 0.24 mmol, 1.2 equiv), the reaction mixture was stirred at 50 °C for 24 h. The crude product was purified by column chromatography (0-2% EtOAc in hexanes) to provide the title compound as a yellow oil in 94% yield (66 mg).

^{1}H NMR (400 MHz, $CDCl_3$) δ 7.4 (d, J = 8.7 Hz, 2H), 7.3 (m, 1H), 7.2 (m, 8H), 6.8 (d, J = 8.8 Hz, 2H), 5.9 (s, 1H), 3.8 (m, 1H), 3.5 (m, 1H), 2.9 (m, 2H). ^{13}C NMR (101 MHz, $CDCl_3$) δ 151.5, 142.1, 137.7, 135.6, 128.6, 128.1, 127.7, 127.6, 127.2, 126.9, 126.64, 126.62, 126.6 (q, J = 4.0 Hz), 126.5 (q, J = 271.7 Hz), 112.2, 62.5, 44.3, 28.1. ^{19}F NMR (376 MHz, $CDCl_3$) δ -60.8.

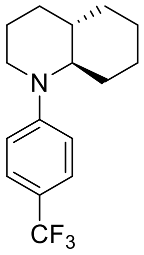

***trans*-1-(4-(trifluoromethyl)phenyl)decahydroquinoline (3h)** [1]

Following the General Procedure using (4aS,8aR)-decahydroquinoline (27.8 mg, 0.2 mmol, 1.0 equiv) and 4-chlorobenzotrifluoride (43.3 mg, 0.24 mmol, 1.2 equiv), the reaction mixture was

stirred at 50 °C for 24 h. The crude product was purified by column chromatography (0-2% EtOAc in hexanes) to provide the title compound as a yellow oil in 99% yield (56 mg).

^{1}H NMR (400 MHz, $CDCl_3$) δ 7.4 (d, J = 8.4 Hz, 2H), 7.1 (d, J = 8.2 Hz, 2H), 3.1 (m, 1H), 2.7 (m, 1H), 2.4 (m, 1H), 1.6 (m, 7H), 1.3 (m, 1H), 1.2 (m, 2H), 1.0 (m, 3H). ^{13}C NMR (101 MHz, $CDCl_3$) δ 155.7, 126.0 (q, J = 4.0 Hz), 125.5 (q, J = 33.3 Hz), 124.6 (q, J = 272.7 Hz), 124.4, 65.1, 56.4, 42.2, 33.2, 31.8, 31.4, 26.3, 26.1, 25.5. ^{19}F NMR (376 MHz, $CDCl_3$) δ -61.9. IR (neat, cm^{-1}) 2926, 1737, 1613, 1325, 1122, 1068. HRMS (ESI) calculated for $C_{16}H_{21}F_3N$ [M+H]$^+$ m/z 284.1621, found 284.1629.

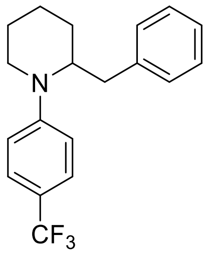

2-benzyl-1-(4-(trifluoromethyl)phenyl)piperidine (3i)

Following the General Procedure using 2-benzylpiperidine (36.0 mg, 0.2 mmol, 1.0 equiv) and 4-chlorobenzotrifluoride (43.3 mg, 0.24 mmol, 1.2 equiv), the reaction mixture was stirred at 80 °C for 24 h. The crude product was purified by column chromatography (0-2% EtOAc in hexanes) to provide the title compound as a yellow oil in 81% yield (54 mg).

^{1}H NMR (400 MHz, $CDCl_3$) δ 7.5 (m, 2H), 7.3 (m, 2H), 7.2 (m, 3H), 6.9 (d, J = 8.8 Hz, 2H), 4.2 (m, 1H), 3.6 (m, 1H), 3.1 (m, 1H), 2.9 (m 1H), 2.7 (m, 1H), 1.7 (m, 6H). ^{13}C NMR (101 MHz, $CDCl_3$) δ 153.0, 139.6, 129.2, 128.7, 126.6 (q, J = 4.0 Hz), 125.1 (q, J = 271.7Hz), 119.2 (q, J = 33.3 Hz), 126.3, 114.4, 57.2, 42.6, 33.6, 26.3, 25.5, 18.7. ^{19}F NMR (376 MHz, $CDCl_3$) δ -61.1. IR (neat, cm^{-1}) 2931, 1614, 1522, 1328, 1254, 1110. HRMS (ESI) calculated for $C_{19}H_{21}F_3N$ [M+H]$^+$ m/z 320.1621, found 320.1628.

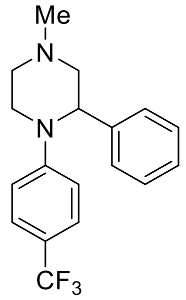

4-methyl-2-phenyl-1-(4-(trifluoromethyl)phenyl)piperazine (3j)

Following the General Procedure using 1-methyl-3-phenylpiperazine (35.2 mg, 0.2 mmol, 1.0 equiv) and 4-chlorobenzotrifluoride (43.3 mg, 0.24 mmol, 1.2 equiv), the reaction mixture was stirred at 80 °C for 24 h. The crude product was purified by column chromatography (5-10% EtOAc in hexanes) to provide the title compound as a yellow oil in 88% yield (56 mg).

^{1}H NMR (400 MHz, $CDCl_3$) δ 7.4 (m, 2H), 7.2 (m, 4H), 7.2 (m, 1H), 6.9 (m, 2H), 4.6 (m, 1H), 3.6 (m, 1H), 3.4 (m, 1H), 2.7 (m, 3H), 2.6 (m, 1H), 2.3 (s, 3H). ^{13}C NMR (101 MHz, $CDCl_3$) δ 153.4, 140.8, 128.6, 127.5, 127.1, 126.2 (q, J = 4.0 Hz), 124.8 (q, J = 271.7 Hz), 121.4 (q, J = 33.3 Hz), 117.9, 62.1, 59.7, 55.4, 49.5, 46.3. ^{19}F NMR (376 MHz, $CDCl_3$) δ -61.5. IR (neat, cm^{-1}) 2939, 1614, 1452, 1328, 1112, 700. HRMS (ESI) calculated for $C_{18}H_{20}F_3N_2$ [M+H]$^+$ m/z 321.1573, found 321.1581.

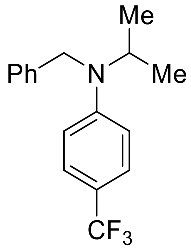

N-benzyl-N-isopropyl-4-(trifluoromethyl)aniline (3k)

Following the General Procedure using N-benzylpropan-2-amine (29.8 mg, 0.2 mmol, 1.0 equiv) and 4-chlorobenzotrifluoride (43.3 mg, 0.24 mmol, 1.2 equiv), the reaction mixture was stirred at 80 °C for 24 h. The crude product was purified by column chromatography (0-2% EtOAc in hexanes) to provide the title compound as a yellow oil in 55% yield (32 mg).

^{1}H NMR (400 MHz, $CDCl_3$) δ 7.4 (m, 2H), 7.3 (m, 2H), 7.2 (m, 3H), 6.7 (d, J = 8.8 Hz, 2H), 4.5 (s, 2H), 4.3 (m, 1H), 1.2 (d, J = 6.6 Hz, 6H). ^{13}C NMR (101 MHz, $CDCl_3$) δ 151.5, 139.6, 128.8, 126.9, 126.6 (q, J = 271.7 Hz), 126.5 (q, J = 4.0 Hz), 126.2, 117.8 (q, J = 32.3 Hz), 112.2,

48.5, 48.2, 20.0. ^{19}F NMR (376 MHz, $CDCl_3$) δ -60.9. IR (neat, cm^{-1}) 2974, 1737, 1614, 1325, 1108, 726. HRMS (ESI) calculated for $C_{17}H_{19}F_3N$ $[M+H]^+$ m/z 294.1464, found 294.1471.

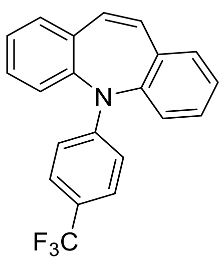

5-(4-(trifluoromethyl)phenyl)-5H-dibenzo[b,f]azepine (3l)

Following the General Procedure using 5H-dibenzo[b,f]azepine (38.6 mg, 0.2 mmol, 1.0 equiv) and 4-chlorobenzotrifluoride (43.3 mg, 0.24 mmol, 1.2 equiv), the reaction mixture was stirred at 80 ºC for 24 h. The crude product was purified by column chromatography (0-2% EtOAc in hexanes) to provide the title compound as a yellow oil in 64% yield (43 mg).

^{1}H NMR (400 MHz, $CDCl_3$) δ 7.5 (m, 6H), 7.4 (m, 2H), 7.2 (m, 2H), 6.8 (s, 2H), 6.3 (m, 2H). ^{13}C NMR (101 MHz, $CDCl_3$) δ 151.4, 142.2, 136.1, 130.7, 130.6, 130.1, 130.1, 127.7, 126.0 (q, J = 4.0 Hz), 125.0 (q, J = 270.7 Hz), 119.9 (q, J = 32.3 Hz), 111.5. ^{19}F NMR (376 MHz, $CDCl_3$) δ -61.1. IR (neat, cm^{-1}) 2926, 1736, 1613, 1316, 1236, 1044. HRMS (EI) calculated for $C_{21}H_{14}F_3N$ $[M]^+$ m/z 337.1073, found 337.1081.

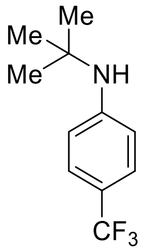

N-(tert-butyl)-4-(trifluoromethyl)aniline (3m) [2]

Following the General Procedure using 2-methylpropan-2-amine (14.6 mg, 0.2 mmol, 1.0 equiv) and 4-chlorobenzotrifluoride (43.3 mg, 0.24 mmol, 1.2 equiv), the reaction mixture was stirred at 50 ºC for 24 h. The crude product was purified by column chromatography (0-2% EtOAc in hexanes) to provide the title compound as a yellow oil in 70% yield (30 mg).

^{1}H NMR (400 MHz, $CDCl_3$) δ 7.4 (d, J = 8.6 Hz, 2H), 6.7 (d, J = 8.5 Hz, 2H), 1.4 (s, 9H). ^{13}C

NMR (101 MHz, $CDCl_3$) δ 149.4, 126.4 (q, *J* = 4.0 Hz), 126.5 (q, *J* = 271.7 Hz), 114.7, 51.6, 29.7. ^{19}F NMR (376 MHz, $CDCl_3$) δ -61.1.

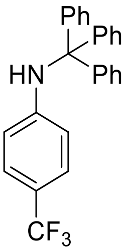

4-(trifluoromethyl)-N-tritylaniline (3n) [3]

Following the General Procedure using triphenylmethanamine (51.8 mg, 0.2 mmol, 1.0 equiv) and 4-chlorobenzotrifluoride (43.3 mg, 0.24 mmol, 1.2 equiv), the reaction mixture was stirred at 80 ºC for 24 h. The crude product was purified by column chromatography (0-2% EtOAc in hexanes) to provide the title compound as a yellow oil in 78% yield (63 mg).

^{1}H NMR (400 MHz, $CDCl_3$) δ 7.3 (m, 15H), 7.2 (d, *J* = 8.5 Hz, 2H), 6.4 (d, *J* = 8.4 Hz, 2H), 5.4 (s, 1H).^{13}C NMR (101 MHz, $CDCl_3$) δ 149.1, 144.8, 129.2, 128.3, 127.3, 125.7 (q, *J* = 4.0 Hz), 125.0 (q, *J* = 271.7 Hz), 119.0 (q, *J* = 33.3 Hz), 115.3, 71.5. ^{19}F NMR (376 MHz, $CDCl_3$) δ -61.1.

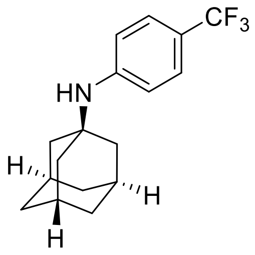

(3s,5s,7s)-N-(4-(trifluoromethyl)phenyl)adamantan-1-amine (3o) [4]

Following the General Procedure using (3s,5s,7s)-adamantan-1-amine (30.2 mg, 0.2 mmol, 1.0 equiv) and 4-chlorobenzotrifluoride (43.3 mg, 0.24 mmol, 1.2 equiv), the reaction mixture was stirred at 50 ºC for 24 h. The crude product was purified by column chromatography (0-2% EtOAc in hexanes) to provide the title compound as a yellow oil in 77% yield (46 mg).

^{1}H NMR (400 MHz, $CDCl_3$) δ 7.4 (d, *J* = 8.5 Hz, 2H), 6.8 (d, *J* = 8.4 Hz, 2H), 2.1 (m, 3H), 2.0 (d, *J* = 2.9 Hz, 6H), 1.7 (d, *J* = 14.7 Hz, 6H). ^{13}C NMR (101 MHz, $CDCl_3$) δ 149.1, 126.3 (q, *J*

= 4.0 Hz), 125.1 (q, J = 271.7 Hz), 119.1 (q, J = 32.3Hz) 115.6, 52.4, 42.8, 36.5, 29.7. ^{19}F NMR (376 MHz, $CDCl_3$) δ -61.1.

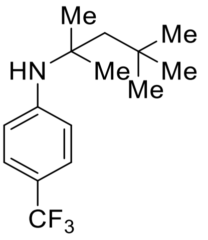

4-(trifluoromethyl)-N-(2,4,4-trimethylpentan-2-yl)aniline (3p)

Following the General Procedure using 2,4,4-trimethylpentan-2-amine (25.8 mg, 0.2 mmol, 1.0 equiv) and 4-chlorobenzotrifluoride (43.3 mg, 0.24 mmol, 1.2 equiv), the reaction mixture was stirred at 80 °C for 24 h. The crude product was purified by column chromatography (0-2% EtOAc in hexanes) to provide the title compound as a yellow oil in 81% yield (44 mg).

^{1}H NMR (400 MHz, $CDCl_3$) δ 7.4 (m, 2H), 6.6 (m, 2H), 4.0 (s, 1H), 1.7 (s, 2H), 1.4 (s, 6H), 1.0 (s, 9H). ^{13}C NMR (101 MHz, $CDCl_3$) δ 149.7, 126.4 (q, J = 4.0 Hz), 125.2 (q, J = 270.7 Hz), 118.0(q, J = 32.3 Hz), 114.2, 55.3, 52.3, 32.0, 31.7, 30.5. ^{19}F NMR (376 MHz, $CDCl_3$) δ -60.9. IR (neat, cm^{-1}) 2954, 1616, 1325, 1110, 1068, 824. HRMS (ESI) calculated for $C_{15}H_{23}F_3N$ $[M+H]^+$ m/z 274.1777, found 274.1787.

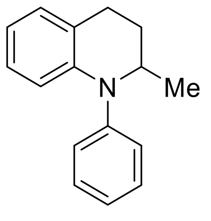

2-methyl-1-phenyl-1,2,3,4-tetrahydroquinoline (4a) [1]

Following the General Procedure using 1,2,3,4-tetrahydro-2-methylquinoline (29.4 mg, 0.2 mmol, 1.0 equiv) and chlorobenzene (26.9 mg, 0.24 mmol, 1.2 equiv), the reaction mixture was stirred at 80 °C for 24 h. The crude product was purified by column chromatography (0-2% EtOAc in hexanes) to provide the title compound as a yellow oil in 90% yield (40 mg).

^{1}H NMR (400 MHz, $CDCl_3$) δ 7.4 (m, 2H), 7.2 (m, 2H), 7.1 (m, 1H), 7.0 (m, 1H), 6.9 (m, 1H), 6.6 (m, 1H), 6.5 (m, 1H), 3.9 (m, 1H), 2.9 (m, 2H), 2.1 (m, 1H), 1.8 (m, 1H), 1.1 (d, J = 6.5 Hz, 3H). ^{13}C NMR (101 MHz, $cdcl_3$) δ 147.5, 144.3, 129.6, 129.3, 127.1, 126.5, 124.7, 123.2, 117.5,

115.9, 54.3, 28.5, 24.4, 19.8.

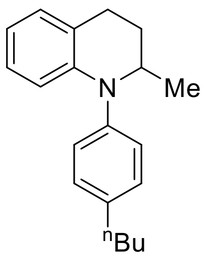

1-(4-butylphenyl)-2-methyl-1,2,3,4-tetrahydroquinoline (4b)

Following the General Procedure using 1,2,3,4-tetrahydro-2-methylquinoline (29.4 mg, 0.2 mmol, 1.0 equiv) and 1-butyl-4-chlorobenzene (40.3 mg, 0.24 mmol, 1.2 equiv), the reaction mixture was stirred at 80 ºC for 24 h. The crude product was purified by column chromatography (0-2% EtOAc in hexanes) to provide the title compound as a yellow oil in 94% yield (53 mg).

^{1}H NMR (400 MHz, $CDCl_3$) δ 7.2 (d, *J* = 8.2 Hz, 2H), 7.1 (d, *J* = 8.4 Hz, 2H), 7.0 (m, 1H), 6.9 (m, 1H), 6.6 (m, 1H), 6.4 (m, 1H), 3.9 (m, 1H), 2.9 (m, 2H), 2.6 (m, 2H), 2.1 (m, 1H), 1.9 (m, 1H), 1.6 (m, 2H), 1.4 (m, 2H), 1.2 (d, *J* = 6.5 Hz, 3H), 1.0 (t, *J* = 7.3 Hz, 3H). ^{13}C NMR (101 MHz, $CDCl_3$) δ 144.9, 144.8, 139.7, 129.6, 129.2, 127.5, 126.5, 122.6, 117.0, 115.3, 54.3, 35.3, 33.8, 28.6, 24.5, 22.6, 20.0, 14.1. IR (neat, cm^{-1}):2926, 1739, 1508, 1491, 1375, 1287, 1209. HRMS (ESI) calculated for $C_{20}H_{26}N$ $[M+H]^+$ *m/z* 280.2060, found 280.2068.

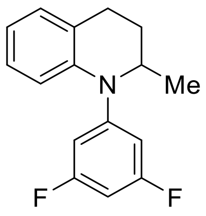

1-(3,5-difluorophenyl)-2-methyl-1,2,3,4-tetrahydroquinoline (4c) [1]

Following the General Procedure using 1,2,3,4-tetrahydro-2-methylquinoline (29.4 mg, 0.2 mmol, 1.0 equiv) and 1-chloro-3,5-difluorobenzene (35.5 mg, 0.24 mmol, 1.2 equiv), the reaction mixture was stirred at r.t. for 24 h. The crude product was purified by column chromatography (0-2% EtOAc in hexanes) to provide the title compound as a yellow oil in 81% yield (42 mg).

^{1}H NMR (400 MHz, $CDCl_3$) δ 7.1 (d, J = 7.4 Hz, 1H), 7.0 (t, J = 7.7 Hz, 1H), 6.9 (d, J = 8.2 Hz, 1H), 6.8 (t, J = 7.4 Hz, 1H), 6.7 (m, 2H), 6.4 (m, 1H), 4.0 (m, 1H), 2.8 (m, 2H), 2.1 (m, 1H), 1.8 (m, 1H), 1.2 (d, J = 6.6 Hz, 3H). ^{13}C NMR (101 MHz, $CDCl_3$) δ 163.9 (dd, J = 246.4 Hz, J = 15.2 Hz), 150.7 (t, J = 13.1 Hz), 141.5, 129.5, 126.6, 126.5, 120.4, 119.3, 105.6 (m), 97.7 (t, J = 6.1 Hz), 54.3, 28.7, 23.8, 19.1. ^{19}F NMR (376 MHz, $CDCl_3$) δ -110.0.

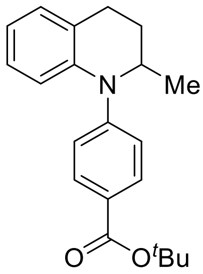

tert-butyl 4-(2-methyl-3,4-dihydroquinolin-1(2H)-yl)benzoate (4d) [1]

Following the General Procedure using 1,2,3,4-tetrahydro-2-methylquinoline (29.4 mg, 0.2 mmol, 1.0 equiv) and tert-butyl 4-chlorobenzoate (50.9 mg, 0.24 mmol, 1.2 equiv), the reaction mixture was stirred at 80 °C for 24 h. The crude product was purified by column chromatography (5-10% EtOAc in hexanes) to provide the title compound as a yellow oil in 75% yield (48 mg).

^{1}H NMR (400 MHz, $CDCl_3$) δ 7.9 (d, J = 8.8 Hz, 2H), 7.2 (d, J = 8.8 Hz, 2H), 7.1 (d, J = 7.3 Hz, 1H), 7.0 (m, 2H), 6.8 (m, 1H), 4.1 (m, 1H), 2.8 (m, 2H), 2.1 (m, 1H), 1.8 (m, 1H), 1.6 (s, 9H), 1.3 (d, J = 6.6 Hz, 3H). ^{13}C NMR (101 MHz, $CDCl_3$) δ 165.7, 151.9, 141.5, 130.9, 129.5, 126.4, 126.4, 125.2, 121.6, 120.2, 119.1, 80.5, 53.9, 28.6, 28.4, 23.7, 19.0.

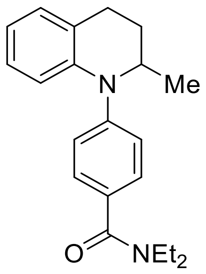

N,N-diethyl-4-(2-methyl-3,4-dihydroquinolin-1(2H)-yl)benzamide (4e) [1]

Following the General Procedure using 1,2,3,4-tetrahydro-2-methylquinoline (29.4 mg, 0.2 mmol, 1.0 equiv) and 4-chloro-N,N-diethylbenzamide (50.6 mg, 0.24 mmol, 1.2 equiv), the

reaction mixture was stirred at 80 °C for 24 h. The crude product was purified by column chromatography (10-20% EtOAc in hexanes) to provide the title compound as a yellow oil in 64% yield (42 mg).

^{1}H NMR (400 MHz, $CDCl_3$) δ 7.4 (d, J = 8.0 Hz, 2H), 7.2 (d, J = 8.5 Hz, 2H), 7.1 (d, J = 7.8 Hz, 1H), 6.9 (t, J = 7.7 Hz, 1H), 6.7 (m, 2H), 4.0 (m, 1H), 3.5 (m, 4H), 2.9 (m, 2H), 2.1 (m, 1H), 1.8 (m, 1H), 1.2 (m, 9H). ^{13}C NMR (101 MHz, $CDCl_3$) δ 171.2, 148.8, 142.9, 132.0, 129.4, 128.0, 126.4, 124.7, 124.5, 118.7, 117.4, 54.2, 28.2, 23.9, 19.4.

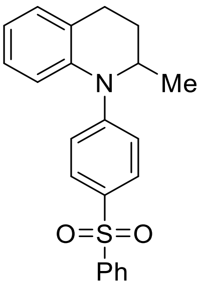

2-methyl-1-(4-(phenylsulfonyl)phenyl)-1,2,3,4-tetrahydroquinoline (4f) [1]

Following the General Procedure using 1,2,3,4-tetrahydro-2-methylquinoline (29.4 mg, 0.2 mmol, 1.0 equiv) and 1-chloro-4-(phenylsulfonyl)benzene (60.5 mg, 0.24 mmol, 1.2 equiv), the reaction mixture was stirred at r.t. for 24 h. The crude product was purified by column chromatography (5-10% EtOAc in hexanes) to provide the title compound as a solid in 84% yield (61 mg).

^{1}H NMR (400 MHz, $CDCl_3$) δ 7.9 (d, J = 6.7 Hz, 2H), 7.8 (d, J = 9.0 Hz, 2H), 7.5 (m, 3H), 7.2 (d, J = 8.9 Hz, 2H), 7.1 (m, 1H), 7.0 (m, 2H), 6.9 (m, 1H), 4.1 (m, 1H), 2.8 (m, 2H), 2.1 (m, 1H), 1.7 (m, 1H), 1.2 (d, J = 6.6 Hz, 3H). ^{13}C NMR (101 MHz, $CDCl_3$) δ 152.5, 142.7, 140.0, 132.8, 131.9, 129.5, 129.3, 129.3, 128.5, 127.4, 126.5, 121.9, 120.7, 119.8, 53.8, 29.3, 23.7, 18.7.

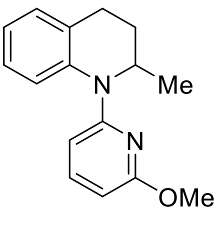

1-(6-methoxypyridin-2-yl)-2-methyl-1,2,3,4-tetrahydroquinoline (4g)

Following the General Procedure using 1,2,3,4-tetrahydro-2-methylquinoline (29.4 mg, 0.2 mmol, 1.0 equiv) and 2-chloro-6-methoxypyridine (34.3 mg, 0.24 mmol, 1.2 equiv), the reaction mixture was stirred at r.t. for 24 h. The crude product was purified by column chromatography (5-10% EtOAc in hexanes) to provide the title compound as a yellow oil in 90% yield (46 mg).

^{1}H NMR (400 MHz, $CDCl_3$) δ 7.3 (m, 2H), 7.2 (d, J = 8.7 Hz, 1H), 7.1 (t, J = 7.7 Hz, 1H), 6.9 (t, J = 7.4 Hz, 1H), 6.6 (d, J = 8.0 Hz, 1H), 6.2 (d, J = 7.9 Hz, 1H), 4.8 (m, 1H), 3.9 (s, 3H), 2.8 (m, 2H), 2.2 (m, 1H), 1.7 (m, 1H), 1.3 (d, J = 6.6 Hz, 3H). ^{13}C NMR (101 MHz, $cdcl_3$) δ 163.4, 156.7, 140.0, 139.4, 130.4, 128.9, 126.0, 122.3, 121.9, 103.1, 99.9, 53.2, 50.2, 30.4, 24.7, 19.2. IR (neat, cm^{-1}):2971, 1575, 1433, 1411, 1255, 1045,753. HRMS (ESI) calculated for $C_{16}H_{19}N_2O$ $[M+H]^+$ *m/z* 255.1492, found 255.1492.

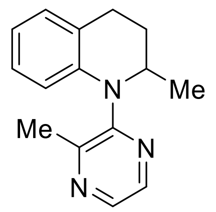

2-methyl-1-(3-methylpyrazin-2-yl)-1,2,3,4-tetrahydroquinoline (4h)

Following the General Procedure using 1,2,3,4-tetrahydro-2-methylquinoline (29.4 mg, 0.2 mmol, 1.0 equiv) and 2-chloro-3-methylpyrazine (30.7 mg, 0.24 mmol, 1.2 equiv), the reaction mixture was stirred at 80 ºC for 24 h. The crude product was purified by column chromatography (5-10% EtOAc in hexanes) to provide the title compound as a yellow oil in 75% yield (36 mg).

^{1}H NMR (400 MHz, $CDCl_3$) δ 8.3 (m, 2H), 7.1 (m, 1H), 6.9 (m, 1H), 6.8 (m, 1H), 6.1 (m, 1H), 4.3 (m, 1H), 2.9 (m, 2H), 2.3 (s, 3H), 2.1 (m, 1H), 1.9 (m, 1H), 1.2 (d, J = 6.5 Hz, 3H). ^{13}C NMR (101 MHz, $cdcl_3$) δ 153.7, 151.6, 141.9, 141.2, 139.4, 129.2, 126.6, 125.4, 119.7, 115.6, 52.0, 29.5, 24.4, 21.8, 19.5. IR (neat, cm^{-1}): 1737, 1493, 1406, 1373, 1237, 1044. HRMS (EI) calculated for $C_{15}H_{17}N_3$ $[M]^+$ *m/z* 239.1179, found 239.1174.

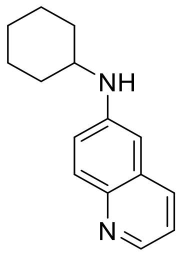

N-cyclohexylquinolin-6-amine (4i) [5]

Following the General Procedure using cyclohexanamine (19.8 mg, 0.2 mmol, 1.0 equiv) and 6-chloroquinoline (39.1 mg, 0.24 mmol, 1.2 equiv), the reaction mixture was stirred at r.t. for 24 h. The crude product was purified by column chromatography (0-5% EtOAc in hexanes) to provide the title compound as a yellow oil in 93% yield (42 mg).

^{1}H NMR (400 MHz, $CDCl_3$) δ 8.6 (d, *J* = 4.2 Hz, 1H), 7.9 (m, 2H), 7.2 (m, 1H), 7.0 (m, 1H), 6.7 (d, *J* = 2.6 Hz, 1H), 3.9 (s, 1H), 3.4 (t, *J* = 9.9 Hz, 1H), 2.1 (m, 2H), 1.8 (m, 2H), 1.7 (m, 1H), 1.4 (m, 2H), 1.2 (m, 3H). ^{13}C NMR (101 MHz, $CDCl_3$) δ 145.9, 145.3, 143.0, 133.7, 130.3, 130.3, 121.7, 121.4, 103.1, 51.8, 33.2, 25.9, 25.0.

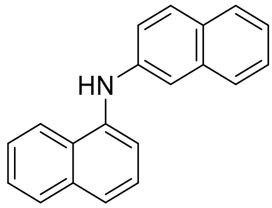

N-(naphthalen-2-yl)naphthalen-1-amine (4j) [6]

Following the General Procedure using naphthalen-1-amine (28.6 mg, 0.2 mmol, 1.0 equiv) and 2-chloronaphthalene (38.9 mg, 0.24 mmol, 1.2 equiv), the reaction mixture was stirred at 80 °C for 24 h. The crude product was purified by column chromatography (0-5% EtOAc in hexanes) to provide the title compound as a yellow oil in 84% yield (45 mg).

^{1}H NMR (400 MHz, $CDCl_3$) δ 8.0 (d, *J* = 8.4 Hz, 1H), 7.9 (d, *J* = 7.3 Hz, 1H), 7.7 (d, *J* = 8.4 Hz, 2H), 7.6 (m, 2H), 7.4 (m, 4H), 7.4 (m, 1H), 7.2 (m, 3H), 6.0 (s, 1H). ^{13}C NMR (101 MHz, $CDCl_3$) δ 142.7, 138.7, 134.9, 134.8, 129.3, 129.1, 128.7, 128.1, 127.8, 126.5, 126.5, 126.3, 126.2, 125.9, 123.6, 123.4, 122.1, 119.8, 116.7, 111.5.

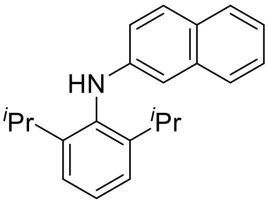

N-(2,6-diisopropylphenyl)naphthalen-2-amine (4k) [7]

Following the General Procedure using 2,6-diisopropylaniline (35.4 mg, 0.2 mmol, 1.0 equiv) and 2-chloronaphthalene (38.9 mg, 0.24 mmol, 1.2 equiv), the reaction mixture was stirred at 80 ºC for 24 h. The crude product was purified by column chromatography (0-5% EtOAc in hexanes) to provide the title compound as a yellow oil in 84% yield (51 mg).

^{1}H NMR (400 MHz, $CDCl_3$) δ 7.7 (m, 2H), 7.5 (m, 1H), 7.3 (m, 2H), 7.3 (m, 2H), 7.2 (m, 1H), 6.9 (m, 1H), 6.6 (m, 1H), 5.3 (s, 1H), 3.2 (m, 2H), 1.1 (d, J = 6.9 Hz, 12H). ^{13}C NMR (101 MHz, $CDCl_3$) δ 147.7, 145.9, 135.2, 135.1, 129.2, 127.8, 127.8, 127.5, 126.4, 126.1, 124.1, 122.2, 117.2, 105.9, 28.4, 24.0.

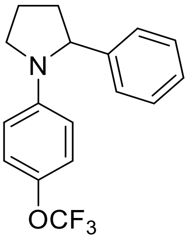

2-phenyl-1-(4-(trifluoromethoxy)phenyl)pyrrolidine (4l)

Following the General Procedure using 2-phenylpyrrolidine (29.4 mg, 0.2 mmol, 1.0 equiv) and 1-chloro-4-(trifluoromethoxy)benzene (47.0 mg, 0.24 mmol, 1.2 equiv), the reaction mixture was stirred at r.t. for 24 h. The crude product was purified by column chromatography (0-5% EtOAc in hexanes) to provide the title compound as a yellow oil in 80% yield (49 mg).

^{1}H NMR (400 MHz, $CDCl_3$) δ 7.3 (m, 2H), 7.2 (m, 3H), 7.0 (m, 2H), 6.4 (d, J = 9.1 Hz, 2H), 4.7 (m, 1H), 3.7 (m, 1H), 3.4 (m, 1H), 2.4 (m, 1H), 2.0 (m, 3H). ^{13}C NMR (101 MHz, $CDCl_3$) δ 146.1, 144.2, 139.6 (q, J = 2.0 Hz), 128.7, 127.0, 126.0, 122.3, 120.9 (q, J = 255.5 Hz), 112.6, 63.3, 49.5, 36.3, 23.3. ^{19}F NMR (376 MHz, $CDCl_3$) δ -58.5. IR (neat, cm^{-1}): 2928, 1515, 1369, 1257, 1225, 1258, 700. HRMS (ESI) calculated for $C_{17}H_{17}F_3NO$ [M+H]$^+$ *m/z* 308.1257, found 308.1265.

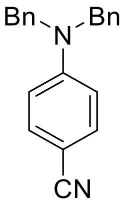

4-(dibenzylamino)benzonitrile (4m) [8]

Following the General Procedure using dibenzylamine (39.4 mg, 0.2 mmol, 1.0 equiv) and 4-chlorobenzonitrile (32.9 mg, 0.24 mmol, 1.2 equiv), the reaction mixture was stirred at r.t. for 24 h. The crude product was purified by column chromatography (0-5% EtOAc in hexanes) to provide the title compound as a yellow oil in 80% yield (48 mg).

^{1}H NMR (400 MHz, $CDCl_3$) δ 7.4 (d, J = 9.0 Hz, 2H), 7.3 (m, 4H), 7.3 (m, 2H), 7.2 (m, 4H), 6.7 (d, J = 9.0 Hz, 2H), 4.7 (s, 4H). ^{13}C NMR (101 MHz, $CDCl_3$) δ 151.9, 136.9, 133.8, 129.1, 127.6, 126.4, 120.5, 112.2, 98.6, 54.2.

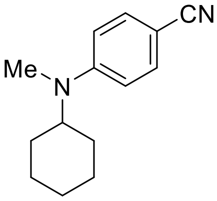

4-(cyclohexyl(methyl)amino)benzonitrile (4n)

Following the General Procedure using N-methylcyclohexanamine (22.6 mg, 0.2 mmol, 1.0 equiv) and 4-chlorobenzonitrile (32.9 mg, 0.24 mmol, 1.2 equiv), the reaction mixture was stirred at 80 °C for 24 h. The crude product was purified by column chromatography (0-5% EtOAc in hexanes) to provide the title compound as a yellow oil in 88% yield (38 mg).

^{1}H NMR (400 MHz, $CDCl_3$) δ 7.4 (d, J = 9.1 Hz, 2H), 6.7 (d, J = 9.1 Hz, 2H), 3.6 (m, 1H), 2.8 (s, 3H), 1.9 (m, 2H), 1.7 (m, 3H), 1.4 (m, 4H), 1.1 (m, 1H). ^{13}C NMR (101 MHz, $cdcl_3$) δ 152.3, 133.6, 121.0, 111.8, 96.9, 57.5, 31.1, 30.2, 26.1, 25.7. IR (neat, cm^{-1}): 2929, 2211, 1763, 1518, 1395, 1234, 816. HRMS (ESI) calculated for $C_{14}H_{19}N_2$ $[M+H]^+$ m/z 215.1543, found 215.1543.

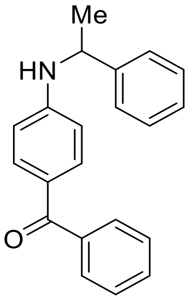

phenyl(4-((1-phenylethyl)amino)phenyl)methanone (4o)

Following the General Procedure using 1-phenylethan-1-amine (24.2 mg, 0.2 mmol, 1.0 equiv) and (4-chlorophenyl)(phenyl)methanone (51.8 mg, 0.24 mmol, 1.2 equiv), the reaction mixture was stirred at r.t. for 24 h. The crude product was purified by column chromatography (5-10% EtOAc in hexanes) to provide the title compound as a yellow oil in 91% yield (55 mg).

^{1}H NMR (400 MHz, $CDCl_3$) δ 7.7 (m, 4H), 7.5 (t, *J* = 7.4 Hz, 1H), 7.4 (t, *J* = 7.5 Hz, 2H), 7.3 (m, 4H), 7.2 (m, 1H), 6.5 (d, *J* = 8.4 Hz, 2H), 4.8 (m, 1H), 4.6 (m, 1H), 1.5 (d, *J* = 6.7 Hz, 3H). ^{13}C NMR (101 MHz, $CDCl_3$) δ 195.3, 151.2, 144.2, 139.2, 132.9, 131.3, 129.5, 128.9, 128.1, 127.3, 126.1, 125.8, 112.1, 53.2, 24.8. IR (neat, cm^{-1}): 3348, 1735, 1592, 1525, 1315, 1147, 699. HRMS (ESI) calculated for $C_{21}H_{20}NO$ $[M+H]^+$ *m/z* 302.1539, found 302.1547.

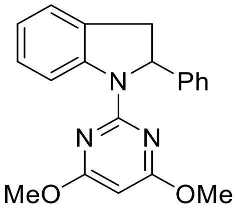

1-(4,6-dimethoxypyrimidin-2-yl)-2-phenylindoline (4p)

Following the General Procedure using 2-phenylindoline (39.0 mg, 0.2 mmol, 1.0 equiv) and 2-chloro-4,6-dimethoxypyrimidine (41.8 mg, 0.24 mmol, 1.2 equiv), the reaction mixture was stirred at r.t. for 24 h. The crude product was purified by column chromatography (5-10% EtOAc in hexanes) to provide the title compound as a yellow oil in 87% yield (58 mg).

^{1}H NMR (400 MHz, $CDCl_3$) δ 8.4 (d, *J* = 8.1 Hz, 1H), 7.3 (t, *J* = 7.5 Hz, 1H), 7.2 (d, *J* = 4.3 Hz, 4H), 7.2 (m, 2H), 7.0 (m, 1H), 5.8 (m, 1H), 5.5 (s, 1H), 3.7 (m, 7H), 3.0 (m, 1H). ^{13}C NMR (101 MHz, $CDCl_3$) δ 171.4, 157.8, 145.2, 144.2, 130.6, 128.5, 127.5, 126.9, 125.4, 125.0, 122.0, 115.5, 80.9, 63.4, 53.9, 37.8. IR (neat, cm^{-1}):2974, 1573, 1505, 1358, 1193, 1044, 800. HRMS

(ESI) calculated for $C_{20}H_{20}N_3O_2$ $[M+H]^+$ *m/z* 334.1550, found 334.1557.

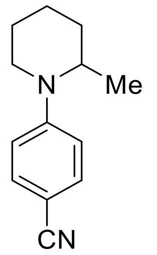

4-(2-methylpiperidin-1-yl)benzonitrile (4q) [9]

Following the General Procedure using 2-methylpiperidine (19.8 mg, 0.2 mmol, 1.0 equiv) and 4-chlorobenzonitrile (32.9 mg, 0.24 mmol, 1.2 equiv), the reaction mixture was stirred at 80 ºC for 24 h. The crude product was purified by column chromatography (0-5% EtOAc in hexanes) to provide the title compound as a yellow oil in 58% yield (23 mg).

^{1}H NMR (400 MHz, $CDCl_3$) δ 7.4 (d, *J* = 8.9 Hz, 2H), 6.8 (d, *J* = 8.9 Hz, 2H), 4.2 (m, 1H), 3.6 (m, 1H), 3.0 (m, 1H), 1.8 (m, 2H), 1.6 (m, 3H), 1.6 (m, 1H), 1.1 (d, *J* = 6.8 Hz, 3H). ^{13}C NMR (101 MHz, $CDCl_3$) δ 153.2, 133.6, 120.6, 113.8, 98.4, 49.3, 41.6, 30.7, 25.6, 18.5, 13.7.

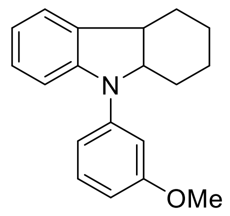

9-(3-methoxyphenyl)-2,3,4,4a,9,9a-hexahydro-1H-carbazole (4r)

Following the General Procedure using 2,3,4,4a,9,9a-hexahydro-1H-carbazole (34.6 mg, 0.2 mmol, 1.0 equiv) and 1-chloro-3-methoxybenzene (34 mg, 0.24 mmol, 1.2 equiv), the reaction mixture was stirred at 80 ºC for 24 h. The crude product was purified by column chromatography (0-5% EtOAc in hexanes) to provide the title compound as a yellow oil in 45% yield (25 mg).

^{1}H NMR (400 MHz, $CDCl_3$) δ 7.2 (m, 1H), 7.1 (d, *J* = 7.3 Hz, 1H), 7.0 (t, *J* = 7.7 Hz, 1H), 6.9 (d, *J* = 7.9 Hz, 1H), 6.8 (m, 3H), 6.6 (m, 1H), 4.1 (m, 1H), 3.8 (s, 3H), 3.3 (m, 1H), 1.8 (m, 3H), 1.7 (m, 1H), 1.5 (m, 3H), 1.3 (m, 1H). ^{13}C NMR (101 MHz, $CDCl_3$) δ 160.7, 148.0, 144.9, 135.0, 130.0, 127.1, 123.3, 119.2, 113.7, 109.8, 107.8, 107.1, 64.6, 55.4, 40.7, 27.4, 26.3, 22.5, 21.7. IR (neat, cm^{-1}): 2928, 1737, 1594, 1476, 1372, 1235, 1044. HRMS (ESI) calculated for

$C_{19}H_{22}NO$ $[M+H]^+$ *m/z* 280.1696, found 280.1703.

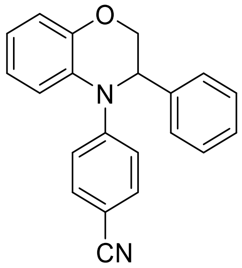

4-(3-methyl-2,3-dihydro-4H-benzo[b][1,4]oxazin-4-yl)benzonitrile (4s)

Following the General Procedure using 3-phenyl-3,4-dihydro-2H-benzo[b][1,4]oxazine (42.2 mg, 0.2 mmol, 1.0 equiv) and 4-chlorobenzonitrile (32.9 mg, 0.24 mmol, 1.2 equiv), the reaction mixture was stirred at 80 °C for 24 h. The crude product was purified by column chromatography (0-5% EtOAc in hexanes) to provide the title compound as a yellow oil in 62% yield (39 mg).

^{1}H NMR (400 MHz, $CDCl_3$) δ 7.5 (d, *J* = 8.8 Hz, 2H), 7.3 (m, 8H), 6.9 (m, 3H), 4.9 (t, *J* = 2.8 Hz, 1H), 4.6 (dd, *J* = 10.9, 2.6 Hz, 1H), 4.4 (dd, *J* = 10.9, 2.9 Hz, 1H). ^{13}C NMR (101 MHz, $CDCl_3$) δ 150.5, 145.8, 138.4, 133.7, 129.1, 128.8, 128.0, 126.6, 122.5, 121.6, 121.1, 119.3, 118.4, 117.9, 105.0, 68.4, 60.8. IR (neat, cm^{-1}): 2923, 2220, 1595, 1506, 1379, 1253, 750. HRMS (EI) calculated for $C_{21}H_{16}N_2O$ $[M]^+$ *m/z* 312.1263, found 312.1268.

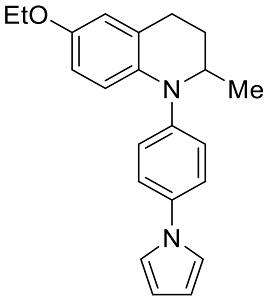

1-(4-(*1H*-pyrrol-1-yl)phenyl)-6-ethoxy-2-methyl-1,2,3,4-tetrahydroquinoline (4t)

Following the General Procedure using 6-ethoxy-2-methyl-1,2,3,4-tetrahydroquinoline (38.2 mg, 0.2 mmol, 1.0 equiv) and 1-(4-chlorophenyl)-1H-pyrrole (42.5 mg, 0.24 mmol, 1.2 equiv), the reaction mixture was stirred at 80 °C for 24 h. The crude product was purified by column chromatography (5-10% EtOAc in hexanes) to provide the title compound as a yellow oil in 43% yield (29 mg).

^{1}H NMR (400 MHz, $CDCl_3$) δ 7.3 (d, J = 8.8 Hz, 2H), 7.2 (d, J = 8.8 Hz, 2H), 7.0 (t, J = 2.2 Hz, 2H), 6.7 (m, 2H), 6.6 (m, 1H), 6.3 (t, J = 2.2 Hz, 2H), 4.0 (m, 2H), 3.9 (m, 1H), 2.8 (m, 2H), 2.1 (m, 1H), 1.8 (m, 1H), 1.4 (t, J = 7.0 Hz, 3H), 1.2 (d, J = 6.6 Hz, 3H). ^{13}C NMR (101 MHz, $CDCl_3$) δ 152.1, 146.8, 137.2, 136.1, 126.2, 125.6, 121.8, 119.6, 119.3, 115.1, 113.4, 110.1, 64.0, 54.6, 28.4, 24.5, 19.5, 15.2. IR (neat, cm^{-1}): 2927, 1737, 1516, 1497, 1235, 1043, 726. HRMS (ESI) calculated for $C_{22}H_{25}N_2O$ $[M+H]^+$ *m/z* 333.1961, found 333.1963.

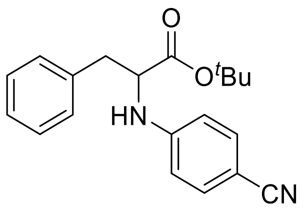

tert-butyl (4-cyanophenyl)phenylalaninate (5a)

Following the General Procedure using *tert*-butyl phenylalaninate (44.2 mg, 0.2 mmol, 1.0 equiv) and 4-chlorobenzonitrile (32.9 mg, 0.24 mmol, 1.2 equiv), the reaction mixture was stirred at 80 °C for 24 h. The crude product was purified by column chromatography (0-5% EtOAc in hexanes) to provide the title compound as a yellow oil in 62% yield (40 mg).

^{1}H NMR (400 MHz, $CDCl_3$) δ 7.4 (d, J = 8.8 Hz, 2H), 7.3 (m, 3H), 7.2 (m, 2H), 6.5 (d, J = 8.8 Hz, 2H), 4.7 (m, 1H), 4.3 (m, 1H), 3.1 (m, 2H), 1.4 (s, 9H). ^{13}C NMR (101 MHz, $CDCl_3$) δ 171.2, 149.9, 135.9, 133.9, 129.6, 128.6, 127.3, 120.3, 113.0, 99.9, 82.8, 57.2, 38.3, 28.0. IR (neat, cm^{-1}): 3365, 2927, 2215, 1730, 1607, 1524, 1152. HRMS (ESI) calculated for $C_{20}H_{22}N_2NaO_2$ $[M+Na]^+$ *m/z* 345.1573, found 345.1576.

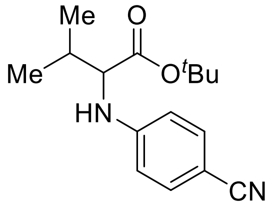

tert-butyl (4-cyanophenyl)valinate (5b)

Following the General Procedure using *tert*-butyl valinate (34.6 mg, 0.2 mmol, 1.0 equiv) and 4-chlorobenzonitrile (32.9 mg, 0.24 mmol, 1.2 equiv), the reaction mixture was stirred at 80 °C for 24 h. The crude product was purified by column chromatography (0-5% EtOAc in hexanes)

to provide the title compound as a yellow oil in 80% yield (44 mg).

^{1}H NMR (400 MHz, $CDCl_3$) δ 7.4 (d, J = 8.7 Hz, 2H), 6.6 (d, J = 8.6 Hz, 2H), 4.7 (d, J = 8.6 Hz, 1H), 3.8 (m, 1H), 2.1 (m, 1H), 1.5 (s, 9H), 1.0 (m, 6H). ^{13}C NMR (101 MHz, $CDCl_3$) δ 171.6, 150.8, 133.8, 120.3, 113.0, 99.5, 82.4, 61.8, 31.4, 28.1, 18.9, 18.6. IR (neat, cm^{-1}): 3368, 2969, 2214, 1724, 1606, 1524, 1147. HRMS (ESI) calculated for $C_{16}H_{22}N_2NaO_2$ $[M+Na]^+$ *m/z* 297.1573, found 297.1576.

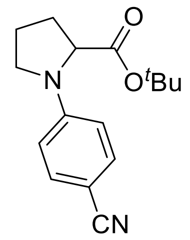

***tert*-butyl (4-cyanophenyl)prolinate (5c)**

Following the General Procedure using *tert*-butyl prolinate (34.2 mg, 0.2 mmol, 1.0 equiv) and 4-chlorobenzonitrile (32.9 mg, 0.24 mmol, 1.2 equiv), the reaction mixture was stirred at 80 ºC for 24 h. The crude product was purified by column chromatography (0-5% EtOAc in hexanes) to provide the title compound as a yellow oil in 78% yield (42 mg).

^{1}H NMR (400 MHz, $CDCl_3$) δ 7.4 (m, 2H), 6.5 (m, 2H), 4.2 (m, 1H), 3.5 (m, 1H), 3.4 (m, 1H), 2.3 (m, 1H), 2.2 (m, 2H), 2.1 (m, 1H), 1.4 (s, 9H). ^{13}C NMR (101 MHz, $CDCl_3$) δ 172.4, 149.5, 133.6, 120.7, 112.1, 98.2, 82.0, 61.5, 48.3, 30.8, 28.1, 23.7. IR (neat, cm^{-1}): 2977, 2213, 1735, 1606, 1521, 1385, 1150. HRMS (ESI) calculated for $C_{16}H_{20}N_2NaO_2$ $[M+Na]^+$ *m/z* 295.1417, found 295.1425.

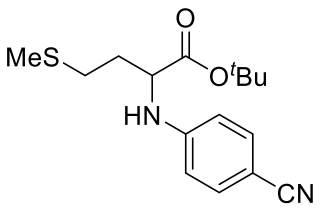

***tert*-butyl (4-cyanophenyl)methioninate (5d)**

Following the General Procedure using *tert*-butyl methioninate (41.0 mg, 0.2 mmol, 1.0 equiv) and 4-chlorobenzonitrile (32.9 mg, 0.24 mmol, 1.2 equiv), the reaction mixture was stirred at 80 ºC for 24 h. The crude product was purified by column chromatography (0-5% EtOAc in

hexanes) to provide the title compound as a yellow oil in 87% yield (53 mg).

^{1}H NMR (400 MHz, CDCl$_3$) δ 7.4 (d, J = 8.8 Hz, 2H), 6.6 (d, J = 8.8 Hz, 2H), 4.8 (d, J = 8.3 Hz, 1H), 4.2 (m, 1H), 2.6 (t, J = 7.3 Hz, 2H), 2.1 (m, 1H), 2.1 (s, 3H), 2.0 (m, 1H), 1.4 (s, 9H). ^{13}C NMR (101 MHz, CDCl$_3$) δ 171.7, 150.2, 133.8, 120.2, 113.0, 99.9, 82.8, 55.2, 31.9, 30.1, 28.1, 15.6. IR (neat, cm^{-1}): 3359, 2978, 2214, 1730, 1606, 1524, 1369, 1150. HRMS (ESI) calculated for $C_{16}H_{23}N_2O_2S$ [M+H]$^+$ *m/z* 307.1475, found 307.1479.

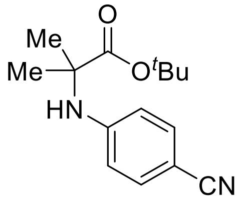

tert-butyl 2-((4-cyanophenyl)amino)-2-methylpropanoate (5e)

Following the General Procedure using *tert*-butyl 2-amino-2-methylpropanoate (31.8 mg, 0.2 mmol, 1.0 equiv) and 4-chlorobenzonitrile (32.9 mg, 0.24 mmol, 1.2 equiv), the reaction mixture was stirred at 80 ºC for 24 h. The crude product was purified by column chromatography (0-5% EtOAc in hexanes) to provide the title compound as a yellow oil in 70% yield (37 mg).

^{1}H NMR (400 MHz, CDCl$_3$) δ 7.4 (d, J = 8.8 Hz, 2H), 6.5 (m, 2H), 4.6 (s, 1H), 1.5 (s, 6H), 1.4 (s, 9H). ^{13}C NMR (101 MHz, CDCl$_3$) δ 174.2, 149.4, 133.5, 120.4, 114.0, 99.4, 82.0, 57.8, 27.9, 26.0.

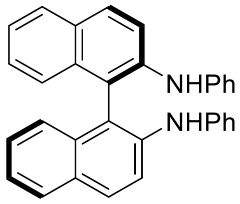

N^2,N$^{2'}$-diphenyl-[1,1'-binaphthalene]-2,2'-diamine (6a)

Following the General Procedure using [1,1'-binaphthalene]-2,2'-diamine (56.8 mg, 0.2 mmol, 1.0 equiv) and chlorobenzene (53.8 mg, 0.48 mmol, 2.4 equiv), the reaction mixture was stirred at r.t.. for 24 h. The crude product was purified by column chromatography (0-5% EtOAc in hexanes) to provide the title compound as a yellow oil in 95% yield (83 mg).

^{1}H NMR (400 MHz, CDCl$_3$) δ 7.8 (m, 4H), 7.7 (d, J = 9.0 Hz, 2H), 7.3 (m, 2H), 7.2 (m, 8H),

6.9 (m, 6H), 5.6 (s, 2H). ^{13}C NMR (101 MHz, $CDCl_3$) δ 142.6, 140.5, 134.1, 129.5, 129.5, 129.3, 128.3, 127.2, 124.6, 123.6, 122.3, 120.0, 118.0, 116.5.

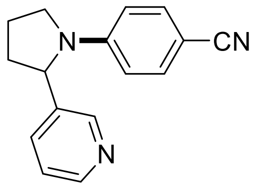

4-(2-(pyridin-3-yl)pyrrolidin-1-yl)benzonitrile (6b)

Following the General Procedure using 3-(pyrrolidin-2-yl)pyridine (29.6 mg, 0.2 mmol, 1.0 equiv) and 4-chlorobenzonitrile (32.9 mg, 0.24 mmol, 1.2 equiv), the reaction mixture was stirred at r.t. for 24 h. The crude product was purified by column chromatography (5-10% EtOAc in hexanes) to provide the title compound as a yellow oil in 78% yield (39 mg).

^{1}H NMR (400 MHz, $CDCl_3$) δ 8.5 (m 2H), 7.4 (d, J = 7.9 Hz, 1H), 7.4 (d, J = 8.9 Hz, 2H), 7.3 (m, 1H), 6.4 (d, J = 9.0 Hz, 2H), 4.9 (d, J = 8.6 Hz, 1H), 3.7 (m, 1H), 3.5 (m 1H), 2.5 (m, 1H), 2.0 (m, 3H). ^{13}C NMR (101 MHz, $CDCl_3$) δ 149.3, 148.8, 147.9, 138.2, 133.5(2C), 123.7, 120.6, 112.6, 98.1, 60.7, 49.1, 35.8, 22.8.

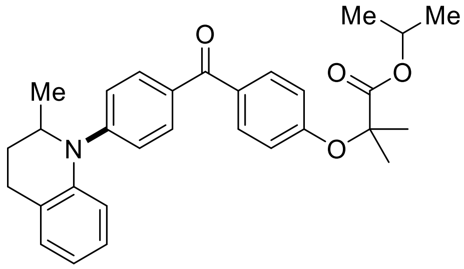

Isopropyl2-methyl-2-(4-(4-(2-methyl-3,4-dihydroquinolin-1(2H)

yl)benzoyl)phenoxy)propanoate (6c)

Following the General Procedure using 2-methyl-1,2,3,4-tetrahydroquinoline (29.4 mg, 0.2 mmol, 1.0 equiv) and isopropyl 2-(4-(4-chlorobenzoyl)phenoxy)-2-methylpropanoate (86.4 mg, 0.24 mmol, 1.2 equiv), the reaction mixture was stirred at 80 °C for 24 h. The crude product was purified by column chromatography (5-10% EtOAc in hexanes) to provide the title compound as a yellow oil in 66% yield (62 mg).

^{1}H NMR (400 MHz, $CDCl_3$) δ 7.7 (m, 4H), 7.2 (d, J = 8.7 Hz, 2H), 7.1 (d, J = 7.5 Hz, 1H), 7.0 (m, 2H), 6.9 (m, 3H), 5.1 (p, J = 6.1 Hz, 1H), 4.2 (m, 1H), 2.8 (m, 2H), 2.1 (m, 1H), 1.8 (m,

1H), 1.7 (s, 6H), 1.3 (d, J = 6.6 Hz, 3H), 1.2 (d, J = 6.2 Hz, 6H). ^{13}C NMR (101 MHz, $CDCl_3$) δ 194.3, 173.4, 159.2, 151.9, 141.1, 131.9, 131.8, 131.6, 130.9, 129.5, 127.2, 126.5, 120.8, 120.7, 119.9, 117.3, 79.4, 69.4, 53.9, 28.9, 25.5, 25.5, 23.8, 21.7, 19.0.

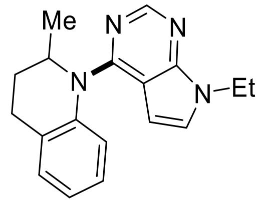

1-(7-ethyl-7H-pyrrolo[2,3-d]pyrimidin-4-yl)-2-methyl-1,2,3,4-tetrahydroquinoline (6d)

Following the General Procedure using 2-methyl-1,2,3,4-tetrahydroquinoline (29.4 mg, 0.2 mmol, 1.0 equiv) and 4-chloro-7-ethyl-7H-pyrrolo[2,3-d]pyrimidine (43.4 mg, 0.24 mmol, 1.2 equiv), the reaction mixture was stirred at r.t. for 24 h. The crude product was purified by column chromatography (5-10% EtOAc in hexanes) to provide the title compound as a yellow oil in 67% yield (39 mg).

^{1}H NMR (400 MHz, $CDCl_3$) δ 8.5 (s, 1H), 7.1 (m, 4H), 6.8 (d, J = 3.6 Hz, 1H), 5.5 (d, J = 3.6 Hz, 1H), 5.0 (m, 1H), 4.2 (q, J = 7.3 Hz, 2H), 2.7 (m, 2H), 2.3 (m, 1H), 1.6 (m, 1H), 1.4 (t, J = 7.3 Hz, 3H), 1.3 (d, J = 6.6 Hz, 3H). ^{13}C NMR (101 MHz, $CDCl_3$) δ 156.2, 151.5, 151.0, 138.7, 132.8, 128.2, 125.8, 124.7, 124.1, 122.9, 105.5, 101.5, 50.9, 39.5, 31.4, 24.9, 19.4, 15.8.

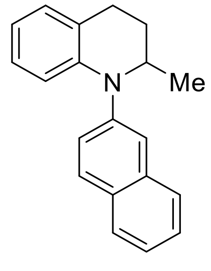

2-methyl-1-(naphthalen-2-yl)-1,2,3,4-tetrahydroquinoline (6e)

Following the General Procedure using 2-methyl-1,2,3,4-tetrahydroquinoline (29.4 mg, 0.2 mmol, 1.0 equiv) and phenol derivative (0.24 mmol, 1.2 equiv), the reaction mixture was stirred at indicated temperature for 24 h. The crude product was purified by column chromatography (0-5% EtOAc in hexanes) to provide the title compound as a yellow oil.

^{1}H NMR (400 MHz, $CDCl_3$) δ 7.80 (d, J = 9.0 Hz, 2H), 7.73 (d, J = 7.8 Hz, 1H), 7.62 (d, J =

1.9 Hz, 1H), 7.41 (m, 3H), 7.07 (d, *J* = 7.3 Hz, 1H), 6.90 (m, 1H), 6.69 (td, *J* = 7.3, 0.9 Hz, 1H), 6.61 (d, *J* = 8.2 Hz, 1H), 4.05 (m, 1H), 2.94 (m, 1H), 2.84 (m, 1H), 2.11 (m, 1H), 1.88 (m, 1H), 1.21 (d, *J* = 6.5 Hz, 3H). ^{13}C NMR (101 MHz, $CDCl_3$) δ 145.1, 144.0, 134.7, 131.0, 129.4, 129.2, 127.7, 127.4, 126.6, 126.4, 126.3, 125.1, 123.7, 123.3, 118.0, 116.4, 54.4, 28.5, 24.3, 19.8.

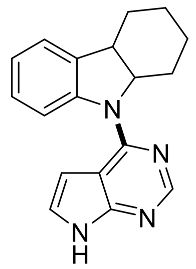

9-(7H-pyrrolo[2,3-d]pyrimidin-4-yl)-2,3,4,4a,9,9a-hexahydro-1H-carbazole (7)

Following the General Procedure using 2,3,4,4a,9,9a-hexahydro-1H-carbazole (34.6 mg, 0.2 mmol, 1.0 equiv) and 4-chloro-7H-pyrrolo[2,3-d]pyrimidine (36.7 mg, 0.24 mmol, 1.2 equiv), the reaction mixture was stirred at r.t. for 20 h. The crude product was purified by column chromatography (5-10% EtOAc in hexanes) to provide the title compound in 72% yield (42 mg).

^{1}H NMR (400 MHz, $CDCl_3$) δ 11.9 (s, 1H), 8.5 (s, 1H), 8.3 (d, *J* = 7.9 Hz, 1H), 7.2 (m, 3H), 7.1 (t, *J* = 7.4 Hz, 1H), 6.6 (d, *J* = 3.6 Hz, 1H), 4.9 (m, 1H), 3.6 (m, 1H), 2.3 (m, 2H), 1.9 (m, 1H), 1.6 (m, 2H), 1.3 (m, 3H). ^{13}C NMR (101 MHz, $CDCl_3$) δ 153.5, 152.3, 150.7, 143.6, 134.6, 127.0, 122.7, 122.5, 121.4, 118.0, 103.1, 101.4, 62.6, 40.4, 27.5, 24.2, 22.6, 21.0.

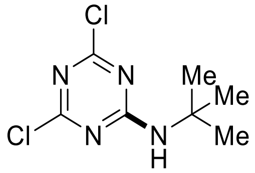

N-(*tert*-butyl)-4,6-dichloro-1,3,5-triazin-2-amine (8)

Following the General Procedure using 2-methylpropan-2-amine (14.6 mg, 0.2 mmol, 1.0 equiv) and 2,4,6-trichloro-1,3,5-triazine (44.2 mg, 0.24 mmol, 1.2 equiv), the reaction mixture was stirred at 80 °C for 24 h. The crude product was purified by column chromatography (0-5% EtOAc in hexanes) to provide the title compound in 90% yield (40 mg).

^{1}H NMR (400 MHz, $CDCl_3$) δ 5.8 (s, 1H), 1.5 (s, 9H). ^{13}C NMR (101 MHz, $cdcl_3$) δ 53.1, 28.5.

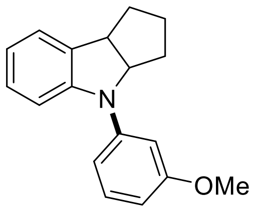

4-(3-methoxyphenyl)-1,2,3,3a,4,8b-hexahydrocyclopenta[*b*]indole (9)

Following the General Procedure using 1,2,3,3a,4,8b-hexahydrocyclopenta[b]indole (31.8 mg, 0.2 mmol, 1.0 equiv) and 1-chloro-3-methoxybenzene (34.1 mg, 0.24 mmol, 1.2 equiv), the reaction mixture was stirred at 80 °C for 24 h. The crude product was purified by column chromatography (0-5% EtOAc in hexanes) to provide the title compound in 77% yield (41 mg). ^{1}H NMR (400 MHz, $CDCl_3$) δ 7.2 (t, J = 8.1 Hz, 1H), 7.1 (m, 1H), 7.0 (m, 2H), 6.9 (m, 2H), 6.7 (m, 1H), 6.5 (m, 1H), 4.7 (m, 1H), 3.8 (m, 1H), 3.8 (s, 3H), 1.9 (m, 4H), 1.6 (m, 1H), 1.5 (m, 1H). ^{13}C NMR (101 MHz, $CDCl_3$) δ 160.5, 147.2, 144.8, 135.2, 129.9, 127.2, 124.8, 119.0, 111.6, 108.7, 106.3, 105.1, 55.3, 45.6, 34.9, 34.2, 24.6.

5-mmol-scale C–N cross-coupling

Gram-scale reaction

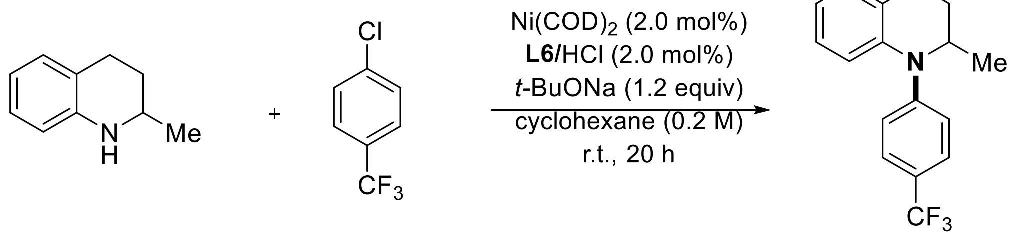

In a nitrogen-filled glove box, an oven-dried screw-cap reaction tube equipped with a magnetic stir bar was charged with $Ni(COD)_2$ (27.5 mg, 100 μmol, 2.0 mol%), **L6**/HCl (81.0 mg, 100 μmol, 2.0 mol%), ^{t}BuOK (672 mg, 6 mmol, 1.2 equiv) and cyclopentane (25.0 mL, 0.2 M). The reaction tube was sealed with a screw-cap septum and stirred at room temperature (r.t.) for 20 min until the color of the reaction mixture changed from yellow to brown. 4-chlorobenzotrifluoride (1083 mg, 6 mmol, 1.2 equiv) and 1,2,3,4-tetrahydro-2-methylquinoline (735 mg, 5 mmol, 1.0 equiv) were added. The reaction tube was wrapped with electrical tape and removed from the glove box. The reaction mixture was stirred at r.t. for 24 h, followed by adding a saturated aqueous solution of NaCl (20 mL) and EtOAc (20 mL). The phases were

separated, and the aqueous layer was extracted with EtOAc (2 x 20 mL). The combined organic phases were washed with a saturated aqueous solution of NaCl (2 x 20 mL) and then concentrated in vacuo. The crude residue was purified via column chromatography to afford the aminated product **3a** in 93% yield.

DFT Calculations of the Ni-Catalyzed *N*-Arylation

Computational Details

All density functional theory (DFT) calculations were carried out using Gaussian16 software package.[10] Geometry optimizations of all intermediates and transition states were carried out at the M06[11] level of theory using a mixed basis set of def2-SVP[3]. Frequency analysis was also performed at the same level of theory as geometry optimization to confirm whether optimized stationary points were either local minimum or transition state, as well as to evaluate zero-point vibrational energies (ZPVE) and thermal corrections at 298 K. The single-point energies were computed based on the gas-phase optimized structures at the M06[11] level of theory with a mixed basis set of def2-TZVP[12], with the inclusion of solvation energy corrections using a self-consistent reaction field (SCRF) based on SMD implicit solvation model based on density[13] with cyclohexane as solvent.

Table of energies

Zero-point correction (*ZPE*), thermal correction to enthalpy (*TCH*), thermal correction to Gibbs free energy (*TCG*), energies (*E*), enthalpies (*H*), and Gibbs free energies (*G*) (in Hartree) of the

structures calculated at the M06/def2-TZVP-SMD(cyclohexane)//M06/def2-SVP level of theory.

Energies for All Calculated Species*

Structures	*ZPE*	*TCH*	*TCG*	*E*	*H*	*G*	Imaginary Frequency
**INT1-L6**	1.106011	1.169543	1.006254	-4300.127914	-4298.958371	-4299.121660	
Chlorobenzene	0.090526	0.096972	0.061410	-691.743683	-691.646711	-691.682273	
Tetrahydroquinoline anion	0.192915	0.202987	0.159023	-442.920218	-442.717231	-442.761195	
**INT2-L6**	1.002882	1.062948	0.907343	-4548.914210	-4547.851262	-4548.006867	
**TS3-L6**	1.001649	1.061672	0.904443	-4548.903588	-4547.841916	-4547.999145	153.9*i*
**INT4-L6**	1.002516	1.063244	0.904356	-4548.932398	-4547.869154	-4548.028042	
**INT5-L6**	1.200934	1.270500	1.097835	-4991.930467	-4990.659967	-4990.832632	
Chloride anion	0.000000	0.002360	-0.015023	-460.306569	-460.304209	-460.321592	
**INT6-L6**	1.201147	1.269136	1.099352	-4531.589401	-4530.320265	-4530.490049	
**TS7-L6**	1.200263	1.267849	1.098989	-4531.564707	-4530.296858	-4530.465718	328.7*i*
**INT8-L6**	1.201003	1.269052	1.098248	-4531.600096	-4530.331044	-4530.501848	
**3a**	0.288646	0.303474	0.247769	-674.423724	-674.120250	-674.175955	
**INT1-L2**	0.867074	0.916049	0.786591	-3494.189468	-3493.273419	-3493.402877	
**INT2-L2**	0.763488	0.808882	0.688561	-3742.986870	-3742.177988	-3742.298309	
**TS3-L2**	0.763200	0.808235	0.687877	-3742.971478	-3742.163243	-3742.283601	206.5*i*
**INT4-L2**	0.764161	0.810141	0.686417	-3743.000646	-3742.190506	-3742.314230	
**INT5-L2**	0.961057	1.016670	0.875286	-4186.001763	-4184.985093	-4185.126477	
**INT6-L2**	0.961220	1.015177	0.875798	-3725.664913	-3724.649736	-3724.789115	
**TS7-L2**	0.961091	1.014354	0.877069	-3725.634700	-3724.620346	-3724.757631	543.3*i*
**INT8-L2**	0.962557	1.016120	0.878859	-3725.665868	-3724.649748	-3724.787009	
**INT1-L4**	1.346170	1.423685	1.232635	-5106.065290	-5104.641605	-5104.832655	
**INT2-L4**	1.242231	1.316782	1.129590	-5354.842218	-5353.525436	-5353.712628	
**TS3-L4**	1.241475	1.315672	1.129221	-5354.829389	-5353.513717	-5353.700168	145.0*i*
**INT4-L4**	1.242994	1.317575	1.130127	-5354.858935	-5353.54136	-5353.728808	
**INT5-L4**	1.440047	1.523845	1.320779	-5797.846987	-5796.323142	-5796.526208	
**INT6-L4**	1.439750	1.522440	1.319246	-5337.516653	-5335.994213	-5336.197407	
**TS7-L4**	1.439431	1.521404	1.321363	-5337.490765	-5335.969361	-5336.169402	335.5*i*
**INT8-L4**	1.440793	1.522962	1.322456	-5337.516558	-5335.993596	-5336.194102	

*-**L6**, -**L2**, -**L4** stands for species coordinated by ligand L6, L2 and L4 separately.

Cartesian Coordinates of the Structures

INT1-L6

C	0.18934600	-0.00221800	-0.93432400
N	1.34122400	0.37767000	-1.65439100

N	0.73114100	-0.45802600	0.29061400
C	1.28890600	1.09206000	-2.88263100
C	1.22650600	2.50164300	-5.27399700
C	1.19440100	2.49558900	-2.84799700
C	1.36272500	0.38492200	-4.09663400
C	1.32621000	1.11409500	-5.28763900
C	1.16040200	3.18311400	-4.06460500
H	1.36806800	0.58444300	-6.24504400
H	1.06885500	4.27471300	-4.06174200
C	-0.11111100	-0.79377900	1.38365100
C	-1.74873300	-1.46880500	3.55035300
C	-0.37515800	0.16593000	2.37151100
C	-0.71599600	-2.06272700	1.42420900
C	-1.51677200	-2.38399900	2.51808000
C	-1.19283400	-0.19393200	3.44513500
H	-2.00903300	-3.36319900	2.55127700
H	-1.45484000	0.56649100	4.19210700
C	-0.54811100	-2.96502100	0.21683000
H	-0.78488400	-2.29030800	-0.63819800
C	0.11716400	1.59193300	2.19615500
C	-2.61153800	-1.82701800	4.72206000
H	-3.30062000	-1.00453200	4.97489200
C	1.38321800	-1.12638500	-4.10495800
H	1.81808500	-1.45027900	-3.14111100
C	1.05521500	3.24910100	-1.54316300
H	1.31295400	2.54653000	-0.73065500
C	2.23604100	-1.72246500	-5.21480600
H	1.79615200	-1.55246600	-6.21349400
H	2.31495100	-2.81543700	-5.08597500
H	3.25784800	-1.30595200	-5.21922900

C	-0.39719100	3.66432100	-1.32392400
H	-0.74721800	4.33804100	-2.12758600
H	-0.51651200	4.18747600	-0.35799200
H	-1.04455800	2.76596400	-1.30770700
Ni	-1.51849500	0.23389700	-1.28633000
H	-2.00848100	-2.03117100	5.62457700
H	-3.21537500	-2.72563500	4.52077800
C	-1.02321900	2.57163900	2.42243200
C	-1.03527000	3.54598600	3.42024900
C	-2.12663000	2.46093900	1.56353700
C	-2.13989100	4.39084300	3.56386400
H	-0.17577000	3.65229200	4.09146400
C	-3.22772300	3.29416000	1.71160700
H	-2.10300500	1.71580800	0.75283300
C	-3.23698800	4.26448400	2.71666400
H	-2.13874500	5.15291500	4.35037000
H	-4.08380900	3.16701400	1.03823500
H	-4.10363800	4.92219500	2.83827600
C	0.87846200	-3.42682400	0.02771000
C	1.70209900	-3.76832200	1.10437000
C	1.40927800	-3.49988900	-1.26405100
C	3.02600000	-4.15378800	0.89790700
H	1.30063500	-3.70722000	2.12336400
C	2.73312300	-3.87480900	-1.47445100
H	0.76835000	-3.22707900	-2.11017400
C	3.54984400	-4.20067900	-0.39178400
H	3.66084400	-4.39869900	1.75578300
H	3.13461800	-3.89770500	-2.49350700
H	4.59514500	-4.48093700	-0.55290500
C	4.90769200	-0.98777800	2.86220200

C	5.95253600	-0.57468700	2.06173500
C	5.69315500	-0.09547800	0.74334900
C	4.36238400	-0.08295200	0.34013700
C	3.27255400	-0.51443600	1.15692500
C	3.55229200	-0.96394400	2.42750600
H	7.71343400	0.38609400	0.05868800
H	5.12064600	-1.34292800	3.87572800
H	6.98258000	-0.60715200	2.43337200
C	6.65011800	0.37596200	-0.20480100
C	3.91891600	0.37293200	-0.94161400
H	2.75661600	-1.29469800	3.10313100
C	4.86490600	0.82595600	-1.83622300
C	6.23420100	0.81705200	-1.44415600
H	4.58095000	1.18804500	-2.83003500
H	6.98097300	1.17689600	-2.15937800
C	2.10027100	-0.31076000	0.31798100
C	2.47877800	0.20504900	-0.89418800
C	1.39592700	1.89968000	2.94516000
C	2.40676300	2.62738800	2.30694700
C	1.61737200	1.47599900	4.25967800
C	3.60677500	2.91291800	2.95321100
H	2.25436200	2.95459500	1.27105700
C	2.81213000	1.76869200	4.91478100
H	0.84569300	0.89318000	4.77358900
C	3.81433800	2.48384600	4.26228300
H	4.39017800	3.46263900	2.42242200
H	2.96478900	1.42424900	5.94275600
H	4.75969300	2.69875300	4.76998200
C	-1.57436100	-4.07412300	0.16409500
C	-1.30403400	-5.38409200	0.56764000

C	-2.86978300	-3.75654100	-0.26714800
C	-2.30734600	-6.35380300	0.55422500
H	-0.29303000	-5.65022800	0.89503200
C	-3.87183900	-4.72106800	-0.27525700
H	-3.08899800	-2.72971400	-0.58734400
C	-3.59455400	-6.02503700	0.13695000
H	-2.07795700	-7.37574100	0.87359400
H	-4.87830200	-4.44841600	-0.60681300
H	-4.38226900	-6.78520000	0.12909500
C	2.01102700	4.42974500	-1.43746400
H	3.05911300	4.12005000	-1.59212300
H	1.77903600	5.21889300	-2.17410800
H	1.93585700	4.89343300	-0.43884500
C	-0.05237200	-1.64578300	-4.16248400
H	-0.54390400	-1.31552200	-5.09579800
H	-0.08069400	-2.75035500	-4.13996600
H	-0.64279300	-1.25499200	-3.30989900
H	1.19456400	3.05644400	-6.21711500
C	-4.88546500	-0.52534700	1.58050800
C	-3.97147800	-0.27619100	0.57238200
C	-4.26266500	0.57802400	-0.52768000
C	-5.56539400	1.16415000	-0.54492800
C	-6.46908800	0.89204200	0.48130900
C	-6.15730100	0.05987600	1.55616700
H	-4.59545500	-1.19225900	2.40277700
H	-2.96494700	-0.71283700	0.60289200
C	-5.94437800	2.05830400	-1.69409400
H	-7.46412300	1.35582900	0.42463000
H	-6.88725500	-0.13430500	2.34812600
C	-5.10054900	1.73232500	-2.91133000

C	-3.62457500	1.74231000	-2.53480500
H	-7.02499000	1.96361800	-1.91094900
H	-5.35423700	0.72197200	-3.28997200
N	-3.31267300	0.79363800	-1.48087400
C	-2.74462600	1.48210700	-3.74364400
H	-3.00753600	0.50858400	-4.19684200
H	-2.85267000	2.27166000	-4.50948700
H	-1.68640900	1.41519200	-3.43090300
H	-3.39786100	2.78167800	-2.17754400
H	-5.28694500	2.44565300	-3.73556100
H	-5.78484100	3.12471100	-1.42783000

Chlorobenzene

C	0.00000000	1.21359200	-0.17513100
C	0.00000000	1.20541100	-1.56778900
C	0.00000000	0.00000000	-2.26705700
C	0.00000000	-1.20541100	-1.56778900
C	0.00000000	-1.21359200	-0.17513100
C	0.00000000	0.00000000	0.50938600
H	0.00000000	2.15037300	0.38759700
H	0.00000000	2.15517200	-2.11033500
H	0.00000000	-2.15517200	-2.11033500
H	0.00000000	-2.15037300	0.38759700
Cl	0.00000000	0.00000000	2.25099700
H	0.00000000	0.00000000	-3.36041500

Tetrahydroquinoline anion

C	2.71094200	-1.07924900	0.09987800
C	1.40627500	-1.52742600	0.01968200
C	0.27902600	-0.63931000	-0.09456500
C	0.62218300	0.76929500	-0.09715300
C	1.94368900	1.18503600	-0.00061400

C	3.01854400	0.29177100	0.08929000
H	3.52286900	-1.81634600	0.17770200
H	1.17247900	-2.59935300	0.03450700
C	-0.50949300	1.76005000	-0.17089500
H	2.14059300	2.26923800	0.00740800
H	4.05185900	0.64861700	0.15867000
C	-1.78605100	1.13677300	0.36900500
C	-2.02653500	-0.20760400	-0.32239800
H	-0.24837800	2.68744000	0.37681600
H	-1.68086500	0.94789600	1.45734900
N	-0.94816700	-1.13981800	-0.17501900
C	-3.30831200	-0.85353200	0.18159900
H	-3.21728600	-1.05235300	1.26523800
H	-4.20188900	-0.22365900	0.00885400
H	-3.45199600	-1.82795800	-0.31395800
H	-2.20093000	0.04042500	-1.40783700
H	-2.65486100	1.81254100	0.23746500
H	-0.69602800	2.07741400	-1.22006000

INT2-L6

C	0.62088800	-0.22542300	-0.43640400
N	0.15003700	-0.87153700	-1.55210600
N	-0.48869700	0.38671300	0.11019500
C	0.93702800	-1.70233100	-2.41290800
C	2.28589200	-3.35885600	-4.16437800
C	1.25507300	-3.00717400	-1.99591000
C	1.33366000	-1.18816200	-3.65893900
C	2.00755300	-2.04688100	-4.53090900
C	1.92837300	-3.82656200	-2.90530000
H	2.32920300	-1.68103200	-5.51026000
H	2.18907400	-4.84883400	-2.61718500

C	-0.39529400	0.99454800	1.39804900
C	0.04057500	2.04879000	3.93754500
C	-0.71327100	0.21852600	2.51785900
C	0.14732000	2.28865700	1.51103700
C	0.35613600	2.79195300	2.79105200
C	-0.48479200	0.76704500	3.78333100
H	0.80510200	3.78385200	2.91170100
H	-0.69050000	0.15762500	4.67217400
C	0.54416700	3.00785000	0.23429900
H	1.18457300	2.27439300	-0.29650000
C	-1.25520800	-1.18811300	2.34126200
H	-0.95801200	-1.51413900	1.32846700
C	0.27215900	2.63251300	5.29731900
H	0.25952400	1.85995300	6.08050900
C	1.14204100	0.27681600	-3.98810800
H	0.21087200	0.61812200	-3.49711900
C	0.88902600	-3.49852500	-0.61085100
H	0.97649000	-2.62383200	0.06116400
C	1.00151400	0.56544800	-5.47424800
H	1.94378300	0.38571000	-6.02008700
H	0.74131800	1.62498100	-5.63210800
H	0.21549200	-0.04993200	-5.94243000
C	1.84900600	-4.55012200	-0.07570900
H	1.73129500	-5.52130800	-0.58809700
H	1.65370300	-4.72497600	0.99585900
H	2.89848000	-4.22938900	-0.18867900
Ni	2.36170700	-0.02033500	0.17881900
H	-0.50586000	3.37280200	5.55217500
H	1.23842800	3.15899800	5.35009400
C	-0.57918600	-2.18543900	3.26505500

C	-1.24436400	-2.87078700	4.28166900
C	0.78157300	-2.44571900	3.04831900
C	-0.56383000	-3.80081100	5.07057100
H	-2.30993500	-2.68731600	4.45449700
C	1.45441900	-3.38058600	3.82656800
H	1.31022900	-1.89494600	2.25547400
C	0.78319000	-4.06256000	4.84344400
H	-1.09901000	-4.33093700	5.86411400
H	2.51331800	-3.58435800	3.63270700
H	1.31220200	-4.79956900	5.45463200
C	-0.64277700	3.29225100	-0.66751900
C	-1.86068800	3.76267800	-0.16974400
C	-0.51556700	3.09744500	-2.04606300
C	-2.92544700	4.02808800	-1.02985100
H	-1.98691700	3.90065300	0.91111500
C	-1.58187800	3.34485500	-2.90585800
H	0.43909700	2.72996300	-2.43999900
C	-2.79374300	3.81287000	-2.39926200
H	-3.87561200	4.38774600	-0.62229200
H	-1.46682300	3.16620400	-3.97985900
H	-3.63624800	4.00242400	-3.07060300
C	-5.34754800	1.04018700	-0.51649600
C	-5.70986500	0.38134700	-1.67403300
C	-4.73237800	-0.32926400	-2.42771100
C	-3.42934400	-0.30401700	-1.93238400
C	-3.04439900	0.38170700	-0.74251300
C	-4.01217500	1.05026800	-0.02969800
H	-5.94225700	-1.11527000	-4.06582100
H	-6.11338400	1.57018600	0.05802400
H	-6.74994600	0.39660300	-2.01595500

C	-4.93893600	-1.06408500	-3.63025700
C	-2.33210700	-0.96715500	-2.55998700
H	-3.77566800	1.57676600	0.89998000
C	-2.56095200	-1.67497600	-3.71929700
C	-3.88296800	-1.70907400	-4.24272400
H	-1.75244000	-2.20464900	-4.23413700
H	-4.06361200	-2.26986500	-5.16442100
C	-1.61378900	0.12440400	-0.64409700
C	-1.20774200	-0.66203700	-1.69115600
C	4.31044000	0.02423300	-0.13381400
C	3.96318500	0.77700100	1.02533500
C	3.76902900	0.10413900	2.25927500
C	4.00894100	-1.25869200	2.36829100
C	4.36853300	-2.01440200	1.23739000
C	4.46756300	-1.37890600	0.00722200
H	4.62004700	0.50139300	-1.07031400
H	3.97767100	1.87279100	1.01111700
H	3.90464000	-1.75376100	3.33832600
H	4.54874900	-3.09083100	1.30950000
Cl	4.84572100	-2.31191800	-1.41984500
C	-2.77097300	-1.24579600	2.35924100
C	-3.42697400	-2.10168600	1.46732700
C	-3.54477300	-0.48242800	3.23823100
C	-4.81549100	-2.19076300	1.44704800
H	-2.83126500	-2.69619000	0.76467000
C	-4.93620800	-0.57455400	3.22670900
H	-3.05470600	0.20318600	3.93750200
C	-5.57647400	-1.42631400	2.32945800
H	-5.30628400	-2.85393500	0.72843800
H	-5.52452100	0.03008600	3.92372000

H	-6.66842700	-1.49091500	2.31414700
C	1.40584500	4.24058300	0.40235400
C	0.91325500	5.43248600	0.94695400
C	2.72834200	4.21511400	-0.05126000
C	1.72906600	6.55443200	1.06131500
H	-0.13014300	5.48648000	1.27654100
C	3.54773200	5.33772700	0.05763700
H	3.11219900	3.29504000	-0.50974200
C	3.05138000	6.50995400	0.62050000
H	1.32640300	7.47637200	1.49141000
H	4.57876000	5.29556500	-0.30571800
H	3.69040300	7.39343400	0.70743400
C	-0.55022700	-4.00328400	-0.55750100
H	-1.27207000	-3.24774800	-0.91353700
H	-0.67113500	-4.89819800	-1.19365500
H	-0.82617300	-4.28271900	0.47496700
C	2.30369900	1.06825300	-3.38402400
H	3.26420100	0.70944200	-3.79607500
H	2.21937500	2.14521600	-3.61525500
H	2.34583500	0.94266500	-2.28512100
H	2.80858500	-4.01963000	-4.86224900
H	3.48404400	0.68130500	3.14405200

TS3-L6

C	0.50157600	-0.18695100	-0.65191500
N	-0.49612500	0.35130100	0.13308400
N	-0.16639100	-0.99184400	-1.54696600
C	-0.22440500	1.17182300	1.26988000
C	0.26830800	2.74245200	3.52212100
C	-0.03195200	2.55738300	1.10564500
C	-0.12801200	0.55645800	2.52278800

C	0.12353300	1.36252200	3.63786400
C	0.20023900	3.31922300	2.24686700
H	0.24156100	0.88874300	4.62007000
H	0.36026000	4.39862900	2.14472700
C	0.48203400	-1.85856900	-2.48037000
C	1.75789200	-3.54137300	-4.26053900
C	0.76871200	-1.38168900	-3.76994400
C	0.78216100	-3.16910500	-2.07090700
C	1.43320700	-3.99869700	-2.98762800
C	1.42436900	-2.24777500	-4.64892500
H	1.69564400	-5.02088000	-2.69665700
H	1.67517500	-1.90429700	-5.65742300
C	0.47046200	-3.65104900	-0.66962800
H	-0.29664800	-2.97455100	-0.24906500
C	0.43051900	0.03704900	-4.17055100
H	-0.39697100	0.36920500	-3.51707700
C	-0.24491800	-0.95155500	2.65115100
H	-0.03348600	-1.36951300	1.65113700
C	-0.02472800	3.15298800	-0.29487200
H	0.70003200	2.52999200	-0.85825400
C	0.51783100	3.60448000	4.72076400
H	0.69071400	3.00538800	5.62697700
C	1.70681700	-3.53592600	0.21795600
H	2.10766800	-2.50546700	0.19131900
H	2.50660500	-4.21442400	-0.12797200
H	1.46957900	-3.79033000	1.26640400
C	-0.05506000	0.16009600	-5.60740700
H	-0.41416700	1.18535900	-5.79878500
H	-0.88283800	-0.53579500	-5.82260900
H	0.75025400	-0.03462500	-6.33653200

Ni	2.36563900	-0.01241100	-0.49279800
H	1.39462200	4.25472300	4.56930500
H	-0.33870800	4.27124800	4.91815100
C	0.86063400	-1.50077100	3.53449100
C	2.17299400	-1.39060100	3.05487700
C	0.64134800	-2.09663600	4.77588700
C	3.24189600	-1.86325000	3.80431200
H	2.33914500	-0.93800600	2.06432200
C	1.71768800	-2.56636200	5.53231700
H	-0.38009900	-2.20299100	5.15628000
C	3.01753500	-2.45069300	5.05126100
H	4.25820500	-1.77857300	3.40714300
H	1.53264400	-3.03163800	6.50522700
H	3.85875500	-2.82322400	5.64315600
C	-1.35990600	3.01442500	-0.99578200
C	-2.56956000	3.25242200	-0.33820600
C	-1.39548300	2.65663000	-2.34631900
C	-3.78450300	3.11639900	-1.00861000
H	-2.55849900	3.53202400	0.72264900
C	-2.60640400	2.50052800	-3.01504200
H	-0.44889300	2.48493100	-2.87144200
C	-3.80852700	2.72660900	-2.34511000
H	-4.72289300	3.29478900	-0.47419700
H	-2.61036800	2.19458100	-4.06645900
H	-4.76255700	2.59735000	-2.86428900
C	-4.59014400	-2.71098500	-2.82550500
C	-5.51802400	-2.16199900	-1.96324600
C	-5.10369800	-1.26626700	-0.93610700
C	-3.73920200	-0.98849400	-0.86807900
C	-2.77448100	-1.54940200	-1.75784400

C	-3.20117400	-2.41620700	-2.73908800
H	-7.00851700	-0.81011800	0.02856600
H	-4.92927500	-3.39837700	-3.60598600
H	-6.57878800	-2.41463400	-2.06299100
C	-5.92875500	-0.62862200	0.03367800
C	-3.15167000	-0.11621800	0.09603000
H	-2.49834000	-2.87317200	-3.44346500
C	-3.97254200	0.47580300	1.02789300
C	-5.36730300	0.20556900	0.97928700
H	-3.57533000	1.14636500	1.79632700
H	-6.01417200	0.67797400	1.72479700
C	-1.52087200	-0.97328000	-1.30344800
C	-1.73312900	-0.13780500	-0.23860100
C	4.90592300	-0.66005700	0.71311500
C	5.47238000	-1.92466300	0.71818000
C	5.36651700	-2.76768700	-0.40098300
C	4.69485000	-2.33726900	-1.53770900
C	4.10948200	-1.06386700	-1.57928400
C	4.18373400	-0.25221500	-0.42565200
H	4.98171700	0.00902600	1.57644100
H	6.01236800	-2.26912200	1.60654300
H	4.63480700	-2.98313500	-2.42022100
H	3.69413900	-0.67770600	-2.51539600
Cl	3.92813200	1.68093700	-0.63741400
H	2.27043300	-4.20515700	-4.96328100
C	-0.11450000	-5.05588900	-0.63985200
H	-0.99729600	-5.14382900	-1.29496600
H	0.61985400	-5.81731300	-0.95377400
H	-0.42340100	-5.31891400	0.38566300
C	1.62257400	0.95684100	-3.91220500

H	2.49007500	0.64825700	-4.52381200
H	1.38752300	2.00212000	-4.17911900
H	1.92920100	0.93085600	-2.85071700
C	-1.63861100	-1.42782600	3.00677900
C	-2.14013100	-2.57750500	2.38663500
C	-2.44669800	-0.77166400	3.94008100
C	-3.41447500	-3.05523100	2.67938700
H	-1.51748800	-3.09982600	1.64926600
C	-3.71982400	-1.25163300	4.24341900
H	-2.08030300	0.13600300	4.43141200
C	-4.20998600	-2.39226100	3.61170900
H	-3.79026200	-3.94790900	2.17085000
H	-4.33668800	-0.72407900	4.97743100
H	-5.21337400	-2.76195100	3.84198400
C	0.51723900	4.57077200	-0.33395100
C	-0.30855500	5.69829500	-0.32577400
C	1.90402600	4.76010300	-0.34100900
C	0.23833900	6.98124700	-0.31289200
H	-1.39627200	5.57739600	-0.33795900
C	2.45190900	6.03890200	-0.32949200
H	2.55924800	3.88157000	-0.35197800
C	1.61928600	7.15694200	-0.31287000
H	-0.42516900	7.85133600	-0.30854500
H	3.53893300	6.16233800	-0.33796400
H	2.04712400	8.16378200	-0.30679500
H	5.83331000	-3.75653000	-0.38102400

INT4-L6

C	0.52560600	-0.06585000	-0.64244700
N	-0.59583100	0.13244300	0.11571100
N	0.26812400	-1.19664100	-1.36899900

C	-0.73494900	1.16400500	1.10202300
C	-0.99089000	3.14030100	3.04259300
C	-1.24313500	2.41880800	0.72575900
C	-0.30661100	0.89212500	2.40501200
C	-0.44137800	1.90032100	3.36249100
C	-1.38130000	3.38193800	1.72021900
H	-0.08176100	1.71613000	4.38214700
H	-1.76510100	4.37301700	1.45264800
C	1.21209200	-1.79365000	-2.26399600
C	3.07522100	-2.90782000	-3.96592500
C	1.30583600	-1.29674200	-3.57427400
C	2.00811300	-2.85165600	-1.78822700
C	2.94516000	-3.39632600	-2.66893200
C	2.26370000	-1.87126300	-4.41438600
H	3.59150700	-4.21211100	-2.33207200
H	2.37487700	-1.49856700	-5.43740300
C	1.90048200	-3.32649200	-0.35413600
H	0.86417500	-3.12994000	-0.02129100
C	0.45096600	-0.13773400	-4.03391600
H	-0.42078100	-0.09278900	-3.35689100
C	0.30610500	-0.45486100	2.74772700
H	0.75013200	-0.84444000	1.81443200
C	-1.52594700	2.71217600	-0.73975400
H	-0.62413100	2.35530300	-1.27358400
C	-1.10720600	4.22410700	4.06884500
H	-1.00007400	3.83392400	5.09192100
C	2.83520200	-2.52259700	0.54917100
H	2.63873700	-1.43551900	0.48623900
H	3.89042100	-2.67555700	0.26146500
H	2.72207500	-2.81973600	1.60649800

C	-0.08810000	-0.31223900	-5.44660600
H	-0.81087600	0.48865600	-5.67640400
H	-0.60183300	-1.27940700	-5.57320800
H	0.70947500	-0.24867100	-6.20658600
Ni	2.32220400	0.75579800	-0.57983800
H	-0.32276500	4.98611000	3.92259400
H	-2.07447800	4.74654000	3.99745700
C	1.48351300	-0.32175000	3.69808600
C	2.64213100	0.29939800	3.20978100
C	1.47121800	-0.79081500	5.01191600
C	3.76087900	0.44692400	4.02043600
H	2.65438400	0.67092300	2.17568400
C	2.59395500	-0.63608000	5.82798600
H	0.58004000	-1.29128600	5.40475300
C	3.73943400	-0.01886200	5.33630800
H	4.65805900	0.92671600	3.61805500
H	2.57016400	-1.00901100	6.85645400
H	4.61982200	0.09635000	5.97548500
C	-2.69746600	1.92996800	-1.29554700
C	-3.87223900	1.73066900	-0.56718000
C	-2.62515400	1.41489400	-2.59404700
C	-4.93566100	1.01090100	-1.11127600
H	-3.94728200	2.13689800	0.44903200
C	-3.67439000	0.67742100	-3.13428000
H	-1.72106700	1.60116500	-3.18629700
C	-4.83447800	0.46683600	-2.38872400
H	-5.84480700	0.85606300	-0.52174500
H	-3.58637800	0.26389200	-4.14415200
H	-5.65990400	-0.11722600	-2.80581000
C	-3.03928000	-4.76908000	-2.10205900

C	-4.10624900	-4.52483900	-1.26051600
C	-4.10013300	-3.38917100	-0.40215700
C	-2.97987100	-2.56110000	-0.47434900
C	-1.87508600	-2.80564800	-1.34352500
C	-1.90322100	-3.91597500	-2.15794900
H	-6.00792000	-3.62632900	0.63069300
H	-3.06266100	-5.64918200	-2.75115000
H	-4.96240100	-5.20724000	-1.24575700
C	-5.10640400	-3.01291900	0.53168800
C	-2.80861500	-1.38863500	0.31812700
H	-1.07785100	-4.14810000	-2.83870100
C	-3.78938800	-1.05949300	1.22457400
C	-4.93964200	-1.88931600	1.31568600
H	-3.69956700	-0.17859300	1.86749900
H	-5.71676800	-1.62310300	2.03849100
C	-0.97529200	-1.70025700	-1.06012500
C	-1.51469900	-0.87167700	-0.11059400
C	5.17692900	1.46557200	0.11665300
C	6.47642800	1.01383000	-0.11169000
C	6.73574200	0.06375100	-1.10396300
C	5.69526900	-0.43773900	-1.88335800
C	4.39410500	0.02361400	-1.66153800
C	4.13473700	0.94322700	-0.64484600
H	4.96530500	2.21092900	0.89151800
H	7.30228800	1.40228700	0.49365100
H	5.89349700	-1.16736000	-2.67595600
H	3.57237100	-0.36363500	-2.29357700
Cl	2.04579300	2.71060400	0.25357000
H	3.81968800	-3.34469500	-4.63800900
C	2.14429200	-4.81917000	-0.18924000

H	1.51910400	-5.41732700	-0.87246700
H	3.19935100	-5.08736900	-0.36913500
H	1.91043600	-5.12778300	0.84294600
C	1.21813200	1.17712900	-3.89596900
H	2.10598600	1.18245400	-4.55332400
H	0.58745400	2.03868200	-4.17655400
H	1.57056200	1.34178000	-2.86079900
C	-0.73189700	-1.47678700	3.16734200
C	-0.64101600	-2.78662200	2.68520500
C	-1.78517600	-1.16058900	4.03118000
C	-1.58034200	-3.75192400	3.03876100
H	0.18379200	-3.04942200	2.01130100
C	-2.72117600	-2.12583200	4.39764200
H	-1.87950700	-0.13837200	4.41437100
C	-2.62620200	-3.42338600	3.89845300
H	-1.49583700	-4.76584700	2.63627000
H	-3.53795400	-1.85859200	5.07534700
H	-3.36907700	-4.17680600	4.17605200
C	-1.62062900	4.19750300	-1.04652500
C	-2.84225500	4.86827500	-1.14986700
C	-0.43667600	4.92863800	-1.20629700
C	-2.88193100	6.24024400	-1.39706400
H	-3.78017200	4.31363000	-1.04471200
C	-0.47669900	6.29715700	-1.45579300
H	0.52461000	4.40885000	-1.11339900
C	-1.70003400	6.95948600	-1.55053100
H	-3.84799300	6.74811200	-1.47594000
H	0.45868800	6.85132500	-1.57846600
H	-1.73126700	8.03503200	-1.74867600
H	7.76039900	-0.27989400	-1.27546100

INT5-L6

C	-0.12909000	-0.29217000	-0.83798900
N	-0.77436500	0.41945300	0.16063500
N	-1.12763300	-0.62830100	-1.72460800
C	-0.15393800	0.74390700	1.41635100
C	1.23290200	1.20620000	3.79535100
C	0.57549300	1.94095400	1.56037800
C	-0.27514500	-0.17897200	2.47380400
C	0.44703600	0.06403800	3.64236900
C	1.25258900	2.14640800	2.76335100
H	0.41158000	-0.67334300	4.45255900
H	1.85637600	3.05267000	2.88165500
C	-1.05298400	-1.43782400	-2.91002200
C	-1.22137700	-3.01528400	-5.18858700
C	-1.07157600	-0.81314100	-4.17189400
C	-1.13213300	-2.84120200	-2.76815000
C	-1.20416600	-3.61036800	-3.93173800
C	-1.15707600	-1.63504700	-5.30181700
H	-1.25860500	-4.69955800	-3.85411100
H	-1.16908700	-1.17140900	-6.29343900
C	-1.21941800	-3.51018100	-1.40971500
H	-0.58151400	-2.92812000	-0.71731600
C	-1.02455300	0.68822600	-4.36572500
H	-0.73966700	1.13673100	-3.40056700
C	-1.24185000	-1.35045800	2.38345500
H	-1.33324400	-1.61994400	1.31611300
C	0.66274200	2.92043900	0.40169200
H	0.94604400	2.30577800	-0.47011300
C	2.07525300	1.39751800	5.01783700
H	1.62380900	0.92796800	5.90609900

C	-0.70967500	-4.94434500	-1.39948300
H	0.28212600	-5.03977400	-1.86738300
H	-1.40687700	-5.62826400	-1.91594200
H	-0.62003200	-5.30006000	-0.35929100
C	-2.37280600	1.25879500	-4.79712800
H	-2.27788400	2.34162200	-4.98773200
H	-3.15475700	1.12602400	-4.03319000
H	-2.72954700	0.78991400	-5.73286300
Ni	1.75524300	-0.41219400	-1.01919500
H	3.06786300	0.93536100	4.87381800
H	2.24540800	2.46392200	5.23349300
C	-0.80881300	-2.62910000	3.08723100
C	-0.21753800	-3.65575000	2.34632300
C	-1.03326300	-2.84190200	4.45364600
C	0.13718300	-4.86124800	2.94861800
H	-0.01125900	-3.50187600	1.28054400
C	-0.66341200	-4.03838400	5.06207700
H	-1.52793100	-2.06778200	5.05010000
C	-0.08042900	-5.05641300	4.30930100
H	0.60474400	-5.64207800	2.34101500
H	-0.84619500	-4.18072000	6.13193000
H	0.20172800	-6.00128000	4.78403300
C	-0.66746100	3.55468600	0.04358700
C	-1.50106200	4.14672100	0.99710300
C	-1.05980100	3.58686300	-1.30090800
C	-2.69045000	4.76831000	0.61895800
H	-1.22257200	4.10157600	2.05769300
C	-2.26048000	4.18469000	-1.67636800
H	-0.39767000	3.12344800	-2.04466100
C	-3.07885800	4.78294400	-0.71838200

H	-3.33259100	5.22398400	1.38065300
H	-2.55908500	4.18563000	-2.72976500
H	-4.02313600	5.25160500	-1.01288900
C	-5.94802400	-0.28573300	-2.60774100
C	-6.50561000	0.49959000	-1.61762000
C	-5.69111100	1.02609700	-0.57620800
C	-4.33291500	0.71381700	-0.62987200
C	-3.74466700	-0.07163800	-1.66440700
C	-4.56000300	-0.58764800	-2.64883800
H	-7.16897700	2.10816200	0.61428400
H	-6.59226600	-0.69052300	-3.39426700
H	-7.57955500	0.71530900	-1.62371200
C	-6.11453400	1.82568900	0.52445600
C	-3.37823900	1.12320700	0.34960500
H	-4.15579700	-1.21223000	-3.45387700
C	-3.82224500	1.87903800	1.40752200
C	-5.19998200	2.22729400	1.47422600
H	-3.14204200	2.20979500	2.19622400
H	-5.53898700	2.83069200	2.32267700
C	-2.33659000	-0.10898100	-1.30475300
C	-2.12044600	0.55555700	-0.13037700
C	2.48687800	-2.00536800	1.32449000
C	2.98185300	-3.16662200	1.91515100
C	3.02837500	-4.36425200	1.20036600
C	2.55459500	-4.38152900	-0.11028700
C	2.06680900	-3.20994100	-0.69674200
C	2.04510500	-1.98400500	-0.01065500
H	2.49809100	-1.07861200	1.91013400
H	3.33779300	-3.13494400	2.95156800
H	2.57893400	-5.31246900	-0.69110500

H	1.73484900	-3.24838200	-1.74492700
C	4.83457200	1.37146100	1.81559800
C	4.01351400	0.84648000	0.83238500
C	4.50730300	0.01816400	-0.21440400
C	5.90352200	-0.30019400	-0.16005700
C	6.70143400	0.23360700	0.84811900
C	6.20295800	1.08177900	1.83875900
H	4.39619600	2.03709400	2.57163400
H	2.94363400	1.08119600	0.82593400
C	6.47792700	-1.24256000	-1.18232400
H	7.76576900	-0.04185000	0.85577300
H	6.85896400	1.49469400	2.61133000
C	5.37868600	-2.08793300	-1.80134300
C	4.23023900	-1.19494600	-2.27168800
H	7.00698700	-0.68375000	-1.98231500
H	5.76641600	-2.69220800	-2.64257800
N	3.67246000	-0.43174800	-1.17449500
C	4.64248400	-0.28731900	-3.42824300
H	5.39411100	0.45117100	-3.09992500
H	5.07229100	-0.86896300	-4.26548000
H	3.77209600	0.28246200	-3.78753100
H	3.42847100	-1.86243300	-2.65123000
H	4.97852600	-2.79022600	-1.04537000
H	7.24926100	-1.88096500	-0.71234700
Cl	1.65130000	1.37511900	-2.50067900
H	-1.28406400	-3.63773600	-6.08668500
C	-2.65485800	-3.49538200	-0.88559600
H	-3.05626900	-2.47725200	-0.76207100
H	-3.32317200	-4.03174800	-1.58390700
H	-2.71073200	-4.00812800	0.09140800

C	0.05539700	1.08550300	-5.36650600
H	0.20705900	2.17726900	-5.33727600
H	1.02041900	0.62169100	-5.11340200
H	-0.22156300	0.81352800	-6.40156100
C	1.76409800	3.96053200	0.52792800
C	2.95723800	3.77262700	-0.17913600
C	1.63746500	5.10050100	1.33210900
C	4.00394900	4.68521100	-0.05907800
H	3.05975200	2.90080700	-0.83519000
C	2.68241300	6.01322900	1.45146200
H	0.70439800	5.28176800	1.87623900
C	3.87435800	5.80422000	0.75844500
H	4.93318400	4.50665600	-0.60834500
H	2.56343600	6.89520900	2.08943900
H	4.69938100	6.51751400	0.85395300
C	-2.63840800	-0.96618000	2.85770200
C	-3.74630100	-1.59354000	2.27753800
C	-2.86135600	-0.06606200	3.90340500
C	-5.03754300	-1.33025900	2.72227700
H	-3.58375500	-2.29908400	1.45564000
C	-4.15317900	0.18944300	4.36284300
H	-2.01087900	0.45096200	4.36145800
C	-5.24675100	-0.43869700	3.77329500
H	-5.88783700	-1.82031500	2.23803300
H	-4.30409500	0.89583100	5.18566500
H	-6.26148600	-0.22783700	4.12468900
H	3.42576300	-5.27460900	1.66290100

Chloride

Cl	0.00000000	0.00000000	0.00000000

INT6-L6

C	-0.06867100	-0.49757300	-0.47904900
N	0.85687300	0.07417000	0.35792600
N	0.46921900	-1.70674900	-0.83443000
C	0.76688100	1.42318200	0.83596400
C	0.49657900	4.06090400	1.67458500
C	-0.04274300	1.70139700	1.94958800
C	1.46528600	2.42801500	0.14094600
C	1.29288500	3.74250600	0.57360400
C	-0.13980900	3.02805900	2.36501700
H	1.77024100	4.55835100	0.02278200
H	-0.75989400	3.27231600	3.23395800
C	-0.12793500	-2.60157700	-1.77989100
C	-1.06249000	-4.47208100	-3.58211900
C	-1.00653600	-3.59772100	-1.31952400
C	0.22931100	-2.46911800	-3.13310600
C	-0.24979900	-3.43397000	-4.02321400
C	-1.45173000	-4.54060300	-2.24884600
H	0.02057600	-3.37167200	-5.08211700
H	-2.12640200	-5.33796300	-1.92324500
C	1.10744700	-1.33641600	-3.62462100
H	1.16151300	-0.58233200	-2.81631800
C	-1.45277000	-3.65848100	0.12602300
H	-1.33178400	-2.63806400	0.53887800
C	2.38988900	2.05817600	-1.01245400
H	1.99510800	1.12057900	-1.43882300
C	-0.76461600	0.56239800	2.65846100
H	-1.22287900	-0.04328200	1.84985900
C	0.28382900	5.48776500	2.07232500
H	1.15818600	6.11463500	1.83898500
C	0.50584400	-0.64098700	-4.84024900

H	-0.50185800	-0.24931600	-4.62143100
H	0.42704400	-1.31762400	-5.70764000
H	1.14045000	0.20417900	-5.15710600
C	-0.57237400	-4.60633900	0.93467800
H	-0.90171300	-4.64164500	1.98760200
H	0.48923000	-4.30567500	0.91831500
H	-0.63648300	-5.63254800	0.53128900
Ni	-1.88741800	-0.26089800	-0.91862300
H	-0.57538700	5.91322200	1.52446100
H	0.06307800	5.58478800	3.14624600
C	2.45234500	3.02497800	-2.18358000
C	1.95923200	2.61787900	-3.42698600
C	3.05762100	4.28451800	-2.10031800
C	2.01057100	3.45479900	-4.53892100
H	1.52470200	1.61717400	-3.52411500
C	3.10305500	5.13079700	-3.20525300
H	3.52914400	4.60435300	-1.16556300
C	2.57060500	4.72449900	-4.42761500
H	1.60927300	3.10867500	-5.49647800
H	3.57314500	6.11430900	-3.11301200
H	2.60966400	5.38990600	-5.29467700
C	0.22472900	-0.33444800	3.37436300
C	1.12591800	0.19564300	4.30409700
C	0.24073400	-1.71117200	3.14128500
C	2.01491600	-0.63114600	4.98568700
H	1.12025200	1.27527400	4.49720100
C	1.14052700	-2.54120600	3.80931900
H	-0.45920600	-2.13700700	2.41144400
C	2.02875700	-2.00324700	4.73668700
H	2.71040700	-0.20011300	5.71276700

H	1.14626400	-3.61539100	3.59930300
H	2.73779000	-2.65107400	5.26046800
C	4.24108400	-4.75933700	-0.01950600
C	5.06805100	-4.15729200	0.90916100
C	4.76352700	-2.86132500	1.41073500
C	3.61019800	-2.25542900	0.91071600
C	2.74659800	-2.87459700	-0.04006800
C	3.06437200	-4.13178100	-0.50845500
H	6.41326800	-2.54577000	2.80552400
H	4.49300500	-5.75672500	-0.39110000
H	5.96222100	-4.67764400	1.26773600
C	5.50020200	-2.11894800	2.37783100
C	3.15431800	-0.96407800	1.30984200
H	2.42873700	-4.64795900	-1.23627100
C	3.88353000	-0.27381600	2.24869600
C	5.06258700	-0.87216500	2.77270900
H	3.57715400	0.71710400	2.59543700
H	5.64018400	-0.31376400	3.51581000
C	1.68785100	-1.89481100	-0.22146300
C	1.92685100	-0.78119100	0.54316000
C	-3.02525600	2.32876300	-0.54366600
C	-3.10578600	3.71249200	-0.71607800
C	-2.18043000	4.38111500	-1.51734100
C	-1.17707700	3.65070500	-2.15103300
C	-1.09117200	2.27070900	-1.95945800
C	-1.99775000	1.58672300	-1.14227200
H	-3.79266000	1.82584300	0.06049600
H	-3.91282100	4.27053800	-0.22807100
H	-0.44014700	4.15465600	-2.78905500
H	-0.28618600	1.71755200	-2.45548400

C	-5.27141600	-0.57942100	1.99438100
C	-4.32156300	-0.78358700	1.00649000
C	-4.61295300	-0.59270400	-0.36369900
C	-5.93192900	-0.20422400	-0.71032600
C	-6.87471700	-0.00980700	0.30156300
C	-6.56649700	-0.18844000	1.64721700
H	-4.99683200	-0.72448500	3.04482000
H	-3.30743800	-1.10735600	1.27548900
C	-6.29315500	0.01890100	-2.15267400
H	-7.88466100	0.30708900	0.01269700
H	-7.32578900	-0.02523100	2.41743700
C	-5.05638800	0.28065200	-2.99297500
C	-3.98037700	-0.75986300	-2.70109200
H	-6.83344000	-0.86587900	-2.54613700
H	-5.30256500	0.28394500	-4.06938800
N	-3.59821400	-0.75701000	-1.28939700
C	-4.37195600	-2.16485500	-3.14049500
H	-5.18245400	-2.57362600	-2.51226200
H	-4.71217600	-2.17955200	-4.19072400
H	-3.50874000	-2.84548100	-3.04667500
H	-3.07651800	-0.47603700	-3.27764800
H	-4.64239500	1.27679300	-2.75067600
H	-7.00958100	0.85540600	-2.23282200
H	-1.41810000	-5.22427400	-4.29218400
C	2.53061500	-1.81192800	-3.89948000
H	3.17241300	-0.96485800	-4.19945400
H	2.98221300	-2.28316500	-3.00961500
H	2.55119100	-2.55280100	-4.71803500
C	-2.92670500	-4.00987200	0.26843100
H	-3.13970500	-5.04779800	-0.03980800

H	-3.23796000	-3.91700600	1.32189900
H	-3.54974700	-3.32343600	-0.32888200
C	-1.92883200	0.99111300	3.54029200
C	-2.09182500	0.53707300	4.85220000
C	-2.92963000	1.80710800	2.99464700
C	-3.21310200	0.90076500	5.60043400
H	-1.34000400	-0.11683100	5.30357300
C	-4.04517900	2.17238200	3.73807300
H	-2.82849500	2.15356700	1.96112600
C	-4.19143800	1.72206400	5.05012400
H	-3.31758500	0.53293100	6.62565700
H	-4.81576100	2.80029500	3.28024800
H	-5.06951700	2.00582300	5.63767800
C	3.80578500	1.73911400	-0.55390500
C	4.50550200	0.70752400	-1.18901700
C	4.45566100	2.47343400	0.44083800
C	5.81841100	0.41130500	-0.83685100
H	4.00082900	0.12082500	-1.96779700
C	5.77555500	2.18761800	0.78737500
H	3.91755000	3.27048500	0.96624500
C	6.46007800	1.15435500	0.15293100
H	6.34263800	-0.40983200	-1.33485300
H	6.26982300	2.77427400	1.56795600
H	7.49174000	0.92251900	0.43319000
H	-2.24840200	5.46482700	-1.65560300

TS7-L6

C	0.13307200	-0.49962600	-0.44679300
N	0.98484700	0.48377500	0.01324000
N	0.88437400	-1.22287200	-1.33750700
C	0.64353000	1.30732300	1.12955800

C	-0.11189800	2.81497500	3.34694600
C	1.01527500	0.86967100	2.40706600
C	-0.09580900	2.48607400	0.92940200
C	-0.44091600	3.23371500	2.05165100
C	0.60490200	1.63013500	3.50575900
H	-1.02229500	4.15370200	1.92179100
H	0.82752800	1.26829400	4.51699700
C	0.44846500	-2.43714400	-1.96244000
C	-0.39159600	-4.76930500	-3.16841800
C	0.44936500	-3.62264200	-1.20229000
C	0.05935000	-2.39332800	-3.31017400
C	-0.35948400	-3.58831400	-3.90093500
C	0.01006400	-4.78646300	-1.83719900
H	-0.67764600	-3.59388100	-4.94745900
H	-0.02331800	-5.72571100	-1.27825900
C	0.02747600	-1.07986400	-4.06445300
H	0.84006100	-0.44303700	-3.66454200
C	0.97553600	-3.65012100	0.21974100
H	0.66105000	-2.70429200	0.70265200
C	-0.52110900	2.86168100	-0.48259300
H	-0.93949400	1.92401900	-0.90456200
C	1.82325400	-0.40306600	2.58204600
H	1.76105100	-0.95364900	1.62761700
C	-0.58324200	3.60086900	4.53083300
H	-0.46861000	4.68437000	4.36953900
C	-1.29165200	-0.36434400	-3.78425400
H	-1.44706100	-0.24393800	-2.69728800
H	-2.13994000	-0.95354600	-4.17657400
H	-1.32075100	0.63120900	-4.26154400
C	2.50254000	-3.71904200	0.21933800

H	2.89049900	-3.69804500	1.25327500
H	2.97272300	-2.89582200	-0.34323500
H	2.83776000	-4.66447300	-0.24284700
Ni	-1.68218600	-0.51435900	0.03034500
H	-1.65639000	3.41905900	4.71642100
H	-0.04050800	3.32871400	5.44831000
C	-1.66039800	3.86307200	-0.51279100
C	-2.89981000	3.44508300	-0.01102800
C	-1.54920900	5.16012900	-1.01555800
C	-3.99787000	4.29540800	-0.00445800
H	-2.99494900	2.42431100	0.37538700
C	-2.64986400	6.02010900	-1.00595900
H	-0.59818100	5.50692200	-1.43092100
C	-3.87434700	5.59382700	-0.50141300
H	-4.95814800	3.93302500	0.37764900
H	-2.54504400	7.03325600	-1.40580400
H	-4.73548700	6.26852400	-0.50205300
C	3.30249900	-0.12635500	2.78594800
C	3.77531400	0.82506800	3.69631900
C	4.23937800	-0.83629600	2.02728100
C	5.14151800	1.05783900	3.84135700
H	3.06773100	1.40365500	4.29781300
C	5.60554900	-0.60521300	2.16636200
H	3.88595600	-1.57383300	1.29887700
C	6.06243700	0.34565800	3.07531900
H	5.48872300	1.80728600	4.55901000
H	6.31464700	-1.16577500	1.54979700
H	7.13426700	0.53470600	3.18504600
C	5.29349400	-1.45611200	-3.52082300
C	6.07178400	-0.43935500	-3.00349200

C	5.54362000	0.43740500	-2.01355200
C	4.22526600	0.20775400	-1.62140000
C	3.41560600	-0.84368500	-2.14772500
C	3.95319500	-1.67957300	-3.10156200
H	7.25020400	1.74763900	-1.64387800
H	5.71903800	-2.11931600	-4.27949800
H	7.10084600	-0.30288600	-3.35180000
C	6.21311700	1.52085100	-1.37566900
C	3.54032500	0.99059600	-0.64612500
H	3.37187900	-2.50237100	-3.53148900
C	4.21521000	2.02262600	-0.03711600
C	5.56102900	2.27422700	-0.42023000
H	3.74242800	2.64084600	0.73220000
H	6.09526000	3.09508500	0.06736900
C	2.15440000	-0.68942000	-1.44006200
C	2.22119200	0.37800900	-0.58402400
C	-3.46012700	-2.25574000	-1.44647000
C	-3.81313700	-3.55324900	-1.79285700
C	-3.66074000	-4.60962500	-0.89301200
C	-3.11908100	-4.33260100	0.36009300
C	-2.73774800	-3.03983000	0.71310200
C	-2.90951100	-1.95564100	-0.17872400
H	-3.62173000	-1.45856300	-2.17596300
H	-4.22362400	-3.73872900	-2.79137300
H	-2.97457600	-5.14196600	1.08525200
H	-2.27886500	-2.88786200	1.69547000
C	-5.08432200	1.42886700	-2.26270400
C	-4.13213300	0.81254600	-1.46596600
C	-4.45884700	0.25610700	-0.21539200
C	-5.79248500	0.36491500	0.23683800

C	-6.73316700	1.00775200	-0.57408300
C	-6.40269300	1.53294500	-1.81795200
H	-4.78851000	1.85099800	-3.22789400
H	-3.08562500	0.76919100	-1.78555700
C	-6.20293800	-0.16058900	1.58443300
H	-7.76360900	1.08355800	-0.20589700
H	-7.16230700	2.02489400	-2.43176700
C	-5.16000000	-1.08866000	2.17505800
C	-3.76103500	-0.51615600	1.97707100
H	-6.37828800	0.69488200	2.26701400
H	-5.35161500	-1.26698400	3.24731200
N	-3.46678100	-0.38607100	0.54498600
H	-5.19681100	-2.07214200	1.67145700
H	-7.17964400	-0.66879800	1.50300700
H	-0.73568600	-5.69322600	-3.64250200
C	0.26726500	-1.22251200	-5.55903700
H	1.19095600	-1.78418200	-5.77636300
H	-0.56896500	-1.73601900	-6.06370000
H	0.35738900	-0.22803700	-6.02585200
C	0.43104400	-4.78488900	1.07301700
H	0.83269900	-5.76390300	0.75712100
H	0.73218600	-4.63427800	2.12406400
H	-0.66709200	-4.83529800	1.03675900
C	1.18580900	-1.34424300	3.58930000
C	1.87085400	-1.88994700	4.67445300
C	-0.14588100	-1.72850000	3.36681000
C	1.23729600	-2.79617700	5.52838100
H	2.91466900	-1.61235600	4.85341200
C	-0.77523000	-2.62954400	4.21730600
H	-0.67952100	-1.31754400	2.49468900

C	-0.08408900	-3.16729200	5.30522500
H	1.78868200	-3.21570500	6.37515000
H	-1.81370000	-2.92355200	4.02915200
H	-0.57801300	-3.87775500	5.97428400
C	0.67249000	3.22569100	-1.33855500
C	0.88530500	2.58185100	-2.56027800
C	1.59491800	4.19140800	-0.92258800
C	1.99772700	2.88200300	-3.34382800
H	0.17289500	1.81403700	-2.88577500
C	2.69873200	4.50764200	-1.71142200
H	1.44544600	4.69459200	0.04066500
C	2.90786800	3.84867300	-2.92190000
H	2.15634200	2.34978300	-4.28755400
H	3.41208800	5.26463700	-1.37065300
H	3.78596000	4.08216200	-3.53120000
H	-3.95789500	-5.62644300	-1.16395500
C	-3.52828300	0.79745500	2.70914600
H	-4.14404400	1.61832100	2.30231100
H	-3.77034100	0.69263500	3.78105100
H	-2.47108100	1.10207800	2.61915000
H	-3.03800900	-1.23724300	2.38886000

INT8-L6

C	-0.05236500	-0.53558200	0.04512000
N	1.00380100	0.36331700	-0.07290400
N	0.08685700	-1.02546800	1.33585400
C	1.31755300	0.97721000	-1.31839800
C	1.83526200	2.09691300	-3.81845000
C	0.66004700	2.16255700	-1.68303200
C	2.23463800	0.34432900	-2.17056000
C	2.45926800	0.91032000	-3.42679700

C	0.94713100	2.71372500	-2.93037300
H	3.13763100	0.40488900	-4.12530000
H	0.44195800	3.63764000	-3.23600200
C	-0.73903200	-2.02772400	1.93360400
C	-2.14399300	-3.98944200	3.29373400
C	-1.76789800	-1.63775800	2.81128800
C	-0.45086100	-3.38051300	1.66808800
C	-1.17041100	-4.35154100	2.36865100
C	-2.44873100	-2.64913000	3.49802000
H	-0.96065600	-5.41084900	2.19150800
H	-3.24481500	-2.38067000	4.19877700
C	0.61562800	-3.76980600	0.66622300
H	0.64470100	-2.95699600	-0.08326900
C	-2.14275700	-0.18208000	3.01411500
H	-1.90782000	0.33923400	2.06494300
C	2.97419100	-0.89597000	-1.69971700
H	2.34969500	-1.34956700	-0.91128500
C	-0.32367500	2.78118900	-0.70245900
H	-0.89592900	1.92484000	-0.29534300
C	2.12417700	2.71238900	-5.15345600
H	2.91619200	3.47778300	-5.07781100
C	0.29339200	-5.05462600	-0.08436900
H	-0.73528700	-5.05083600	-0.48281700
H	0.40205400	-5.94868100	0.55437500
H	0.98909200	-5.18022000	-0.93192100
C	-1.33790200	0.47455700	4.13320700
H	-1.67106300	1.51670100	4.28114700
H	-0.25627900	0.49203500	3.93162500
H	-1.49236400	-0.06249700	5.08667200
Ni	-1.23282900	-1.07191800	-1.27625700

H	2.46802100	1.96160000	-5.88116200
H	1.23604400	3.21303000	-5.56935700
C	3.09464000	-1.97128000	-2.76329600
C	2.03784000	-2.87836700	-2.90162900
C	4.20359700	-2.10333600	-3.60263200
C	2.07881800	-3.88420000	-3.86288000
H	1.16486800	-2.76545900	-2.24572600
C	4.24548800	-3.10813900	-4.56920100
H	5.05360000	-1.42143200	-3.49004000
C	3.18496600	-4.00081000	-4.70390000
H	1.24073300	-4.58395500	-3.95382700
H	5.12186600	-3.19822900	-5.21804900
H	3.22314000	-4.79151200	-5.45881000
C	0.39680600	3.40974900	0.47191600
C	1.40541600	4.35903100	0.27534200
C	0.06445100	3.04951400	1.78073200
C	2.06347800	4.93417200	1.35973100
H	1.67637500	4.64679800	-0.74774600
C	0.72968100	3.61341300	2.86846700
H	-0.71623900	2.29562100	1.94828900
C	1.73131100	4.55819000	2.66078400
H	2.84960400	5.67599700	1.18789200
H	0.46680000	3.30426400	3.88487400
H	2.25953800	4.99653700	3.51253200
C	2.92979500	-0.90518000	5.36430400
C	3.92094400	0.03277800	5.15117300
C	3.98375900	0.74186700	3.91814000
C	3.00543500	0.43377900	2.97394700
C	1.96818900	-0.52220200	3.19079000
C	1.93612200	-1.19902400	4.39123300

H	5.72951800	2.01385500	4.24293400
H	2.90075400	-1.44288000	6.31661300
H	4.66509000	0.23431700	5.92879000
C	4.93967400	1.72927400	3.53992100
C	2.92960300	1.03380600	1.68181800
H	1.16219500	-1.94518400	4.60396300
C	3.86866000	1.97869900	1.34241200
C	4.87140800	2.31621400	2.29359000
H	3.86030500	2.46792800	0.36374000
H	5.61715300	3.06755400	2.01571500
C	1.20098500	-0.48492200	1.95420700
C	1.76825600	0.39699400	1.07469300
C	-2.97217900	-0.40821600	-2.39364400
C	-1.90497000	-0.62877300	-3.28503100
C	-1.22496000	-1.86010800	-3.28781700
C	-1.61453100	-2.85687900	-2.36867400
C	-2.67001200	-2.63587400	-1.45646400
C	-3.37198800	-1.40133000	-1.46117200
H	-3.51271300	0.54237300	-2.41744800
H	-1.61677600	0.15790200	-3.99051500
H	-1.08293600	-3.81469100	-2.34860300
H	-2.92987700	-3.41506600	-0.73318800
C	-4.13318100	2.31133500	0.60780100
C	-3.81286100	1.06208200	0.09766600
C	-4.79273400	0.06532100	-0.07329300
C	-6.11583600	0.34674500	0.33304000
C	-6.40859400	1.60913800	0.85820400
C	-5.44331800	2.59998400	0.99090800
H	-3.34304600	3.06591400	0.70627800
H	-2.77766000	0.82620000	-0.17003000

C	-7.20037600	-0.69103300	0.24021600
H	-7.44346300	1.81138000	1.15968500
H	-5.70677300	3.58400300	1.38791400
C	-6.80856100	-1.84405600	-0.66247400
C	-5.38682500	-2.30825000	-0.36713400
H	-7.43263500	-1.06898000	1.25556700
H	-7.50939300	-2.68914100	-0.55198900
N	-4.46004200	-1.19563900	-0.59568900
C	-5.22727500	-2.90986800	1.01990800
H	-5.47181700	-2.18201500	1.81135400
H	-5.89095900	-3.78122400	1.14884400
H	-4.18718500	-3.23268200	1.19493500
H	-5.12917800	-3.07894100	-1.11283800
H	-6.85060500	-1.52627600	-1.71966500
H	-8.13569100	-0.22207300	-0.11167600
H	-2.68634900	-4.76246900	3.84652300
C	1.98962400	-3.87466700	1.32102600
H	2.76061800	-4.10756200	0.56484200
H	2.28097000	-2.94270100	1.83366000
H	2.00083100	-4.68390800	2.07318200
C	-3.62864900	0.01209600	3.28296000
H	-3.91329700	-0.34546900	4.28814700
H	-3.88443400	1.08326300	3.23483800
H	-4.25451100	-0.50458000	2.53768100
C	4.30009800	-0.56604600	-1.03475700
C	4.69514900	-1.31440800	0.07946900
C	5.15131100	0.44437100	-1.49096400
C	5.90103800	-1.06129100	0.72502100
H	4.02920000	-2.09959300	0.45496600
C	6.36527500	0.69582400	-0.85186600

H	4.85618300	1.05719800	-2.34962500
C	6.74252900	-0.05243300	0.26020400
H	6.17948000	-1.64895500	1.60486100
H	7.01792900	1.49158300	-1.22415900
H	7.68872800	0.15412300	0.76891700
C	-1.37018400	3.67585900	-1.34119200
C	-2.18078000	3.12858900	-2.34515600
C	-1.63337100	4.97961900	-0.91639900
C	-3.22450800	3.85444300	-2.90419500
H	-1.98312700	2.10183100	-2.67114800
C	-2.68221300	5.71268000	-1.47681400
H	-1.02518900	5.42888400	-0.12550600
C	-3.48218600	5.15487000	-2.46781300
H	-3.84981500	3.39965400	-3.67883000
H	-2.87480000	6.73096300	-1.12575400
H	-4.30717100	5.72862900	-2.89977200
H	-0.39360100	-2.03357000	-3.97699400

3a

C	-1.80090100	-1.06196900	-0.81670100
C	-3.11920200	-1.48417800	-0.94117300
C	-4.14675400	-0.81953700	-0.27212600
C	-3.83343300	0.27414500	0.53056400
C	-2.51332600	0.69145700	0.67524900
C	-1.47435200	0.03164600	0.00172100
H	-1.00592300	-1.57573800	-1.36502600
H	-3.34879500	-2.33698700	-1.58732300
H	-4.62513400	0.80539600	1.06745000
H	-2.27852000	1.53400700	1.33266900
C	1.91774500	-2.52635200	0.74413900
C	0.83060600	-1.66707000	0.69172500

C	0.94961700	-0.37537200	0.14598600
C	2.21244800	0.05052800	-0.31945900
C	3.29320100	-0.83317300	-0.24569200
C	3.16430700	-2.11604100	0.27016700
H	1.79162800	-3.52496300	1.17307600
H	-0.13648500	-1.98927100	1.08743200
C	2.41905400	1.44004300	-0.85901500
H	4.26569600	-0.48865600	-0.61732900
H	4.02585400	-2.78794400	0.30933900
C	1.10820200	2.14963400	-1.13940000
C	0.11991100	1.92868100	0.00123700
H	3.01614000	2.02578100	-0.13247100
H	1.27001200	3.22952200	-1.29756600
N	-0.15115200	0.49238400	0.11349500
C	0.58697800	2.52287000	1.32087500
H	1.46997900	1.99215300	1.71247100
H	0.85385400	3.58616000	1.20168100
H	-0.19902500	2.45066400	2.08907400
H	-0.83437500	2.40262900	-0.28415900
H	0.65145000	1.75364600	-2.06405900
H	3.04017000	1.39683000	-1.77062900
H	-5.18307100	-1.15178700	-0.37767200

INT1-L2

C	0.14461200	0.17964000	-0.10184900
N	0.75027200	-1.09678900	-0.14688100
N	1.25982000	1.03743800	0.03597500
C	0.00000800	-2.30213000	-0.19647700
C	-1.35498600	-4.71518800	-0.30425000
C	-0.48053300	-2.75711700	-1.43718500
C	-0.23736000	-3.00242700	0.99938700

C	-0.91285100	-4.22341400	0.91914800
C	-1.15632900	-3.97882500	-1.46855800
H	-1.12030600	-4.78682500	1.83475500
H	-1.54641000	-4.35612300	-2.41891300
C	1.13779000	2.44910400	0.10578000
C	0.99108700	5.21157300	0.25580000
C	1.38589400	3.20792300	-1.05152900
C	0.77003300	3.04538400	1.32662300
C	0.70642000	4.43959800	1.37861200
C	1.31648100	4.60059600	-0.95040700
H	0.41984700	4.93229100	2.31282300
H	1.49985900	5.21755200	-1.83617200
C	0.44294200	2.17936100	2.52379300
H	-0.04260500	1.27397400	2.11088000
C	1.57252500	2.52137200	-2.38786100
H	2.02209000	1.52817000	-2.20022100
C	0.08703200	-2.37292400	2.33703800
H	0.91224500	-1.65254800	2.18313700
C	-0.27521100	-1.92037300	-2.68068300
H	-0.27770700	-0.86882400	-2.33823300
C	-0.56377900	2.81482400	3.46856500
H	-1.47102500	3.13690500	2.93046900
H	-0.14845300	3.68867200	4.00351400
H	-0.87417600	2.08506200	4.23491100
C	2.49274300	3.26835600	-3.34074800
H	2.67479300	2.66616400	-4.24623300
H	3.46997500	3.49380200	-2.88055900
H	2.04936600	4.22208800	-3.67774100
C	0.54168700	-3.37068900	3.39128100
H	-0.27334100	-4.05339500	3.68966700

H	0.86266800	-2.84169200	4.30416400
H	1.38755600	-3.98629200	3.04004700
C	-1.41329600	-2.05204800	-3.68017100
H	-1.43879400	-3.04695500	-4.16117300
H	-1.29687400	-1.30746300	-4.48572700
H	-2.38872100	-1.87538100	-3.19634000
Ni	-1.56647500	0.55211000	-0.13126800
C	6.17360900	1.18876500	0.38065700
C	6.64367300	-0.10759200	0.34410200
C	5.72550600	-1.19444400	0.21922700
C	4.37896100	-0.86101600	0.13791600
C	3.87785100	0.47943100	0.17451100
C	4.78751500	1.50824200	0.29819400
H	7.08376400	-2.90850800	0.23168900
H	6.88950900	2.01139500	0.47803700
H	7.71804500	-0.31031800	0.41126800
C	6.03846600	-2.58637700	0.17087400
C	3.32241200	-1.81586400	0.01023700
H	4.46204200	2.55326900	0.33610500
C	3.65317900	-3.15238500	-0.03389100
C	5.02794400	-3.51748700	0.04909500
H	2.88336300	-3.92516400	-0.13194400
H	5.28536400	-4.58093200	0.01389200
C	2.43849800	0.32368500	0.06671800
C	2.11659900	-1.00526400	-0.03966700
H	0.93846500	6.30339000	0.31725300
C	-1.13099200	-1.57456700	2.80394900
H	-1.98976800	-2.24539700	2.98691700
H	-0.91094000	-1.02820900	3.73883200
H	-1.43595000	-0.84198600	2.02996200

C	1.07248500	-2.22123500	-3.32828300
H	1.11639700	-3.27192400	-3.67070400
H	1.24162200	-1.57341700	-4.20614300
H	1.91048900	-2.06321200	-2.62826700
C	1.70806600	1.76792500	3.27021900
H	2.42551500	1.24517300	2.61572700
H	2.22095200	2.65368500	3.68921000
H	1.46564700	1.09114200	4.10815700
C	0.19536700	2.28502000	-3.00915800
H	-0.29215400	3.25057700	-3.23787200
H	0.27615200	1.70960800	-3.94863800
H	-0.46067800	1.73058300	-2.30979100
H	-1.88629100	-5.67139500	-0.34785000
C	-4.77151900	-2.54007900	0.05732300
C	-3.89227800	-1.47551800	-0.02986000
C	-4.32502600	-0.12122100	0.06049200
C	-5.72840700	0.08272700	0.23519900
C	-6.59226600	-1.00888000	0.30958400
C	-6.14547900	-2.32745300	0.23131200
H	-4.37305400	-3.56004100	-0.01483200
H	-2.81453100	-1.63856200	-0.16301400
C	-6.25194600	1.49143600	0.30450400
H	-7.66604400	-0.80541700	0.43087700
H	-6.84701100	-3.16548200	0.29548300
C	-5.30111800	2.43887900	-0.40194600
C	-3.88567800	2.24566200	0.12953100
H	-7.26935100	1.54188400	-0.12751800
H	-5.29271600	2.22580200	-1.48976700
N	-3.41347500	0.88495900	-0.03641500
C	-2.91845100	3.21167500	-0.53014000

H	-2.92201400	3.05479300	-1.62428400
H	-3.18113600	4.26503900	-0.32092300
H	-1.88848900	3.01178900	-0.18191600
H	-3.92172000	2.50308800	1.21999700
H	-5.61343500	3.49290400	-0.27844900
H	-6.35830900	1.81838200	1.36016300

INT2-L2

C	-0.34244900	0.22868100	0.10846400
N	0.12833300	-1.07114800	0.08243300
N	0.81000700	0.99608100	0.09064300
C	-0.72405000	-2.21526400	0.03831100
C	-2.36495700	-4.43259900	-0.05168800
C	-1.27608600	-2.59435100	-1.19843300
C	-0.97036200	-2.91737200	1.22921900
C	-1.80960900	-4.03311600	1.15884600
C	-2.09782300	-3.72297500	-1.21772000
H	-2.03394100	-4.59918500	2.06823600
H	-2.54976800	-4.04568600	-2.16027200
C	0.78337200	2.41902600	0.00314900
C	0.67049300	5.17166400	-0.18724900
C	0.89857600	3.01077100	-1.26511000
C	0.61102300	3.16873800	1.17997500
C	0.56158800	4.55901300	1.05839100
C	0.83650300	4.40623800	-1.33556100
H	0.42950000	5.17700700	1.95147300
H	0.91189100	4.90154900	-2.30888900
C	0.50996500	2.47750200	2.52331200
H	-0.01873800	1.52246700	2.34669800
C	1.00191900	2.17242700	-2.52365900
H	1.35323400	1.16457800	-2.23448400

C	-0.39718100	-2.44473500	2.55002100
H	0.52110500	-1.86848700	2.33053500
C	-0.96127200	-1.81757300	-2.46012200
H	-0.83479200	-0.76193600	-2.15760700
C	-0.29993400	3.25601300	3.54843500
H	-1.28443700	3.55665400	3.15294800
H	0.22238000	4.16851900	3.88557300
H	-0.47102200	2.63748300	4.44488800
C	2.00058300	2.72628000	-3.53019400
H	2.12228900	2.02565700	-4.37221800
H	2.99284300	2.88626800	-3.07728000
H	1.66586500	3.68618200	-3.95972800
C	-0.00275500	-3.58606500	3.47611600
H	-0.88289200	-4.13956500	3.84621500
H	0.52269500	-3.19507200	4.36241000
H	0.66472600	-4.30783400	2.97698800
C	-2.08150800	-1.85156400	-3.48843900
H	-2.18489600	-2.84663000	-3.95627200
H	-1.86919700	-1.13837500	-4.30244200
H	-3.05198200	-1.58465200	-3.04015800
Ni	-2.09522100	0.83982000	0.17701300
C	5.73618100	0.78014300	-0.10398300
C	6.09522400	-0.55219400	-0.11848500
C	5.09331800	-1.56453400	-0.07681500
C	3.77200500	-1.12701500	-0.02392200
C	3.38970900	0.24900300	-0.00579000
C	4.37943100	1.20585200	-0.04615800
H	6.31215600	-3.37583800	-0.12622100
H	6.51935900	1.54318700	-0.13618300
H	7.15111900	-0.83824900	-0.16228700

C	5.29404900	-2.97520400	-0.08587200
C	2.64584300	-2.00362900	0.01578800
H	4.14150800	2.27467000	-0.02976000
C	2.86883900	-3.36252900	0.00423400
C	4.21173100	-3.83065000	-0.04577100
H	2.03902400	-4.07683000	0.02775400
H	4.38588000	-4.91056100	-0.05479200
C	1.93622700	0.20125100	0.05605100
C	1.50650500	-1.09872600	0.05331700
C	-3.91766500	0.41379900	1.18630500
C	-3.39961500	1.64276800	1.65596400
C	-3.07087800	2.67644300	0.75216500
C	-3.21243100	2.46366000	-0.63379900
C	-3.71512700	1.23422300	-1.12369600
C	-4.07194900	0.22607500	-0.20170000
H	-4.18985300	-0.39107000	1.87488500
H	-3.25685700	1.78748800	2.73197800
H	-2.91899900	3.24738100	-1.33964000
H	-3.83696800	1.06036300	-2.19706800
Cl	-4.74438600	-1.28281700	-0.77822400
H	-2.66533200	3.62420600	1.11988100
H	0.62427200	6.26224700	-0.26226900
C	-1.37388500	-1.49145400	3.23572500
H	-2.31436200	-2.01681100	3.48321200
H	-0.94779700	-1.09771700	4.17479200
H	-1.62299100	-0.64117300	2.57731900
C	0.34838000	-2.29706900	-3.08213100
H	0.26574500	-3.35520500	-3.38906100
H	0.59430400	-1.70530100	-3.98064400
H	1.19941100	-2.21797100	-2.38595000

C	1.89958400	2.16137300	3.07175400
H	2.48629900	1.53083900	2.38411200
H	2.46921600	3.09273500	3.24070400
H	1.82965300	1.62994600	4.03582100
C	-0.38047100	1.99917900	-3.14983800
H	-0.78655900	2.97362200	-3.47719500
H	-0.33323400	1.33833700	-4.03299600
H	-1.08508700	1.56137400	-2.42102400
H	-3.01864700	-5.30923700	-0.08650100

TS3-L2

C	-0.19262700	0.17717300	0.15008200
N	0.32106900	-1.09244000	0.02527800
N	0.89592800	1.00580800	0.07381300
C	-0.48089700	-2.27865400	0.04736700
C	-1.99060600	-4.59084400	0.11446900
C	-1.28581300	-2.58830400	-1.06599500
C	-0.44209600	-3.07805500	1.20234700
C	-1.20757300	-4.24737400	1.20967400
C	-2.03763600	-3.76490400	-1.00316700
H	-1.20080300	-4.89391200	2.09171900
H	-2.67873100	-4.04076100	-1.84402700
C	0.78118100	2.42833100	0.13966200
C	0.50179600	5.16973000	0.26186100
C	0.61379800	3.14041300	-1.05908900
C	0.80495000	3.04825600	1.39771000
C	0.66060500	4.43755700	1.43255200
C	0.47427000	4.52707000	-0.97128700
H	0.65897200	4.95487200	2.39729700
H	0.32837100	5.11375100	-1.88370200
C	0.87327700	2.24546300	2.67936400

H	1.20877600	1.22282200	2.42201700
C	0.48251100	2.42513600	-2.38679100
H	0.93893300	1.42327800	-2.27777000
C	0.30372900	-2.62190000	2.43940100
H	1.20612300	-2.07073700	2.11544500
C	-1.34491500	-1.67084000	-2.27210700
H	-1.43948000	-0.64493100	-1.86110000
C	-0.52650200	2.12891200	3.27959900
H	-1.21298300	1.63334600	2.57059100
H	-0.94609500	3.12690300	3.49685800
H	-0.50800400	1.55425100	4.22108100
C	1.20873800	3.12996600	-3.52311200
H	1.20196200	2.50163800	-4.42870400
H	2.25952100	3.34300000	-3.26561200
H	0.72523800	4.08420500	-3.79392900
Ni	-2.00087600	0.67955200	0.18786200
C	5.78384200	1.08200400	-0.55503000
C	6.20807800	-0.22530800	-0.68259400
C	5.26883200	-1.29428300	-0.61364400
C	3.93707700	-0.93589500	-0.41243700
C	3.48992900	0.41299200	-0.27563900
C	4.41870400	1.42653000	-0.35002800
H	6.56644400	-3.02757000	-0.89316700
H	6.52056300	1.88844900	-0.61266300
H	7.26858800	-0.44737300	-0.83946400
C	5.53793700	-2.68784800	-0.73380600
C	2.86681600	-1.87671800	-0.32740000
H	4.12772900	2.47755800	-0.25542600
C	3.15537700	-3.21770300	-0.44925100
C	4.50894100	-3.60464800	-0.65293900

H	2.36947700	-3.97815700	-0.39484400
H	4.73637000	-4.67015700	-0.74999400
C	2.05333300	0.27934700	-0.09024700
C	1.69170500	-1.04312800	-0.12276500
C	-4.61944900	0.69748100	-1.08108400
C	-5.40608500	-0.43301800	-1.24489700
C	-5.40723900	-1.46142900	-0.28609900
C	-4.63953600	-1.34424700	0.86514600
C	-3.85188200	-0.20216400	1.07716000
C	-3.80755300	0.77861200	0.06262200
H	-4.58939400	1.49439600	-1.83098300
H	-6.02145000	-0.53151800	-2.14536000
H	-4.66189700	-2.13323300	1.62476300
H	-3.35707300	-0.03898000	2.04134200
Cl	-3.13938700	2.64798900	0.53163900
H	0.38596200	6.25637500	0.31155500
C	1.87017200	2.81247200	3.68030400
H	2.87972900	2.89780100	3.24536900
H	1.57169200	3.81214700	4.03941000
H	1.93624800	2.16125900	4.56715800
C	-0.99592300	2.21644000	-2.71181800
H	-1.52043900	3.18462500	-2.79926100
H	-1.12078100	1.66738300	-3.66145800
H	-1.49737000	1.64686800	-1.90672500
H	-6.03301700	-2.34537700	-0.43938100
H	-2.58420600	-5.50959700	0.13602400
C	-0.06620300	-1.75964300	-3.10204700
H	0.83857100	-1.50186100	-2.52828100
H	0.06756800	-2.78534400	-3.49009300
H	-0.11532500	-1.07870900	-3.96871200

C	-2.56195600	-1.89728400	-3.15310200
H	-2.62060800	-1.10729800	-3.91996000
H	-2.51225500	-2.86220100	-3.68860900
H	-3.49924200	-1.86706200	-2.57300200
C	0.77003500	-3.76005100	3.33331400
H	1.35184000	-4.51284800	2.77621100
H	1.40812200	-3.37040100	4.14248500
H	-0.07651800	-4.27638600	3.81768100
C	-0.57701700	-1.64465100	3.21780400
H	-1.49950800	-2.15037200	3.55634500
H	-0.05211100	-1.26070100	4.10897100
H	-0.87914500	-0.78623500	2.59387900

INT4-L2

C	-0.17364900	0.22602600	0.28875500
N	0.13716500	-1.10218400	0.14947100
N	1.00940900	0.88384000	0.13593300
C	-0.84127100	-2.14620100	0.17472600
C	-2.68536600	-4.19365500	0.21712200
C	-1.65722900	-2.33538600	-0.95632500
C	-0.94876900	-2.92762100	1.33834600
C	-1.88751300	-3.96233700	1.33350700
C	-2.57807900	-3.38521000	-0.90878800
H	-2.00388700	-4.59326300	2.21920400
H	-3.23568200	-3.56379700	-1.76375700
C	1.18396000	2.30452600	0.10950700
C	1.54437100	5.02933800	0.02680400
C	1.06779600	2.95283500	-1.12907600
C	1.47791600	2.97046600	1.30551900
C	1.66078600	4.35370400	1.23587000
C	1.24944300	4.33660700	-1.14305900

H	1.88253000	4.91447700	2.14989600
H	1.14904600	4.88463600	-2.08541400
C	1.56175700	2.24757800	2.63304800
H	1.34801900	1.17736700	2.45272600
C	0.69460600	2.20367500	-2.39253900
H	0.85096200	1.12354500	-2.20898100
C	-0.15028700	-2.58168100	2.57793600
H	0.82184200	-2.16780300	2.25101100
C	-1.53948200	-1.44215600	-2.17676200
H	-1.30717300	-0.42298000	-1.80986600
C	0.50044600	2.75568600	3.60348700
H	-0.50239600	2.70803600	3.14874300
H	0.68934900	3.80455800	3.89152000
H	0.50283600	2.15565900	4.52904600
C	1.57305600	2.58415700	-3.57703900
H	1.34403800	1.94530200	-4.44602200
H	2.64434700	2.46775300	-3.34257500
H	1.40770400	3.62814700	-3.89355800
Ni	-2.04202700	0.83635200	0.35642000
C	5.79526800	0.25765300	-0.82556400
C	6.02223700	-1.09839900	-0.94983200
C	4.95065000	-2.02354300	-0.79288000
C	3.69885400	-1.47855800	-0.50939500
C	3.45781300	-0.07877800	-0.37192800
C	4.51013100	0.79393700	-0.53565700
H	5.97066700	-3.92477100	-1.12187500
H	6.63114300	0.95115900	-0.95409000
H	7.02740600	-1.46970500	-1.17398500
C	5.01309200	-3.44187900	-0.90191300
C	2.51581400	-2.25919500	-0.33827000

H	4.37189400	1.87658700	-0.44806500
C	2.60473900	-3.62859000	-0.45326000
C	3.87403400	-4.20434100	-0.73550700
H	1.72583200	-4.27102300	-0.33616700
H	3.94356900	-5.29209500	-0.82585500
C	2.03253300	-0.00713500	-0.08838300
C	1.48603300	-1.26335700	-0.08059200
C	-4.76977300	1.63019900	-0.59842700
C	-5.99888300	1.07162900	-0.94536300
C	-6.31232400	-0.24065300	-0.57720300
C	-5.39755500	-1.00291100	0.14619600
C	-4.16575000	-0.44153200	0.49877900
C	-3.84242200	0.85771900	0.09896600
H	-4.51720000	2.65870400	-0.87813600
H	-6.72580100	1.66275800	-1.51237700
H	-5.64803500	-2.02364400	0.45513500
H	-3.44962800	-1.04389600	1.08756700
Cl	-1.96541100	2.92774900	0.80635200
H	1.67944400	6.11446300	-0.00441600
C	2.96203200	2.33984200	3.22953500
H	3.72574800	1.93453000	2.54474800
H	3.23556200	3.38560900	3.45310200
H	3.02191900	1.77587400	4.17518700
C	-0.78735800	2.39178500	-2.71183100
H	-1.02397400	3.45717700	-2.87871800
H	-1.06402700	1.83466500	-3.62406000
H	-1.42388400	2.04860900	-1.87786100
H	-7.28372200	-0.66586500	-0.84765900
H	-3.41389400	-5.00965100	0.23122200
C	-0.38902500	-1.89452600	-3.07312700

H	0.57550800	-1.92693200	-2.53946500
H	-0.58618500	-2.90674300	-3.46862700
H	-0.27383200	-1.21337100	-3.93322000
C	-2.83200200	-1.32507900	-2.97029100
H	-2.72667200	-0.54445600	-3.74116300
H	-3.08113000	-2.26316600	-3.49650000
H	-3.68526500	-1.04978300	-2.32776700
C	0.14626400	-3.77876500	3.46745300
H	0.61728500	-4.60324800	2.90712700
H	0.83056000	-3.48780600	4.28010900
H	-0.76679900	-4.17145100	3.94676900
C	-0.87726700	-1.48751600	3.36166900
H	-1.85679600	-1.85646000	3.71462900
H	-0.29326300	-1.17957300	4.24480600
H	-1.06300000	-0.58946000	2.74678000

INT5-L2

C	-0.37178400	0.00588500	-0.07233600
N	-1.22011700	1.05842500	-0.31680600
N	-1.20258800	-1.09454800	0.02418100
C	-0.88284300	2.44762600	-0.24297400
C	-0.37827100	5.15697000	-0.08610100
C	-0.50253900	3.12575500	-1.41039800
C	-1.01186700	3.08551800	1.00620000
C	-0.75773100	4.45758800	1.05507600
C	-0.24897400	4.49742700	-1.30271300
H	-0.84025900	4.98405300	2.01150400
H	0.05523500	5.05507900	-2.19468200
C	-0.81571000	-2.43857600	0.34052200
C	-0.29233600	-5.07052600	1.03259000
C	-0.83663300	-3.42102600	-0.66734600

C	-0.52800300	-2.74561000	1.68851500
C	-0.25955900	-4.07807400	2.00706600
C	-0.57650600	-4.74232200	-0.28418000
H	-0.02438300	-4.34949800	3.03951900
H	-0.58283800	-5.52660200	-1.04791300
C	-0.55159000	-1.67911100	2.76686500
H	-0.07356300	-0.77637900	2.34054400
C	-1.11817100	-3.12101100	-2.12695400
H	-1.06116700	-2.02670100	-2.26042000
C	-1.42914100	2.33981300	2.26051200
H	-1.25293200	1.26410600	2.08024600
C	-0.41650400	2.42591700	-2.74835400
H	-0.40925300	1.33634000	-2.57013400
C	0.24043400	-2.05430600	4.01071100
H	1.25319800	-2.41462700	3.76531400
H	-0.26711600	-2.83769200	4.60253200
H	0.35233600	-1.17006600	4.66019700
C	-2.49731000	-3.61687200	-2.55263300
H	-2.66108300	-3.41110100	-3.62389900
H	-3.31484500	-3.13436600	-1.99486500
H	-2.59072600	-4.70916200	-2.40897900
Ni	1.50175700	-0.12066100	-0.16332100
C	-5.94505500	-2.43394900	0.16710400
C	-6.70653600	-1.32127100	-0.12680400
C	-6.07327100	-0.07733900	-0.41331800
C	-4.68018000	-0.07186500	-0.39087100
C	-3.88220500	-1.22401900	-0.11503900
C	-4.52227000	-2.40663300	0.18213900
H	-7.80087200	1.22236500	-0.72460000
H	-6.44803400	-3.37792000	0.39808100

H	-7.79991500	-1.38454100	-0.12856800
C	-6.70754100	1.16853400	-0.69015900
C	-3.88571600	1.09689700	-0.59642800
H	-3.95804000	-3.31396500	0.42439500
C	-4.52543200	2.28937000	-0.85106700
C	-5.94760100	2.30094000	-0.90139900
H	-3.96200700	3.21750600	-0.99657200
H	-6.45190700	3.25014100	-1.10649100
C	-2.51428600	-0.72719100	-0.19312700
C	-2.52134500	0.61898100	-0.42932600
C	2.18872500	2.24944100	1.16055400
C	2.74240600	3.07861800	2.13719500
C	3.08864700	2.56601500	3.38699800
C	2.87767600	1.20987500	3.63403500
C	2.33889100	0.38784300	2.64182400
C	1.97901000	0.87419500	1.37514000
H	1.93449500	2.69617500	0.18980800
H	2.90141000	4.14075400	1.91485400
H	3.14969300	0.77959900	4.60578900
H	2.23380400	-0.68103300	2.86287900
C	5.23400700	1.81190100	-2.05803300
C	4.18667000	1.15132900	-1.44112400
C	4.36506900	-0.09030600	-0.76853800
C	5.69565300	-0.62032000	-0.75813300
C	6.72997000	0.07398900	-1.37900600
C	6.53147100	1.28658500	-2.04057800
H	5.03390300	2.76329300	-2.56665000
H	3.17354900	1.56382500	-1.46512400
C	5.94231200	-1.92511500	-0.05440200
H	7.73778000	-0.36365200	-1.33593400

H	7.36337000	1.80928500	-2.52327000
C	4.90837300	-2.13312600	1.03820900
C	3.49709600	-1.97610300	0.47395400
H	5.89227400	-2.77521400	-0.76705200
H	5.01798900	-3.12495400	1.51524900
N	3.31893200	-0.70013100	-0.17765500
C	3.09761400	-3.13297400	-0.44008500
H	3.67575200	-3.11359500	-1.38056300
H	3.24340800	-4.11327200	0.05212300
H	2.03606500	-3.03396600	-0.72126400
H	2.79772500	-2.01154600	1.34083000
H	5.04990200	-1.36871600	1.82592600
H	6.96775900	-1.94749500	0.35976200
Cl	1.18186100	-0.68745900	-2.37625700
H	-0.08334000	-6.10962800	1.30553800
C	-1.98377800	-1.32674200	3.16879200
H	-2.57796000	-0.91534100	2.33716100
H	-2.51119000	-2.22269500	3.54496700
H	-1.98294200	-0.57502500	3.97725500
C	-0.04888500	-3.71475700	-3.03886800
H	-0.20231800	-3.35445800	-4.06978400
H	0.95692500	-3.38898000	-2.73422100
H	-0.09066900	-4.81889500	-3.06367600
H	3.52230300	3.21379900	4.15646900
C	-2.91555500	2.53408600	2.55475800
H	-3.56457400	2.16489300	1.74498300
H	-3.19818800	2.00285400	3.47993300
H	-3.14295100	3.60534600	2.70372500
C	-0.60624400	2.72541500	3.48320300
H	-0.86638600	3.73620700	3.84624700

H	-0.81300300	2.02587200	4.31168200
H	0.47436600	2.69926600	3.27951200
C	-1.63624500	2.76560300	-3.60048100
H	-2.57833600	2.48137100	-3.10001300
H	-1.68608300	3.84861900	-3.81840400
H	-1.59609500	2.23112300	-4.56436600
C	0.87935000	2.72950300	-3.48591200
H	0.94470400	2.10902800	-4.39433700
H	0.95398500	3.78865500	-3.79300700
H	1.75617800	2.47521300	-2.86892300
H	-0.17412400	6.23056600	-0.02365200

INT6-L2

C	0.40013700	0.03271500	0.23775000
N	1.27154100	-1.00313200	0.04201400
N	1.17005600	1.16883000	0.16949100
C	0.92189300	-2.38283300	-0.13454100
C	0.43524900	-5.06417800	-0.54509400
C	0.62439000	-2.81835300	-1.43616100
C	0.94521800	-3.24285800	0.97682900
C	0.70056700	-4.59694700	0.73836000
C	0.38667100	-4.18364300	-1.61847700
H	0.70269700	-5.30080400	1.57482200
H	0.15543200	-4.56105700	-2.61962600
C	0.65813700	2.50442100	0.27063500
C	-0.17396700	5.12875800	0.43511600
C	0.00464200	3.07031400	-0.83923300
C	0.86728600	3.20249800	1.47271700
C	0.44224800	4.53172900	1.52991600
C	-0.40159500	4.40313800	-0.72843800
H	0.58910300	5.10866800	2.44723000

H	-0.91359000	4.88074900	-1.56800300
C	1.43615300	2.49732400	2.68554700
H	2.16921300	1.74775000	2.33277600
C	-0.23855900	2.27739900	-2.10861300
H	-0.43052600	1.23014400	-1.79970200
C	1.21916900	-2.72184900	2.37347900
H	0.76032300	-1.71542600	2.43005400
C	0.57259700	-1.86804500	-2.61639400
H	0.48536200	-0.83933700	-2.21924700
C	0.31028000	1.74186200	3.39168500
H	-0.20426900	1.04991000	2.70321400
H	-0.44506100	2.45190100	3.77369200
H	0.69430600	1.15852000	4.24551000
C	0.99472000	2.28421700	-3.00792000
H	0.81481100	1.68668100	-3.91773000
H	1.88775200	1.87375200	-2.50659000
H	1.23373500	3.31460300	-3.32598400
Ni	-1.46256600	0.24590900	0.26281000
C	5.76327700	2.79019200	-0.56545300
C	6.56464200	1.68635600	-0.77749200
C	6.00592700	0.37647800	-0.75174800
C	4.63654500	0.29199400	-0.50272200
C	3.80182800	1.42800300	-0.28182500
C	4.36730400	2.68292100	-0.31484000
H	7.76605700	-0.85028600	-1.15262300
H	6.21231300	3.78712900	-0.59116300
H	7.63533300	1.81248600	-0.96777600
C	6.68971000	-0.85599200	-0.95300600
C	3.91607700	-0.93818300	-0.44237400
H	3.76679200	3.58468400	-0.15608700

C	4.60321900	-2.11530600	-0.64129000
C	6.00022800	-2.05097200	-0.89742100
H	4.09479200	-3.08497400	-0.60586700
H	6.54336000	-2.98702100	-1.05530200
C	2.48443500	0.84525700	-0.07851300
C	2.54828300	-0.51969000	-0.16403500
C	-2.47164200	-2.37320000	0.51009200
C	-3.04599500	-3.45259800	1.18139400
C	-3.19031800	-3.42468200	2.56880500
C	-2.74152000	-2.31216500	3.27785500
C	-2.14803200	-1.23985600	2.60657700
C	-2.01062200	-1.25462700	1.21347500
H	-2.40652200	-2.40228500	-0.58602800
H	-3.40017400	-4.31735000	0.61004900
H	-2.85246300	-2.27439100	4.36724200
H	-1.81183400	-0.37471900	3.19261100
C	-4.22423700	-0.62484400	-3.15090400
C	-3.42666000	-0.12588600	-2.13362200
C	-3.97549100	0.47031600	-0.97457100
C	-5.38891700	0.53489100	-0.87729000
C	-6.17220300	0.03241200	-1.91782700
C	-5.61546400	-0.54222800	-3.05624200
H	-3.75626300	-1.07657200	-4.03254700
H	-2.33269300	-0.17310000	-2.21177300
C	-6.02273800	1.11294700	0.35614000
H	-7.26315300	0.08687700	-1.81478900
H	-6.25511500	-0.92720000	-3.85531100
C	-5.05640100	1.05749800	1.52403400
C	-3.71050300	1.65664000	1.13070300
H	-6.33318500	2.16177500	0.17456100

H	-5.46417100	1.58091700	2.40657000
N	-3.11897900	0.95548800	-0.00603900
H	-4.88706600	0.00339600	1.81576000
H	-6.95492600	0.56794200	0.58668100
H	-0.49755300	6.17188500	0.49589400
C	2.16241700	3.42004200	3.65095900
H	2.93876200	4.01872200	3.14646000
H	1.46948400	4.11636500	4.15360800
H	2.65102800	2.83163700	4.44372100
C	-1.47670600	2.72509700	-2.86808600
H	-1.35068300	3.72355800	-3.32143000
H	-1.68911000	2.02063900	-3.68910900
H	-2.36086500	2.73952900	-2.20875500
H	-3.65629600	-4.26437000	3.09372800
C	0.58076800	-3.57345400	3.46084200
H	0.64249300	-3.05274200	4.43056000
H	-0.48206400	-3.77534800	3.25475200
H	1.10417100	-4.53836100	3.58222400
C	2.71710100	-2.58488500	2.64057500
H	3.21791100	-3.56395500	2.53241800
H	3.21437000	-1.87853600	1.95799800
H	2.89484900	-2.23234800	3.67029300
C	-0.64475500	-2.11035800	-3.50150900
H	-1.57356100	-2.18570500	-2.91229100
H	-0.76643300	-1.28058200	-4.21963900
H	-0.54392700	-3.03636000	-4.09336100
C	1.85729200	-1.93936700	-3.43830600
H	2.74653200	-1.66954700	-2.84525000
H	2.01226900	-2.96073900	-3.82880100
H	1.80809000	-1.25653500	-4.30286100

H	0.24785200	-6.12978400	-0.70651100
C	-3.77508100	3.15293400	0.85330800
H	-4.35519400	3.36839000	-0.06088700
H	-4.24005400	3.70130200	1.69130900
H	-2.75808000	3.55276600	0.69744800
H	-3.02287500	1.51486200	1.98985900

TS7-L2

C	0.45987800	0.09943600	0.12938700
N	1.23410300	-1.01400400	-0.08057600
N	1.34308600	1.15354100	0.07753600
C	0.74415500	-2.35892000	-0.11444700
C	-0.01056800	-5.00891200	-0.20818900
C	0.43587700	-2.92480400	-1.36094100
C	0.61754200	-3.06165200	1.09829300
C	0.23963000	-4.40359000	1.01987400
C	0.07004800	-4.27420100	-1.38410800
H	0.12361300	-4.98678100	1.93702800
H	-0.16571200	-4.75280900	-2.33998100
C	0.98669100	2.51633600	0.32416200
C	0.32966500	5.15151500	0.81855400
C	0.69099200	3.35096500	-0.76638900
C	0.98881400	2.97225900	1.65371100
C	0.64628900	4.30793200	1.87807700
C	0.35384600	4.67861300	-0.48918100
H	0.62390500	4.69425100	2.90178100
H	0.10217600	5.35390500	-1.31265900
C	1.26931700	2.03269800	2.80690000
H	1.84655900	1.17586500	2.41146300
C	0.65330400	2.81164700	-2.17999300
H	1.29366700	1.91013100	-2.21113200

C	0.85310800	-2.37359700	2.42946500
H	0.39674400	-1.36869400	2.33143700
C	0.48692200	-2.11554200	-2.64092400
H	0.45373000	-1.04623700	-2.35612900
C	-0.05011000	1.48013500	3.34229400
H	-0.61473400	0.98395400	2.53156300
H	-0.67786300	2.29407900	3.74921200
H	0.11996000	0.74367500	4.14749200
C	1.19573700	3.78779200	-3.21340600
H	1.25058800	3.30451300	-4.20211600
H	2.20657800	4.14247000	-2.95236700
H	0.54504100	4.67144000	-3.32774800
Ni	-1.38907200	0.32251400	0.21319000
C	6.09366200	2.32380000	-0.46226900
C	6.79495500	1.14854200	-0.64197900
C	6.11046200	-0.10088100	-0.65973000
C	4.72713600	-0.05143600	-0.49462700
C	3.99486700	1.15923300	-0.30602300
C	4.68183800	2.35213200	-0.28846200
H	7.76557400	-1.49491700	-0.94688500
H	6.63860300	3.27208600	-0.44908200
H	7.88230200	1.17009500	-0.76765300
C	6.68344800	-1.39537600	-0.81408100
C	3.88819200	-1.20602200	-0.47686600
H	4.16623400	3.30680300	-0.14253700
C	4.47081600	-2.44598200	-0.61882500
C	5.88070600	-2.51846000	-0.78968500
H	3.87157100	-3.36277200	-0.60205600
H	6.34043600	-3.50425300	-0.90432900
C	2.62019500	0.70607500	-0.16951900

C	2.55310300	-0.65825900	-0.27350200
C	-2.89480200	-2.10054200	0.29530100
C	-3.27684700	-3.25222500	0.97120700
C	-3.44871000	-3.25645100	2.35697100
C	-3.19447900	-2.07653000	3.05490600
C	-2.78819200	-0.92066400	2.39403900
C	-2.63840400	-0.89108900	0.98591300
H	-2.79092300	-2.14639800	-0.79236900
H	-3.44867900	-4.16751700	0.39439300
H	-3.29715500	-2.05129600	4.14563900
H	-2.57768900	-0.03163600	2.99646900
C	-4.03597400	-0.39411000	-3.30702600
C	-3.32924800	-0.13711500	-2.14144800
C	-3.95540400	0.40596700	-1.00372100
C	-5.33057700	0.72075500	-1.07930400
C	-6.01861500	0.46693600	-2.26863900
C	-5.39615300	-0.09185900	-3.37929000
H	-3.51593600	-0.82050000	-4.17121400
H	-2.25303100	-0.33853700	-2.08043300
C	-6.04090100	1.37089400	0.07565600
H	-7.08536300	0.71871800	-2.31057100
H	-5.96271200	-0.28238000	-4.29502000
C	-5.23793700	1.29618100	1.36029100
C	-3.77008300	1.60922800	1.09315900
H	-6.24105900	2.43062600	-0.18054300
H	-5.64587900	1.98462200	2.12045500
N	-3.21248100	0.62136100	0.16565400
H	-5.29077900	0.27691800	1.78494500
H	-7.03675200	0.91267600	0.20706200
H	0.06195000	6.19392700	1.01458100

C	2.10070900	2.66678800	3.91219500
H	3.03207800	3.10880300	3.52134800
H	1.54652600	3.45876600	4.44445400
H	2.37433600	1.90932900	4.66460500
C	-0.77037200	2.37516400	-2.51659000
H	-1.45725900	3.24047400	-2.52265500
H	-0.82370600	1.88781000	-3.50532900
H	-1.15144100	1.66216000	-1.76130400
H	-3.76967200	-4.16108900	2.88107900
C	0.16717200	-3.07139600	3.59383700
H	0.20965900	-2.43129800	4.49091900
H	-0.89170500	-3.28527200	3.38228700
H	0.67092300	-4.01996300	3.85266800
C	2.33879300	-2.21014100	2.74760200
H	2.83184500	-3.19779800	2.79643500
H	2.88339500	-1.60329200	2.00843300
H	2.46834500	-1.72617100	3.73091700
C	-0.71659700	-2.38063300	-3.53807700
H	-1.66613800	-2.32436600	-2.98047800
H	-0.75635500	-1.63946700	-4.35437500
H	-0.66449000	-3.37650400	-4.01082300
C	1.78597900	-2.36041400	-3.40343000
H	2.67391300	-2.09368600	-2.80795400
H	1.87611500	-3.42566400	-3.68120600
H	1.81338200	-1.76881400	-4.33364300
H	-0.29771200	-6.06401000	-0.24406900
H	-3.21933100	1.51133800	2.04226900
C	-3.52204200	3.02147100	0.58225300
H	-4.00266500	3.76807400	1.23804200
H	-2.43850600	3.23339400	0.55144100

H	-3.91243800	3.16441300	-0.43959300

INT8-L2

C	-0.05236500	-0.53558200	0.04512000
N	1.00380100	0.36331700	-0.07290400
N	0.08685700	-1.02546800	1.33585400
C	1.31755300	0.97721000	-1.31839800
C	1.83526200	2.09691300	-3.81845000
C	0.66004700	2.16255700	-1.68303200
C	2.23463800	0.34432900	-2.17056000
C	2.45926800	0.91032000	-3.42679700
C	0.94713100	2.71372500	-2.93037300
H	3.13763100	0.40488900	-4.12530000
H	0.44195800	3.63764000	-3.23600200
C	-0.73903200	-2.02772400	1.93360400
C	-2.14399300	-3.98944200	3.29373400
C	-1.76789800	-1.63775800	2.81128800
C	-0.45086100	-3.38051300	1.66808800
C	-1.17041100	-4.35154100	2.36865100
C	-2.44873100	-2.64913000	3.49802000
H	-0.96065600	-5.41084900	2.19150800
H	-3.24481500	-2.38067000	4.19877700
C	0.61562800	-3.76980600	0.66622300
H	0.64470100	-2.95699600	-0.08326900
C	-2.14275700	-0.18208000	3.01411500
H	-1.90782000	0.33923400	2.06494300
C	2.97419100	-0.89597000	-1.69971700
H	2.34969500	-1.34956700	-0.91128500
C	-0.32367500	2.78118900	-0.70245900
H	-0.89592900	1.92484000	-0.29534300
C	2.12417700	2.71238900	-5.15345600

H	2.91619200	3.47778300	-5.07781100
C	0.29339200	-5.05462600	-0.08436900
H	-0.73528700	-5.05083600	-0.48281700
H	0.40205400	-5.94868100	0.55437500
H	0.98909200	-5.18022000	-0.93192100
C	-1.33790200	0.47455700	4.13320700
H	-1.67106300	1.51670100	4.28114700
H	-0.25627900	0.49203500	3.93162500
H	-1.49236400	-0.06249700	5.08667200
Ni	-1.23282900	-1.07191800	-1.27625700
H	2.46802100	1.96160000	-5.88116200
H	1.23604400	3.21303000	-5.56935700
C	3.09464000	-1.97128000	-2.76329600
C	2.03784000	-2.87836700	-2.90162900
C	4.20359700	-2.10333600	-3.60263200
C	2.07881800	-3.88420000	-3.86288000
H	1.16486800	-2.76545900	-2.24572600
C	4.24548800	-3.10813900	-4.56920100
H	5.05360000	-1.42143200	-3.49004000
C	3.18496600	-4.00081000	-4.70390000
H	1.24073300	-4.58395500	-3.95382700
H	5.12186600	-3.19822900	-5.21804900
H	3.22314000	-4.79151200	-5.45881000
C	0.39680600	3.40974900	0.47191600
C	1.40541600	4.35903100	0.27534200
C	0.06445100	3.04951400	1.78073200
C	2.06347800	4.93417200	1.35973100
H	1.67637500	4.64679800	-0.74774600
C	0.72968100	3.61341300	2.86846700
H	-0.71623900	2.29562100	1.94828900

C	1.73131100	4.55819000	2.66078400
H	2.84960400	5.67599700	1.18789200
H	0.46680000	3.30426400	3.88487400
H	2.25953800	4.99653700	3.51253200
C	2.92979500	-0.90518000	5.36430400
C	3.92094400	0.03277800	5.15117300
C	3.98375900	0.74186700	3.91814000
C	3.00543500	0.43377900	2.97394700
C	1.96818900	-0.52220200	3.19079000
C	1.93612200	-1.19902400	4.39123300
H	5.72951800	2.01385500	4.24293400
H	2.90075400	-1.44288000	6.31661300
H	4.66509000	0.23431700	5.92879000
C	4.93967400	1.72927400	3.53992100
C	2.92960300	1.03380600	1.68181800
H	1.16219500	-1.94518400	4.60396300
C	3.86866000	1.97869900	1.34241200
C	4.87140800	2.31621400	2.29359000
H	3.86030500	2.46792800	0.36374000
H	5.61715300	3.06755400	2.01571500
C	1.20098500	-0.48492200	1.95420700
C	1.76825600	0.39699400	1.07469300
C	-2.97217900	-0.40821600	-2.39364400
C	-1.90497000	-0.62877300	-3.28503100
C	-1.22496000	-1.86010800	-3.28781700
C	-1.61453100	-2.85687900	-2.36867400
C	-2.67001200	-2.63587400	-1.45646400
C	-3.37198800	-1.40133000	-1.46117200
H	-3.51271300	0.54237300	-2.41744800
H	-1.61677600	0.15790200	-3.99051500

H	-1.08293600	-3.81469100	-2.34860300
H	-2.92987700	-3.41506600	-0.73318800
C	-4.13318100	2.31133500	0.60780100
C	-3.81286100	1.06208200	0.09766600
C	-4.79273400	0.06532100	-0.07329300
C	-6.11583600	0.34674500	0.33304000
C	-6.40859400	1.60913800	0.85820400
C	-5.44331800	2.59998400	0.99090800
H	-3.34304600	3.06591400	0.70627800
H	-2.77766000	0.82620000	-0.17003000
C	-7.20037600	-0.69103300	0.24021600
H	-7.44346300	1.81138000	1.15968500
H	-5.70677300	3.58400300	1.38791400
C	-6.80856100	-1.84405600	-0.66247400
C	-5.38682500	-2.30825000	-0.36713400
H	-7.43263500	-1.06898000	1.25556700
H	-7.50939300	-2.68914100	-0.55198900
N	-4.46004200	-1.19563900	-0.59568900
C	-5.22727500	-2.90986800	1.01990800
H	-5.47181700	-2.18201500	1.81135400
H	-5.89095900	-3.78122400	1.14884400
H	-4.18718500	-3.23268200	1.19493500
H	-5.12917800	-3.07894100	-1.11283800
H	-6.85060500	-1.52627600	-1.71966500
H	-8.13569100	-0.22207300	-0.11167600
H	-2.68634900	-4.76246900	3.84652300
C	1.98962400	-3.87466700	1.32102600
H	2.76061800	-4.10756200	0.56484200
H	2.28097000	-2.94270100	1.83366000
H	2.00083100	-4.68390800	2.07318200

C	-3.62864900	0.01209600	3.28296000
H	-3.91329700	-0.34546900	4.28814700
H	-3.88443400	1.08326300	3.23483800
H	-4.25451100	-0.50458000	2.53768100
C	4.30009800	-0.56604600	-1.03475700
C	4.69514900	-1.31440800	0.07946900
C	5.15131100	0.44437100	-1.49096400
C	5.90103800	-1.06129100	0.72502100
H	4.02920000	-2.09959300	0.45496600
C	6.36527500	0.69582400	-0.85186600
H	4.85618300	1.05719800	-2.34962500
C	6.74252900	-0.05243300	0.26020400
H	6.17948000	-1.64895500	1.60486100
H	7.01792900	1.49158300	-1.22415900
H	7.68872800	0.15412300	0.76891700
C	-1.37018400	3.67585900	-1.34119200
C	-2.18078000	3.12858900	-2.34515600
C	-1.63337100	4.97961900	-0.91639900
C	-3.22450800	3.85444300	-2.90419500
H	-1.98312700	2.10183100	-2.67114800
C	-2.68221300	5.71268000	-1.47681400
H	-1.02518900	5.42888400	-0.12550600
C	-3.48218600	5.15487000	-2.46781300
H	-3.84981500	3.39965400	-3.67883000
H	-2.87480000	6.73096300	-1.12575400
H	-4.30717100	5.72862900	-2.89977200
H	-0.39360100	-2.03357000	-3.97699400

INT1-L4

C	-0.03230800	0.01317500	0.14449400
N	0.56583300	-0.09378800	-1.12522200

N	1.07595500	-0.02962700	1.01186500
C	-0.24013600	-0.33091600	-2.26855400
C	-1.89260900	-0.84066400	-4.45851500
C	-0.90593900	0.74414400	-2.88526200
C	-0.44665100	-1.65657500	-2.67563700
C	-1.28069800	-1.88924600	-3.77286800
C	-1.70844600	0.46530600	-3.98855000
H	-1.50311100	-2.92360900	-4.06483300
H	-2.25765500	1.28345200	-4.46873200
C	0.90475100	0.31823300	2.37532900
C	0.45639800	1.06636700	5.02468400
C	1.13302500	1.64659000	2.75829500
C	0.38848100	-0.63489800	3.27480700
C	0.19657000	-0.24229000	4.59625700
C	0.90122600	2.00032100	4.09137000
H	-0.21981700	-0.96029300	5.31248200
H	1.02189400	3.04835500	4.39444000
C	-0.05452900	-1.98310300	2.73405100
H	-0.55480700	-1.72281300	1.77287400
C	1.52143900	2.67398800	1.70915200
H	1.30347100	2.21250300	0.73158900
C	0.17681500	1.45568500	6.44452300
H	0.53437000	2.47227300	6.66861000
C	0.14554900	-2.79595600	-1.86357800
H	0.45921300	-2.35851800	-0.90038000
C	-0.81255600	2.12872000	-2.26468800
H	-0.83294600	1.93047900	-1.17022700
Ni	-1.75247800	0.14867700	0.53764000
H	0.65327600	0.76138600	7.15662000
H	-0.90659100	1.43051000	6.65445500

C	-0.92311900	-3.80131400	-1.46753200
C	-1.98197000	-3.32868600	-0.67612300
C	-0.87874900	-5.15602900	-1.79658600
C	-2.97753700	-4.19170100	-0.23601200
H	-2.00188100	-2.26801900	-0.37208100
C	-1.87677800	-6.02502900	-1.34628700
H	-0.04877900	-5.54362000	-2.39750500
C	-2.92786500	-5.54724500	-0.56944900
H	-3.79322700	-3.79487300	0.37755900
H	-1.82668400	-7.08718100	-1.60769000
H	-3.70848000	-6.22988000	-0.21944400
C	0.47408600	2.87107700	-2.55185900
C	1.41994200	2.43272500	-3.48133100
C	0.73928400	4.05504800	-1.84488700
C	2.60081700	3.14645500	-3.69575700
H	1.23078600	1.51346400	-4.04635700
C	1.91022800	4.77206400	-2.06196600
H	0.00724200	4.41246500	-1.11003100
C	2.85041900	4.31951200	-2.99016800
H	3.33202700	2.77416200	-4.42083500
H	2.09771600	5.68504000	-1.48713500
H	3.77706900	4.87831900	-3.15529400
C	0.59862000	3.88093700	1.74025800
C	1.02443300	5.18033700	2.01504500
C	-0.74799600	3.66311700	1.40989100
C	0.12181900	6.24615100	1.96116500
H	2.07655200	5.36880100	2.25500700
C	-1.64451500	4.72356000	1.35681600
H	-1.08562900	2.64100000	1.16090800
C	-1.21168500	6.02285000	1.63243600

H	0.47163900	7.26180200	2.17311400
H	-2.68754400	4.53131300	1.08153800
H	-1.91487900	6.85997100	1.58282600
C	1.05834100	-2.95177800	2.40063500
C	2.38584800	-2.76716100	2.79201200
C	0.73487600	-4.10440800	1.66699800
C	3.36709100	-3.70403200	2.45956200
H	2.65701700	-1.87434700	3.36655300
C	1.70751700	-5.04246800	1.34274300
H	-0.30360800	-4.25939800	1.34678000
C	3.03224600	-4.84679000	1.73940800
H	4.40377700	-3.53209800	2.76739800
H	1.42918700	-5.92444200	0.75644300
H	3.80137500	-5.57989900	1.47638300
C	5.99683800	-0.34190000	1.20314200
C	6.46402200	-0.47117900	-0.08866600
C	5.54586700	-0.48825300	-1.17979500
C	4.19701400	-0.36529200	-0.85827900
C	3.70076900	-0.23952100	0.47617500
C	4.61203900	-0.22599500	1.50879200
H	6.90525200	-0.72296900	-2.87494400
H	6.71356800	-0.32091600	2.03034000
H	7.53842200	-0.55834300	-0.28383700
C	5.85978300	-0.61858700	-2.56481500
C	3.14383400	-0.35313700	-1.82322600
H	4.29386200	-0.11241900	2.55034200
C	3.47716800	-0.48626500	-3.15234400
C	4.84940700	-0.61823500	-3.50442000
H	2.71089900	-0.50831900	-3.93413400
H	5.10468600	-0.73035400	-4.56296300

C	2.25884200	-0.14624600	0.30800900
C	1.93672700	-0.20680500	-1.02426400
C	-2.76786600	-1.09865900	-5.64709900
H	-2.22606900	-0.92608500	-6.59408500
H	-3.13340500	-2.13755500	-5.66342400
H	-3.64297900	-0.42873600	-5.65122900
C	-1.14041600	-2.63230500	3.56637900
C	-0.84694900	-3.46962500	4.64763200
C	-2.48008300	-2.38198200	3.24834000
C	-1.86962600	-4.03666200	5.40509300
H	0.20106100	-3.68267200	4.89114400
C	-3.50289600	-2.95350700	4.00252900
H	-2.70525300	-1.72609400	2.39244100
C	-3.20193300	-3.77996800	5.08393300
H	-1.62447800	-4.68993100	6.24887900
H	-4.54545700	-2.75170400	3.73568300
H	-4.00646400	-4.23032000	5.67412100
C	3.00137800	2.99546400	1.67520800
C	3.75402500	3.23821400	2.82864000
C	3.65121000	3.04578000	0.43654900
C	5.11495900	3.53047600	2.74496000
H	3.27218800	3.18541600	3.81058800
C	5.00959700	3.33632800	0.34842400
H	3.07944900	2.83738600	-0.47559900
C	5.74823400	3.58033800	1.50467700
H	5.68632800	3.71479900	3.66049700
H	5.49319100	3.35415700	-0.63379900
H	6.81851700	3.80010200	1.44049500
C	-2.05639300	2.95265900	-2.52745800
C	-2.15858100	3.84694500	-3.59671800

C	-3.16686900	2.75738500	-1.69825400
C	-3.35421100	4.52179300	-3.84175000
H	-1.29046100	4.01339200	-4.24585300
C	-4.36459400	3.42120000	-1.94897700
H	-3.07767700	2.06601600	-0.84725800
C	-4.46164300	4.30602600	-3.02253900
H	-3.42214100	5.21923900	-4.68326500
H	-5.22696800	3.22879600	-1.30082600
H	-5.40271900	4.82834800	-3.22276500
C	1.40618600	-3.38232900	-2.46607000
C	1.50560900	-3.74061300	-3.81489600
C	2.52856700	-3.56349400	-1.64991900
C	2.68878800	-4.26822700	-4.32949400
H	0.64704500	-3.59445600	-4.47840100
C	3.71282700	-4.08933500	-2.15931900
H	2.47388700	-3.27368200	-0.59428700
C	3.79818200	-4.44366300	-3.50397200
H	2.74477800	-4.53959900	-5.38867700
H	4.57779100	-4.20493800	-1.49769800
H	4.72990700	-4.84904200	-3.91086100
C	-5.00406000	-0.70974300	-2.37843600
C	-4.11858100	-0.45702500	-1.34585700
C	-4.53938700	0.04010800	-0.08192200
C	-5.93571400	0.30350100	0.05719500
C	-6.80677100	0.04577200	-1.00244100
C	-6.37325700	-0.46834900	-2.22233700
H	-4.61138700	-1.10000800	-3.32557100
H	-3.04320200	-0.62297200	-1.48275500
C	-6.45147200	0.91385700	1.33302400
H	-7.87218000	0.27289300	-0.85637000

H	-7.08069000	-0.66433200	-3.03399900
C	-5.32476600	1.59399700	2.08336000
C	-4.13877500	0.64320000	2.20058900
H	-7.27250900	1.62083600	1.10890300
H	-4.98253300	2.49479900	1.53262800
N	-3.60969300	0.27776800	0.89698100
C	-3.06363000	1.23169300	3.09135000
H	-2.73944100	2.21488600	2.70568700
H	-3.43683200	1.37357400	4.12205000
H	-2.17021400	0.58568200	3.12734700
H	-4.52939100	-0.27404200	2.71493600
H	-5.65100800	1.92944700	3.08503700
H	-6.89843200	0.13653400	1.98685700

INT2-L4

C	-0.05915100	-0.18797100	-0.43070000
N	0.27180500	-0.53300800	0.86434500
N	-0.47292500	1.12408100	-0.34070800
C	0.53757800	-1.89825500	1.18997200
C	0.94318900	-4.63515400	1.49501500
C	1.83255600	-2.41579900	1.00536200
C	-0.54440100	-2.70816000	1.55542800
C	-0.31820100	-4.08067100	1.69904700
C	2.00736100	-3.78581200	1.16710100
H	-1.16471500	-4.73829300	1.93245100
H	2.99293800	-4.22687100	0.98650200
C	-0.71055700	1.91715900	-1.50546900
C	-1.06080200	3.39953100	-3.84598900
C	0.33792200	2.72256800	-1.97126400
C	-1.93439300	1.81496600	-2.19823900
C	-2.08264000	2.57832600	-3.35453500

C	0.14527400	3.44360800	-3.15354600
H	-3.01682700	2.51243000	-3.92190700
H	0.97881300	4.02749500	-3.56250900
C	-3.00443200	0.83161000	-1.74407600
H	-2.46036100	-0.13015300	-1.61119300
C	1.66931300	2.77650300	-1.24214800
H	1.68972300	1.91910400	-0.54597200
C	-1.27097300	4.19817500	-5.09502800
H	-0.31897000	4.55254100	-5.51787400
C	-1.92184700	-2.10532300	1.76351800
H	-1.93998200	-1.14308700	1.22177400
C	2.95708800	-1.46485900	0.63858000
H	2.53119000	-0.76865700	-0.10709700
Ni	-0.13781800	-1.30311100	-1.92929400
H	-1.89561000	5.08674900	-4.89911900
H	-1.79161200	3.60927400	-5.86665300
C	-3.00784800	-2.92002800	1.08526800
C	-2.92630400	-3.04692900	-0.30916400
C	-4.09702200	-3.48071400	1.74980400
C	-3.90907500	-3.72313300	-1.02088200
H	-2.08199500	-2.56185700	-0.82422800
C	-5.08704100	-4.15988000	1.03381500
H	-4.18645100	-3.37514600	2.83599600
C	-4.99731700	-4.28488500	-0.34832700
H	-3.83912100	-3.80489100	-2.11112400
H	-5.93914400	-4.59145900	1.56761000
H	-5.77605700	-4.81322700	-0.90580500
C	3.41027500	-0.61530700	1.81380800
C	3.38937200	-1.08132400	3.13090500
C	3.93474600	0.65769800	1.56319500

C	3.87515400	-0.29255200	4.17328400
H	2.97757500	-2.07477300	3.34522000
C	4.41371700	1.44970500	2.60325100
H	3.97275200	1.02611000	0.52799800
C	4.38294800	0.97749000	3.91495400
H	3.84617500	-0.67243500	5.19962700
H	4.81420600	2.44522500	2.38535200
H	4.75744900	1.59905500	4.73332300
C	2.82663100	2.51650800	-2.19356200
C	3.88944700	3.40032100	-2.38054400
C	2.82840900	1.29917500	-2.89037800
C	4.93061600	3.08036700	-3.25535000
H	3.91211200	4.34848200	-1.83378000
C	3.86512900	0.98274600	-3.75987300
H	1.99734600	0.59351800	-2.73640500
C	4.92268600	1.87450500	-3.94788400
H	5.75732600	3.78421600	-3.39032000
H	3.84974200	0.02624100	-4.29488700
H	5.73982800	1.62505100	-4.63084300
C	-3.67473200	1.16349000	-0.42432100
C	-3.65722400	2.43859800	0.14125700
C	-4.42764500	0.16247500	0.20660100
C	-4.36275000	2.70929900	1.31595700
H	-3.09025400	3.23776600	-0.34915600
C	-5.14116200	0.43182800	1.36853900
H	-4.46359600	-0.84109800	-0.23567900
C	-5.11021400	1.70964800	1.93052900
H	-4.32617900	3.71415400	1.74857700
H	-5.71451700	-0.36887600	1.84665500
H	-5.66605200	1.92133800	2.84895900

C	-1.03820600	4.89410200	2.79924400
C	-0.69758700	4.44343700	4.05956800
C	-0.27459200	3.09756100	4.24715000
C	-0.23034400	2.29467700	3.10646200
C	-0.59543100	2.74725200	1.80395500
C	-0.99529700	4.05626000	1.65337000
H	0.09459300	3.06422600	6.39818100
H	-1.34687600	5.93575400	2.66946700
H	-0.74554400	5.11971100	4.91945500
C	0.11199100	2.48092300	5.47159700
C	0.18702900	0.93108700	3.11616200
H	-1.25839700	4.46732800	0.67342200
C	0.54267200	0.35883600	4.31510800
C	0.50085600	1.15690800	5.49012500
H	0.84122200	-0.69138500	4.38028900
H	0.78454400	0.69626900	6.44127600
C	-0.38677900	1.57649600	0.96289100
C	0.07351400	0.52809700	1.72148900
C	0.06040300	-3.49445700	-2.50691600
C	-0.79974000	-2.60940800	-3.21080600
C	-0.21642900	-1.57982600	-3.99596300
C	1.17363700	-1.58783200	-4.23085200
C	1.98712300	-2.58418600	-3.69275900
C	1.42828100	-3.50629600	-2.80135200
H	-0.35126200	-4.26242300	-1.84382100
H	-1.88414300	-2.77026600	-3.18426700
H	1.61113400	-0.82772700	-4.88674400
H	3.05286600	-2.63867600	-3.92909900
Cl	2.45842300	-4.71097100	-2.07138400
C	1.17209400	-6.11245000	1.56743700

H	2.00995000	-6.36084800	2.23919500
H	0.28018500	-6.65259100	1.91788600
H	1.43707900	-6.50644000	0.57093200
H	-0.84746700	-0.87596500	-4.54954400
C	-4.05790700	0.58157200	-2.80806900
C	-5.19505500	1.38954300	-2.91673500
C	-3.88964200	-0.45519000	-3.72885800
C	-6.12847600	1.17360900	-3.92726000
H	-5.35013500	2.19993000	-2.19566300
C	-4.82373300	-0.67907400	-4.73834000
H	-3.00431000	-1.09372700	-3.64764600
C	-5.94653300	0.13807800	-4.84261800
H	-7.01041800	1.81736600	-3.99706000
H	-4.67275700	-1.49947000	-5.44626200
H	-6.68308100	-0.03482200	-5.63264700
C	1.82918900	4.01021100	-0.37248700
C	1.52335800	5.30103600	-0.81608800
C	2.31345600	3.85739900	0.93028600
C	1.70632300	6.40368300	0.01749400
H	1.13015200	5.45326600	-1.82599300
C	2.49679700	4.95455400	1.76707400
H	2.52873000	2.85072200	1.30400600
C	2.19504700	6.23583900	1.31166400
H	1.46209000	7.40496400	-0.35021800
H	2.86364900	4.79976700	2.78709200
H	2.33308800	7.10207800	1.96537900
C	4.15721800	-2.09903300	-0.03522000
C	5.08454200	-2.87466300	0.67023600
C	4.37581700	-1.87344500	-1.39508500
C	6.17572500	-3.44398700	0.02012000

H	4.95699300	-3.02523700	1.74827900
C	5.47751600	-2.42496000	-2.04524600
H	3.66170700	-1.25410400	-1.94975300
C	6.37532000	-3.22261100	-1.34222600
H	6.88600900	-4.05487300	0.58534700
H	5.63560200	-2.22904200	-3.11118800
H	7.23855900	-3.66227700	-1.85023700
C	-2.18827000	-1.75349800	3.21460200
C	-1.95567600	-2.65173000	4.26155300
C	-2.68398000	-0.48348400	3.52664200
C	-2.22413500	-2.29171800	5.58117400
H	-1.55289000	-3.64685300	4.04435000
C	-2.95622200	-0.12047400	4.84279600
H	-2.84738900	0.23994200	2.72020000
C	-2.72749800	-1.02546300	5.87656200
H	-2.03676300	-3.00840000	6.38663000
H	-3.33485500	0.88430800	5.05744900
H	-2.93437800	-0.74384700	6.91333800

TS3-L4

C	0.28888400	0.21682400	0.29359100
N	-0.06112400	-1.05323600	-0.13363500
N	-0.01534200	1.02322200	-0.78214200
C	0.11233400	-2.19416200	0.70241000
C	0.51944200	-4.31837000	2.45706100
C	-0.90210400	-2.52476400	1.61367300
C	1.30018000	-2.93313200	0.60769000
C	1.48689600	-3.98697600	1.50438700
C	-0.67286700	-3.58717200	2.48703900
H	2.42427900	-4.55575000	1.46759400
H	-1.44386100	-3.84612500	3.22333800

C	-0.06475200	2.45084000	-0.84207500
C	-0.25888200	5.18165500	-1.39077600
C	-1.32898900	3.06483900	-0.83070800
C	1.11191800	3.19856900	-0.99607100
C	0.98714100	4.55901100	-1.28186200
C	-1.40232000	4.42630200	-1.12419400
H	1.89543100	5.15922800	-1.40669600
H	-2.38057800	4.91913300	-1.13170500
C	2.45911700	2.54856800	-0.74046000
H	2.29082700	1.89027400	0.13550000
C	-2.54818800	2.29943900	-0.34603100
H	-2.36378200	1.23038000	-0.55594300
C	2.31181600	-2.59790500	-0.47369900
H	2.21561100	-1.51335000	-0.66835000
C	-2.22312800	-1.77473800	1.57495800
H	-1.98490200	-0.72560500	1.31582300
C	0.76302600	-5.41576400	3.44703100
H	-0.16975100	-5.93467300	3.71693900
Ni	1.14363400	0.58100800	1.93293100
H	1.47542400	-6.16165200	3.06314900
H	1.18922300	-5.01502900	4.38348400
C	3.74850000	-2.77683200	-0.01993700
C	4.20359200	-1.92075900	0.99041300
C	4.64267600	-3.69280300	-0.57426900
C	5.51774400	-1.96973700	1.43635000
H	3.50623000	-1.18638800	1.40913200
C	5.96437400	-3.74922500	-0.12339000
H	4.31558500	-4.36035500	-1.37769800
C	6.40707300	-2.89018400	0.87769300
H	5.84589800	-1.27761000	2.21820600

H	6.65521600	-4.47129700	-0.56929400
H	7.44483100	-2.93325100	1.22109100
C	-3.11659400	-2.30659000	0.46912700
C	-3.30986600	-3.67854200	0.27494400
C	-3.79884900	-1.41863500	-0.36757900
C	-4.16631900	-4.14498100	-0.72039100
H	-2.77967100	-4.39325800	0.91468800
C	-4.64670200	-1.87893300	-1.37233600
H	-3.65827400	-0.34079000	-0.23028800
C	-4.83539800	-3.24709100	-1.55053700
H	-4.30743700	-5.22226700	-0.85234700
H	-5.15851100	-1.15865800	-2.01906200
H	-5.49834500	-3.61430300	-2.33943300
C	-1.87652600	1.07334100	-5.32933600
C	-2.25835400	-0.21731400	-5.64152100
C	-2.03401300	-1.27714100	-4.71739200
C	-1.42691400	-0.92951700	-3.51055000
C	-1.03809600	0.40256700	-3.17742600
C	-1.25905300	1.40758000	-4.09392700
H	-2.84650200	-2.98249400	-5.81291600
H	-2.05890100	1.87282800	-6.05333600
H	-2.73576500	-0.43237300	-6.60327800
C	-2.36948100	-2.65191600	-4.88440600
C	-1.14909400	-1.85985100	-2.46444500
H	-0.99128100	2.44760400	-3.87786800
C	-1.48379200	-3.17952200	-2.65415300
C	-2.09523100	-3.55787100	-3.88090300
H	-1.28863200	-3.93540100	-1.88748300
H	-2.35781300	-4.61064700	-4.02441000
C	-0.50399100	0.27098000	-1.83271700

C	-0.55226600	-1.03310500	-1.42172800
C	1.38329000	1.77936900	4.49560800
C	0.66466600	1.43815200	5.63053200
C	0.41146200	0.09235600	5.93990300
C	0.84200000	-0.91682000	5.08593400
C	1.55888300	-0.60821900	3.92383100
C	1.81416200	0.75001600	3.63477500
H	1.61831900	2.82110800	4.25363100
H	0.30338100	2.22784600	6.29764900
H	0.63047500	-1.96494100	5.32503400
H	1.99734400	-1.40785000	3.31654100
Cl	3.35393500	1.15541700	2.52878900
C	1.98309400	-3.28468000	-1.78499000
C	1.96670700	-2.53672900	-2.96588300
C	1.71207200	-4.65492400	-1.85949600
C	1.70388500	-3.13868800	-4.19349300
H	2.14959700	-1.45800400	-2.91737800
C	1.44809500	-5.26173100	-3.08630700
H	1.70639300	-5.25562000	-0.94263600
C	1.44492500	-4.50560600	-4.25809900
H	1.68790000	-2.52686100	-5.10159400
H	1.24167800	-6.33571900	-3.12685100
H	1.23179100	-4.98212800	-5.21965200
C	-2.90383200	-1.70105000	2.92870500
C	-4.11653100	-2.32923900	3.21834100
C	-2.28235500	-0.94128800	3.92711400
C	-4.69064400	-2.21514500	4.48596400
H	-4.62836500	-2.91060100	2.44521100
C	-2.84995100	-0.83495500	5.19157500
H	-1.34103300	-0.42443100	3.69313200

C	-4.05786000	-1.47207100	5.47762200
H	-5.64187400	-2.71380400	4.69529600
H	-2.34713800	-0.23374100	5.95569500
H	-4.50608500	-1.38399100	6.47181700
H	-0.12035600	-0.16278700	6.86130500
C	2.97605500	1.64720800	-1.83597800
C	2.61465200	1.78336900	-3.17684000
C	3.92361800	0.67441800	-1.48786400
C	3.20364700	0.97761300	-4.15438800
H	1.87289700	2.53784300	-3.46347400
C	4.51884300	-0.12282800	-2.45908100
H	4.20454000	0.56080200	-0.43236600
C	4.16344800	0.03258500	-3.80071600
H	2.91123500	1.09710600	-5.20224800
H	5.25372100	-0.87911400	-2.16167800
H	4.62407400	-0.59771100	-4.56753300
C	3.48445700	3.57458700	-0.29518900
C	4.53608000	3.99960200	-1.10871400
C	3.33718000	4.15615100	0.97072000
C	5.41843400	4.98803900	-0.66955800
H	4.67188400	3.55130000	-2.09874200
C	4.21434200	5.14002600	1.41034000
H	2.51503300	3.81592200	1.61269800
C	5.26062100	5.56136100	0.58822700
H	6.23810600	5.30895900	-1.31944100
H	4.08661800	5.57914300	2.40427900
H	5.95467100	6.33352400	0.93296200
C	-3.81741100	2.64525900	-1.09952900
C	-4.63185200	3.72176600	-0.73077800
C	-4.18753800	1.88126200	-2.21188200

C	-5.76981300	4.03942700	-1.46876900
H	-4.37668400	4.30905200	0.15877900
C	-5.32570300	2.19571400	-2.95145700
H	-3.57296500	1.02072000	-2.50373800
C	-6.11895700	3.28015900	-2.58441800
H	-6.39422700	4.88487000	-1.16478600
H	-5.59226500	1.58347700	-3.81886000
H	-7.01486000	3.52880900	-3.16068300
C	-2.69884000	2.39752100	1.16986000
C	-1.77210400	3.04983100	1.98819900
C	-3.79221100	1.76471000	1.78103900
C	-1.94218400	3.08101500	3.37345600
H	-0.89907100	3.54428700	1.54949000
C	-3.95900500	1.79039900	3.16053300
H	-4.53446300	1.24910300	1.16023700
C	-3.03356800	2.45622900	3.96439400
H	-1.20224800	3.59685500	3.99295700
H	-4.81147200	1.27450200	3.61387700
H	-3.16055500	2.47452800	5.05134600
C	-0.35986300	6.62896600	-1.76049600
H	0.50031100	7.20394400	-1.38443800
H	-0.37809900	6.75799800	-2.85662700
H	-1.28021800	7.08650900	-1.36686700

INT4-L4

C	-0.01744800	-0.04471500	-0.43021700
N	0.37081300	-0.54685600	0.78497500
N	-0.65317700	1.12661900	-0.14042000
C	0.96268600	-1.84252200	0.88339500
C	2.07310300	-4.39180600	0.95447400
C	2.35433600	-1.97831200	0.73395400

C	0.11567200	-2.94341400	1.08444400
C	0.69412000	-4.21329000	1.08841600
C	2.88523800	-3.26609000	0.78546300
H	0.04518300	-5.09350500	1.17204200
H	3.96530900	-3.40065900	0.65672700
C	-1.16114000	2.09043300	-1.07433200
C	-2.14312300	4.09629600	-2.73506900
C	-0.35882900	3.21023900	-1.34012800
C	-2.43798400	1.92391200	-1.64589800
C	-2.89467800	2.94556600	-2.47692700
C	-0.87864400	4.21117400	-2.16287400
H	-3.87450700	2.84463700	-2.95416000
H	-0.27359100	5.09901300	-2.37455400
C	-3.26294800	0.66395800	-1.41114300
H	-2.57288300	-0.18392600	-1.58755000
C	1.07687000	3.26831200	-0.84276500
H	1.11330500	2.69974400	0.10438000
C	-1.37345900	-2.74125000	1.30161900
H	-1.60882100	-1.70476300	1.00546800
C	3.22709800	-0.73955600	0.58664700
H	2.68816900	-0.04353300	-0.08708200
Ni	0.50702500	-1.10399900	-2.02935000
C	-2.21370600	-3.57875700	0.35342500
C	-2.04404600	-3.35201000	-1.02045800
C	-3.19347100	-4.48063900	0.76631300
C	-2.82676800	-4.01024000	-1.95966200
H	-1.29319000	-2.62537400	-1.36402400
C	-3.98401400	-5.14344000	-0.17668700
H	-3.35835900	-4.65680200	1.83434400
C	-3.80542100	-4.91244600	-1.53712900

H	-2.66900400	-3.80542800	-3.02313600
H	-4.75240900	-5.84484100	0.16228500
H	-4.43037600	-5.43079500	-2.26992900
C	3.35855600	-0.03544100	1.92526100
C	3.77602500	-0.72909900	3.06683000
C	3.06827800	1.32618400	2.04691000
C	3.90967100	-0.07569800	4.28952700
H	4.00341400	-1.79904900	2.99291300
C	3.18974700	1.98275100	3.27046600
H	2.73530300	1.87975500	1.16225900
C	3.61390200	1.28294000	4.39658200
H	4.24320300	-0.63454200	5.16937500
H	2.94605200	3.04771700	3.33946500
H	3.70696200	1.79281200	5.35993400
C	-1.77621200	4.18994200	3.55937600
C	-1.38318100	3.58411100	4.73754200
C	-0.76104700	2.30460800	4.71379700
C	-0.58631900	1.72293800	3.45714200
C	-1.01409500	2.32888900	2.23774500
C	-1.60118000	3.57448100	2.29077200
H	-0.40509000	1.95966400	6.83958300
H	-2.24251400	5.17864900	3.59801000
H	-1.54178500	4.08989700	5.69559900
C	-0.29101900	1.55300400	5.82938500
C	0.03972100	0.45809200	3.25426100
H	-1.92346900	4.09532200	1.38239100
C	0.47404400	-0.25087500	4.34903200
C	0.29831400	0.31960400	5.63903300
H	0.93431500	-1.23890200	4.24724000
H	0.64370600	-0.24721900	6.50870400

C	-0.64395600	1.35091700	1.22465200
C	-0.01014000	0.28659600	1.81034800
C	2.05380300	-2.15405200	-4.30490600
C	2.94739200	-3.12795200	-4.74642300
C	3.10417200	-4.31780000	-4.03073700
C	2.35058600	-4.53495000	-2.88029000
C	1.45382100	-3.55704900	-2.43891300
C	1.32781500	-2.34224800	-3.12355000
H	1.92204300	-1.23293400	-4.88085100
H	3.52367300	-2.96313500	-5.66327900
H	2.44325900	-5.47771800	-2.32983400
H	0.85770900	-3.76215500	-1.53805400
Cl	-0.73737000	-0.43806800	-3.64216700
C	-1.73091700	-2.81508700	2.77566100
C	-2.35867800	-1.71906500	3.37495800
C	-1.42750900	-3.92162100	3.57850100
C	-2.68537100	-1.72307600	4.72886700
H	-2.58767500	-0.83619900	2.76934900
C	-1.74663500	-3.92894900	4.93435800
H	-0.92972500	-4.79343900	3.14242200
C	-2.37672700	-2.82870200	5.51584200
H	-3.16854600	-0.84343600	5.16743500
H	-1.50013000	-4.80405800	5.54311800
H	-2.62392100	-2.83617200	6.58152900
C	4.55064500	-1.01400000	-0.10819100
C	5.79177000	-0.74358900	0.47092900
C	4.51913000	-1.51833600	-1.41573300
C	6.97348100	-0.98328200	-0.23424400
H	5.84559200	-0.33150400	1.48296400
C	5.69362400	-1.76484500	-2.11584200

H	3.55377100	-1.71520700	-1.89807500
C	6.93007100	-1.49718300	-1.52605600
H	7.93608400	-0.76298500	0.23702900
H	5.63432100	-2.16179600	-3.13434400
H	7.85677800	-1.68720300	-2.07555500
H	3.80094300	-5.08407200	-4.38438700
C	-3.82821800	0.50879500	-0.01034300
C	-3.99252600	1.57627800	0.87217600
C	-4.32197400	-0.74999200	0.36462100
C	-4.63099800	1.39777100	2.10230300
H	-3.63416200	2.57148500	0.58741400
C	-4.97710700	-0.92610400	1.57725700
H	-4.20931700	-1.59694800	-0.32519800
C	-5.13639800	0.15027400	2.45300300
H	-4.73929900	2.24921200	2.78234000
H	-5.35496300	-1.91732500	1.84788700
H	-5.64763600	0.00984200	3.41027100
C	-4.39893800	0.50100800	-2.41265000
C	-5.63315500	1.13123300	-2.21005100
C	-4.22798800	-0.28198300	-3.55724400
C	-6.66379000	0.99768200	-3.13556500
H	-5.79006300	1.73407500	-1.30816400
C	-5.26286600	-0.42274300	-4.48116600
H	-3.26556600	-0.77662700	-3.72417000
C	-6.48171300	0.21812900	-4.27718800
H	-7.62000100	1.49989900	-2.95990300
H	-5.11092700	-1.04243300	-5.37006900
H	-7.29288500	0.10640100	-5.00287600
C	1.53441300	4.66686900	-0.48032200
C	2.09243500	5.53090500	-1.42861300

C	1.39350200	5.12016800	0.83544200
C	2.47818200	6.82128300	-1.07464400
H	2.23788000	5.17805700	-2.45601100
C	1.78130800	6.40991800	1.19326500
H	0.97472500	4.44483800	1.59274900
C	2.32137000	7.26652900	0.23701600
H	2.91284800	7.48355600	-1.82925700
H	1.66516400	6.74413000	2.22864200
H	2.62985400	8.27848400	0.51509300
C	2.03655300	2.56137300	-1.79216400
C	1.61978100	1.98906200	-2.99401600
C	3.38980600	2.45235600	-1.43393000
C	2.53104600	1.32738900	-3.81818700
H	0.57003700	2.02911200	-3.29814900
C	4.29476300	1.77915500	-2.24703600
H	3.74196400	2.91884600	-0.50605900
C	3.86550100	1.21290900	-3.44894700
H	2.17170500	0.89476900	-4.75632900
H	5.34265200	1.69304000	-1.94195200
H	4.57349700	0.67680600	-4.08887800
C	2.67140800	-5.76353500	0.99957900
H	2.88400200	-6.06917700	2.03873700
H	1.98833300	-6.51668700	0.57695800
H	3.62216500	-5.80929400	0.44722200
C	-2.68869500	5.15859700	-3.63804400
H	-2.76095100	4.79432900	-4.67668200
H	-3.70608600	5.45733900	-3.33779300
H	-2.05528300	6.05788800	-3.64265700

INT5-L4

C	0.16985700	0.01113300	-0.06346200

N	-0.70581800	-0.38767400	0.93254300
N	-0.64296000	0.51976600	-1.06153200
C	-0.31505400	-1.14576600	2.09404000
C	0.67415000	-2.69278300	4.21398900
C	0.36038100	-0.50475000	3.15920800
C	-0.58400100	-2.53145200	2.13166800
C	-0.06978400	-3.27782900	3.19550100
C	0.85387000	-1.30853200	4.18795000
H	-0.23497800	-4.36205300	3.20364700
H	1.42498500	-0.84142400	4.99555300
C	-0.29034000	1.28050200	-2.23464100
C	0.00658000	2.69765400	-4.64300300
C	-0.49888000	2.67757300	-2.24721200
C	0.10351400	0.60054800	-3.40329300
C	0.21835500	1.32078800	-4.59151500
C	-0.32751600	3.35118800	-3.46066800
H	0.49969000	0.79031600	-5.50778800
H	-0.44927600	4.44023600	-3.47602100
C	0.37806800	-0.89000500	-3.36566000
H	0.92783800	-1.06550400	-2.41879400
C	-0.94441500	3.47302100	-1.02849300
H	-0.64274900	2.88661600	-0.13915600
C	-1.45772700	-3.24077400	1.10684600
H	-1.41285700	-2.65785200	0.16675600
C	0.57202300	1.00078700	3.16957600
H	1.04385200	1.26456200	2.20507600
C	1.29633800	-3.51820600	5.29698300
H	0.81336300	-4.50303700	5.39021700
Ni	2.07609900	0.05298200	-0.11606400
H	2.36503600	-3.69612700	5.08525900

H	1.24653900	-3.01149000	6.27390900
C	-0.97750600	-4.64539900	0.73506300
C	0.26850300	-4.79301700	0.11549700
C	-1.76071600	-5.79179900	0.92328300
C	0.70402200	-6.03748900	-0.33022400
H	0.90681200	-3.91375900	-0.02337600
C	-1.31911900	-7.04148400	0.48778000
H	-2.74139600	-5.71893400	1.40199200
C	-0.08960200	-7.16905200	-0.15005400
H	1.67967100	-6.10859100	-0.81944300
H	-1.95286800	-7.92016200	0.64515900
H	0.25297400	-8.14676100	-0.50302300
C	-0.69517100	1.83038000	3.26630300
C	-1.84659600	1.39339300	3.92287200
C	-0.64482300	3.15506400	2.80521800
C	-2.92221500	2.25983700	4.12988800
H	-1.89271300	0.36197700	4.29472400
C	-1.70714300	4.02348500	3.02865900
H	0.25334500	3.49168000	2.27103800
C	-2.85155900	3.58010800	3.69518600
H	-3.82006200	1.89716600	4.64218600
H	-1.64739900	5.05298000	2.65932100
H	-3.69045800	4.26259500	3.86437800
C	-5.31463500	0.94737200	-2.52087900
C	-6.12433500	0.62830100	-1.45014900
C	-5.54801000	0.25062900	-0.20447200
C	-4.15532900	0.18318200	-0.15859200
C	-3.31351200	0.53705200	-1.25193100
C	-3.89598800	0.92666000	-2.43560100
H	-7.33098400	0.02135900	1.03430200

H	-5.77435800	1.25292300	-3.46598700
H	-7.21431400	0.68115900	-1.54612500
C	-6.23666600	-0.02170300	1.00990900
C	-3.41025000	-0.18387300	1.00172000
H	-3.29034900	1.23647000	-3.29370200
C	-4.10537800	-0.40756200	2.16834900
C	-5.52292800	-0.31991300	2.15328000
H	-3.59192500	-0.66868000	3.09707100
H	-6.06190800	-0.51852900	3.08512800
C	-1.97187400	0.37115800	-0.71523500
C	-2.01926400	-0.13223500	0.55428200
C	2.71507900	-2.42675700	1.09060700
C	3.32337900	-3.67696000	1.19760800
C	3.68603100	-4.38764400	0.05456200
C	3.39716100	-3.83606100	-1.19187300
C	2.78848900	-2.58129300	-1.28622800
C	2.46079800	-1.81535900	-0.15220400
H	2.46447900	-1.90266200	2.01967000
H	3.52394300	-4.09450300	2.19152100
H	3.65606000	-4.37765400	-2.10989700
H	2.62371200	-2.18769100	-2.29643800
C	5.46137100	0.13635800	2.96842000
C	4.53151700	0.13193400	1.94164400
C	4.90651700	0.22510200	0.57341000
C	6.31214900	0.32813600	0.31293900
C	7.22290500	0.32251700	1.36425000
C	6.82998200	0.23215100	2.70104600
H	5.10375300	0.06499900	4.00347600
H	3.46369300	0.06686700	2.17106100
C	6.76885300	0.41703900	-1.11477300

H	8.29125400	0.39063900	1.11478900
H	7.56917900	0.22977300	3.50804700
C	5.76913500	-0.29018500	-2.01061300
C	4.36491400	0.26410800	-1.78290900
H	6.86226000	1.47533200	-1.43735500
H	6.03943900	-0.20050900	-3.07969000
N	3.96928900	0.20563700	-0.39703400
C	4.18535100	1.66267800	-2.37274500
H	4.74654300	2.41525600	-1.79247600
H	4.52503600	1.70468500	-3.42636100
H	3.12390200	1.96553900	-2.32751900
H	3.68256500	-0.40733200	-2.34112000
H	5.75456200	-1.36882300	-1.76332400
H	7.78038500	-0.01662300	-1.22001200
Cl	1.96281900	2.31902200	0.29925200
C	1.51007800	1.52606200	4.24830900
C	2.72578600	2.11201700	3.88831800
C	1.14845200	1.51488100	5.60327500
C	3.57932900	2.63226000	4.86217000
H	3.00275500	2.16616700	2.82965400
C	1.99834300	2.03215200	6.57517700
H	0.17747900	1.10022300	5.89913300
C	3.22429700	2.58857000	6.20643500
H	4.53478400	3.06788400	4.55398700
H	1.69927100	2.00830000	7.62819400
H	3.89578300	2.99558700	6.96919900
C	-2.91923100	-3.30657800	1.52055900
C	-3.90609600	-3.25615900	0.52963900
C	-3.31296600	-3.55362200	2.83743900
C	-5.24402200	-3.47432300	0.83951300

H	-3.61166500	-3.03759900	-0.50363700
C	-4.65423000	-3.77445800	3.15219700
H	-2.55893100	-3.58557900	3.63078200
C	-5.62393400	-3.74525900	2.15374300
H	-5.99795400	-3.42105100	0.04766900
H	-4.94038700	-3.97530500	4.18965300
H	-6.67604300	-3.91992500	2.39967200
H	4.17636500	-5.36400200	0.13510300
C	-0.23962000	4.82338600	-0.87930000
C	-0.90171200	5.92283100	-0.31793000
C	1.11101700	4.98110700	-1.22097300
C	-0.24494000	7.13589000	-0.11008600
H	-1.95487400	5.83724900	-0.03229100
C	1.76535400	6.19238100	-1.02085300
H	1.66937700	4.13099700	-1.61942500
C	1.09217000	7.27871200	-0.46496600
H	-0.79138000	7.97496500	0.33246800
H	2.82209600	6.27917200	-1.29106500
H	1.61063900	8.22913500	-0.30427900
C	-2.45420000	3.65088300	-0.98198600
C	-3.15709800	4.26414900	-2.02573600
C	-3.16531300	3.27304400	0.15695900
C	-4.52559300	4.49728700	-1.92682000
H	-2.62334000	4.57659100	-2.92908700
C	-4.53555400	3.50552500	0.26427100
H	-2.63396000	2.78459100	0.97899100
C	-5.22217100	4.12035500	-0.77834000
H	-5.05432700	4.98088400	-2.75465200
H	-5.06309300	3.18582300	1.17021300
H	-6.29953000	4.29863600	-0.70234000

C	-0.88348000	-1.72131400	-3.29247000
C	-2.01747600	-1.40664400	-4.04439200
C	-0.88559100	-2.89338000	-2.52937400
C	-3.13620400	-2.23799000	-4.02552400
H	-2.02015700	-0.49770400	-4.65721600
C	-1.99390700	-3.73654000	-2.52205600
H	0.01220400	-3.15583000	-1.95479000
C	-3.12726300	-3.40597900	-3.26601400
H	-4.02342800	-1.96824300	-4.60749300
H	-1.96919300	-4.65246700	-1.91914200
H	-4.00430300	-4.06082300	-3.25115800
C	1.31121000	-1.36986100	-4.47165200
C	1.04008300	-2.49647500	-5.25273900
C	2.52950100	-0.70529200	-4.67885400
C	1.95909100	-2.95003300	-6.20131000
H	0.10056600	-3.04086900	-5.11899400
C	3.44830100	-1.15705200	-5.61795400
H	2.75659300	0.18803600	-4.08992700
C	3.16731800	-2.28776400	-6.38604400
H	1.72223800	-3.83743600	-6.79659500
H	4.39444700	-0.62116500	-5.74473000
H	3.89001000	-2.64884400	-7.12408700
C	0.18382900	3.45436500	-5.92319300
H	-0.41008000	4.38139100	-5.93459100
H	1.23912000	3.74398400	-6.06866000
H	-0.10472500	2.84729100	-6.79578600

INT6-L4

C	0.01638200	-0.12439700	-0.05579400
N	-0.68025200	-0.22028000	1.12283500
N	-0.42542200	1.04385400	-0.63973100

C	-0.63567900	-1.29438300	2.07682300
C	-0.77456300	-3.21834300	4.09617400
C	0.28497500	-1.23699700	3.13996000
C	-1.60247100	-2.31239100	1.98594700
C	-1.64601800	-3.26152700	3.00826800
C	0.17816800	-2.20018600	4.14392000
H	-2.38325200	-4.07043100	2.95091700
H	0.86819600	-2.16487600	4.99390500
C	-0.02464600	1.44760900	-1.95861000
C	0.72963800	2.20889500	-4.54948500
C	1.01444700	2.38176300	-2.12923000
C	-0.70889400	0.90990300	-3.06585700
C	-0.33186300	1.32865200	-4.34248600
C	1.38660300	2.72125400	-3.43040500
H	-0.88626900	0.94799200	-5.20838500
H	2.21948800	3.42061700	-3.57073900
C	-1.85285300	-0.07314800	-2.90831300
H	-2.05365800	-0.16239400	-1.82687100
C	1.68795000	3.07231200	-0.95834800
H	1.59045000	2.40031000	-0.08212200
C	-2.61544000	-2.34088400	0.85054400
H	-2.11677100	-1.89188700	-0.03248900
C	1.29268200	-0.10180100	3.23544600
H	1.54903000	0.17857300	2.19392000
C	-0.83166200	-4.26538400	5.16455800
H	-0.47654700	-3.88036100	6.13268800
Ni	1.52617400	-0.96199400	-0.79380300
H	-1.85230900	-4.65489300	5.29941800
H	-0.19093700	-5.12544600	4.90345100
C	-3.02821900	-3.74034800	0.40625500

C	-2.07791900	-4.76556600	0.33001700
C	-4.33082600	-4.01521700	-0.02676400
C	-2.41915500	-6.02479200	-0.15342800
H	-1.04771300	-4.57753600	0.64667200
C	-4.67266500	-5.27476500	-0.51931400
H	-5.09742100	-3.23538200	0.01594900
C	-3.71947400	-6.28741900	-0.58176400
H	-1.65187200	-6.80438900	-0.19797000
H	-5.69851100	-5.46330800	-0.84994600
H	-3.98898200	-7.27825700	-0.95933400
C	0.62250200	1.11311200	3.85651500
C	0.13171400	1.06762000	5.16627200
C	0.47483600	2.29629700	3.13295900
C	-0.49369600	2.17466000	5.73125300
H	0.24766900	0.14984600	5.75340800
C	-0.16276800	3.40548800	3.68904300
H	0.84994200	2.34190100	2.10313400
C	-0.64865400	3.34724000	4.99128600
H	-0.86755800	2.12132100	6.75827200
H	-0.29365100	4.31127800	3.08783600
H	-1.15525200	4.21249400	5.42870300
C	-3.46858900	4.88338400	0.04950700
C	-4.06474800	4.72123700	1.28504600
C	-3.77012200	3.57776800	2.07776300
C	-2.85731700	2.66758700	1.54220600
C	-2.21930700	2.83803900	0.27752400
C	-2.54134700	3.94546800	-0.47688800
H	-5.02273200	3.93857100	3.82811800
H	-3.70725700	5.76782600	-0.54897500
H	-4.77111500	5.46988900	1.65859100

C	-4.30627500	3.25711000	3.35763200
C	-2.46598200	1.46490900	2.19837500
H	-2.10143000	4.11283600	-1.46446700
C	-3.00375300	1.17627600	3.43048900
C	-3.92734400	2.09554500	3.99879300
H	-2.73090000	0.26606900	3.97467100
H	-4.35036000	1.86583200	4.98139300
C	-1.36539000	1.66228900	0.16410600
C	-1.51755300	0.86725900	1.27105900
C	2.02126500	-3.29453800	0.73148500
C	2.08386800	-4.67438200	0.92955200
C	1.49665700	-5.54415400	0.00850400
C	0.83127400	-5.01559100	-1.09605800
C	0.73535900	-3.63284000	-1.27026600
C	1.33835200	-2.75809600	-0.36298900
H	2.56721100	-2.62742800	1.40833400
H	2.62944300	-5.07351200	1.79256900
H	0.36686500	-5.68177100	-1.83283300
H	0.20243100	-3.24214800	-2.14215000
C	5.55820500	-0.81644500	1.14006600
C	4.39513400	-0.76498100	0.38877500
C	4.31556400	-1.32042700	-0.91068700
C	5.47932600	-1.94617400	-1.42808500
C	6.64194100	-1.98053300	-0.65577600
C	6.70328400	-1.42184300	0.61781900
H	5.57025800	-0.37269900	2.14149400
H	3.50612100	-0.26224400	0.79370700
C	5.43288300	-2.58722900	-2.78601300
H	7.52534500	-2.47814100	-1.07520200
H	7.62867600	-1.46278800	1.19926100

C	4.00471800	-2.94666200	-3.14888600
C	3.08824700	-1.74121700	-2.97725900
H	5.85283500	-1.90316300	-3.55108200
H	3.93586900	-3.32981800	-4.18261300
N	3.13481900	-1.20920400	-1.61769900
H	3.64206300	-3.74845300	-2.47746600
H	6.08388500	-3.47891600	-2.80104500
C	2.62455900	-0.43056200	3.90570600
C	3.37348100	0.59831500	4.49250800
C	3.19383200	-1.71018000	3.87144700
C	4.63517200	0.35783300	5.03123200
H	2.96378900	1.61234100	4.52926300
C	4.45448300	-1.95420300	4.41103500
H	2.64534900	-2.54000000	3.41836500
C	5.18169200	-0.92239300	4.99766500
H	5.19383900	1.18285700	5.48296400
H	4.87194000	-2.96412200	4.36342200
H	6.17099700	-1.11415700	5.42284200
C	-3.81857200	-1.46958700	1.17156500
C	-4.26768000	-0.50795200	0.26437800
C	-4.53495400	-1.64395800	2.36004000
C	-5.38557900	0.27814100	0.53986500
H	-3.73113500	-0.36470100	-0.68053300
C	-5.65864600	-0.87055000	2.63641600
H	-4.20654300	-2.39965400	3.08193600
C	-6.08483200	0.09977800	1.73015400
H	-5.70454500	1.03403200	-0.18565700
H	-6.20521300	-1.02307400	3.57216100
H	-6.96014300	0.71660000	1.95361700
H	1.56906500	-6.62780500	0.14694400

C	1.01152500	4.38193800	-0.59012700
C	0.35673900	5.18743500	-1.52564400
C	1.13203300	4.85782800	0.72064100
C	-0.17176200	6.42566200	-1.15981400
H	0.25361300	4.84540900	-2.56075300
C	0.59927700	6.08863900	1.09205700
H	1.68074500	4.25928200	1.45734500
C	-0.05951600	6.87888000	0.15092400
H	-0.67841900	7.03965700	-1.91099800
H	0.71076100	6.43930900	2.12302600
H	-0.47639700	7.84814400	0.43896200
C	3.18552800	3.24706000	-1.18797400
C	3.81110100	4.49322700	-1.26917600
C	3.96340000	2.09291700	-1.32847900
C	5.18877900	4.57810100	-1.48081000
H	3.22628000	5.41254000	-1.16210800
C	5.33681300	2.17536500	-1.52844900
H	3.47729000	1.10994000	-1.30099400
C	5.95536000	3.42354600	-1.60657100
H	5.66453400	5.56170500	-1.54111500
H	5.92103800	1.25223200	-1.61366700
H	7.03598000	3.49436400	-1.76162300
C	-1.57451900	-1.48905100	-3.38566000
C	-0.53385800	-1.84712900	-4.24519300
C	-2.45133300	-2.49379200	-2.95104600
C	-0.38982200	-3.16806100	-4.67628700
H	0.19054400	-1.09720300	-4.57783500
C	-2.30586200	-3.81120900	-3.36980000
H	-3.27193400	-2.23446000	-2.26920700
C	-1.27458900	-4.15165900	-4.24666300

H	0.43886700	-3.42666000	-5.34322500
H	-2.99663000	-4.57483800	-2.99647700
H	-1.15428200	-5.18610200	-4.58138100
C	-3.12253600	0.50500600	-3.51984600
C	-3.59403400	0.13517100	-4.78191300
C	-3.82845800	1.47630800	-2.80134800
C	-4.73419300	0.73545700	-5.31584100
H	-3.06967600	-0.64010100	-5.35142800
C	-4.96717900	2.07560700	-3.33036800
H	-3.47531900	1.76867600	-1.80579600
C	-5.42353700	1.70754800	-4.59532200
H	-5.08959300	0.43389500	-6.30567200
H	-5.50003700	2.83354500	-2.74682200
H	-6.31932000	2.17315400	-5.01622300
C	1.17939600	2.56202300	-5.93190200
H	0.35378100	2.50499500	-6.65747000
H	1.61299800	3.57264300	-5.97528200
H	1.96236800	1.86164800	-6.27287000
H	2.05193800	-2.09684200	-3.14739300
C	3.35016500	-0.63296400	-3.98919300
H	3.29801600	-1.01202200	-5.02629400
H	2.59996900	0.16913900	-3.86969800
H	4.34142400	-0.16956600	-3.84105800

TS7-L4

C	0.00414000	0.07854600	-0.04474900
N	0.65778900	0.16367900	1.16335700
N	0.66722800	-0.91767600	-0.73175800
C	0.39237600	1.11372700	2.20413800
C	0.10175000	2.85465900	4.36855300
C	-0.52864100	0.79464500	3.21818500

C	1.14696300	2.30003000	2.23579100
C	0.97930000	3.15356300	3.32658200
C	-0.63645800	1.67257900	4.29779100
H	1.54745700	4.09030600	3.36028100
H	-1.33381000	1.43770200	5.10943600
C	0.36461300	-1.25101000	-2.09218700
C	-0.21303200	-1.88614500	-4.76183800
C	-0.48500300	-2.33418200	-2.37716300
C	0.94750000	-0.49383000	-3.12578000
C	0.66931500	-0.85303800	-4.44530100
C	-0.78184200	-2.60872800	-3.71252700
H	1.15816700	-0.30097700	-5.25691300
H	-1.47581700	-3.42753000	-3.93802900
C	1.87814500	0.66868600	-2.84261100
H	2.04861000	0.68141800	-1.75244400
C	-1.04426100	-3.22393000	-1.28503200
H	-1.00376000	-2.64344400	-0.34390500
C	2.15727700	2.61653100	1.14337600
H	1.75279600	2.17890300	0.20796800
C	-1.31080000	-0.50946400	3.16783400
H	-1.50028600	-0.71608100	2.09441000
C	-0.07730600	3.80152100	5.51426600
H	-0.37242700	3.27619800	6.43550000
Ni	-1.61553700	0.76487500	-0.68826900
H	0.84151300	4.37191800	5.71922600
H	-0.87011100	4.53703300	5.29295100
C	2.30316200	4.10631100	0.86086200
C	1.14994000	4.88643300	0.71266600
C	3.54666200	4.71716400	0.67123200
C	1.23704600	6.23945000	0.40460900

H	0.16450100	4.42452400	0.83679300
C	3.63498900	6.07343500	0.35184800
H	4.46586900	4.13172600	0.76964600
C	2.48224200	6.84183200	0.22308700
H	0.31776100	6.82515600	0.30313000
H	4.61914500	6.52986800	0.20938100
H	2.55197100	7.90702100	-0.01678800
C	-0.44090600	-1.64448900	3.68024800
C	0.02940800	-1.64487600	4.99829900
C	-0.08481500	-2.70583800	2.84778200
C	0.83253500	-2.67963100	5.46743900
H	-0.24547200	-0.82182500	5.66761700
C	0.73115600	-3.73954100	3.30778100
H	-0.43715500	-2.70908600	1.80927300
C	1.19060300	-3.72951800	4.62102100
H	1.18696400	-2.66519700	6.50264400
H	1.01908500	-4.54550400	2.62418100
H	1.83565200	-4.53492300	4.98428100
C	4.30379200	-4.24616500	-0.32367800
C	4.84754300	-4.11274800	0.93893700
C	4.35408100	-3.11926700	1.82989600
C	3.31130600	-2.32208000	1.35602000
C	2.73060200	-2.46416800	0.05998800
C	3.24115100	-3.42463100	-0.78599800
H	5.61673000	-3.44794300	3.57969800
H	4.69522700	-5.01513600	-0.99657300
H	5.66260600	-4.76965400	1.26008100
C	4.80340000	-2.85052300	3.15463100
C	2.70462000	-1.28126800	2.11693300
H	2.84725500	-3.56353400	-1.79721000

C	3.15950700	-1.04126200	3.39208200
C	4.21745300	-1.84561400	3.89703600
H	2.72063400	-0.25658700	4.01711400
H	4.57517800	-1.65515500	4.91345300
C	1.69002500	-1.44448300	0.03642300
C	1.68001200	-0.76071600	1.22493600
C	-2.90247300	2.89084100	0.81970600
C	-2.97512800	4.22763900	1.19745200
C	-2.43477000	5.23354800	0.39321900
C	-1.76973000	4.85863100	-0.77375000
C	-1.65616100	3.52011500	-1.13922800
C	-2.26622700	2.49957700	-0.37725700
H	-3.39440900	2.13892100	1.44071200
H	-3.48688500	4.48629500	2.13157500
H	-1.29593700	5.61611500	-1.40981800
H	-1.08170800	3.27479000	-2.03603500
C	-5.65313200	-0.20013500	1.05160600
C	-4.47199600	0.17985400	0.43227700
C	-4.46416300	0.89698800	-0.78151200
C	-5.71107800	1.22574000	-1.36587300
C	-6.88821400	0.82111700	-0.73123500
C	-6.88051100	0.11421400	0.46701400
H	-5.60764700	-0.75778500	1.99300400
H	-3.50821400	-0.09425600	0.87853100
C	-5.78082700	1.97178500	-2.66936300
H	-7.84291800	1.08386100	-1.20346200
H	-7.81907500	-0.18796500	0.93997700
C	-4.45563200	2.62073900	-3.01426300
C	-3.30829300	1.64435500	-2.78345600
H	-6.07991500	1.27267100	-3.47586800

H	-4.44856600	2.97965600	-4.05836400
N	-3.25106700	1.25880700	-1.37166100
H	-4.28274600	3.50075400	-2.36668200
H	-6.58828400	2.72353600	-2.62373000
C	-2.68381800	-0.47830400	3.83054900
C	-3.22651600	-1.63885800	4.39578300
C	-3.48271600	0.67251100	3.81018800
C	-4.51180000	-1.64504700	4.93488000
H	-2.63516300	-2.55926200	4.41707700
C	-4.76546800	0.67059800	4.35076900
H	-3.09263300	1.59315400	3.36657100
C	-5.28614900	-0.48826100	4.92190600
H	-4.90750300	-2.56663200	5.37199600
H	-5.36501000	1.58507600	4.31531900
H	-6.29281000	-0.49057900	5.34974300
C	3.49427100	1.94834200	1.41009200
C	4.13776700	1.22465500	0.40436200
C	4.13985900	2.08509300	2.64327400
C	5.38114100	0.63418600	0.62093100
H	3.65661900	1.11574900	-0.57366500
C	5.38863000	1.50946300	2.86227300
H	3.65578700	2.65393200	3.44507300
C	6.01284900	0.77681800	1.85345400
H	5.85223500	0.06127400	-0.18476500
H	5.87771400	1.62901900	3.83400000
H	6.98808700	0.31423800	2.03085100
H	-2.52233600	6.28658300	0.67781700
C	-0.21966600	-4.47951700	-1.05701900
C	0.59938300	-5.05129600	-2.03357300
C	-0.33185500	-5.13339700	0.17643200

C	1.29463500	-6.23500900	-1.78160300
H	0.70524400	-4.56449800	-3.00866800
C	0.36612300	-6.30842600	0.43473100
H	-0.99567800	-4.71527700	0.94280300
C	1.18728700	-6.86478300	-0.54603300
H	1.93010100	-6.66428900	-2.56255500
H	0.26188000	-6.79842000	1.40797000
H	1.73623500	-7.78952900	-0.34680900
C	-2.51671300	-3.52604900	-1.51821100
C	-2.98633600	-4.77681000	-1.92506700
C	-3.43304400	-2.48485700	-1.34103300
C	-4.34820700	-4.97732600	-2.15400300
H	-2.28521000	-5.60736800	-2.06040800
C	-4.79199200	-2.68385300	-1.56051900
H	-3.06201800	-1.49600500	-1.03704800
C	-5.25264900	-3.93498900	-1.97204700
H	-4.70324500	-5.96234600	-2.47198600
H	-5.49089500	-1.85604700	-1.39829600
H	-6.32076300	-4.09695500	-2.14397700
C	1.33213200	2.04631400	-3.18181300
C	0.29063700	2.28934300	-4.08033500
C	1.95814300	3.14817500	-2.58197200
C	-0.09709100	3.59615100	-4.38474500
H	-0.24148500	1.45442800	-4.54643800
C	1.56773700	4.45038300	-2.87259700
H	2.77878300	2.98102500	-1.87275700
C	0.53908800	4.67951100	-3.78685600
H	-0.91890800	3.76215900	-5.08858600
H	2.06604000	5.28766600	-2.37271500
H	0.22769600	5.70131400	-4.02297000

C	3.24086700	0.41348800	-3.47358200
C	3.65745100	1.02418900	-4.65889300
C	4.10216500	-0.49938700	-2.85355600
C	4.89986600	0.71845600	-5.21456700
H	3.00734600	1.75663100	-5.14987500
C	5.34266100	-0.80424000	-3.40407000
H	3.79087700	-0.97995300	-1.91876900
C	5.74596200	-0.19539400	-4.59201500
H	5.21058300	1.20666700	-6.14308800
H	5.99758600	-1.52142800	-2.89856200
H	6.72126900	-0.42896800	-5.02862600
C	-0.56651300	-2.18837900	-6.18431300
H	-0.81449800	-3.25121200	-6.32652600
H	-1.45173700	-1.60761400	-6.49797300
H	0.25245000	-1.92748300	-6.87192900
C	-3.35007400	0.41966800	-3.68617800
H	-4.20879200	-0.23718300	-3.46610800
H	-3.41204100	0.71781200	-4.74847300
H	-2.43723700	-0.18585500	-3.54640700
H	-2.36871200	2.17337600	-3.00694100

INT8-L4

C	-0.09010600	-0.11597800	-0.30055000
N	-0.61787100	-0.91513800	0.72177900
N	-0.60086600	1.14543400	-0.00474600
C	-0.43727500	-2.32873100	0.85619200
C	-0.21630400	-5.08613500	1.32136100
C	0.63860700	-2.81912100	1.62370800
C	-1.39657300	-3.20199100	0.31061700
C	-1.26149100	-4.57174700	0.55659000

C	0.72223200	-4.19316200	1.84397400
H	-1.99781900	-5.26314800	0.13005900
H	1.55335800	-4.58481800	2.44182100
C	-0.55893700	2.32927800	-0.80821700
C	-0.74088600	4.83718000	-2.06635000
C	0.44247800	3.28970800	-0.59214700
C	-1.63571900	2.59684500	-1.68101200
C	-1.71258300	3.85668200	-2.27477200
C	0.33472300	4.52353900	-1.23908300
H	-2.57261400	4.08751500	-2.91498000
H	1.12066300	5.27089400	-1.07590100
C	-2.70188400	1.55956500	-1.98835100
H	-2.60990800	0.76897900	-1.22330800
C	1.63946100	3.03567700	0.30682900
H	1.71666900	1.93786100	0.43943200
C	-2.59616500	-2.66910800	-0.45426500
H	-2.27388400	-1.70990900	-0.90690800
C	1.64917100	-1.85961300	2.23581200
H	1.83046400	-1.08395300	1.46578300
C	-0.07992300	-6.55838300	1.55984300
H	0.24828000	-6.77195200	2.58927200
Ni	0.91895500	-0.74525400	-1.73049600
H	-1.02651600	-7.09088500	1.38288900
H	0.67537600	-7.00160400	0.88817400
C	-3.05119400	-3.52996100	-1.62923600
C	-2.12337100	-4.21029400	-2.42800900
C	-4.39740100	-3.57918100	-2.01197100
C	-2.51820000	-4.90566900	-3.56721800
H	-1.06704700	-4.19632300	-2.14807800
C	-4.79752100	-4.27245200	-3.15407700

H	-5.15264500	-3.06265200	-1.41172000
C	-3.86099400	-4.93900800	-3.93900100
H	-1.76667500	-5.42555100	-4.16916800
H	-5.85679400	-4.29187400	-3.42709600
H	-4.17530000	-5.48687200	-4.83205800
C	1.06475900	-1.15036300	3.44043600
C	0.59208400	-1.86665800	4.54573500
C	0.98884400	0.24275000	3.46904800
C	0.07083500	-1.20268500	5.65286900
H	0.63811100	-2.96238600	4.53502600
C	0.44909100	0.91361300	4.56583200
H	1.33907900	0.81784800	2.60279500
C	-0.00617900	0.19004900	5.66453900
H	-0.29187800	-1.77780000	6.51065800
H	0.38240800	2.00750100	4.54627100
H	-0.43521500	0.70982400	6.52636900
C	-3.46457900	3.68099400	3.10510200
C	-3.92418600	2.80144300	4.06652000
C	-3.53706500	1.43201800	4.02769000
C	-2.68335900	1.05680400	2.99096400
C	-2.19677400	1.95872600	1.99755700
C	-2.59761700	3.27606400	2.05477000
H	-4.60430600	0.63526300	5.75867200
H	-3.78021400	4.72791600	3.14526700
H	-4.59270400	3.15186600	4.85989800
C	-3.93636500	0.39824700	4.92385800
C	-2.22218200	-0.27708100	2.78478000
H	-2.28577700	3.99916700	1.29371800
C	-2.62578100	-1.25423100	3.66328900
C	-3.48835600	-0.89264200	4.73559300

H	-2.30261600	-2.29412000	3.54834400
H	-3.80890400	-1.67548900	5.43002400
C	-1.39099100	1.11256200	1.13210400
C	-1.40364600	-0.17623700	1.58333700
C	2.48822300	-2.23551500	-2.10590000
C	1.22108400	-2.74295300	-2.44672700
C	0.39366300	-2.02931700	-3.33939500
C	0.82401500	-0.78524700	-3.84722200
C	2.08395800	-0.26374100	-3.48667600
C	2.93142700	-0.99746800	-2.62532200
H	3.16750100	-2.80135000	-1.46093900
H	0.89999100	-3.70203700	-2.02448300
H	0.16830400	-0.22198200	-4.51511200
H	2.43496700	0.69219800	-3.88985300
C	4.47778400	0.14937200	1.21454100
C	3.97095400	-0.02401400	-0.06431000
C	4.79712500	-0.44406500	-1.12239500
C	6.15792900	-0.70688700	-0.85193600
C	6.64490000	-0.50539400	0.44344400
C	5.82834900	-0.07562700	1.48046200
H	3.80128500	0.44661300	2.02531900
H	2.90429200	0.13225900	-0.26768200
C	7.06582700	-1.24512500	-1.92199400
H	7.70282500	-0.72192900	0.63497500
H	6.22906900	0.05473700	2.48930800
C	6.26037900	-1.88013000	-3.03699200
C	5.18605500	-0.91491200	-3.52339200
H	7.70589000	-0.43977800	-2.33310100
H	6.90519700	-2.17973700	-3.88061900
N	4.27219200	-0.57132700	-2.41850400

H	5.76501500	-2.79763700	-2.66617500
H	7.76541600	-1.97470700	-1.47841200
C	3.00909100	-2.48752100	2.50222200
C	3.66376600	-2.41530200	3.73312000
C	3.67988800	-3.09938900	1.43545600
C	4.94656200	-2.94506700	3.89236600
H	3.17702800	-1.92905000	4.58333600
C	4.95766500	-3.61992800	1.58555700
H	3.17305500	-3.15117700	0.46730100
C	5.59948300	-3.54629300	2.82275200
H	5.43874100	-2.87732700	4.86737600
H	5.46225200	-4.07717800	0.72816000
H	6.60704400	-3.95371200	2.94757200
C	-3.74363500	-2.34667300	0.48953600
C	-4.36443400	-1.09758700	0.44441000
C	-4.23774100	-3.29886800	1.38729800
C	-5.43653900	-0.79173000	1.28009400
H	-4.00035600	-0.34173000	-0.25891500
C	-5.31304900	-3.00259100	2.22064700
H	-3.77251500	-4.28913500	1.43713900
C	-5.91330200	-1.74477100	2.17521000
H	-5.89394900	0.20178200	1.22509900
H	-5.68418400	-3.76155800	2.91624900
H	-6.75076100	-1.50891400	2.83823400
H	-0.59281600	-2.41259300	-3.61831600
C	1.52978200	3.63350300	1.69739500
C	0.54682200	4.55019300	2.07167200
C	2.48613400	3.26361500	2.65445500
C	0.50750100	5.07287100	3.36611100
H	-0.20274300	4.86858200	1.34158500

C	2.45849300	3.78915300	3.94046300
H	3.27586300	2.55926300	2.36745600
C	1.46269900	4.69779200	4.30425800
H	-0.28090200	5.78198500	3.63712300
H	3.21638200	3.48308000	4.66772500
H	1.43490600	5.11015600	5.31705400
C	2.90392200	3.46044200	-0.42737800
C	3.69465900	4.54606800	-0.04675600
C	3.24465300	2.75355700	-1.58524800
C	4.80531500	4.91537700	-0.80795800
H	3.44431700	5.11578200	0.85412200
C	4.34631800	3.12268700	-2.34506100
H	2.62061800	1.90264300	-1.88794500
C	5.13481400	4.20679500	-1.95858500
H	5.41507600	5.76819400	-0.49449700
H	4.59210400	2.55181100	-3.24386300
H	6.00464400	4.49718400	-2.55527800
C	-2.52582200	0.86213500	-3.32787300
C	-1.91157300	1.44980000	-4.43610700
C	-3.09464200	-0.40823200	-3.48308100
C	-1.88345100	0.79227500	-5.66717200
H	-1.44146900	2.43334900	-4.34270300
C	-3.05211100	-1.07700900	-4.70194200
H	-3.59383900	-0.88268300	-2.62890100
C	-2.44866200	-0.47273600	-5.80539300
H	-1.40352700	1.27417100	-6.52463800
H	-3.49698300	-2.07497700	-4.78410300
H	-2.41830200	-0.98877800	-6.76933900
C	-4.10617700	2.13808000	-1.84668000
C	-4.86395800	2.55500400	-2.94526100

C	-4.65634200	2.28994800	-0.56829900
C	-6.12408800	3.12576300	-2.76768400
H	-4.46811200	2.42521800	-3.95822400
C	-5.91290600	2.86009800	-0.38733900
H	-4.08840100	1.95546100	0.30579100
C	-6.65313000	3.28428100	-1.48966800
H	-6.69928700	3.44527500	-3.64189700
H	-6.31180000	2.97076300	0.62663000
H	-7.64240500	3.73065900	-1.35328600
C	-0.83603200	6.17091000	-2.74023700
H	-0.33173400	6.95700600	-2.15785300
H	-0.35579900	6.14924000	-3.73385400
H	-1.88340900	6.47307400	-2.89468200
H	4.57117800	-1.44262600	-4.27393700
C	5.77221800	0.32252700	-4.18169900
H	4.98035200	0.92516500	-4.65535000
H	6.28776100	0.96365900	-3.44772000
H	6.49843500	0.04350900	-4.96381200

References

1. Wang, Z.-C., Xie, P.-P., Xu, Y., Hong, X. & Shi, S.-L. Low-Temperature Ni-Catalyzed C−N Cross-Coupling via Kinetic Resolution Enabled by a Bulky and Flexible Chiral N-Heterocyclic Carbene Ligand. *Angew. Chem., Int. Ed*. **60**, 16077–16084 (2021).

2. Cran, J. W., Vidhani, D. V. & Krafft, M. E. Copper-Catalyzed N-tert-Butylation of Aromatic Amines under Mild Conditions Using tert-Butyl 2,2,2-Trichloroacetimidate. *Synlett*. **25**, 1550-1554 (2014).

3. Pirali, T., Zhang, F., Miller, A. H. & Head, J. L. McAusland, D., Greaney, M. F. Transition-Metal-Free Direct Arylation of Anilines. *Angew. Chem. Int. Ed.* **51**, 1006-1009 (2012).

4. Li, C., Kawamata, Y., Nakamura, H., Vantourout, J, C., Liu, Z., Hou, Q., Bao, D., Yan, M. & Baran, P. S. Electrochemically Enabled, Nickel-Catalyzed Amination. *Angew. Chem. Int. Ed.* **56**, 13088-13093 (2017).

5. Zhou, W., Fan, M., Yin, J., Jiang, Y. & Ma, D. CuI/Oxalic Diamide Catalyzed Coupling Reaction of (Hetero)Aryl Chlorides and Amines. *J. Am. Chem. Soc.* **137**, 11942 -11945 (2015).

6. Huang, J. & Yang, L. Nickel-Catalyzed Amination of Aryl Phosphates through Cleaving Aryl C–O Bonds. *Org. Lett.* **13**, 3750-3753 (2011).

7. Jiang, J., Zhu, H., Shen, Y. & Tu, T. Acenaphthoimidazolium chloride-enabled nickel-catalyzed amination of bulky aryl tosylates. *Org. Chem. Front.* **1**, 1172-1175 (2014).

8. Miki, Y., Hirano, K., Satoh, T. & Miura, M. Copper-Catalyzed Electrophilic Amination of Arylsilanes with Hydroxylamines. *Org. Lett.* **15**, 172-175 (2013).

9. Moehrle M. Cyclisierungen über Enamin-Zwischenstufen bei Amindehydrierungen/Cyclizations via Enamine Intermediates with Amine Dehydrogenations. *Zeitschrift fur Naturforschung - Section B Journal of Chemical Sciences*, **54**, 214-224 (1999).

10. M. J. Frisch, G. W. Trucks, H. B. Schlegel, G. E. Scuseria, M. A. Robb, J. R. Cheeseman, G. Scalmani, V. Barone, B. Mennucci, G. A. Petersson, H. Nakatsuji, M. Caricato, X. Li, H. P. Hratchian, A. F. Izmaylov, J. Bloino, G. Zheng, J. L. Sonnenberg, M. Hada, M. Ehara, K. Toyota, R. Fukuda, J. Hasegawa, M. Ishida, T. Nakajima, Y. Honda, O. Kitao, H. Nakai, T. Vreven, J. A., Jr. Montgomery, J. E. Peralta, F. Ogliaro, M. Bearpark, J. J. Heyd, E. Brothers, K. N. Kudin, V. N. Staroverov, R. Kobayashi, J. Normand, K. Raghavachari, A. Rendell, J. C. Burant, S. S. Iyengar, J. Tomasi, M. Cossi, N. Rega, J. M. Millam, M. Klene, J. E. Knox, J. B. Cross, V. Bakken, C. Adamo, J. Jaramillo, R. Gomperts, R. E. Stratmann, O. Yazyev, A. J. Austin, R. Cammi, C. Pomelli, J. W. Ochterski, R. L. Martin, K. Morokuma, V. G. Zakrzewski, G. A. Voth, P. Salvador, J. J. Dannenberg, S. Dapprich, A. D. Daniels, O. Farkas, J. B. Foresman, J. V. Ortiz, J. Cioslowski, D. J. Fox, Gaussian 16, Revision A.03; Gaussian Inc., Wallingford, CT, 2016.

11. Zhao, Y., & Truhlar, D. G. The M06 suite of density functionals for main group thermochemistry, thermochemical kinetics, noncovalent interactions, excited states, and transition elements: two new functionals and systematic testing of four M06-class functionals and 12 other functionals. *Theor. Chem. Acc.* **120**, 215-241 (2008).

12. Weigend, F., & Ahlrichs, R. Balanced basis sets of split valence, triple zeta valence and quadruple zeta valence quality for H to Rn: Design and assessment of accuracy. *Phys. Chem. Chem. Phys.* **7**, 3297-3305 (2005).

13. Marenich, A. V., Cramer, C. J., & Truhlar, D. G. Universal solvation model based on solute electron density and on a continuum model of the solvent defined by the bulk dielectric constant and atomic surface tensions. *J. Phys. Chem. B.* **113**, 6378-6396 (2009).

NMR Spectra

3a

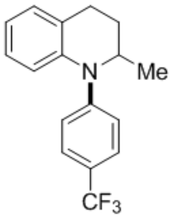

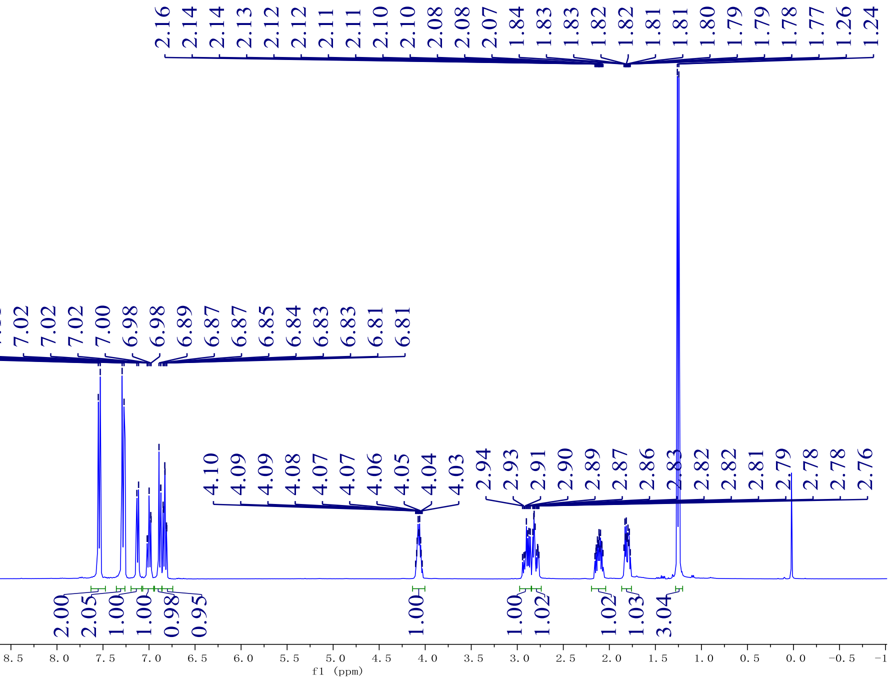

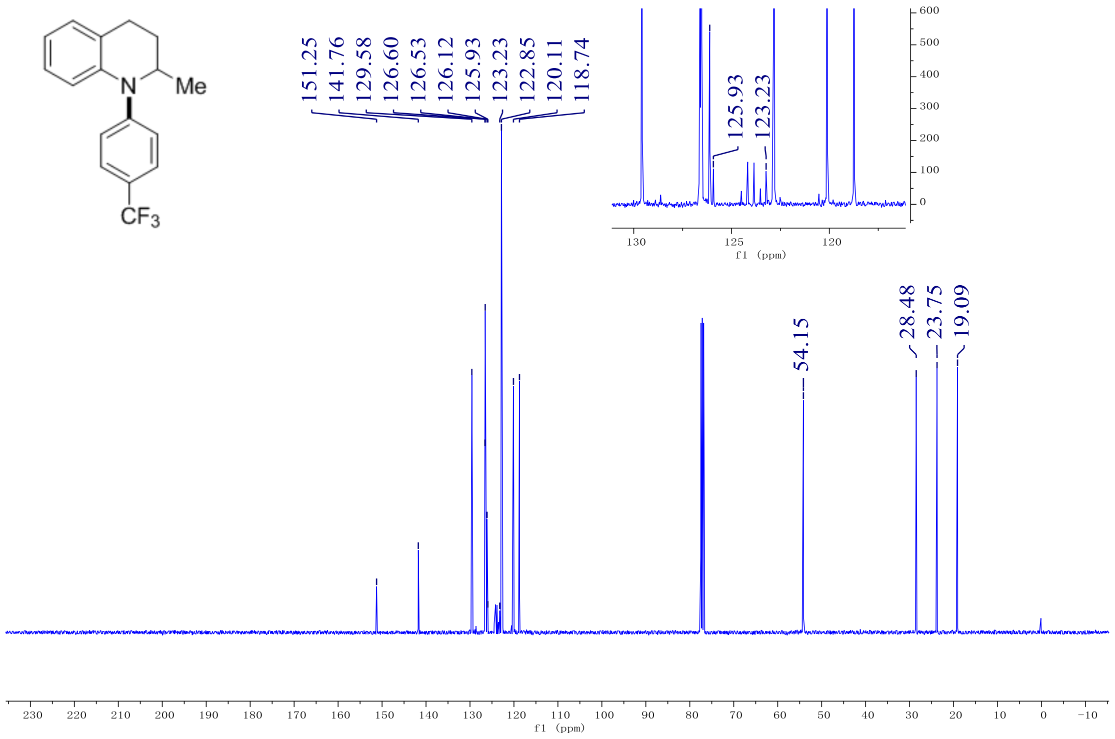

N
Me
CF3
151.25
141.76
129.58
126.60
126.53
126.12
125.93
123.23
122.85
120.11
118.74
125.93
123.23
54.15
28.48
23.75
19.09
f1 (ppm)

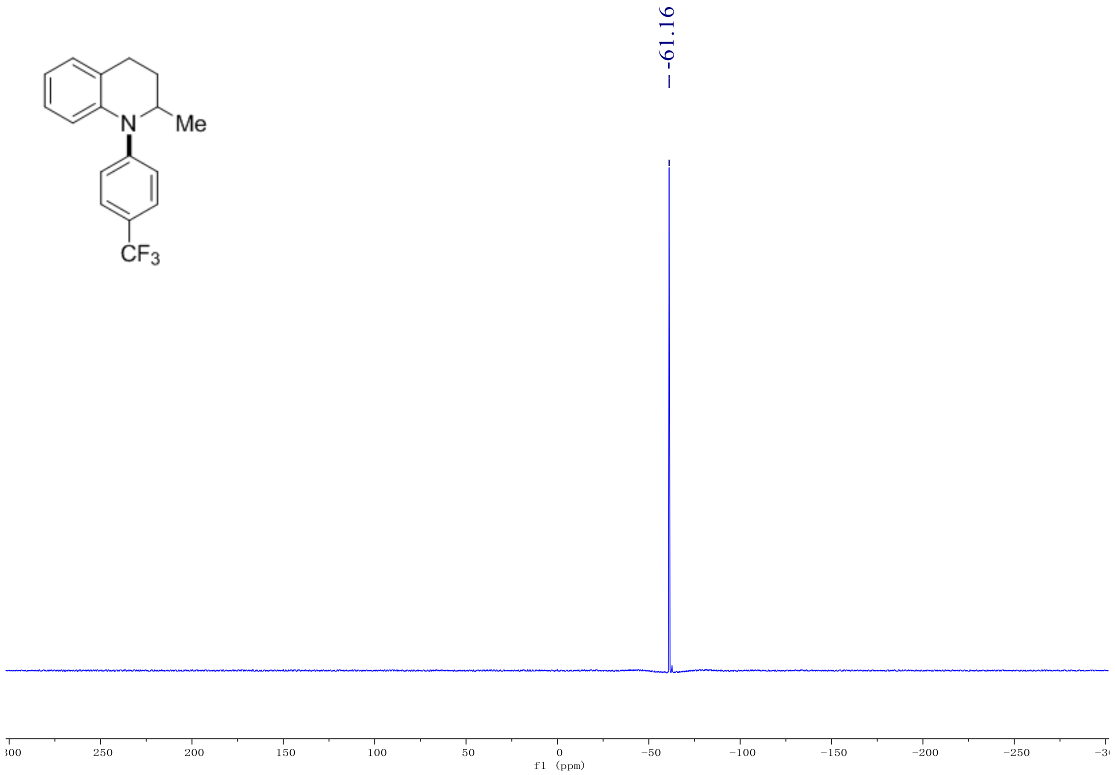

N
Me
CF3
-61.16
f1 (ppm)

3b

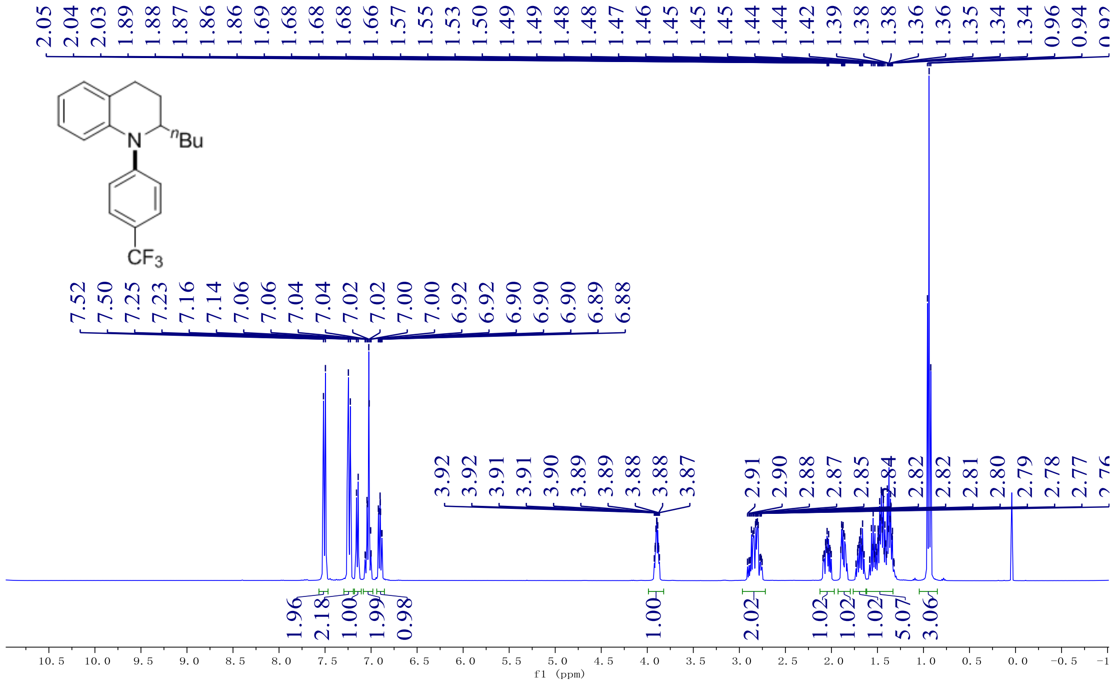
2.05 2.04 2.03 1.89 1.88 1.87 1.86 1.86 1.69 1.68 1.68 1.68 1.66 1.57 1.55 1.53 1.50 1.49 1.49 1.48 1.48 1.47 1.46 1.45 1.45 1.45 1.44 1.44 1.42 1.39 1.38 1.38 1.36 1.36 1.35 1.34 1.34 0.96 0.94
N
nBu
CF3
7.52 7.50 7.25 7.23 7.16 7.14 7.06 7.06 7.04 7.04 7.02 7.02 7.00 7.00 6.92 6.92 6.90 6.90 6.90 6.89 6.88
3.92 3.92 3.91 3.91 3.90 3.89 3.89 3.88 3.88 3.87
2.91 2.90 2.88 2.87 2.85 2.84 2.82 2.82 2.81 2.80 2.79 2.78 2.77
1.96 2.18 1.00 1.99 0.98 1.00 2.02 1.02 1.02 1.02 5.07 3.06
10.5 10.0 9.5 9.0 8.5 8.0 7.5 7.0 6.5 6.0 5.5 5.0 4.5 4.0 3.5 3.0 2.5 2.0 1.5 1.0 0.5 0.0 −0.5 −1
f1 (ppm)

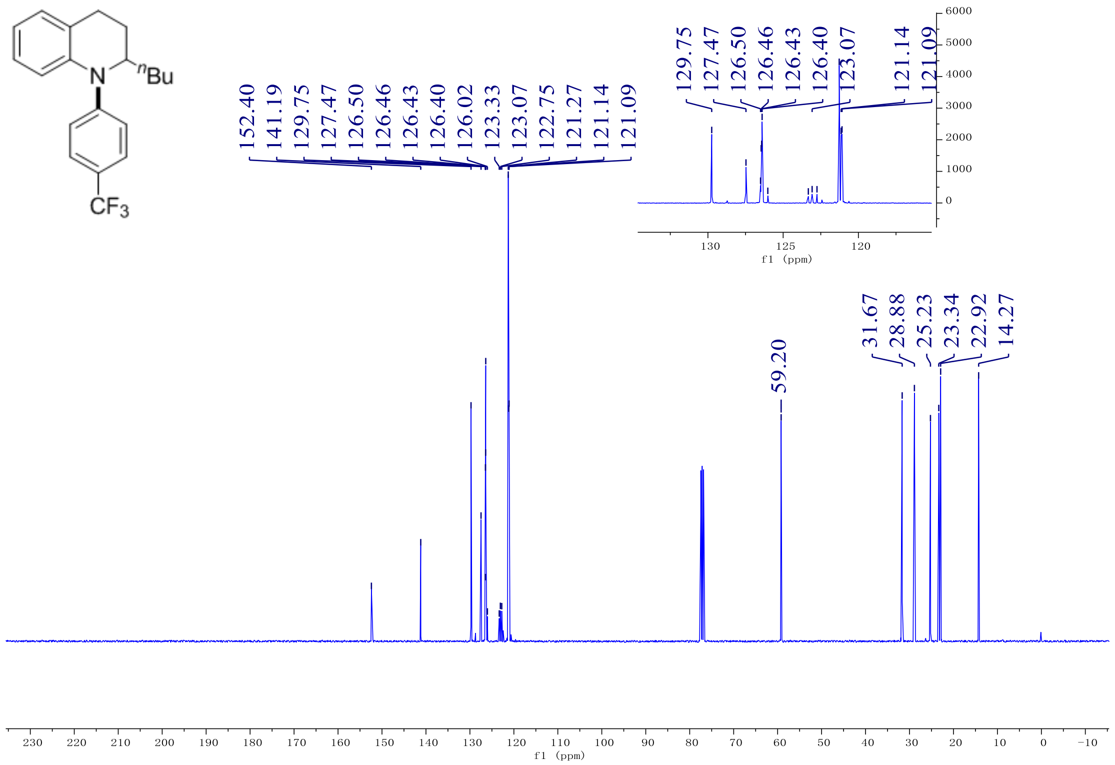
N
nBu
CF3
152.40 141.19 129.75 127.47 126.50 126.46 126.43 126.40 126.02 123.33 123.07 122.75 121.27 121.14 121.09
129.75 127.47 126.50 126.46 126.43 126.40 123.07 121.14 121.09
6000 5000 4000 3000 2000 1000 0
130 125 120
f1 (ppm)
59.20
31.67 28.88 25.23 23.34 22.92 14.27
230 220 210 200 190 180 170 160 150 140 130 120 110 100 90 80 70 60 50 40 30 20 10 0 −10
f1 (ppm)

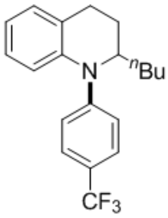
N
nBu
CF3

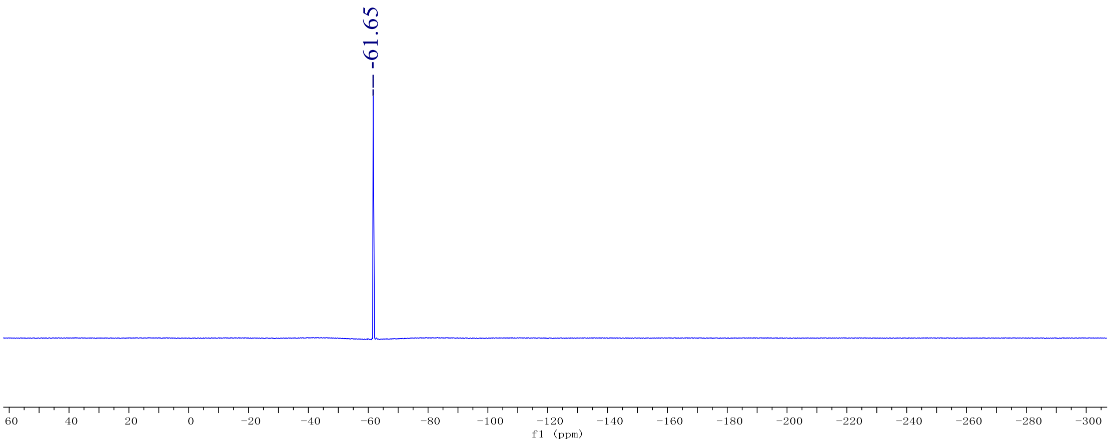
-61.65
60 40 20 0 −20 −40 −60 −80 −100 −120 −140 −160 −180 −200 −220 −240 −260 −280 −300
f1 (ppm)

3c

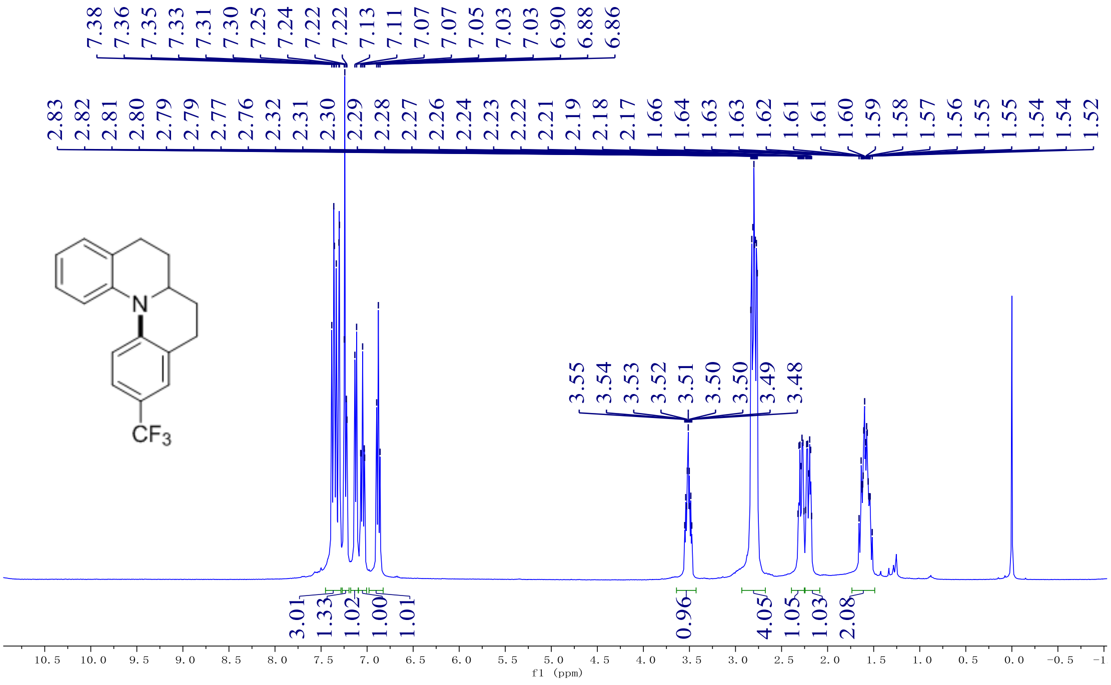
7.38 7.36 7.35 7.33 7.31 7.30 7.25 7.24 7.22 7.22 7.13 7.11 7.07 7.07 7.05 7.03 7.03 6.90 6.88 6.86
2.83 2.82 2.81 2.80 2.79 2.79 2.77 2.76 2.32 2.31 2.30 2.29 2.28 2.27 2.26 2.24 2.23 2.22 2.21 2.19 2.18 2.17 1.66 1.64 1.63 1.63 1.62 1.61 1.61 1.60 1.59 1.58 1.57 1.56 1.55 1.55 1.54 1.54 1.52
3.55 3.54 3.53 3.52 3.51 3.50 3.50 3.49 3.48
N
CF3
3.01 1.33 1.02 1.00 1.01 0.96 4.05 1.05 1.03 2.08
10.5 10.0 9.5 9.0 8.5 8.0 7.5 7.0 6.5 6.0 5.5 5.0 4.5 4.0 3.5 3.0 2.5 2.0 1.5 1.0 0.5 0.0 −0.5 −1
f1 (ppm)

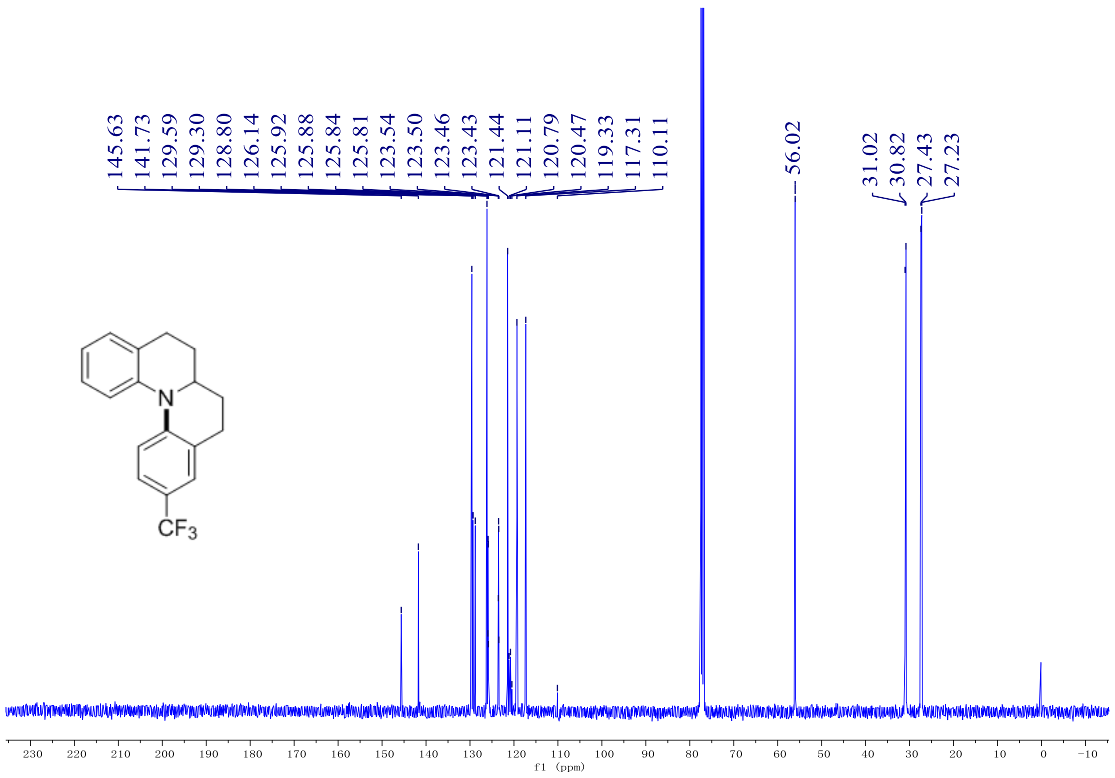
145.63
141.73
129.59
129.30
128.80
126.14
125.92
125.88
125.84
125.81
123.54
123.50
123.46
123.43
121.44
121.11
120.79
120.47
119.33
117.31
110.11
56.02
31.02
30.82
27.43
27.23
N
CF3
f1 (ppm)

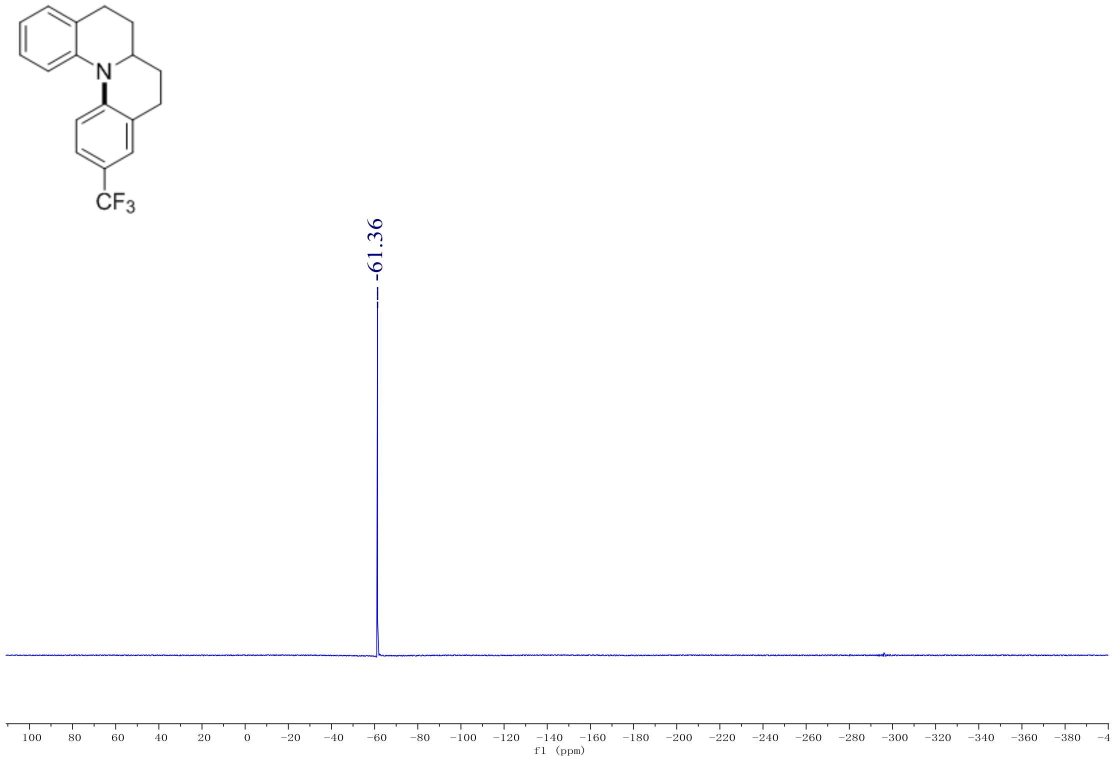
N
CF3
-61.36
f1 (ppm)

3d

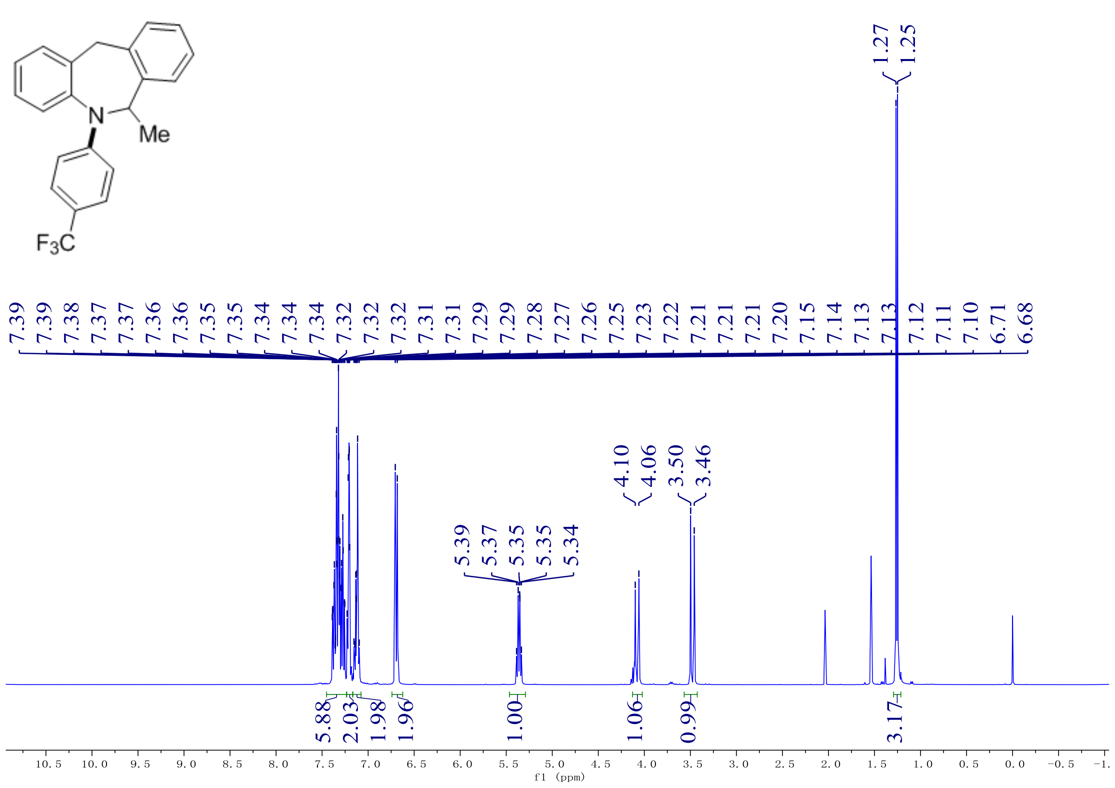
Me
N
F3C
7.39 7.39 7.38 7.37 7.37 7.36 7.36 7.35 7.35 7.34 7.34 7.34 7.32 7.32 7.32 7.31 7.31 7.29 7.29 7.28 7.27 7.26 7.25 7.23 7.22 7.21 7.21 7.21 7.20 7.15 7.14 7.13 7.13 7.12 7.11 7.10 6.71 6.68
5.39 5.37 5.35 5.35 5.34
4.10 4.06 3.50 3.46
1.27 1.25
5.88 2.03 1.98 1.96 1.00 1.06 0.99 3.17
f1 (ppm)

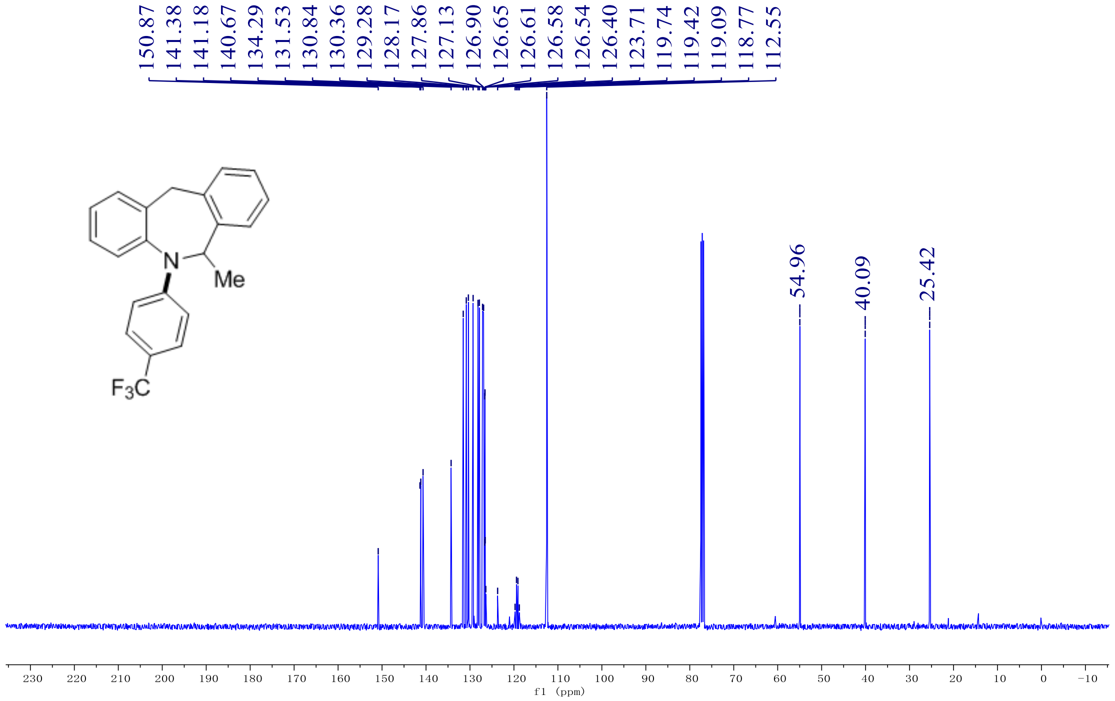
150.87 141.38 141.18 140.67 134.29 131.53 130.84 130.36 129.28 128.17 127.86 127.13 126.90 126.65 126.61 126.58 126.54 126.40 123.71 119.74 119.42 119.09 118.77 112.55
54.96 40.09 25.42
Me
N
F3C
f1 (ppm)

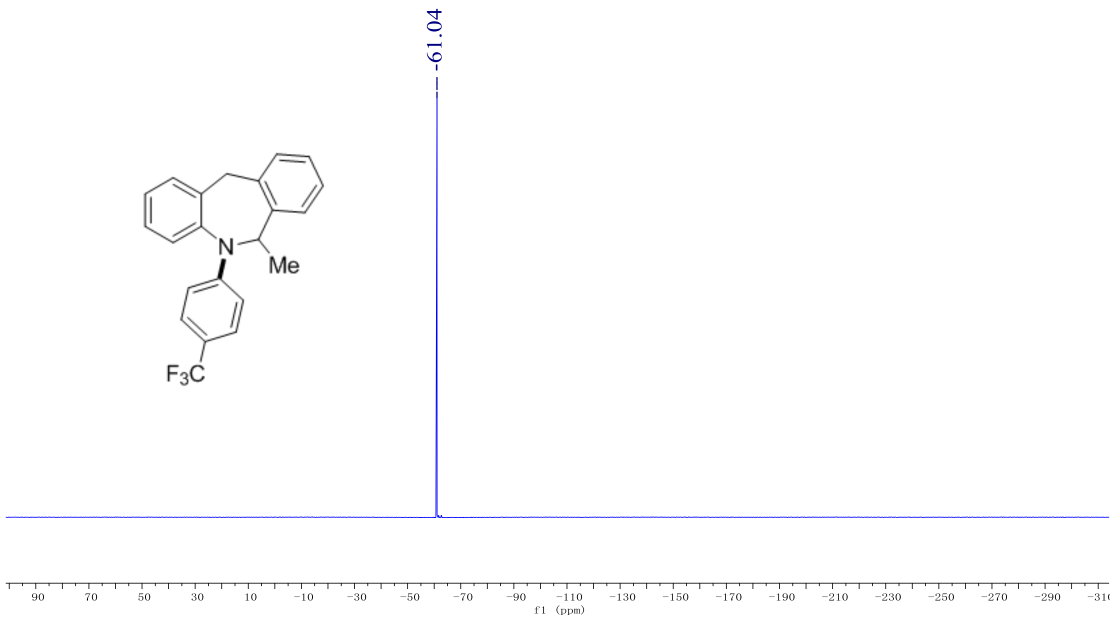
-61.04
Me
N
F3C
90 70 50 30 10 −10 −30 −50 −70 −90 −110 −130 −150 −170 −190 −210 −230 −250 −270 −290
f1 (ppm)

3e

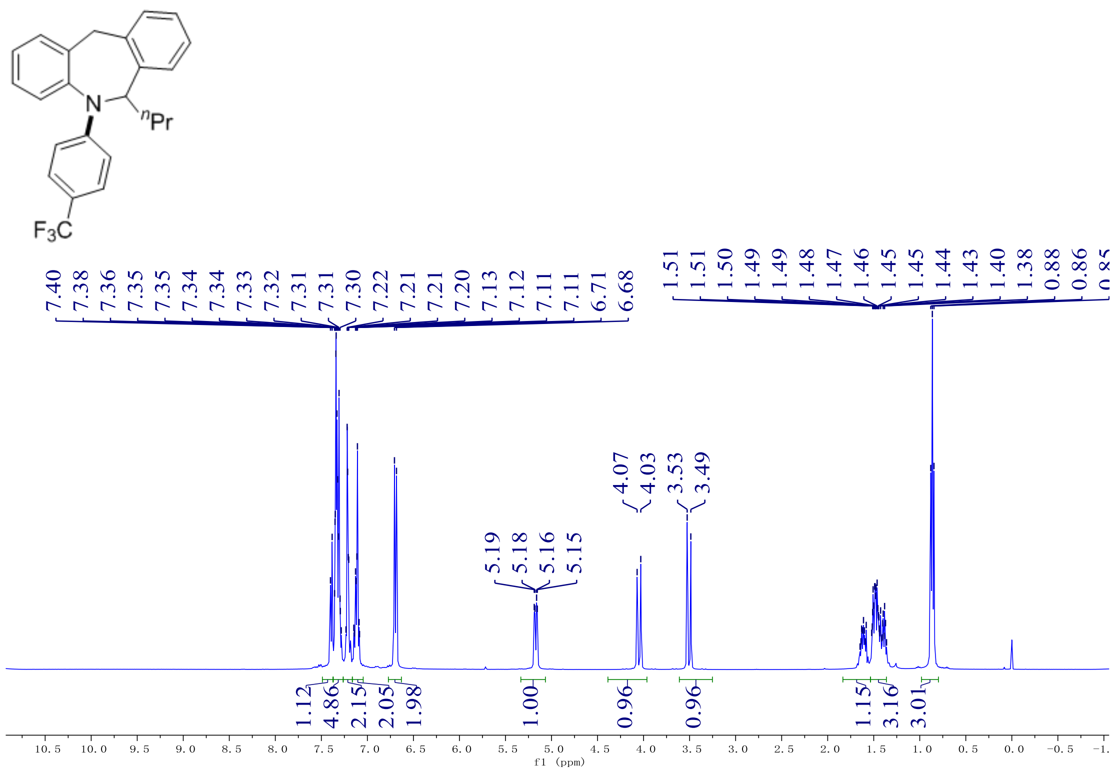

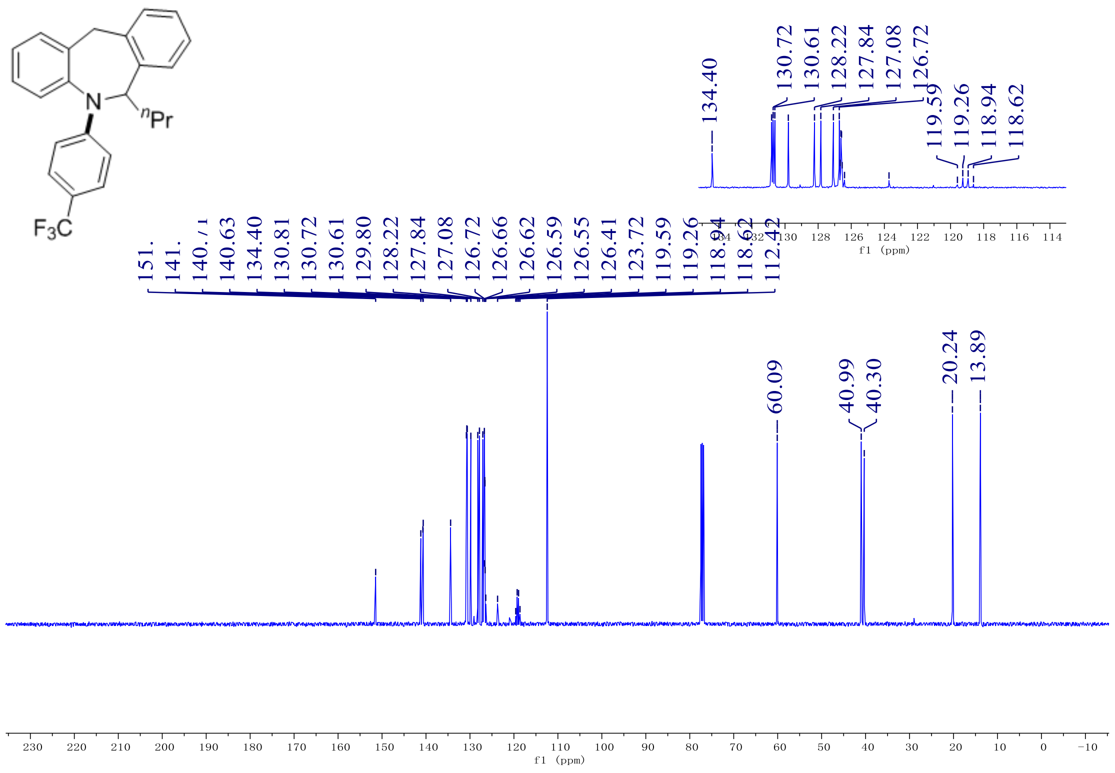

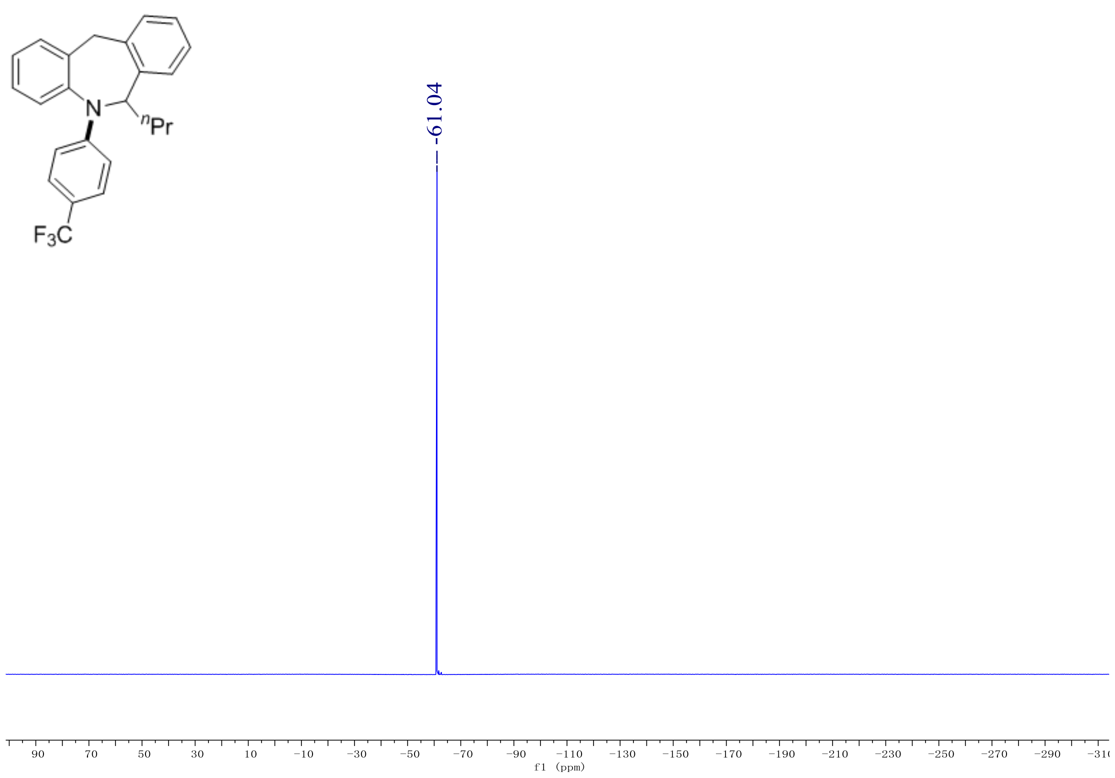

3f

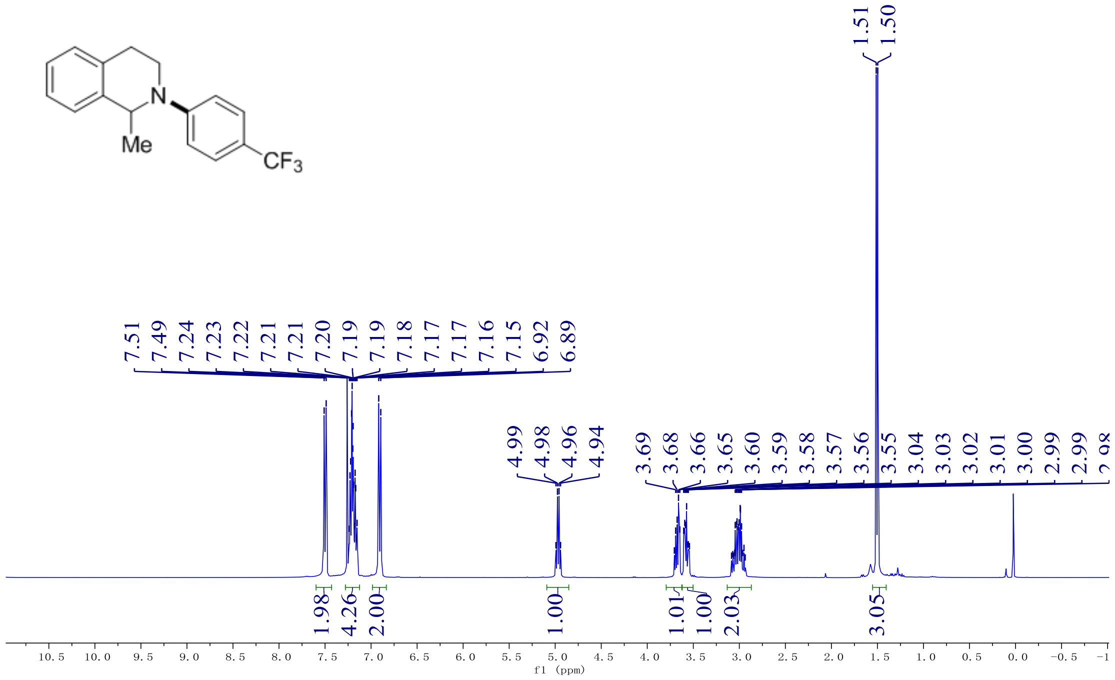

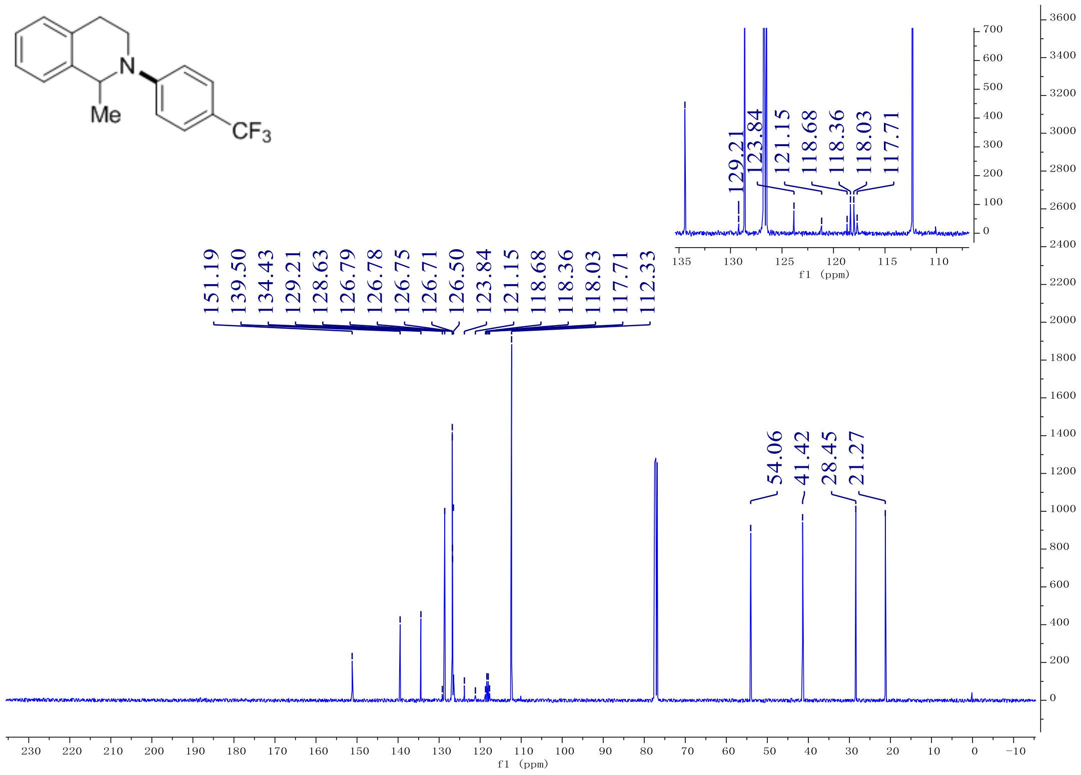
N
Me
CF3
151.19
139.50
134.43
129.21
128.63
126.79
126.78
126.75
126.71
126.50
123.84
121.15
118.68
118.36
118.03
117.71
112.33
54.06
41.42
28.45
21.27
f1 (ppm)

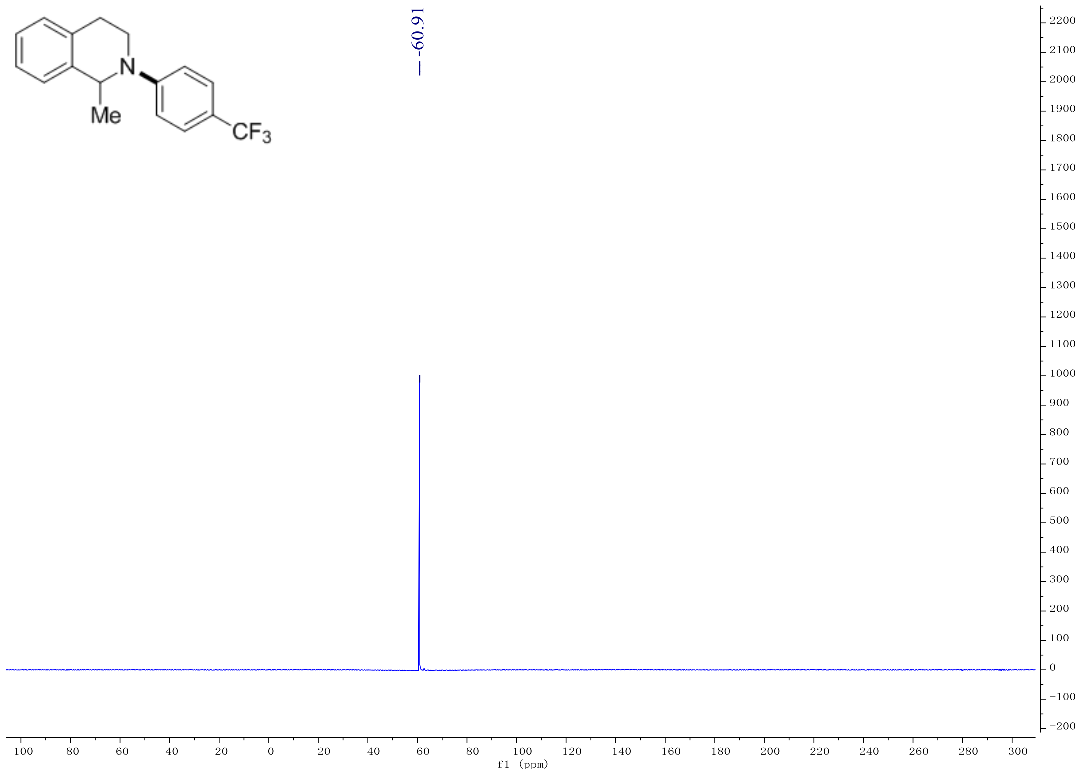
N
Me
CF3
-60.91
f1 (ppm)

3g

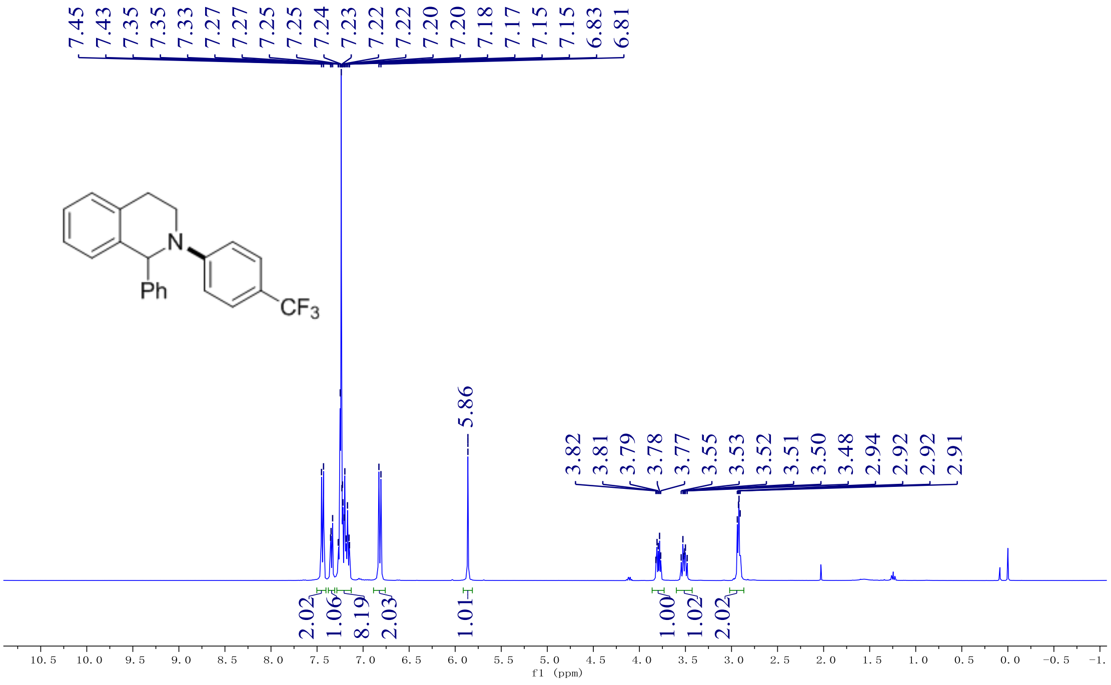
7.45
7.43
7.35
7.35
7.33
7.27
7.27
7.25
7.25
7.24
7.23
7.22
7.22
7.20
7.20
7.18
7.17
7.15
7.15
6.83
6.81
N
Ph
CF3
5.86
3.82
3.81
3.79
3.78
3.77
3.55
3.53
3.52
3.51
3.50
3.48
2.94
2.92
2.92
2.91
2.02
1.06
8.19
2.03
1.01
1.00
1.02
2.02
f1 (ppm)

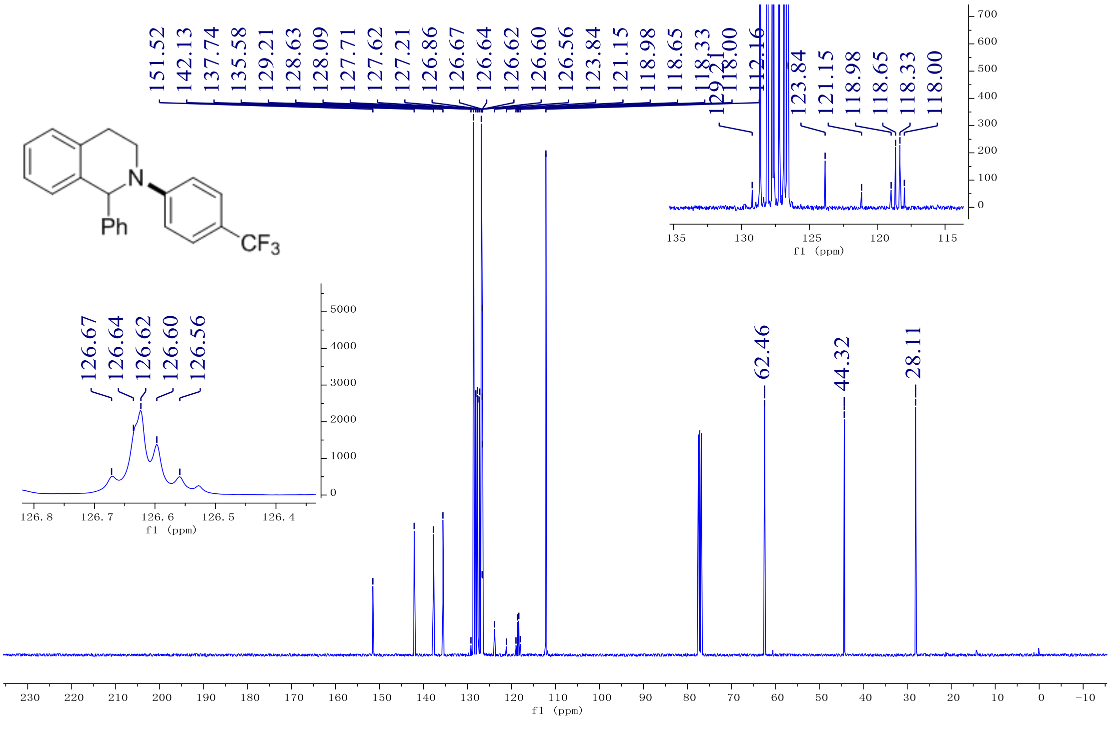
151.52
142.13
137.74
135.58
129.21
128.63
128.09
127.71
127.62
127.21
126.86
126.67
126.64
126.62
126.60
126.56
123.84
121.15
118.98
118.65
118.33
118.00
112.16
129.21
123.84
121.15
118.98
118.65
118.33
118.00
N
Ph
CF3
126.67
126.64
126.62
126.60
126.56
62.46
44.32
28.11
f1 (ppm)

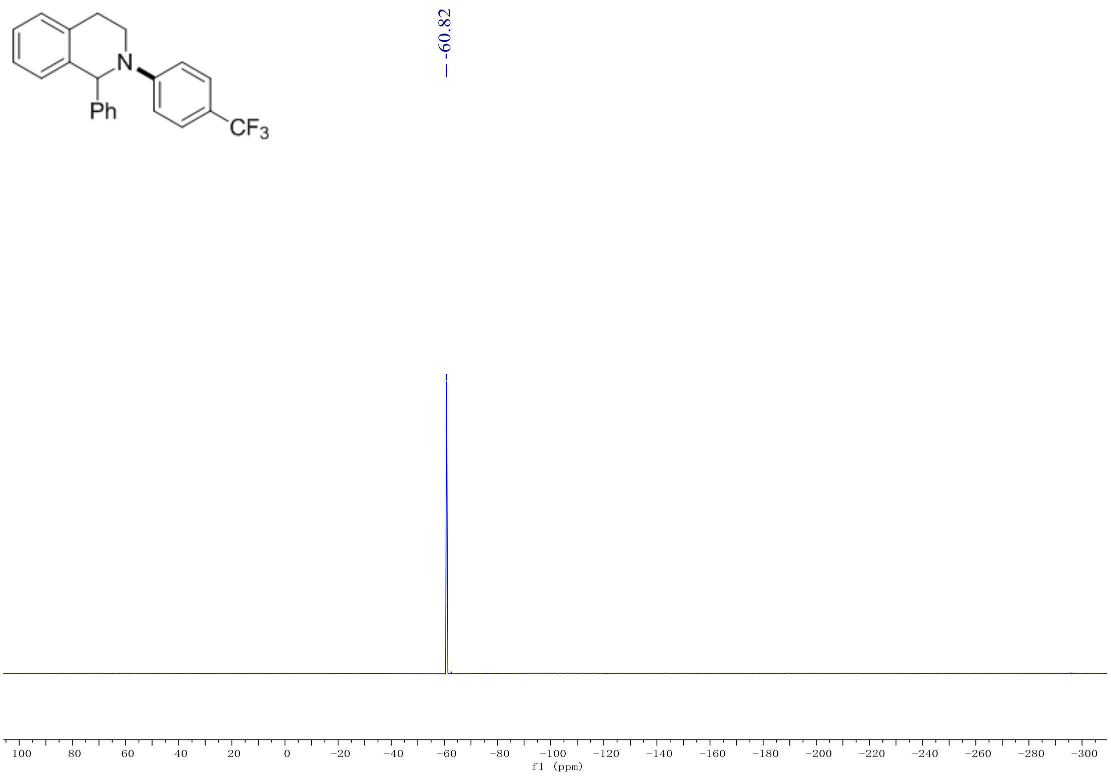

N
Ph
CF3
-60.82
100 80 60 40 20 0 −20 −40 −60 −80 −100 −120 −140 −160 −180 −200 −220 −240 −260 −280 −300
f1 (ppm)

3h

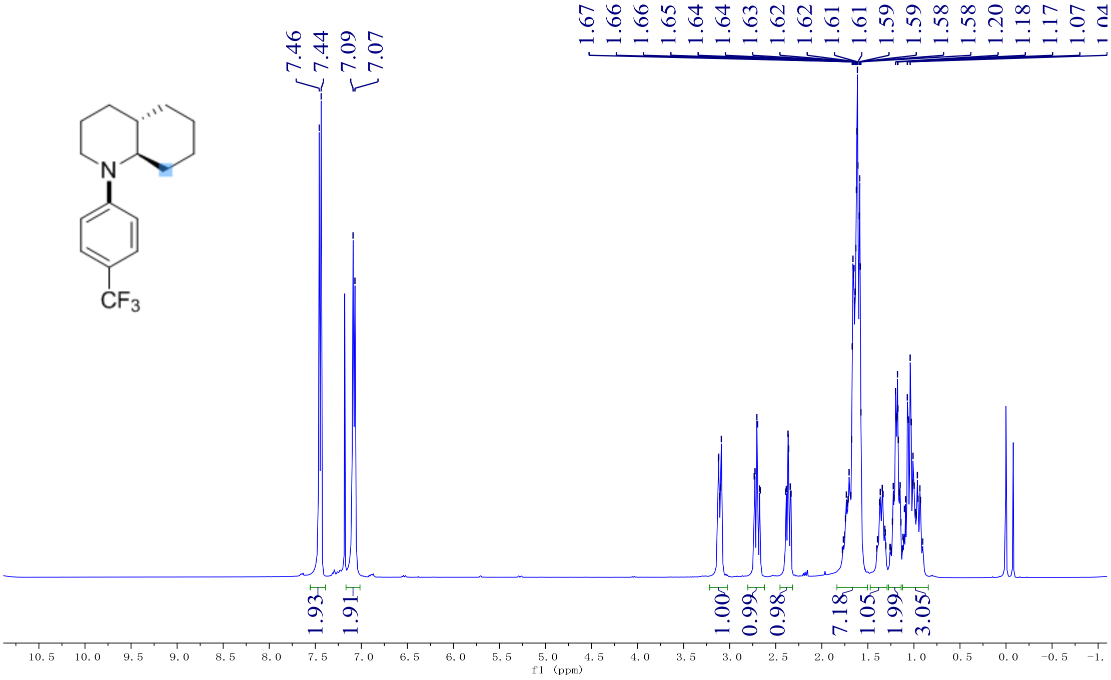

N
CF3
7.46
7.44
7.09
7.07
1.67
1.66
1.66
1.65
1.64
1.64
1.63
1.62
1.62
1.61
1.61
1.59
1.59
1.58
1.58
1.20
1.18
1.17
1.07
1.04
1.93
1.91
1.00
0.99
0.98
7.18
1.05
1.99
3.05
10.5 10.0 9.5 9.0 8.5 8.0 7.5 7.0 6.5 6.0 5.5 5.0 4.5 4.0 3.5 3.0 2.5 2.0 1.5 1.0 0.5 0.0 −0.5 −1.
f1 (ppm)

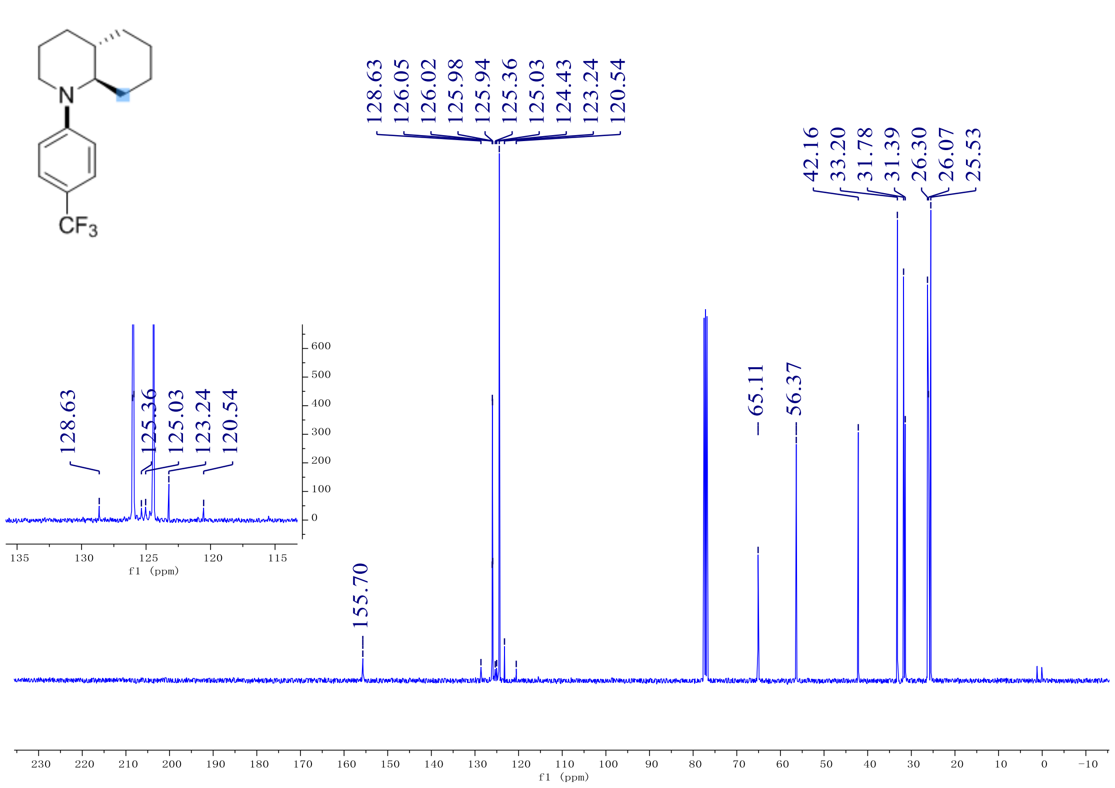
N
CF3
128.63
126.05
126.02
125.98
125.94
125.36
125.03
124.43
123.24
120.54
42.16
33.20
31.78
31.39
26.30
26.07
25.53
65.11
56.37
155.70
f1 (ppm)

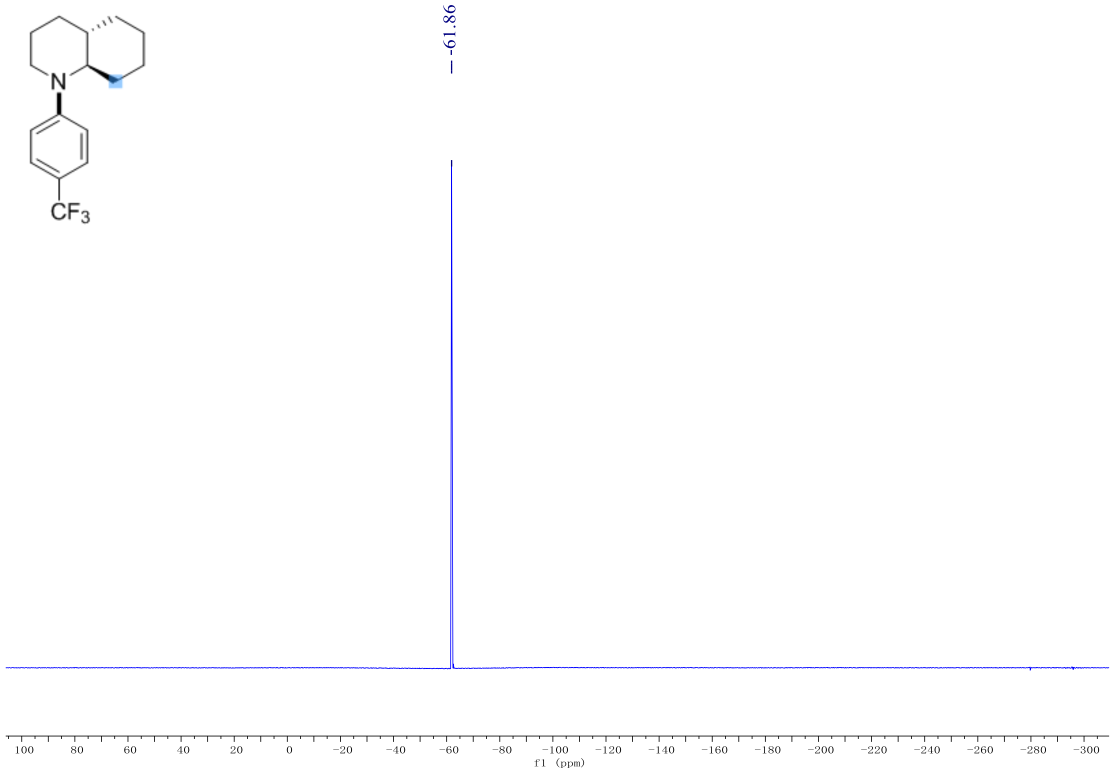
N
CF3
-61.86
f1 (ppm)

3i

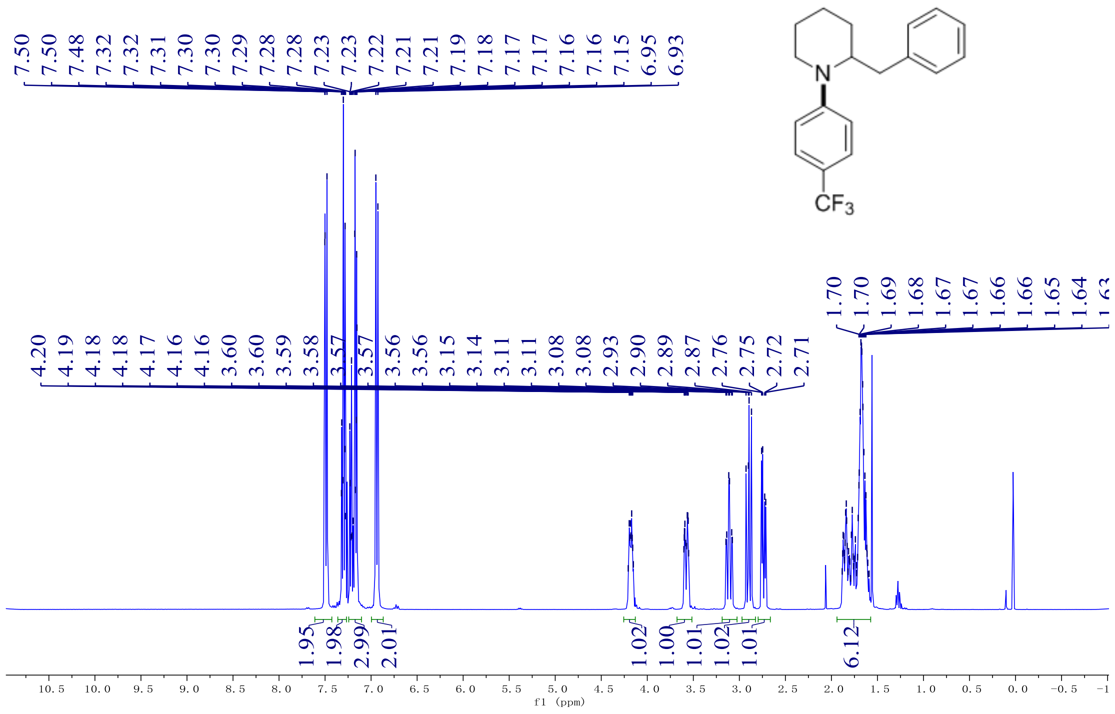

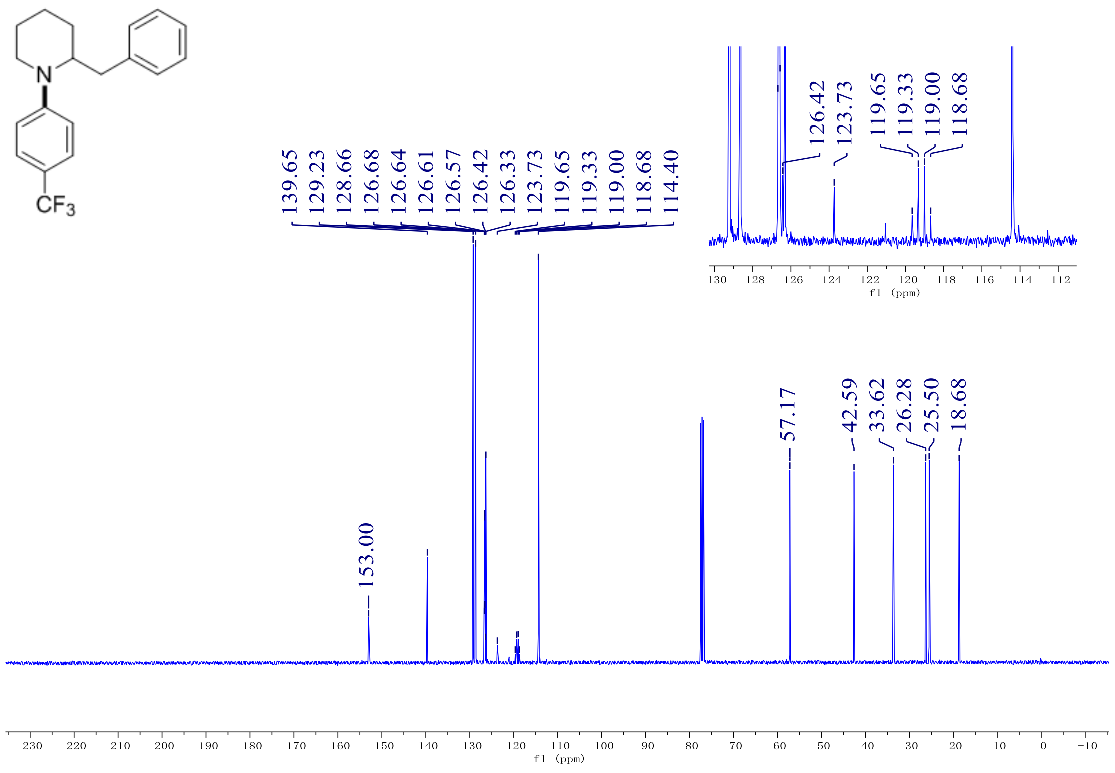

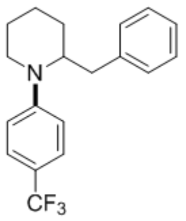
N
CF3

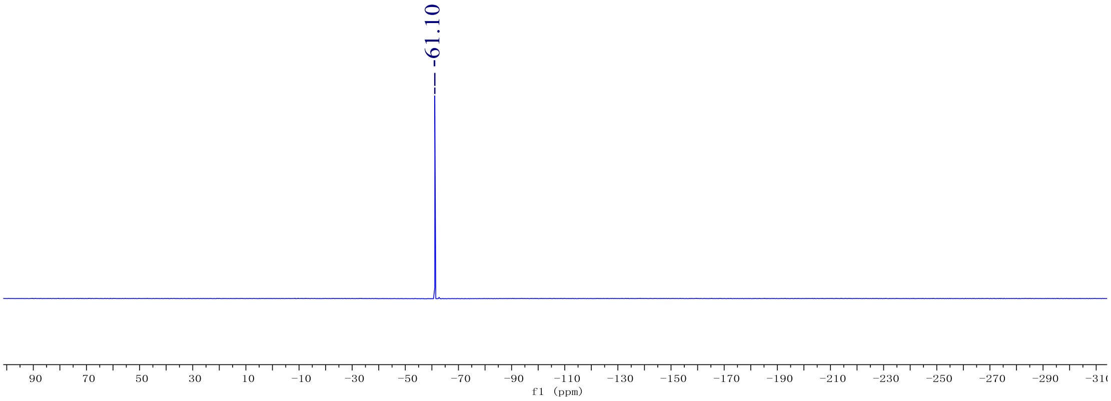
-61.10
f1 (ppm)

3j

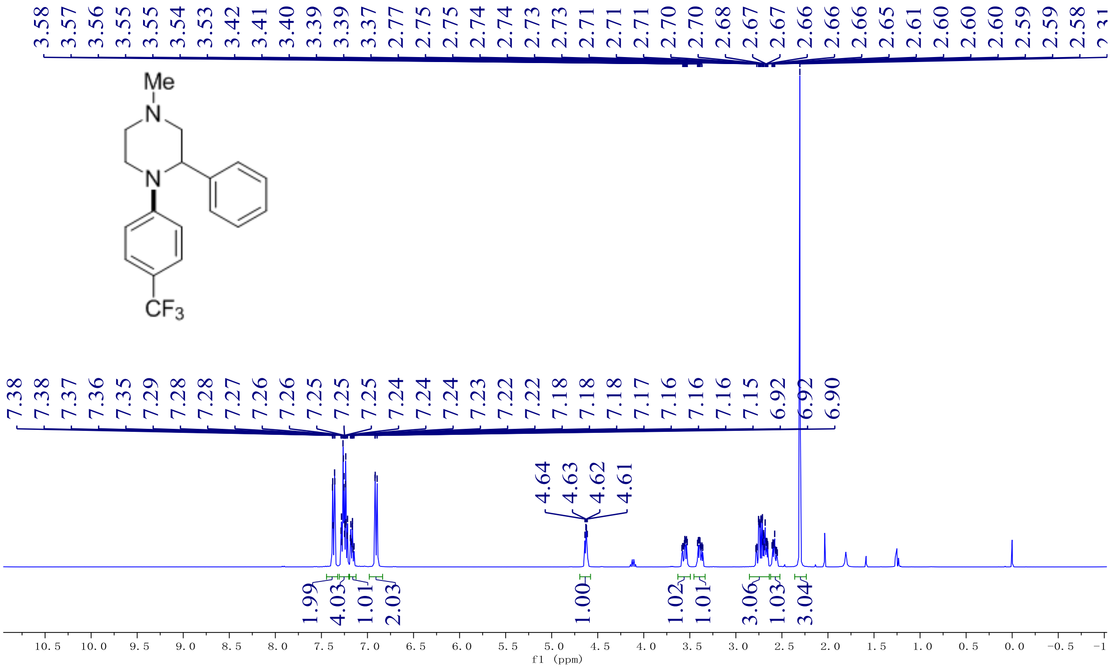
3.58 3.57 3.56 3.55 3.55 3.54 3.53 3.42 3.41 3.40 3.39 3.39 3.37 2.77 2.75 2.75 2.74 2.74 2.73 2.73 2.71 2.71 2.71 2.70 2.70 2.68 2.67 2.67 2.66 2.66 2.66 2.65 2.61 2.60 2.60 2.60 2.59 2.59 2.58
Me
N
N
CF3
7.38 7.38 7.37 7.36 7.35 7.29 7.28 7.28 7.27 7.26 7.26 7.25 7.25 7.25 7.24 7.24 7.24 7.23 7.22 7.22 7.18 7.18 7.18 7.17 7.16 7.16 7.16 7.15 6.92 6.92 6.90
4.64 4.63 4.62 4.61
1.99 4.03 1.01 2.03
1.00
1.02 1.01
3.06 1.03 3.04
f1 (ppm)

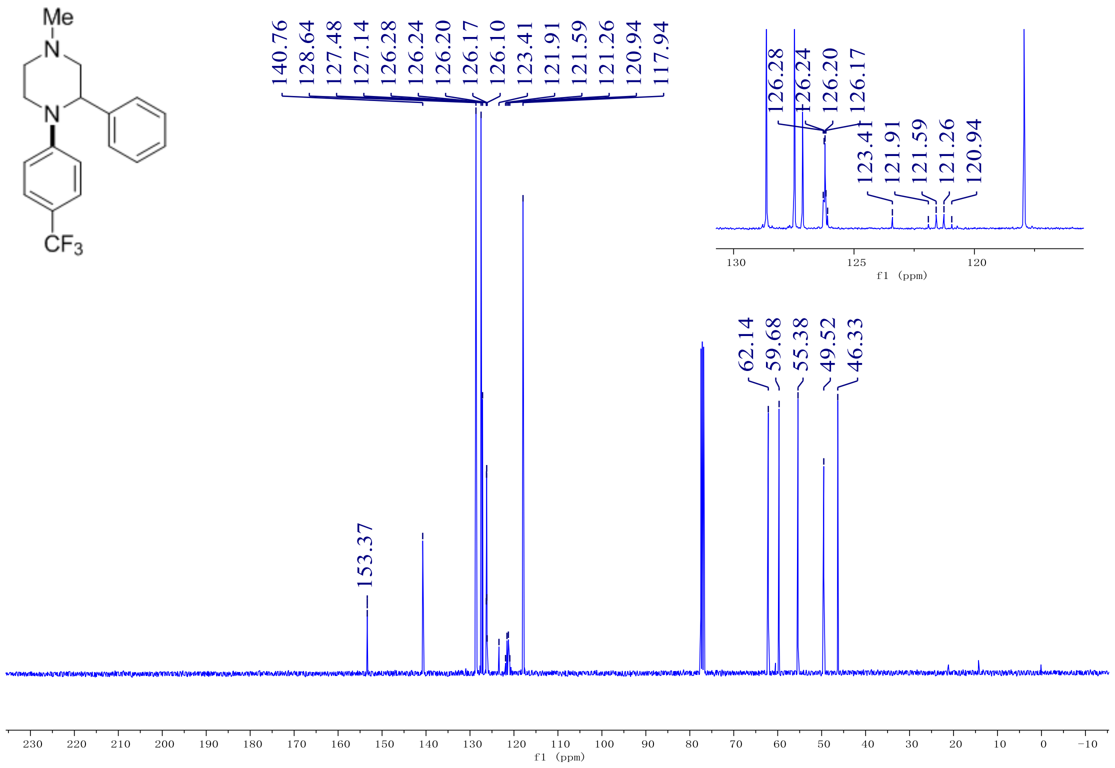
Me
N
N
CF3
140.76
128.64
127.48
127.14
126.28
126.24
126.20
126.17
126.10
123.41
121.91
121.59
121.26
120.94
117.94
153.37
62.14
59.68
55.38
49.52
46.33
f1 (ppm)

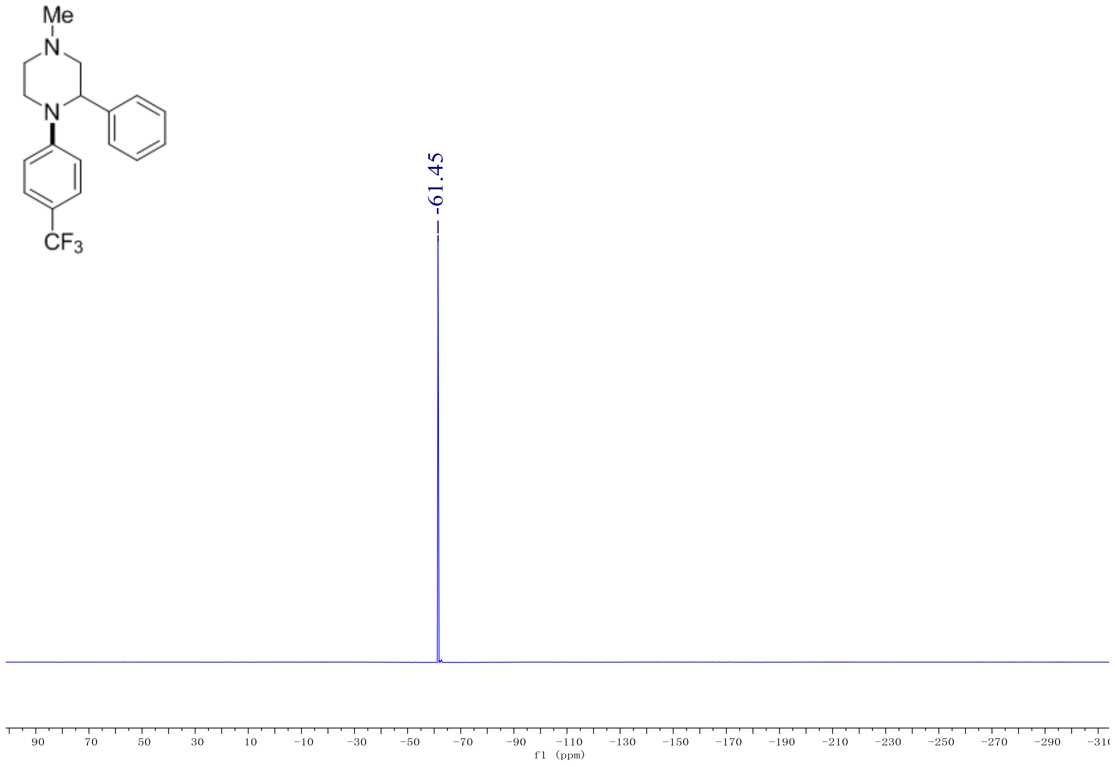
Me
N
N
CF3
-61.45
f1 (ppm)

3k

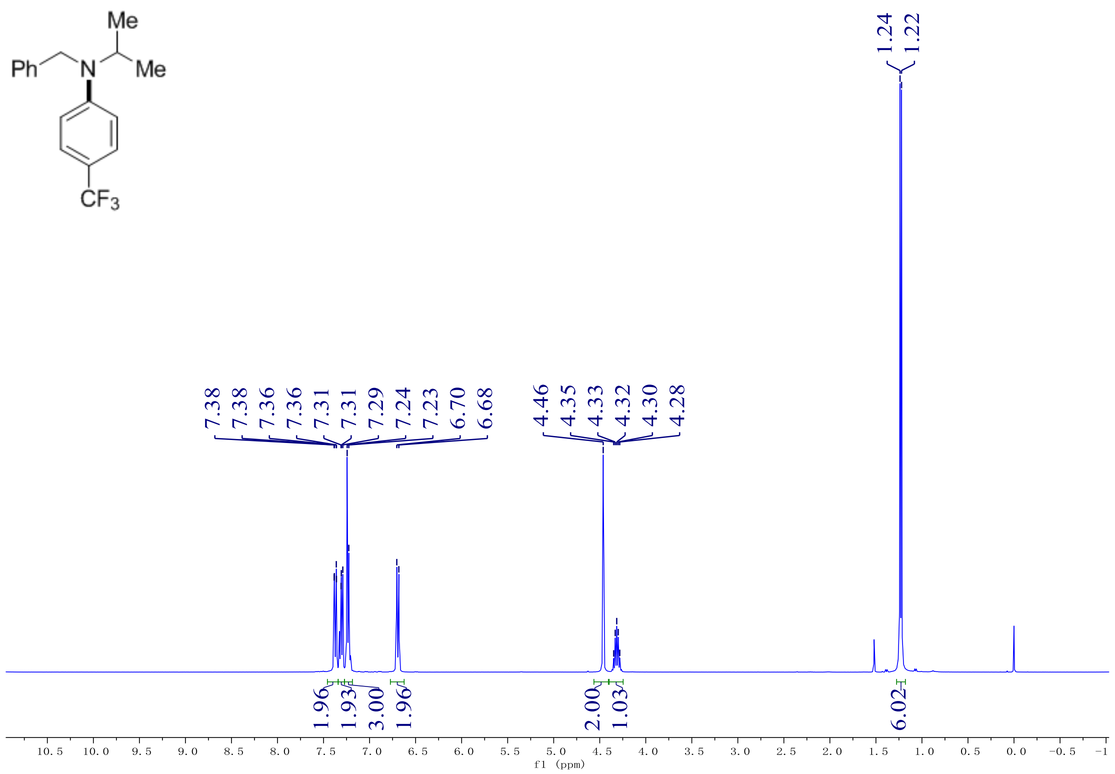
Me
Ph
N
Me
CF3
7.38
7.38
7.36
7.36
7.31
7.31
7.29
7.24
7.23
6.70
6.68
4.46
4.35
4.33
4.32
4.30
4.28
1.24
1.22
1.96
1.93
3.00
1.96
2.00
1.03
6.02
f1 (ppm)

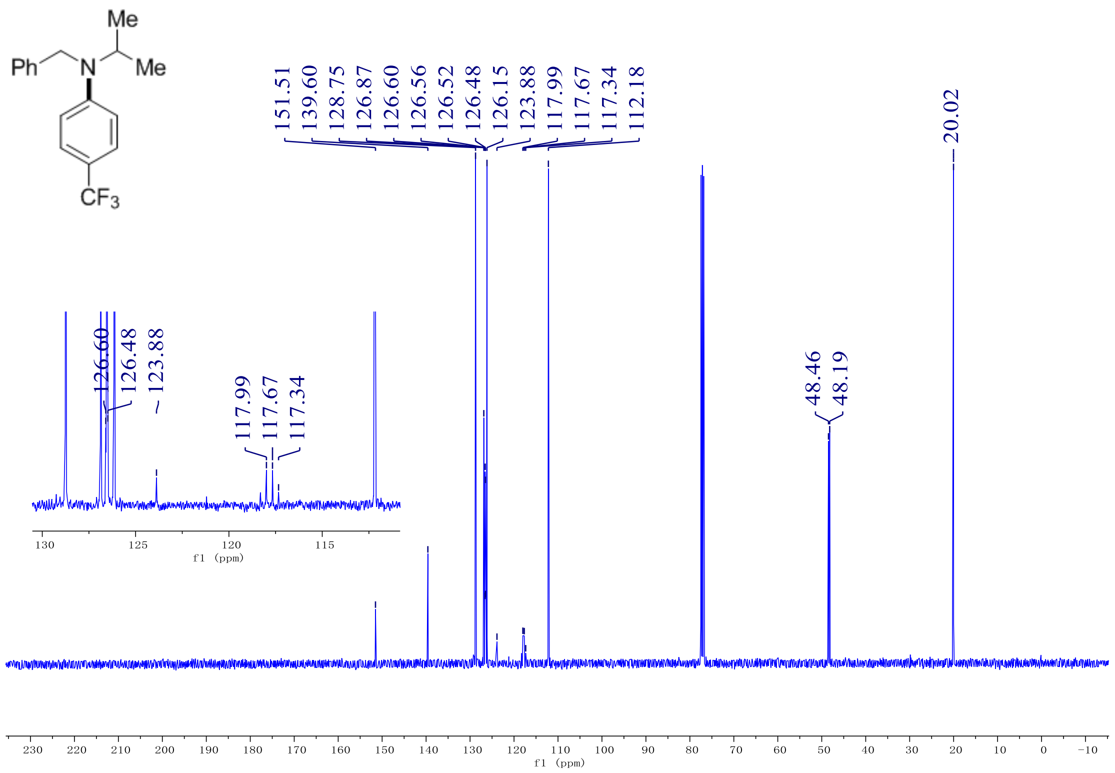
Me
Ph
N
Me
CF3
151.51
139.60
128.75
126.87
126.60
126.56
126.52
126.48
126.15
123.88
117.99
117.67
117.34
112.18
20.02
48.46
48.19
126.60
126.48
123.88
117.99
117.67
117.34
f1 (ppm)
f1 (ppm)

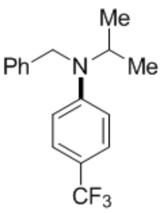

Me
Ph
N
Me
CF3

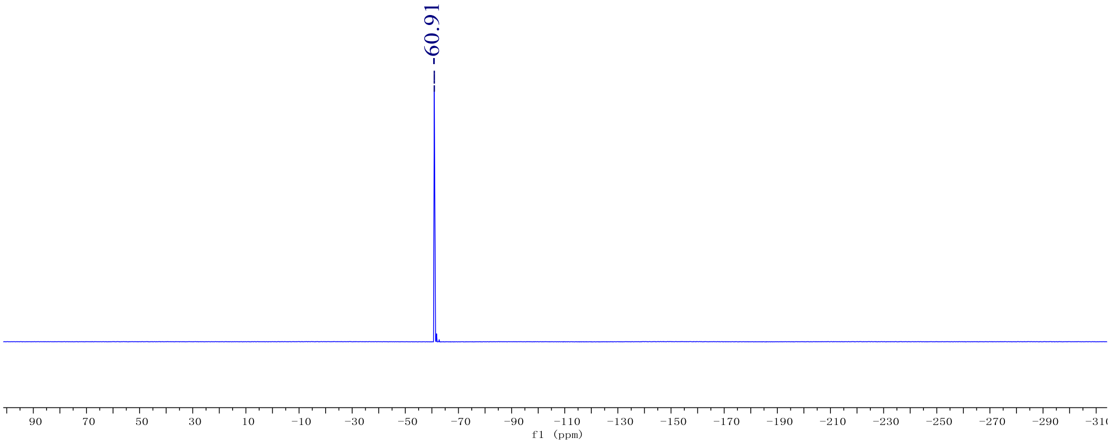

-60.91
90 70 50 30 10 −10 −30 −50 −70 −90 −110 −130 −150 −170 −190 −210 −230 −250 −270 −290
f1 (ppm)

3l

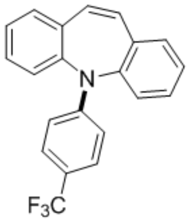

N
F3C

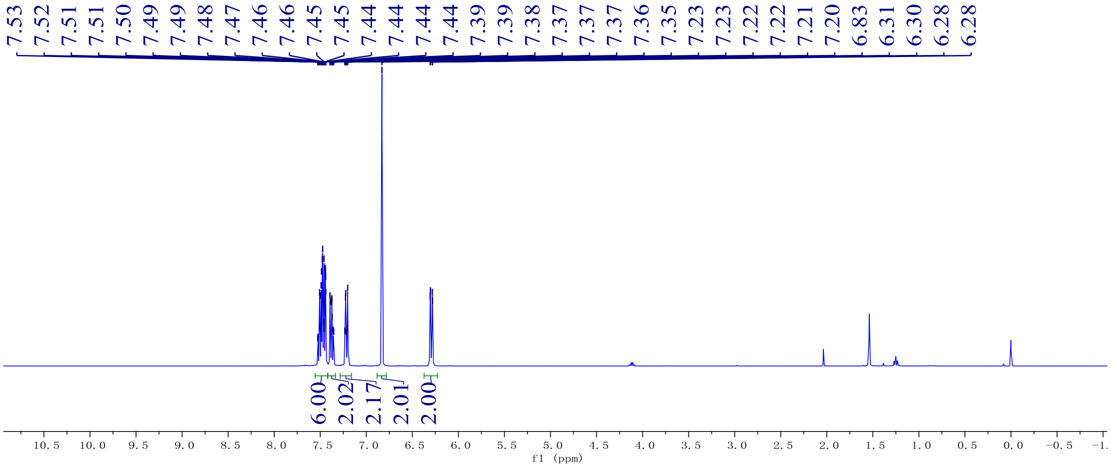

7.53 7.52 7.51 7.51 7.50 7.49 7.49 7.48 7.47 7.46 7.46 7.45 7.45 7.44 7.44 7.44 7.44 7.39 7.39 7.38 7.37 7.37 7.37 7.36 7.35 7.23 7.23 7.22 7.22 7.21 7.20 6.83 6.31 6.30 6.28 6.28
6.00 2.02 2.17 2.01 2.00
10.5 10.0 9.5 9.0 8.5 8.0 7.5 7.0 6.5 6.0 5.5 5.0 4.5 4.0 3.5 3.0 2.5 2.0 1.5 1.0 0.5 0.0 −0.5
f1 (ppm)

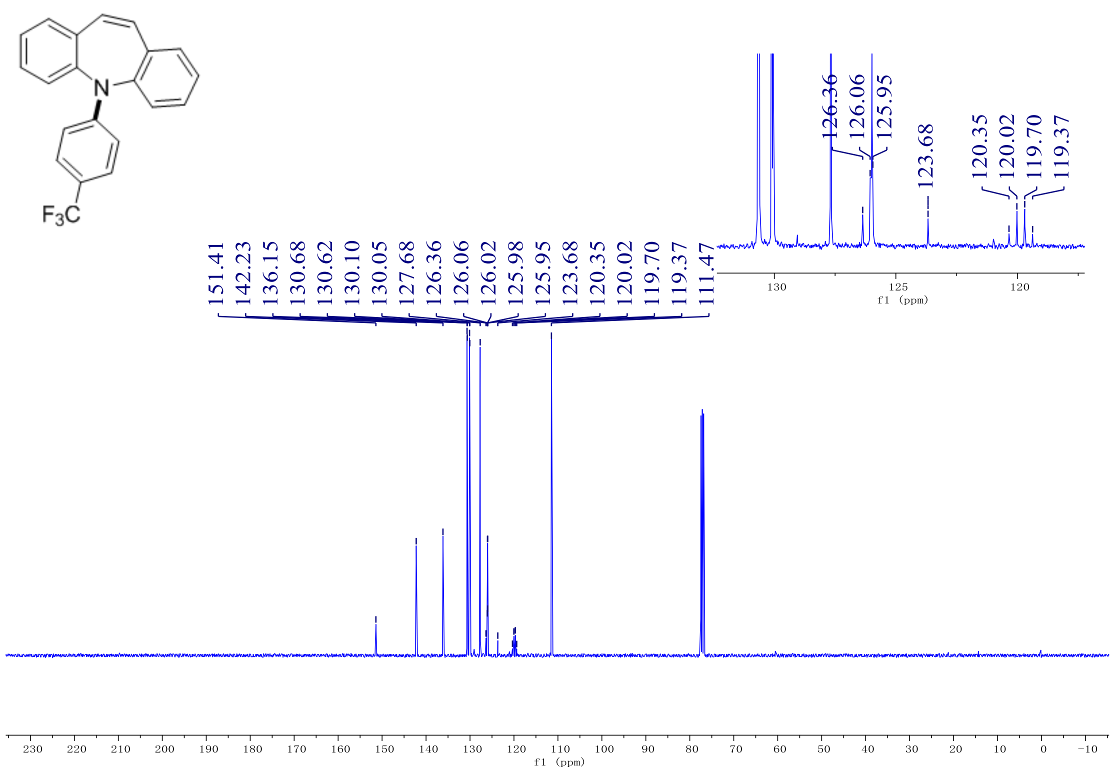
N
F3C
151.41
142.23
136.15
130.68
130.62
130.10
130.05
127.68
126.36
126.06
126.02
125.98
125.95
123.68
120.35
120.02
119.70
119.37
111.47
126.36
126.06
125.95
123.68
120.35
120.02
119.70
119.37
f1 (ppm)
f1 (ppm)

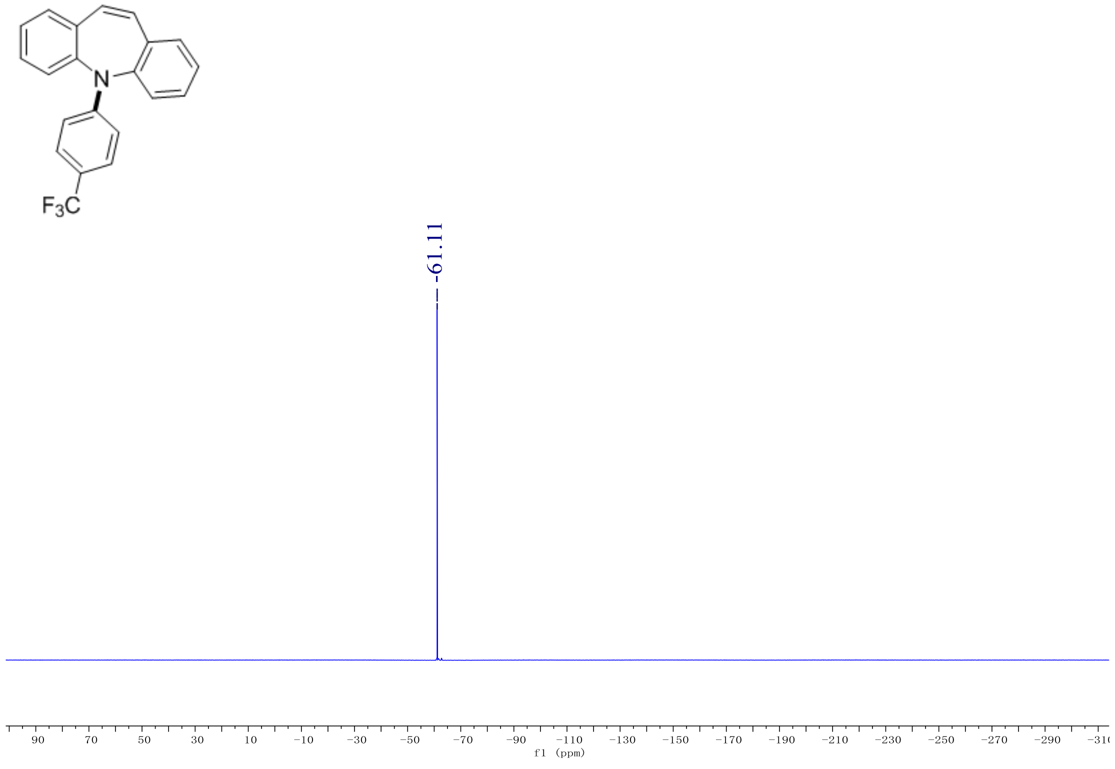
N
F3C
-61.11
f1 (ppm)

3m

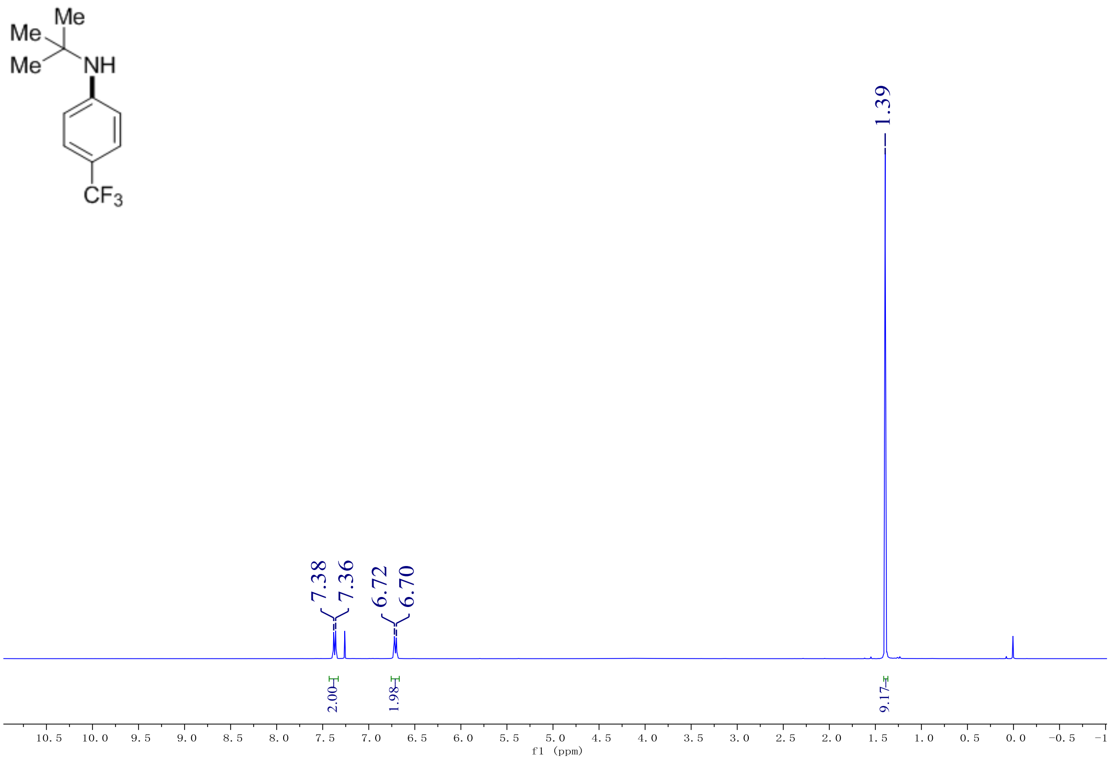
Me
Me
Me
NH
CF3
1.39
7.38
7.36
6.72
6.70
2.00
1.98
9.17
10.5 10.0 9.5 9.0 8.5 8.0 7.5 7.0 6.5 6.0 5.5 5.0 4.5 4.0 3.5 3.0 2.5 2.0 1.5 1.0 0.5 0.0 -0.5 -1
f1 (ppm)

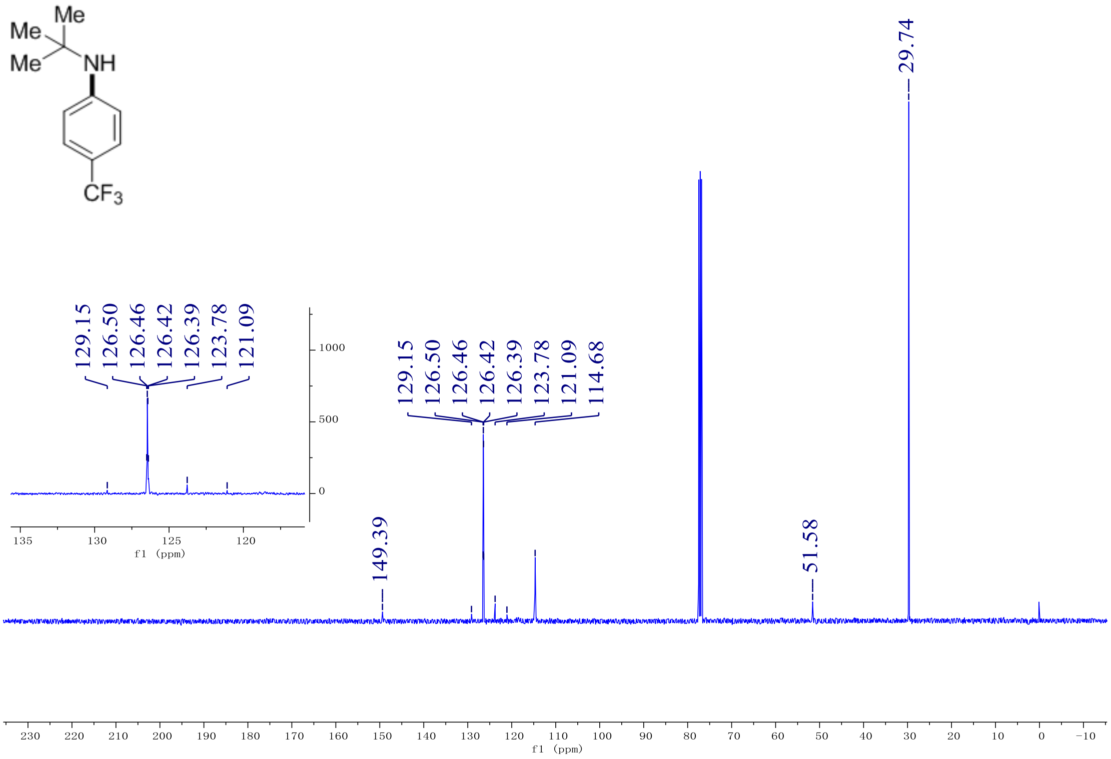
Me
Me
Me
NH
CF3
29.74
129.15
126.50
126.46
126.42
126.39
123.78
121.09
1000
500
0
135 130 125 120
f1 (ppm)
129.15
126.50
126.46
126.42
126.39
123.78
121.09
114.68
149.39
51.58
230 220 210 200 190 180 170 160 150 140 130 120 110 100 90 80 70 60 50 40 30 20 10 0 -10
f1 (ppm)

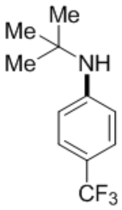

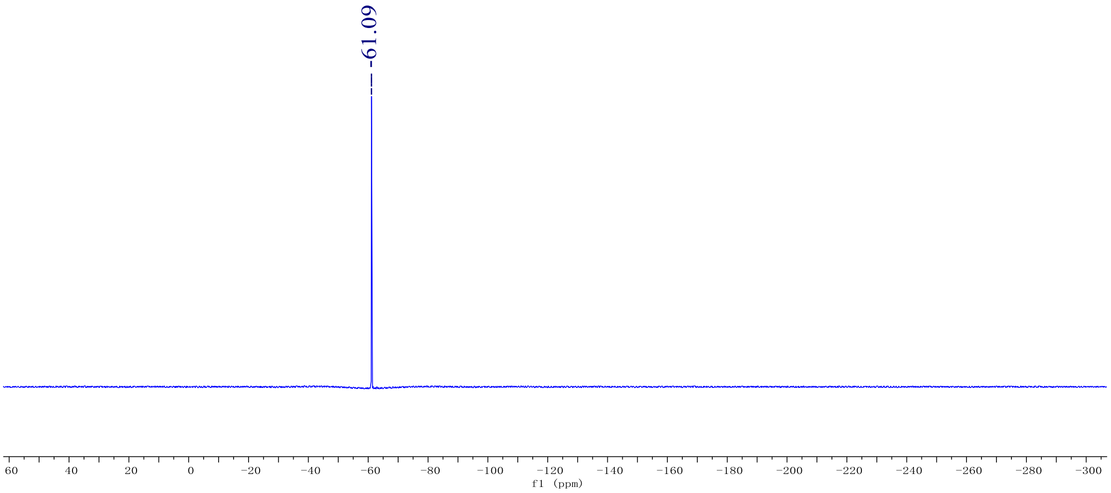

3n

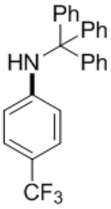

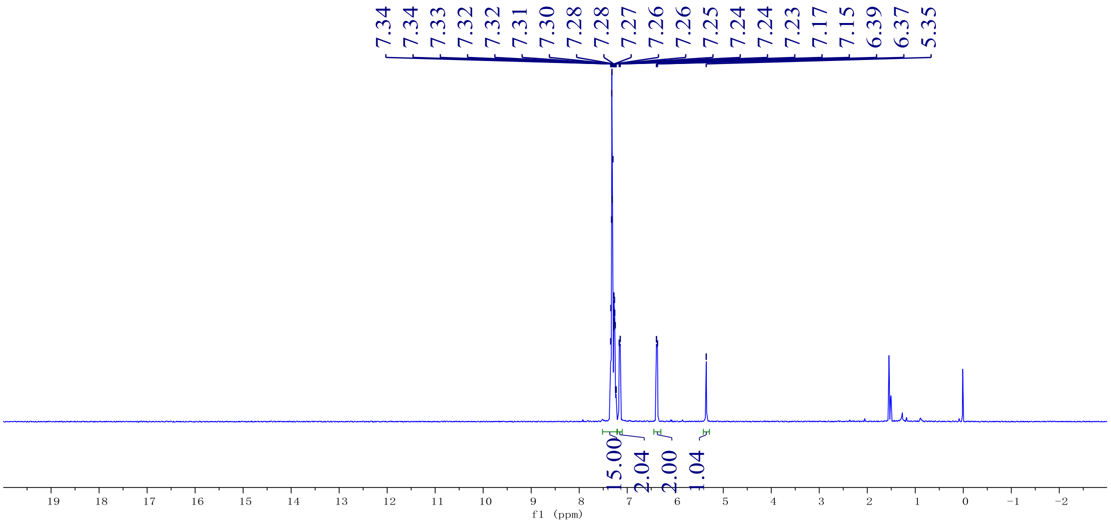

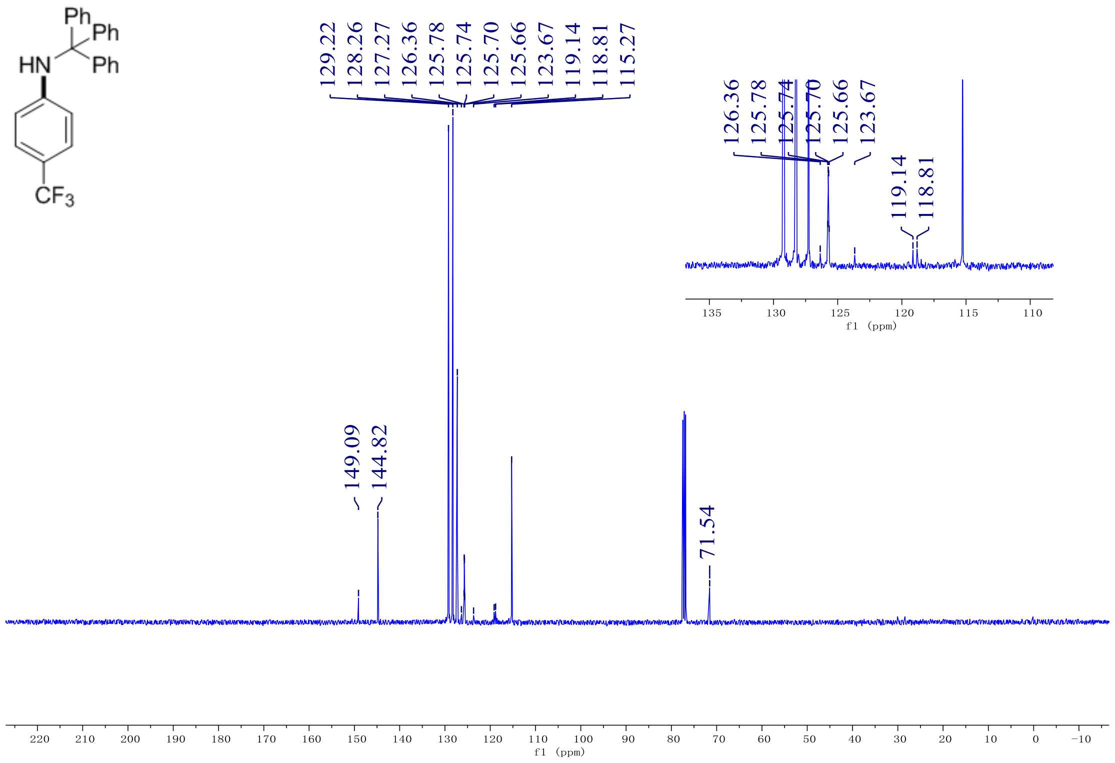
Ph
Ph
Ph
HN
CF3
129.22
128.26
127.27
126.36
125.78
125.74
125.70
125.66
123.67
119.14
118.81
115.27
149.09
144.82
71.54
f1 (ppm)

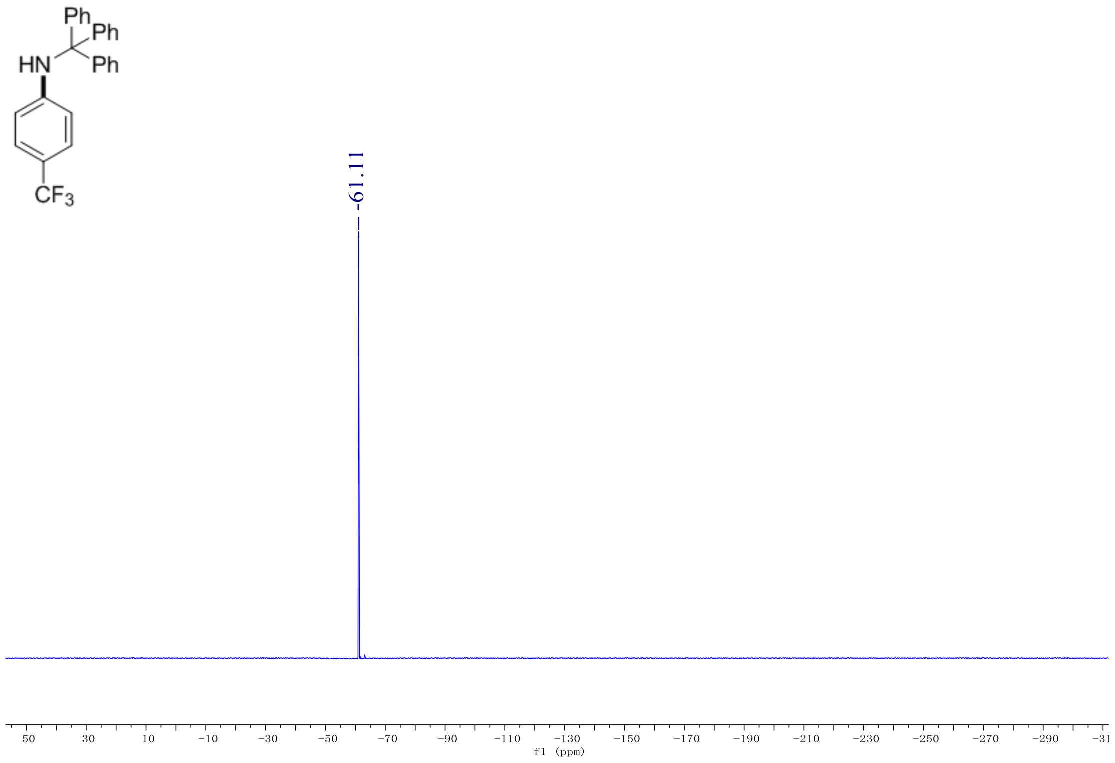
Ph
Ph
Ph
HN
CF3
-61.11
f1 (ppm)

3o

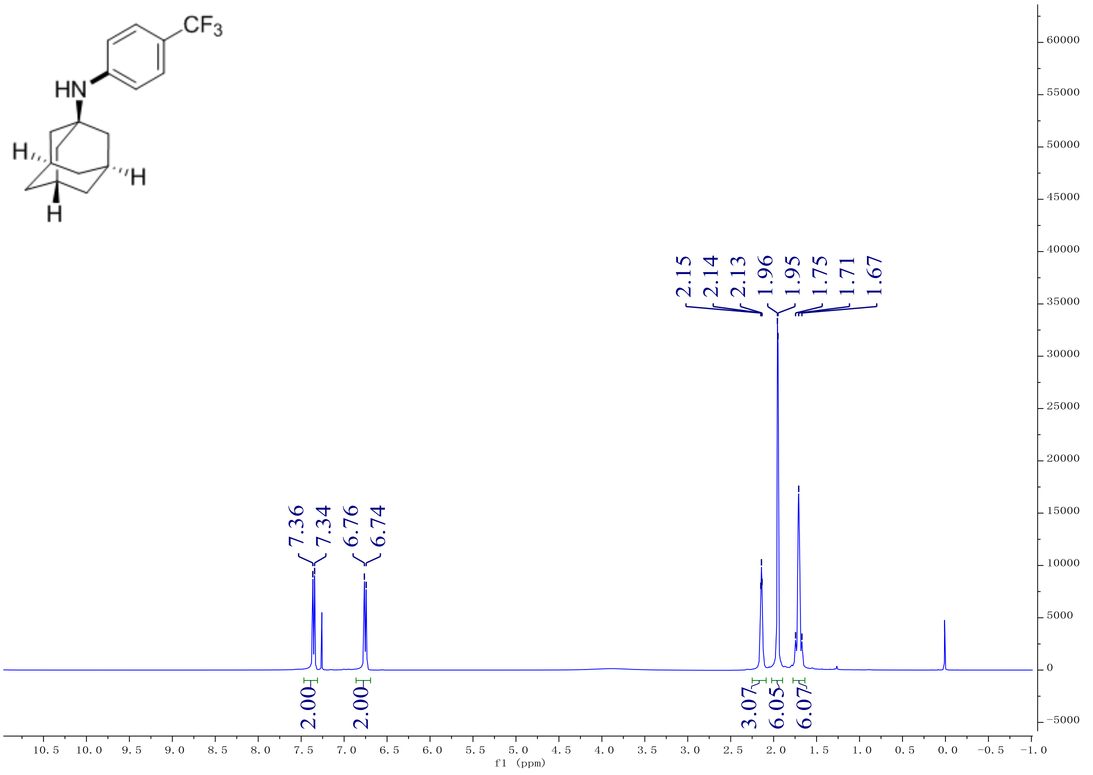
CF3
HN
H
H
H
7.36
7.34
6.76
6.74
2.15
2.14
2.13
1.96
1.95
1.75
1.71
1.67
2.00
2.00
3.07
6.05
6.07
f1 (ppm)

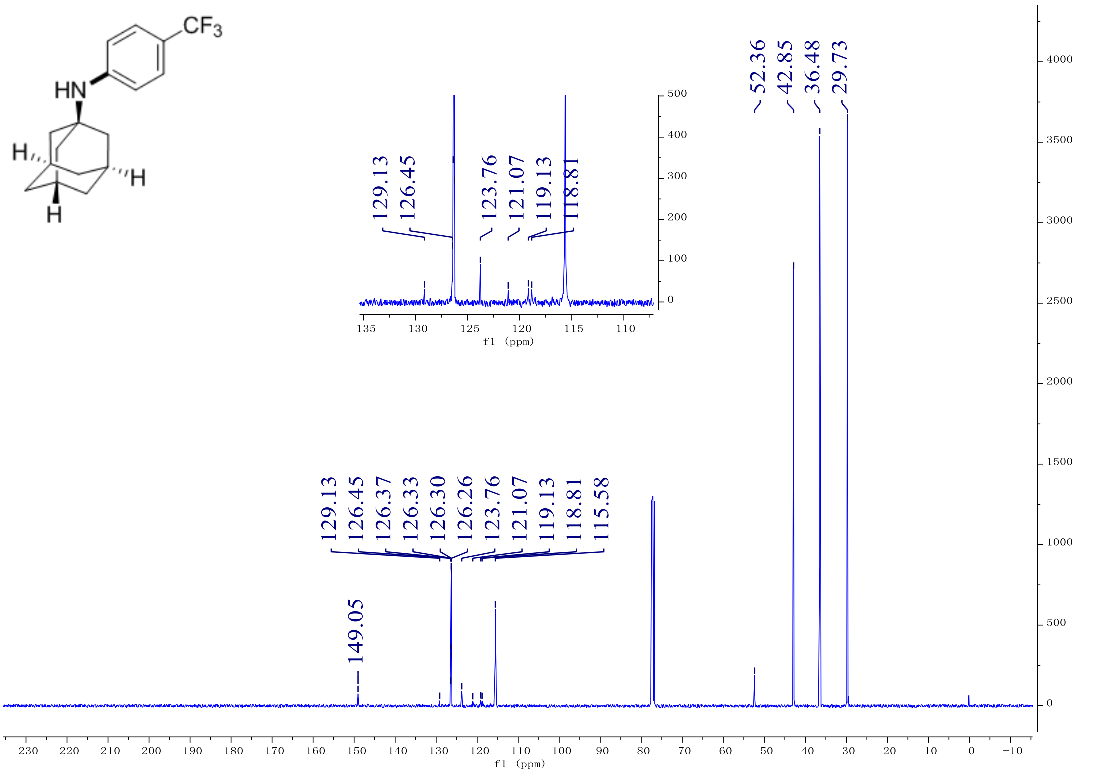
CF3
HN
H
H
H
52.36
42.85
36.48
29.73
129.13
126.45
123.76
121.07
119.13
118.81
129.13
126.45
126.37
126.33
126.30
126.26
123.76
121.07
119.13
118.81
115.58
149.05
f1 (ppm)

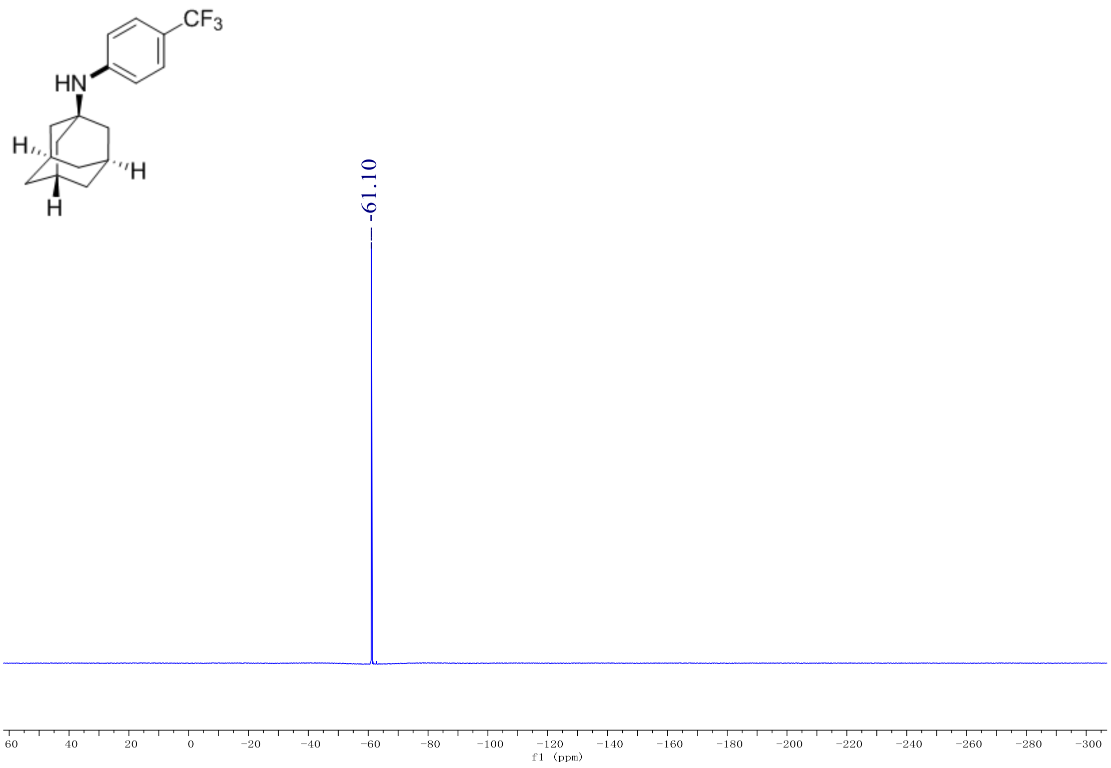
CF3
HN
H
H
H
-61.10
f1 (ppm)

3p

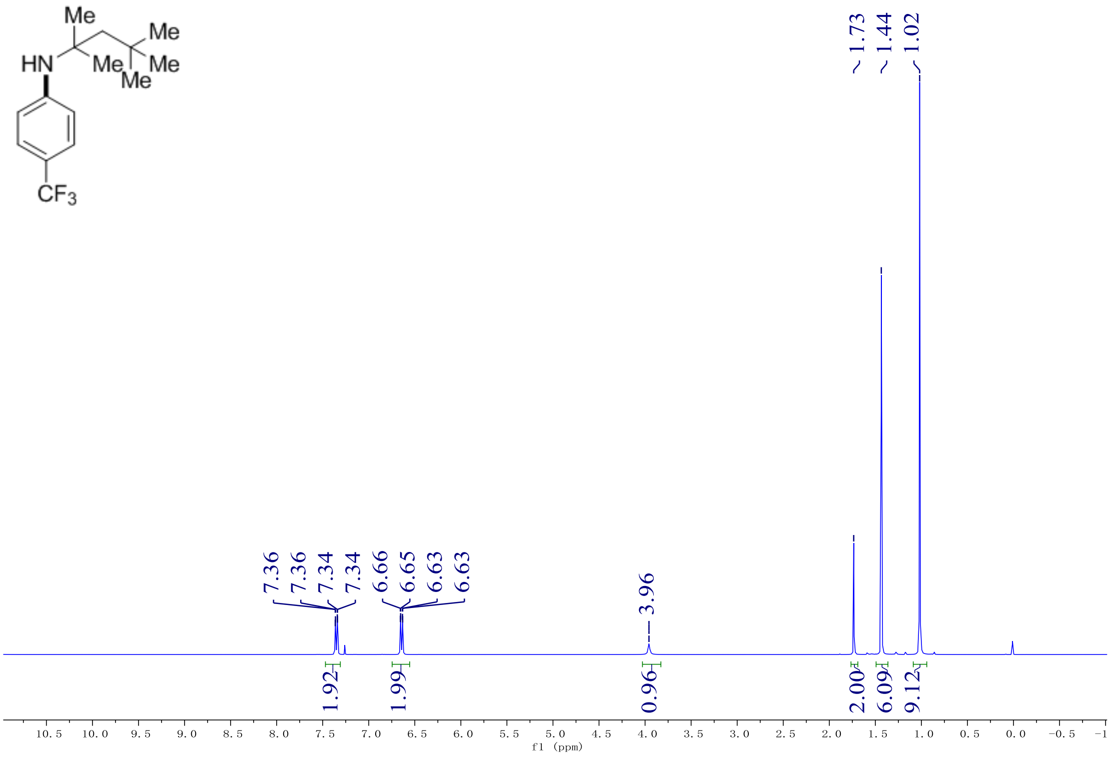
Me
Me
HN
Me
Me
Me
CF3
1.73
1.44
1.02
7.36
7.36
7.34
7.34
6.66
6.65
6.63
6.63
3.96
1.92
1.99
0.96
2.00
6.09
9.12
f1 (ppm)

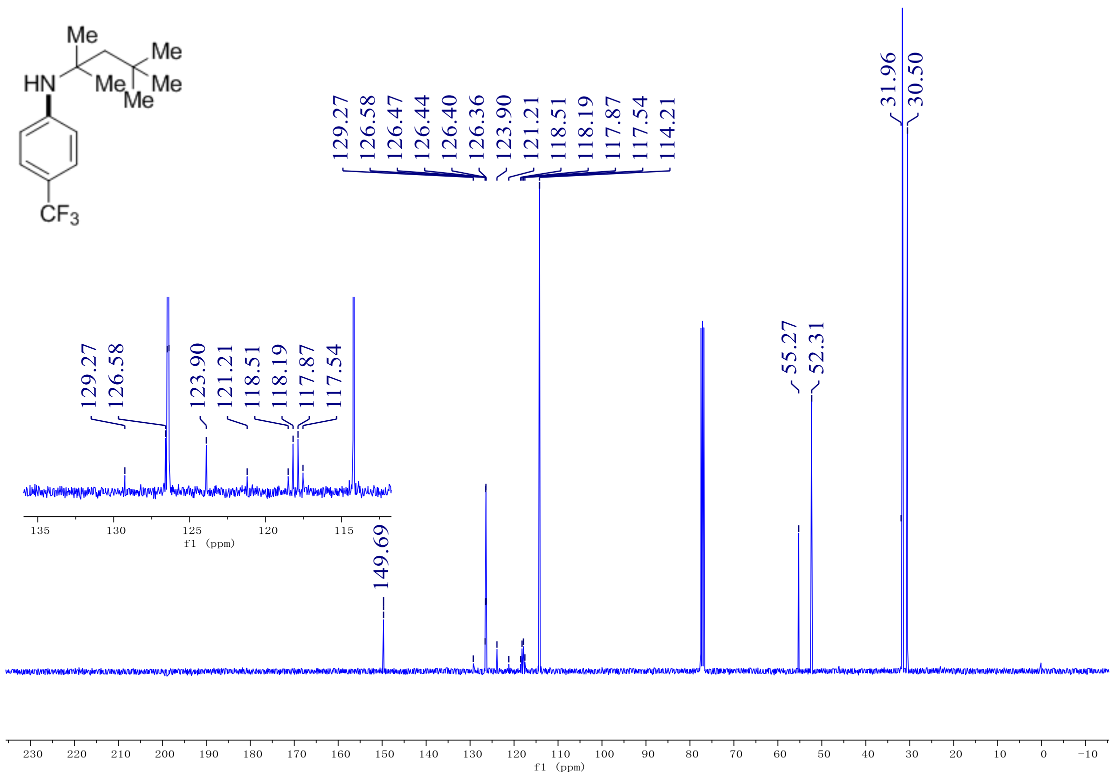
Me
HN
Me
Me
Me
Me
CF3
129.27
126.58
126.47
126.44
126.40
126.36
123.90
121.21
118.51
118.19
117.87
117.54
114.21
31.96
30.50
55.27
52.31
149.69
f1 (ppm)

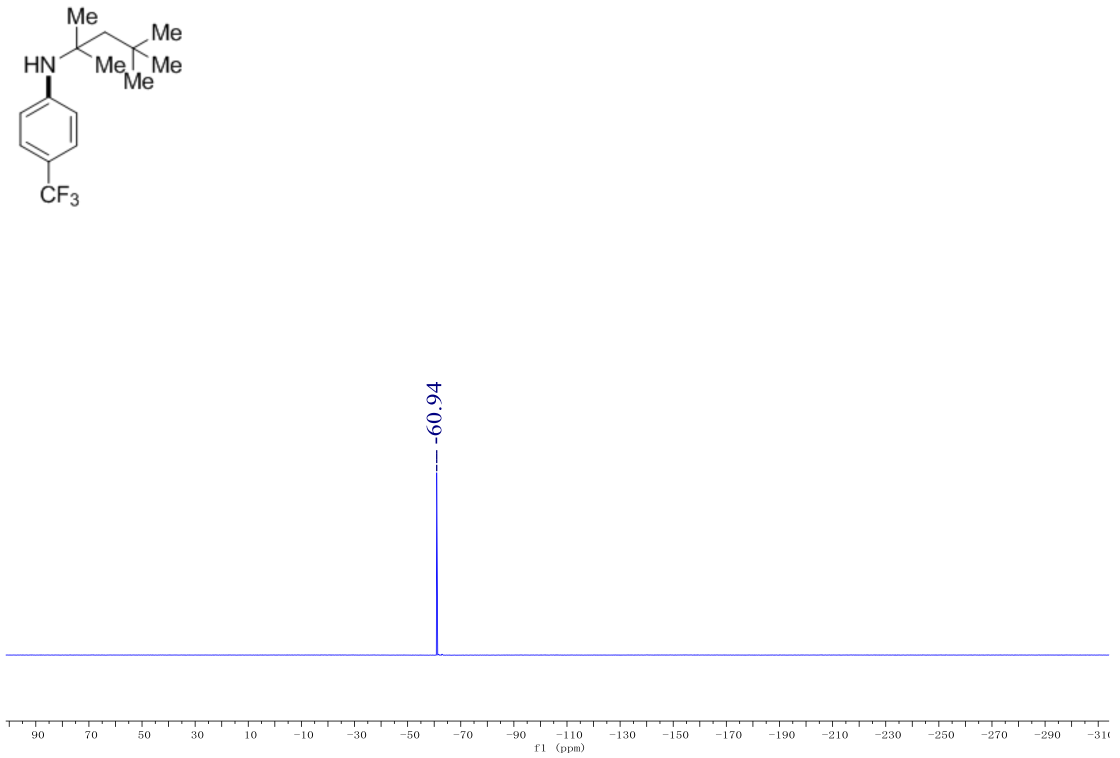
Me
HN
Me
Me
Me
Me
CF3
-60.94
f1 (ppm)

4a

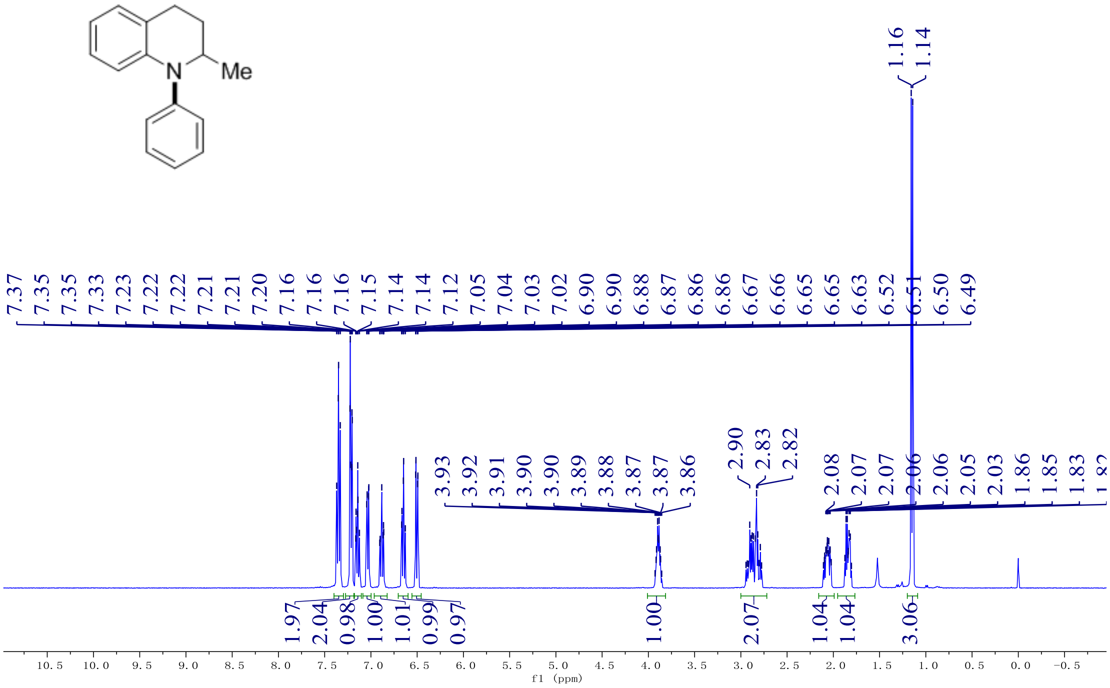

N
Me
7.37 7.35 7.35 7.33 7.23 7.22 7.22 7.21 7.21 7.20 7.16 7.16 7.16 7.15 7.14 7.14 7.12 7.05 7.04 7.03 7.02 6.90 6.90 6.88 6.87 6.86 6.86 6.67 6.66 6.65 6.65 6.63 6.52 6.51 6.50 6.49
1.16 1.14
3.93 3.92 3.91 3.90 3.90 3.89 3.88 3.87 3.87 3.86
2.90 2.83 2.82
2.08 2.07 2.07 2.06 2.06 2.05 2.03 1.86 1.85 1.83
1.97 2.04 0.98 1.00 1.01 0.99 0.97 1.00 2.07 1.04 1.04 3.06
10.5 10.0 9.5 9.0 8.5 8.0 7.5 7.0 6.5 6.0 5.5 5.0 4.5 4.0 3.5 3.0 2.5 2.0 1.5 1.0 0.5 0.0 -0.5
f1 (ppm)

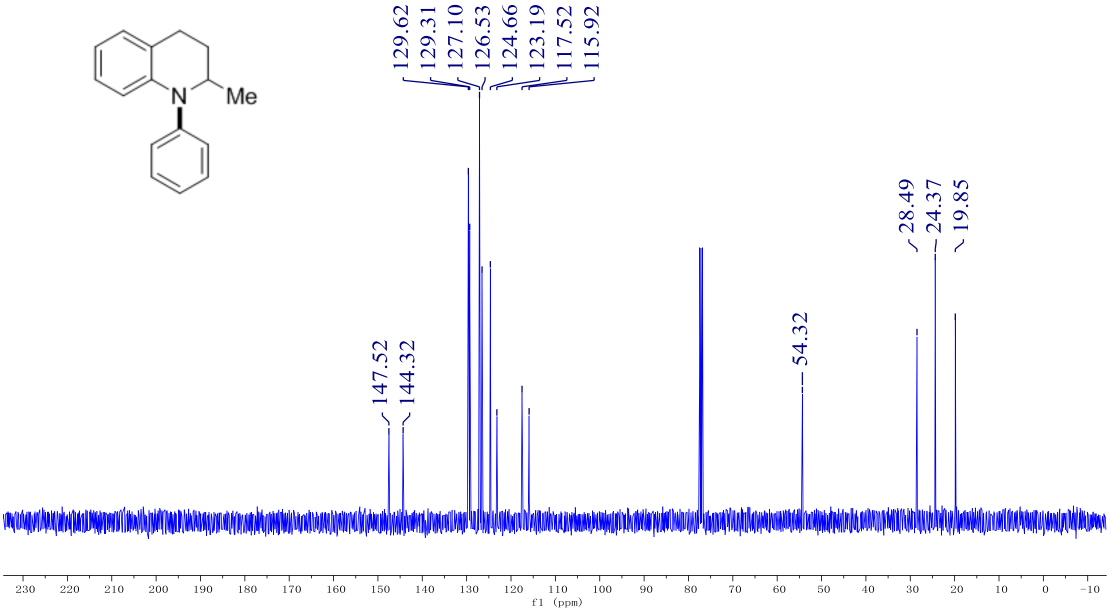

N
Me
129.62 129.31 127.10 126.53 124.66 123.19 117.52 115.92
147.52 144.32
54.32
28.49 24.37 19.85
230 220 210 200 190 180 170 160 150 140 130 120 110 100 90 80 70 60 50 40 30 20 10 0 -10
f1 (ppm)

4b

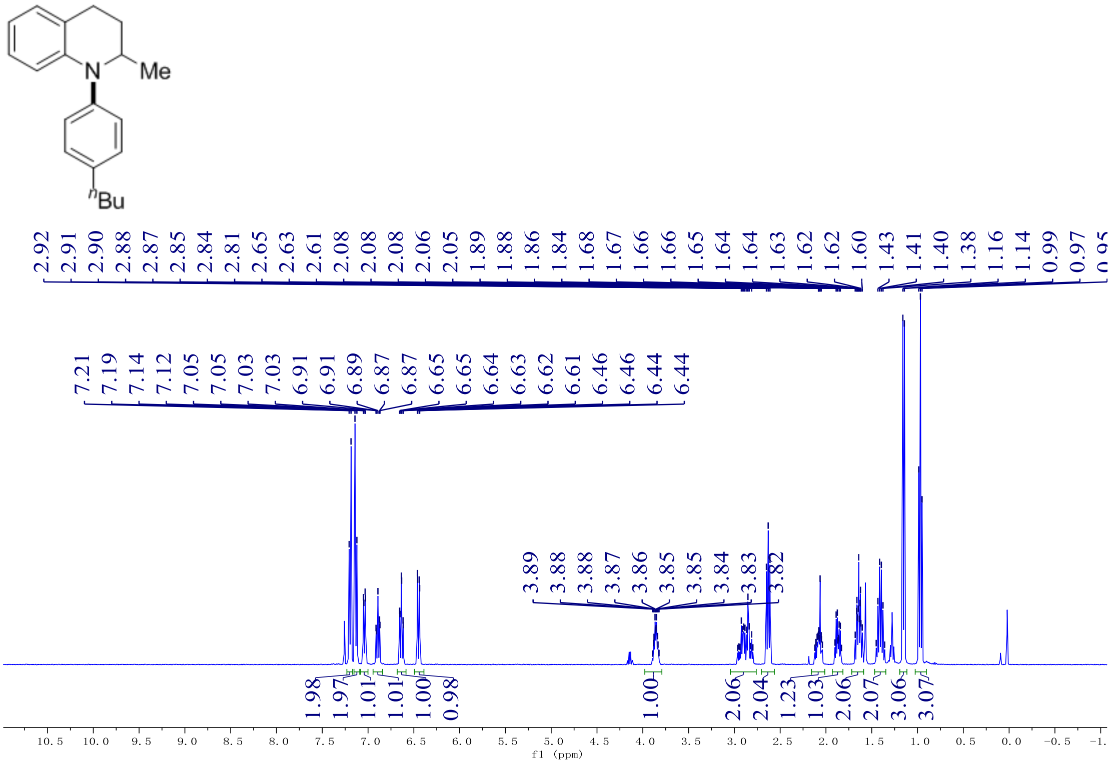

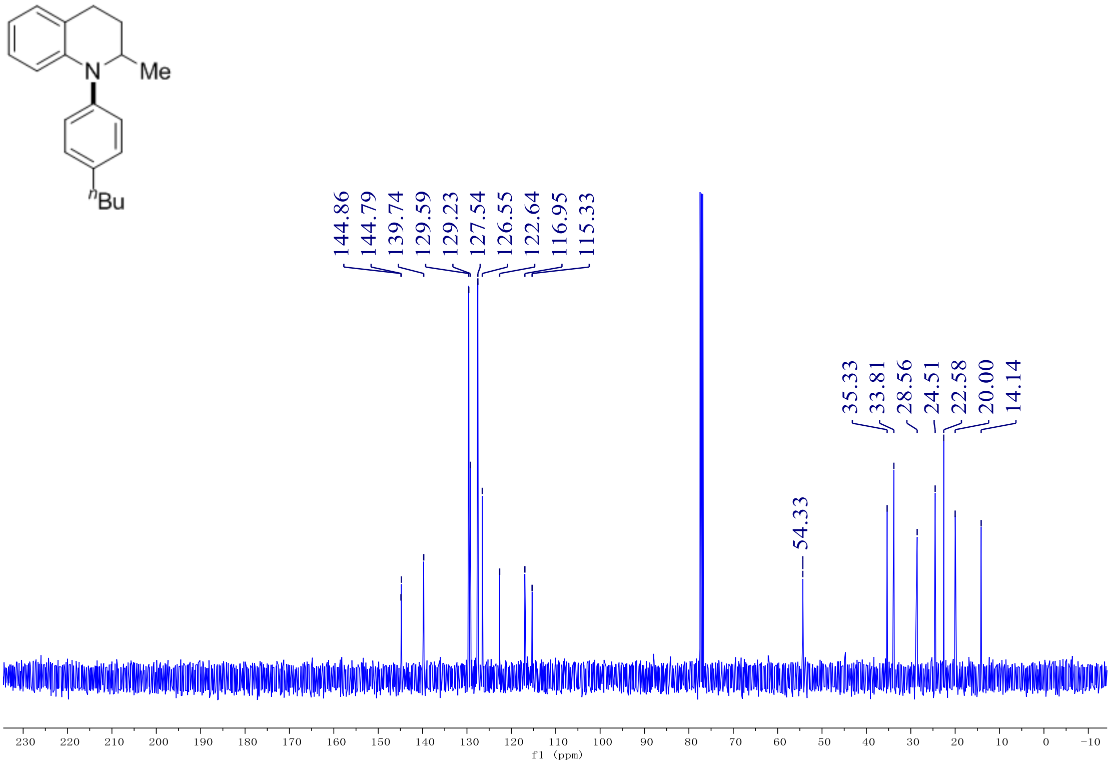

4c

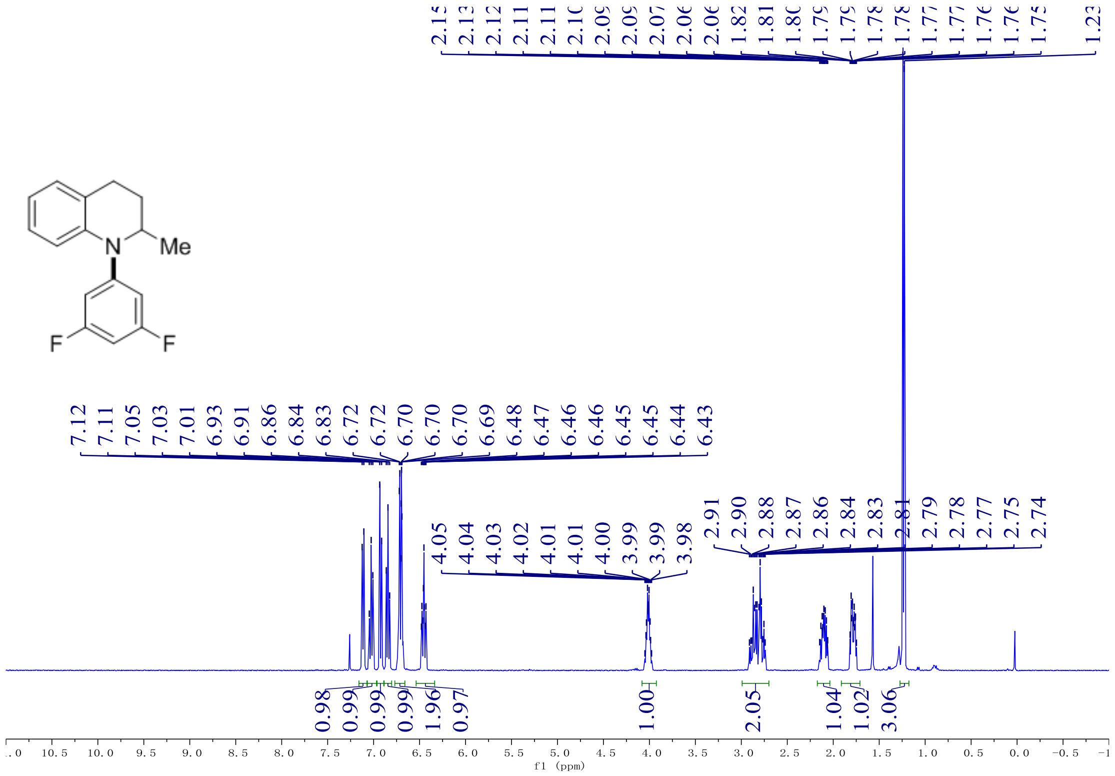
N
Me
F
F
7.12 7.11 7.05 7.03 7.01 6.93 6.91 6.86 6.84 6.83 6.72 6.72 6.70 6.70 6.70 6.69 6.48 6.47 6.46 6.46 6.45 6.45 6.44 6.43
4.05 4.04 4.03 4.02 4.01 4.01 4.00 3.99 3.99 3.98
2.91 2.90 2.88 2.87 2.86 2.84 2.83 2.81 2.79 2.78 2.77 2.75 2.74
1.23
0.98 0.99 0.99 0.99 1.96 0.97 1.00 2.05 1.04 1.02 3.06
f1 (ppm)

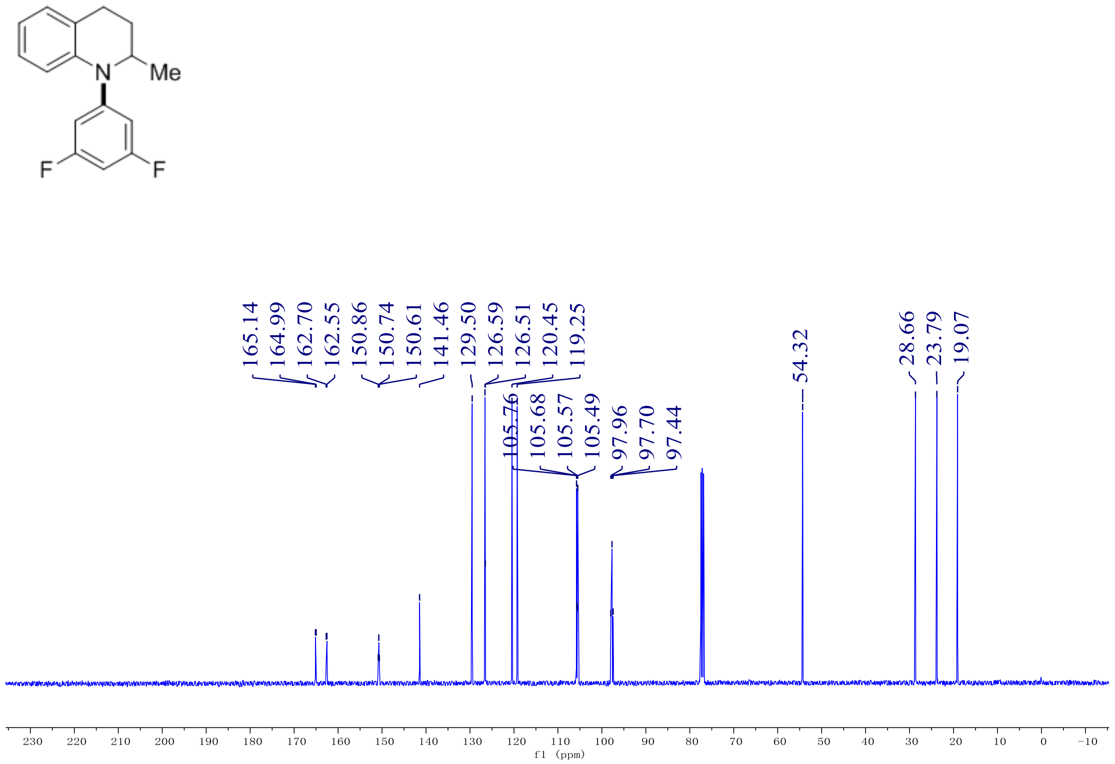
N
Me
F
F
165.14 164.99 162.70 162.55 150.86 150.74 150.61 141.46 129.50 126.59 126.51 120.45 119.25
105.76 105.68 105.57 105.49 97.96 97.70 97.44
54.32
28.66 23.79 19.07
f1 (ppm)

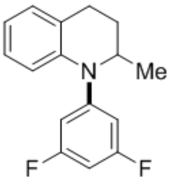

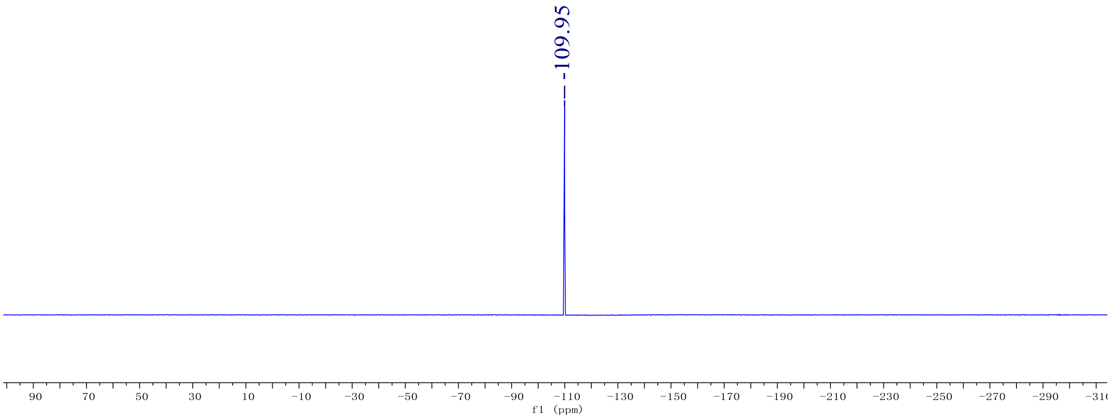

-109.95
90 70 50 30 10 -10 -30 -50 -70 -90 -110 -130 -150 -170 -190 -210 -230 -250 -270 -290 -310
f1 (ppm)

4d

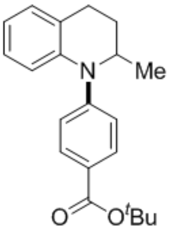

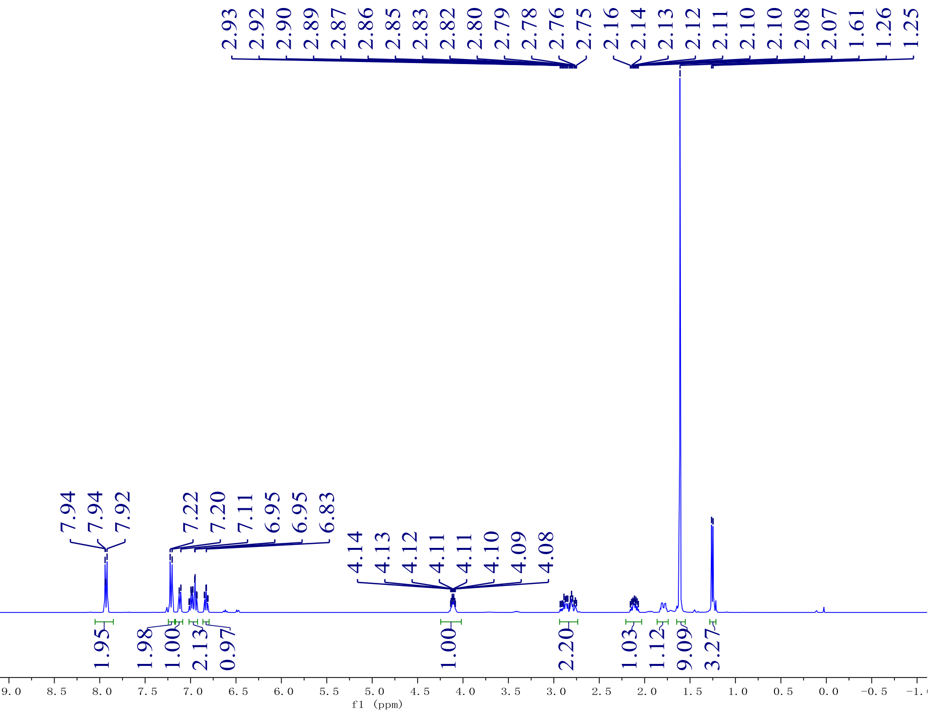

2.93 2.92 2.90 2.89 2.87 2.86 2.85 2.83 2.82 2.80 2.79 2.78 2.76 2.75 2.16 2.14 2.13 2.12 2.11 2.10 2.10 2.08 2.07 1.61 1.26 1.25
7.94 7.94 7.92
7.22 7.20 7.11 6.95 6.95 6.83
4.14 4.13 4.12 4.11 4.11 4.10 4.09 4.08
1.95 1.98 1.00 2.13 0.97 1.00 2.20 1.03 1.12 9.09 3.27
f1 (ppm)

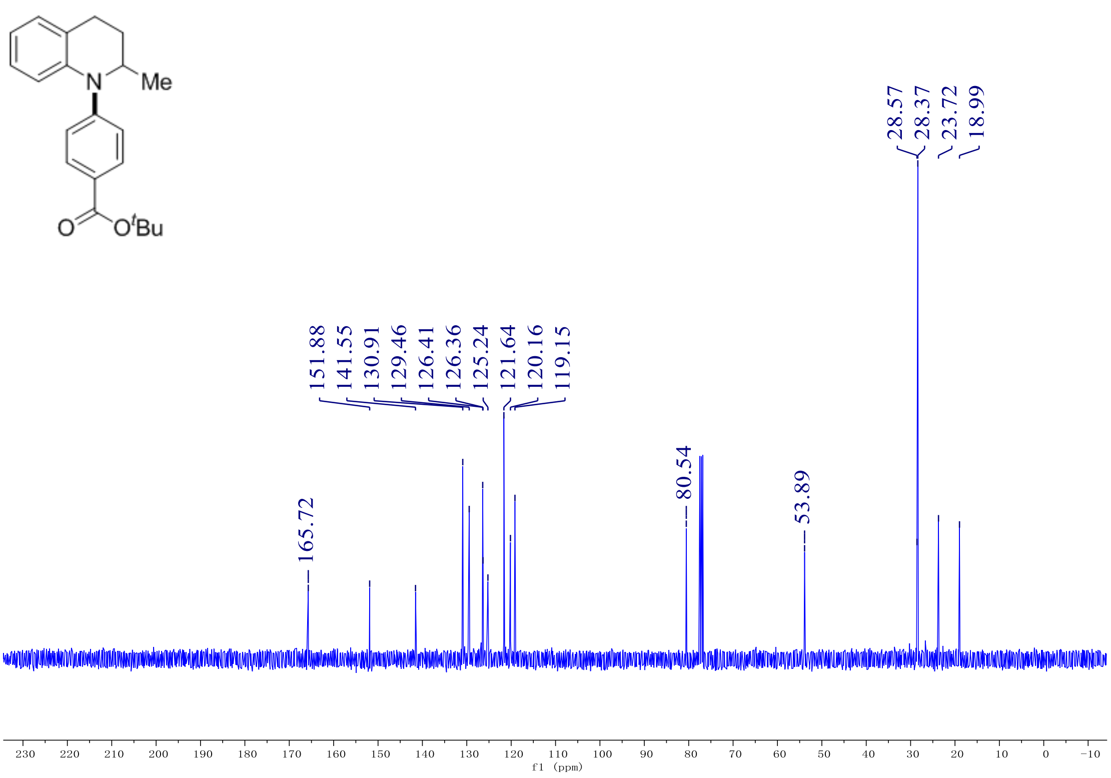
N
Me
O
O^{t}Bu
151.88
141.55
130.91
129.46
126.41
126.36
125.24
121.64
120.16
119.15
165.72
80.54
53.89
28.57
28.37
23.72
18.99
230 220 210 200 190 180 170 160 150 140 130 120 110 100 90 80 70 60 50 40 30 20 10 0 −10
f1 (ppm)

4e

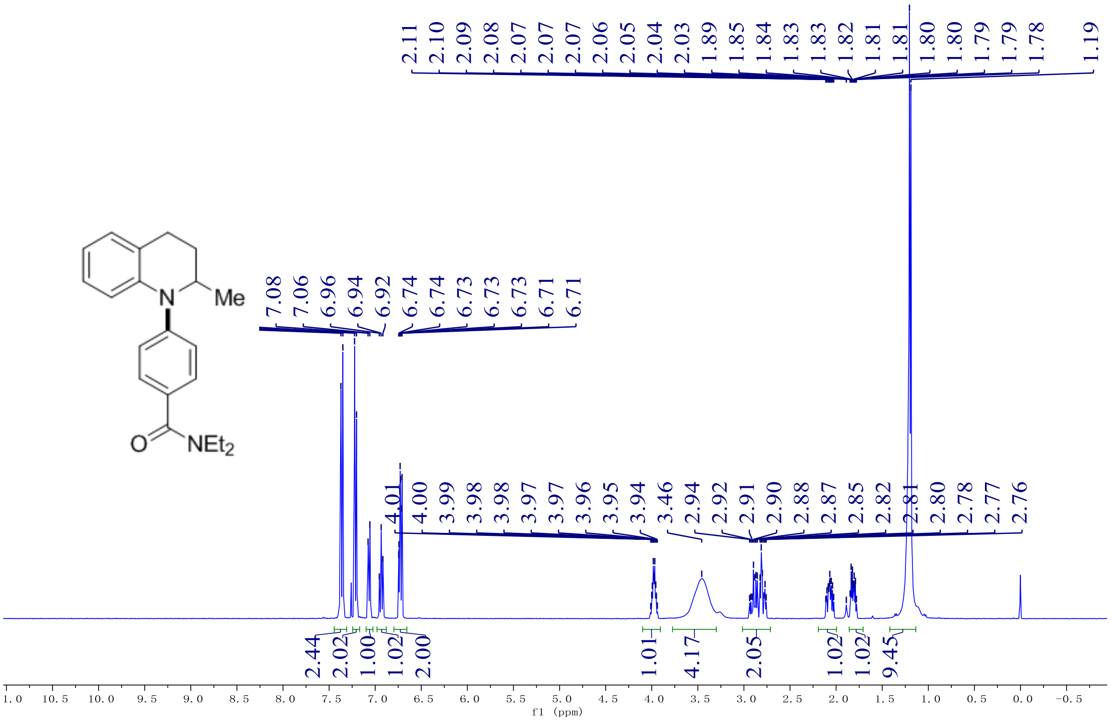
2.11
2.10
2.09
2.08
2.07
2.07
2.07
2.06
2.05
2.04
2.03
1.89
1.85
1.84
1.83
1.83
1.82
1.81
1.81
1.80
1.80
1.79
1.79
1.78
1.19
N
Me
O
NEt$_2$
7.08
7.06
6.96
6.94
6.92
6.74
6.74
6.73
6.73
6.73
6.71
6.71
4.01
4.00
3.99
3.98
3.98
3.97
3.97
3.96
3.95
3.94
3.46
2.94
2.92
2.91
2.90
2.88
2.87
2.85
2.82
2.81
2.80
2.78
2.77
2.76
2.44
2.02
1.00
1.02
2.00
1.01
4.17
2.05
1.02
1.02
9.45
1.0 10.5 10.0 9.5 9.0 8.5 8.0 7.5 7.0 6.5 6.0 5.5 5.0 4.5 4.0 3.5 3.0 2.5 2.0 1.5 1.0 0.5 0.0 −0.5
f1 (ppm)

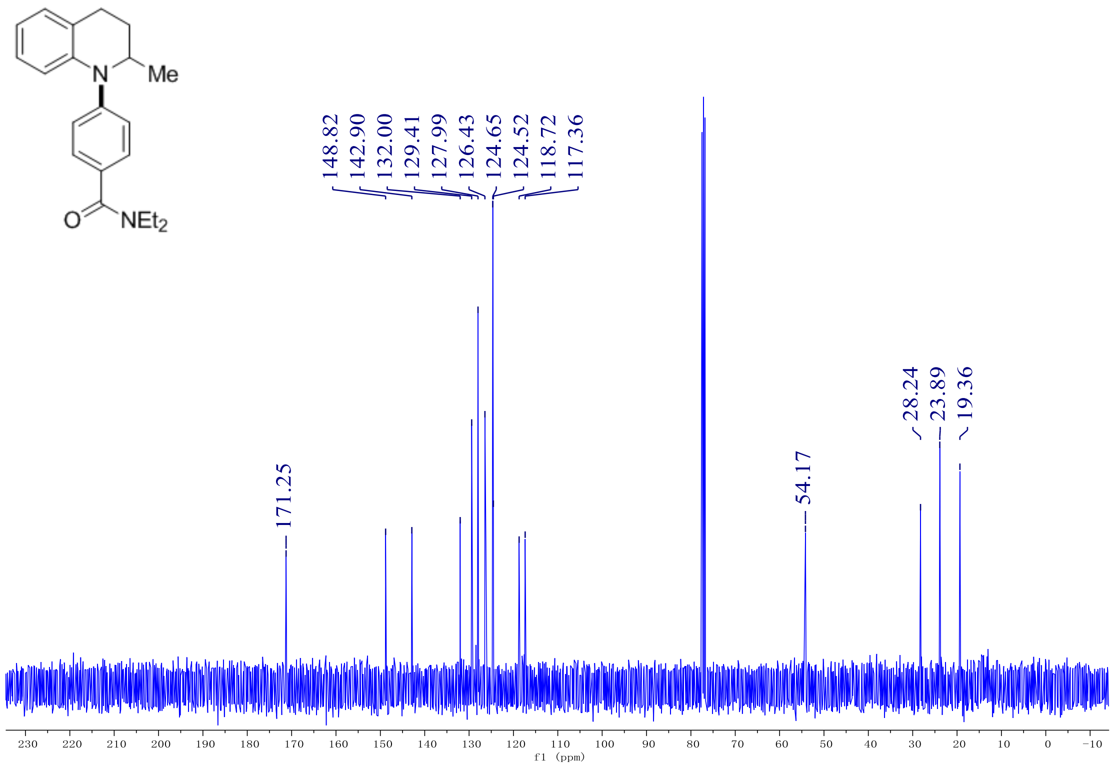
Me
N
O
NEt2
148.82
142.90
132.00
129.41
127.99
126.43
124.65
124.52
118.72
117.36
171.25
54.17
28.24
23.89
19.36
230 220 210 200 190 180 170 160 150 140 130 120 110 100 90 80 70 60 50 40 30 20 10 0 −10
f1 (ppm)

4f

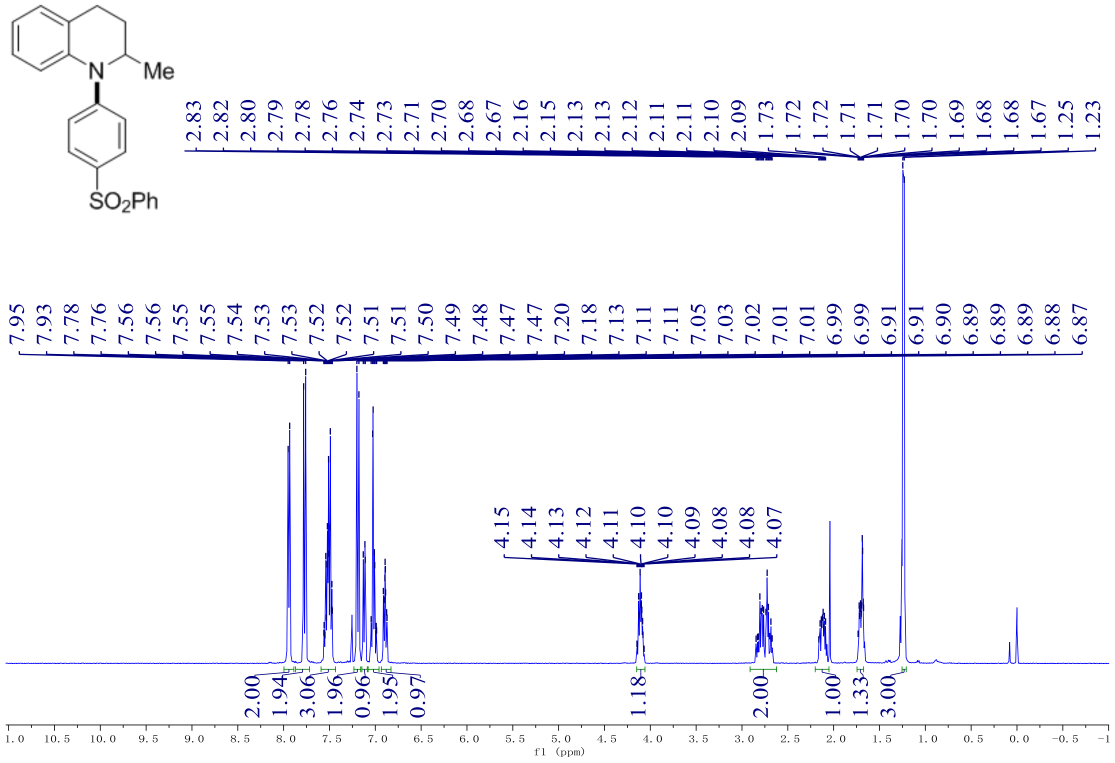
Me
N
SO2Ph
2.83 2.82 2.80 2.79 2.78 2.76 2.74 2.73 2.71 2.70 2.68 2.67 2.16 2.15 2.13 2.13 2.12 2.11 2.11 2.10 2.09 1.73 1.72 1.72 1.71 1.71 1.70 1.70 1.69 1.68 1.68 1.67 1.25 1.23
7.95 7.93 7.78 7.76 7.56 7.56 7.55 7.55 7.54 7.53 7.53 7.52 7.52 7.51 7.51 7.50 7.49 7.48 7.47 7.47 7.20 7.18 7.13 7.11 7.11 7.05 7.03 7.02 7.01 7.01 6.99 6.99 6.91 6.91 6.90 6.89 6.89 6.89 6.88 6.87
4.15 4.14 4.13 4.12 4.11 4.10 4.10 4.09 4.08 4.08 4.07
2.00 1.94 3.06 1.96 0.96 1.95 0.97 1.18 2.00 1.00 1.33 3.00
1.0 10.5 10.0 9.5 9.0 8.5 8.0 7.5 7.0 6.5 6.0 5.5 5.0 4.5 4.0 3.5 3.0 2.5 2.0 1.5 1.0 0.5 0.0 −0.5 −1
f1 (ppm)

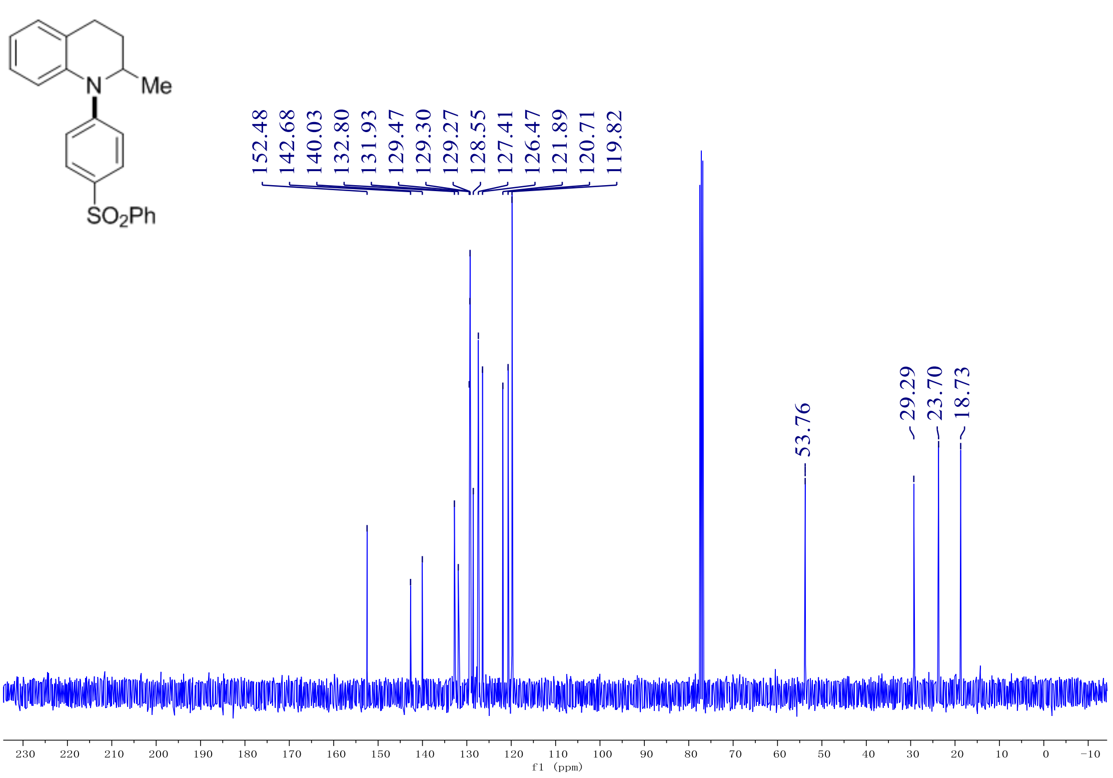
Me
N
SO2Ph
152.48
142.68
140.03
132.80
131.93
129.47
129.30
129.27
128.55
127.41
126.47
121.89
120.71
119.82
53.76
29.29
23.70
18.73
f1 (ppm)

4g

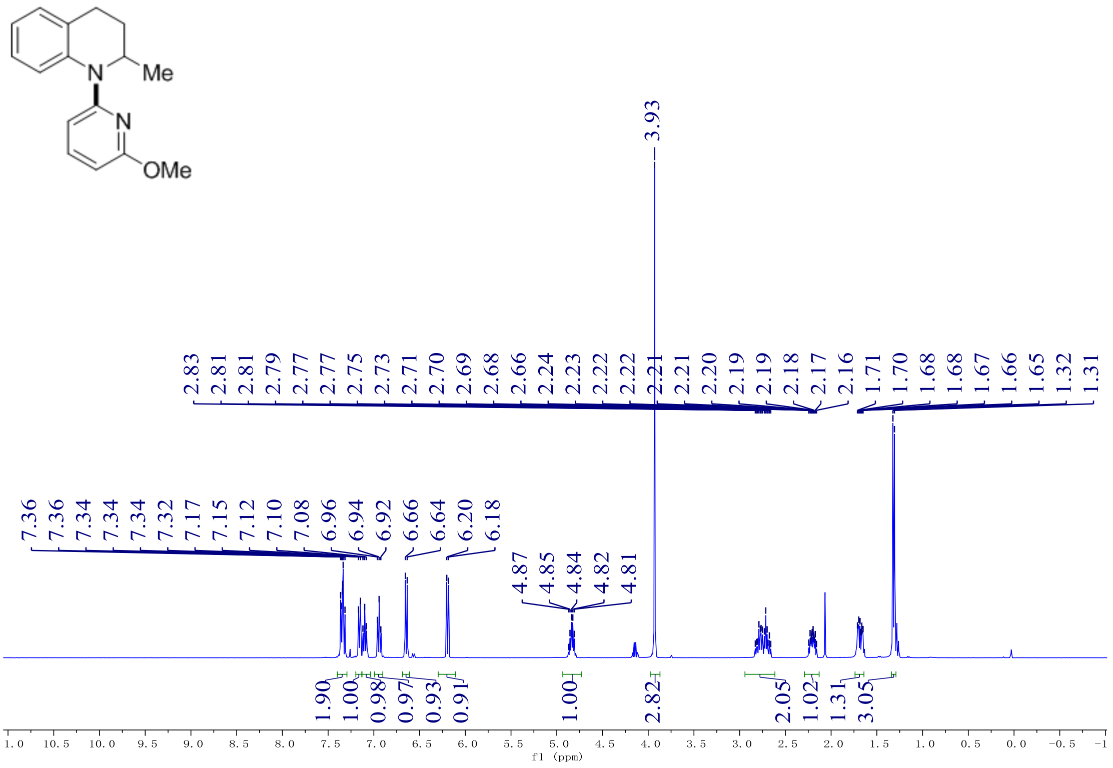
Me
N
N
OMe
3.93
2.83
2.81
2.81
2.79
2.77
2.77
2.75
2.73
2.71
2.70
2.69
2.68
2.66
2.24
2.23
2.22
2.22
2.21
2.21
2.20
2.19
2.19
2.18
2.17
2.16
1.71
1.70
1.68
1.68
1.67
1.66
1.65
1.32
1.31
7.36
7.36
7.34
7.34
7.34
7.32
7.17
7.15
7.12
7.10
7.08
6.96
6.94
6.92
6.66
6.64
6.20
6.18
4.87
4.85
4.84
4.82
4.81
1.90
1.00
0.98
0.97
0.93
0.91
1.00
2.82
2.05
1.02
1.31
3.05
f1 (ppm)

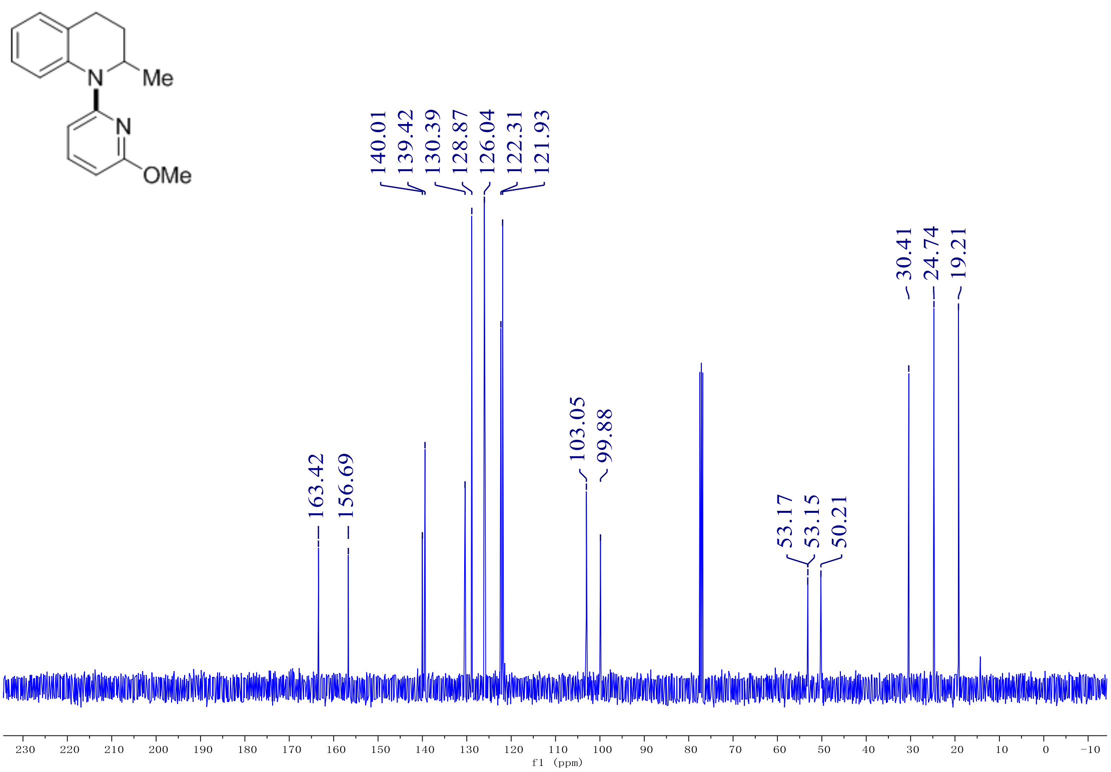

N
Me
OMe
140.01
139.42
130.39
128.87
126.04
122.31
121.93
163.42
156.69
103.05
99.88
53.17
53.15
50.21
30.41
24.74
19.21
230 220 210 200 190 180 170 160 150 140 130 120 110 100 90 80 70 60 50 40 30 20 10 0 -10
f1 (ppm)

4h

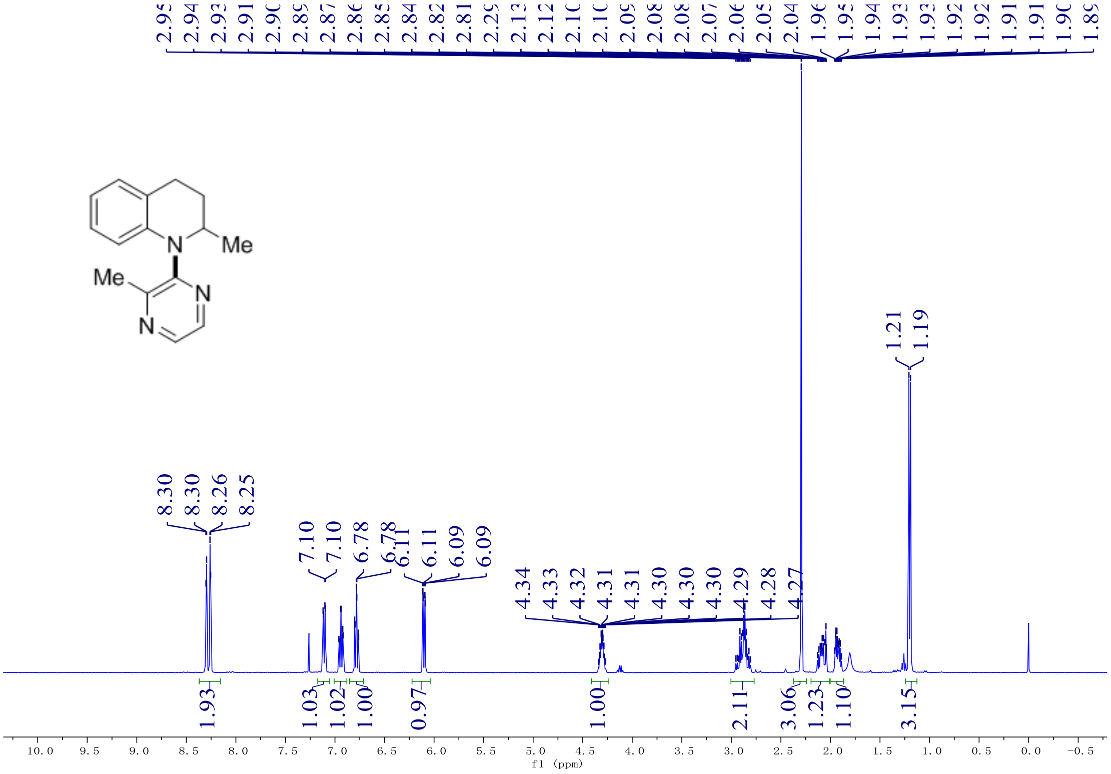

N
Me
N
Me
N
2.94
2.93
2.91
2.87
2.85
2.84
2.82
2.81
2.12
2.09
2.04
1.95
1.94
1.93
1.92
1.91
1.90
1.21
1.19
8.30
8.30
8.26
8.25
7.10
7.10
6.78
6.78
6.11
6.11
6.09
6.09
4.34
4.33
4.32
4.31
4.31
4.30
4.30
4.30
4.29
4.28
4.27
1.93
1.03
1.02
1.00
0.97
1.00
2.11
3.06
1.23
1.10
3.15
10.0 9.5 9.0 8.5 8.0 7.5 7.0 6.5 6.0 5.5 5.0 4.5 4.0 3.5 3.0 2.5 2.0 1.5 1.0 0.5 0.0 -0.5
f1 (ppm)

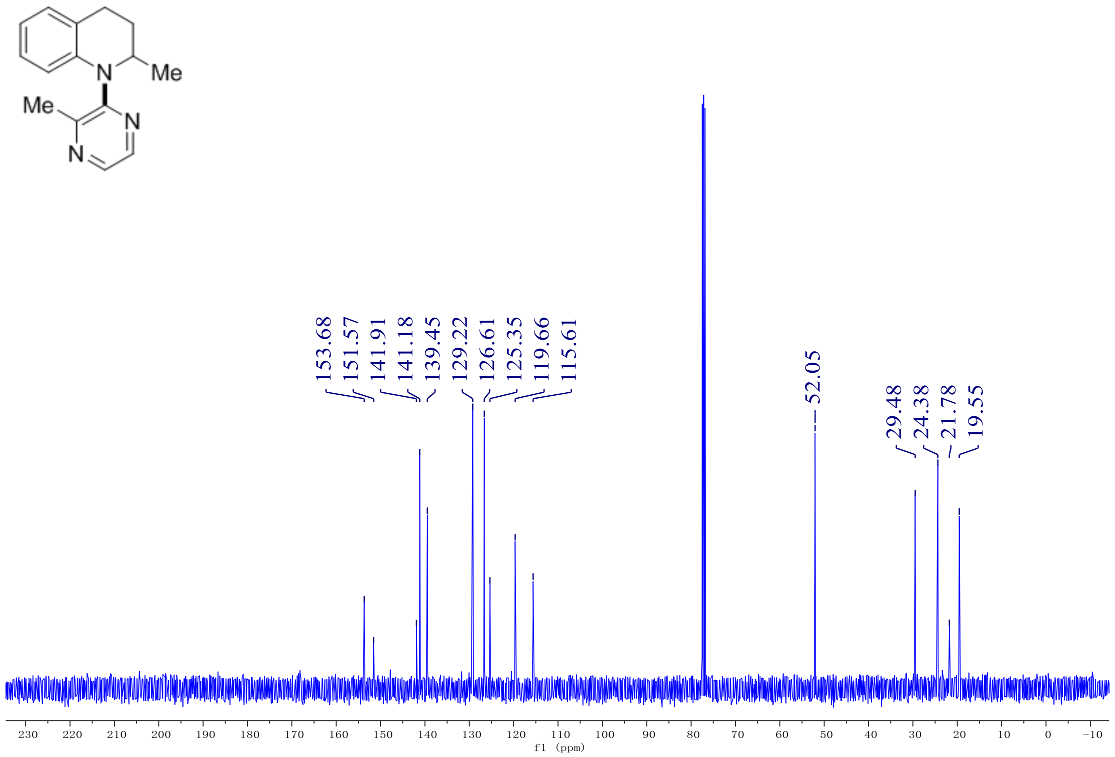
Me
N
Me
N
N
153.68
151.57
141.91
141.18
139.45
129.22
126.61
125.35
119.66
115.61
52.05
29.48
24.38
21.78
19.55
f1 (ppm)

4i

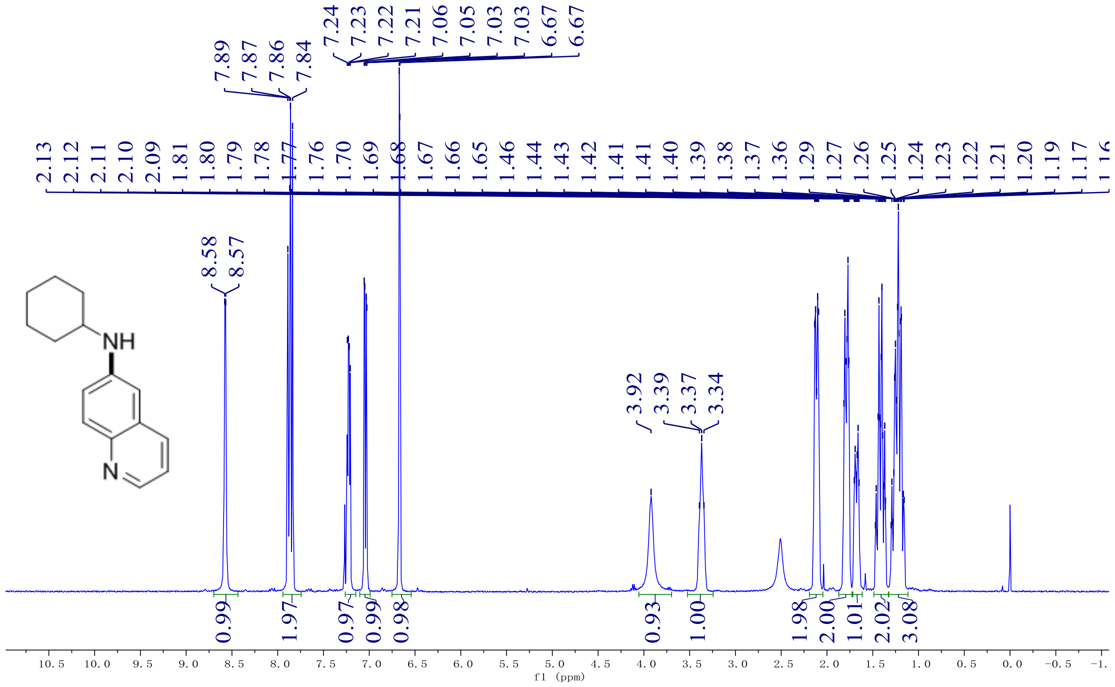
NH
N
7.89
7.87
7.86
7.84
7.24
7.23
7.22
7.21
7.06
7.05
7.03
7.03
6.67
6.67
2.13
2.12
2.11
2.10
2.09
1.81
1.80
1.79
1.78
1.77
1.76
1.70
1.69
1.68
1.67
1.66
1.65
1.46
1.44
1.43
1.42
1.41
1.41
1.40
1.39
1.38
1.37
1.36
1.29
1.27
1.26
1.25
1.24
1.23
1.22
1.21
1.20
1.19
1.17
8.58
8.57
3.92
3.39
3.37
3.34
0.99
1.97
0.97
0.99
0.98
0.93
1.00
1.98
2.00
1.01
2.02
3.08
f1 (ppm)

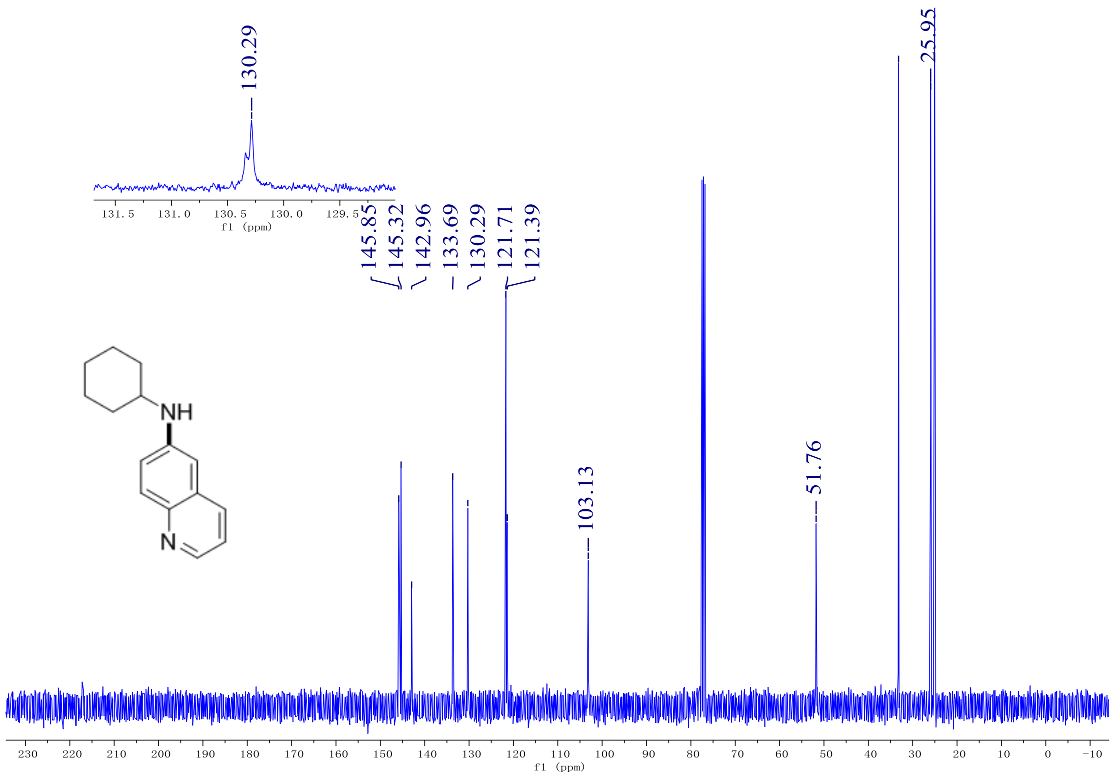
130.29
131.5
131.0
130.5
130.0
129.5
f1 (ppm)
145.85
145.32
142.96
133.69
130.29
121.71
121.39
NH
N
103.13
51.76
25.95
230
220
210
200
190
180
170
160
150
140
130
120
110
100
90
80
70
60
50
40
30
20
10
0
-10
f1 (ppm)

4j

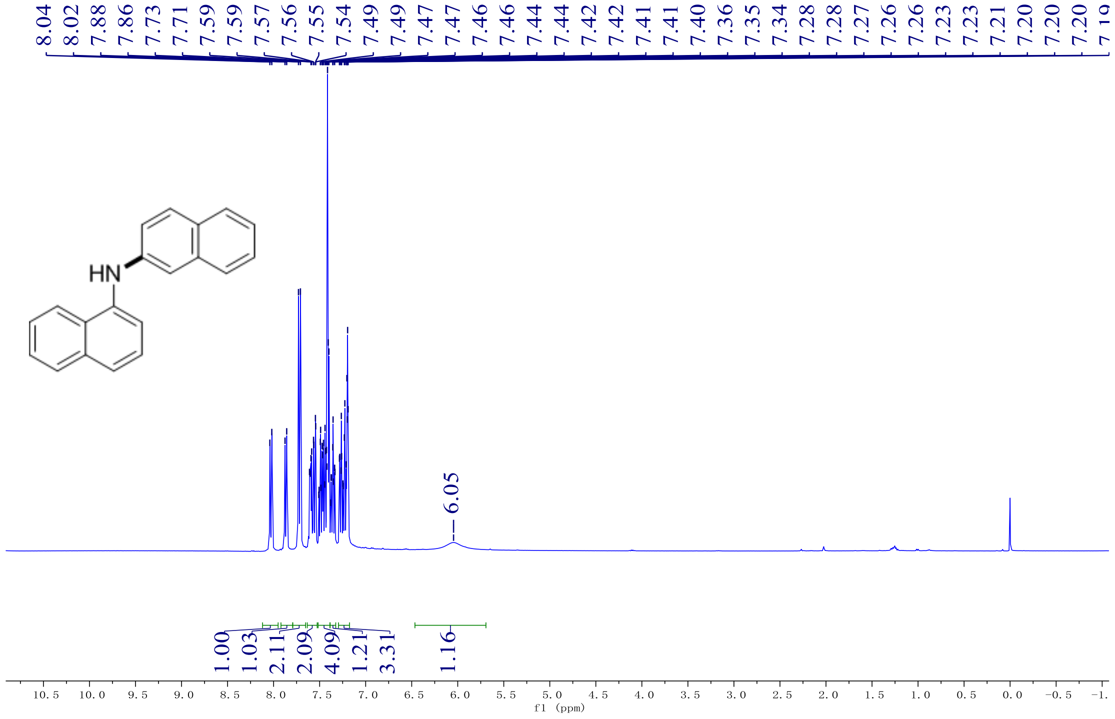
8.04
8.02
7.88
7.86
7.73
7.71
7.59
7.59
7.57
7.56
7.55
7.54
7.49
7.49
7.47
7.47
7.46
7.46
7.44
7.44
7.42
7.42
7.41
7.41
7.40
7.36
7.35
7.34
7.28
7.28
7.27
7.26
7.26
7.23
7.23
7.21
7.20
7.20
7.20
HN
6.05
1.00
1.03
2.11
2.09
4.09
1.21
3.31
1.16
10.5
10.0
9.5
9.0
8.5
8.0
7.5
7.0
6.5
6.0
5.5
5.0
4.5
4.0
3.5
3.0
2.5
2.0
1.5
1.0
0.5
0.0
-0.5
f1 (ppm)

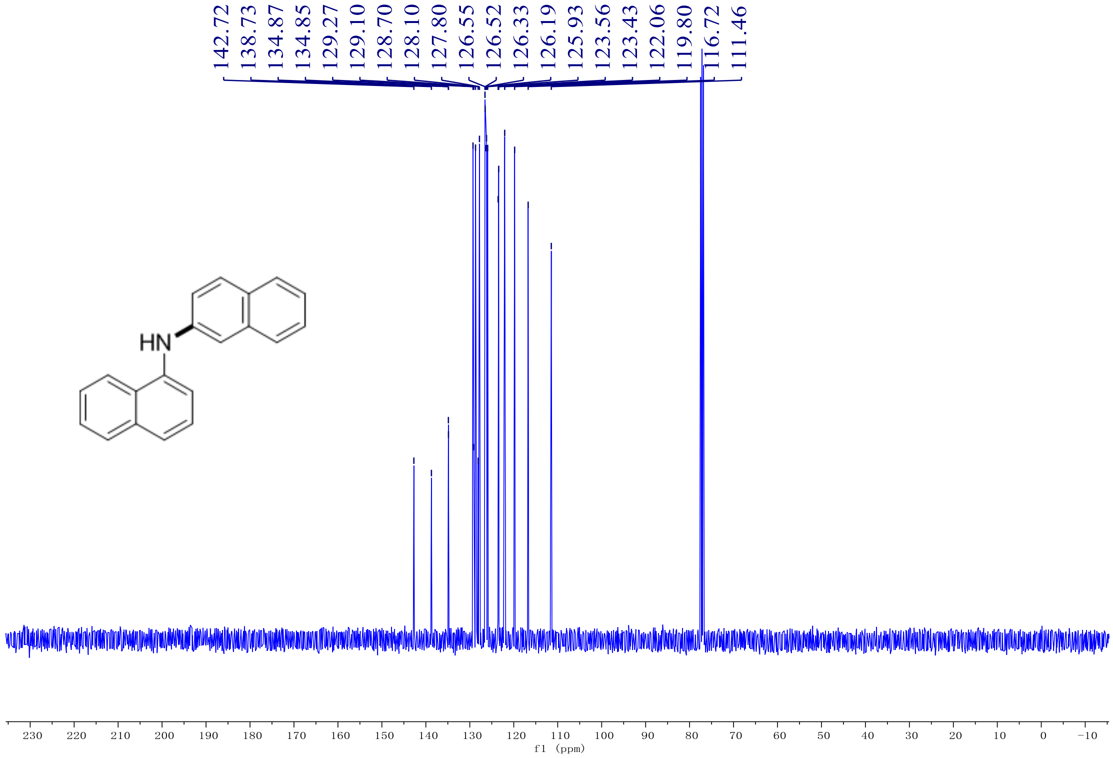
142.72
138.73
134.87
134.85
129.27
129.10
128.70
128.10
127.80
126.55
126.52
126.33
126.19
125.93
123.56
123.43
122.06
119.80
116.72
111.46
HN
f1 (ppm)

4k

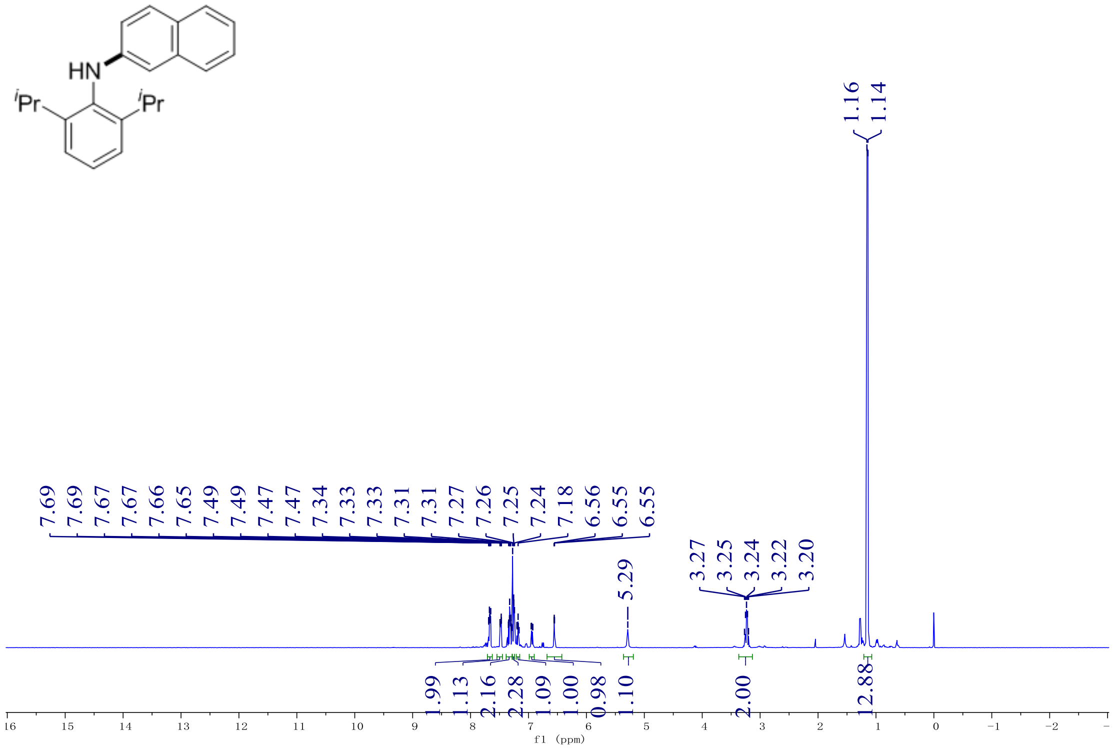
HN
iPr
iPr
1.16
1.14
7.69
7.69
7.67
7.67
7.66
7.65
7.49
7.49
7.47
7.47
7.34
7.33
7.33
7.31
7.31
7.27
7.26
7.25
7.24
7.18
6.56
6.55
6.55
5.29
3.27
3.25
3.24
3.22
3.20
1.99
1.13
2.16
2.28
1.09
1.00
0.98
1.10
2.00
12.88
f1 (ppm)

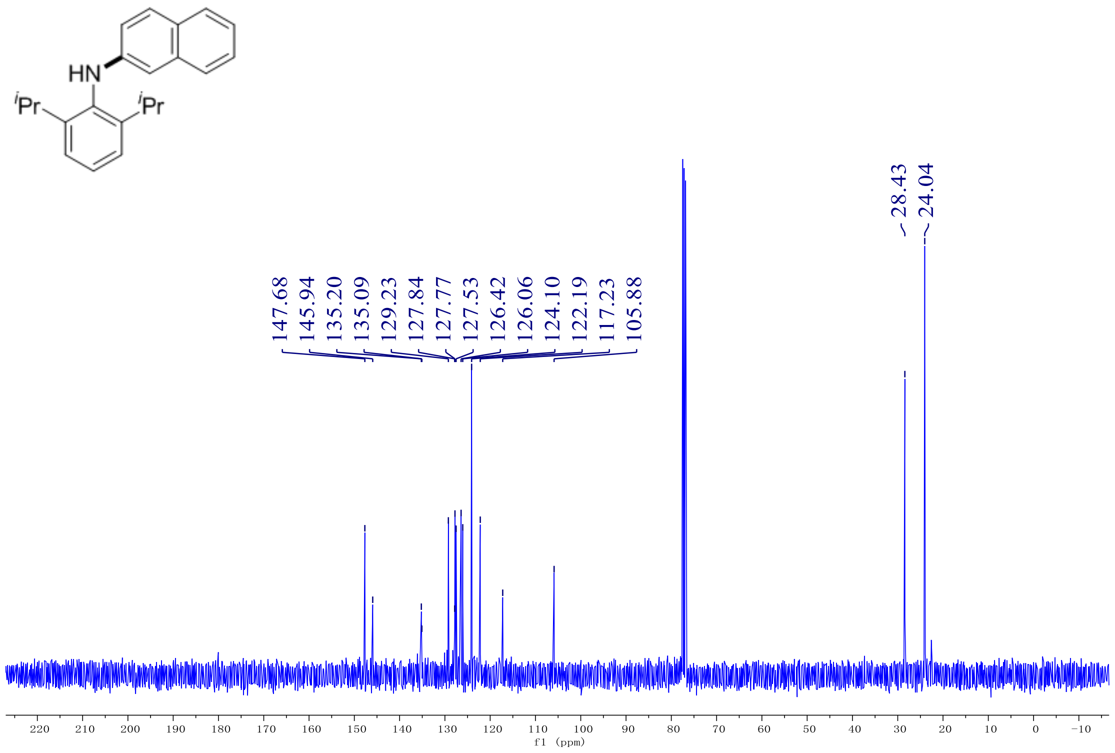
HN
iPr
iPr
147.68
145.94
135.20
135.09
129.23
127.84
127.77
127.53
126.42
126.06
124.10
122.19
117.23
105.88
28.43
24.04
f1 (ppm)

4l

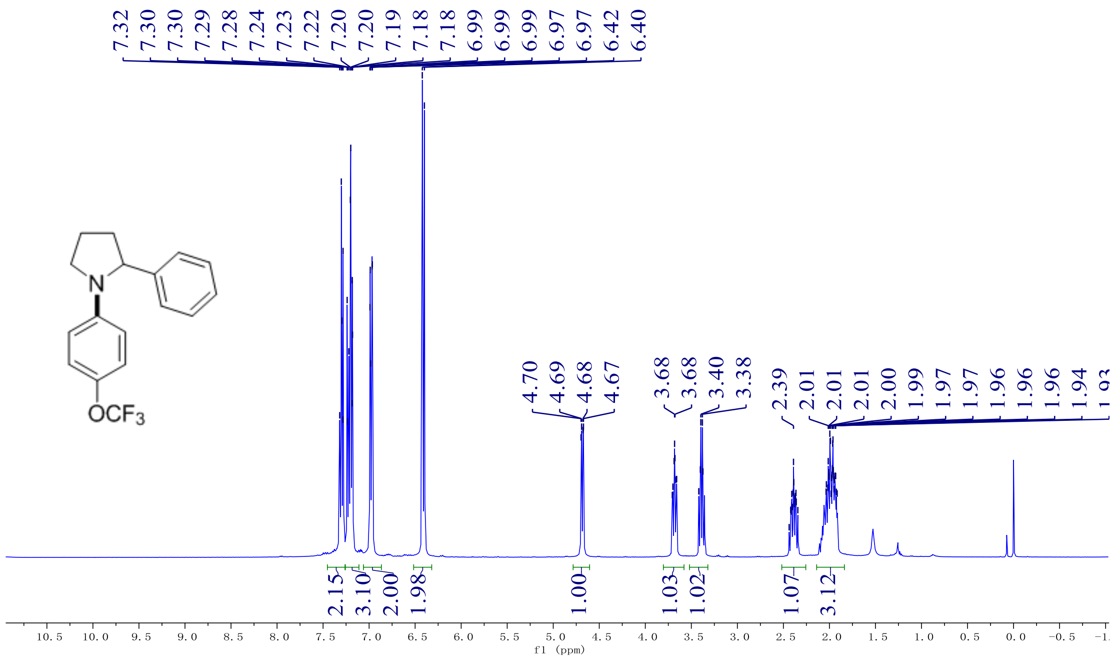
N
OCF3
7.32
7.30
7.30
7.29
7.28
7.24
7.23
7.22
7.20
7.20
7.19
7.18
6.99
6.99
6.99
6.97
6.97
6.42
6.40
4.70
4.69
4.68
4.67
3.68
3.68
3.40
3.38
2.39
2.01
2.01
2.01
2.00
1.99
1.97
1.97
1.96
1.96
1.96
1.94
2.15
3.10
2.00
1.98
1.00
1.03
1.02
1.07
3.12
f1 (ppm)

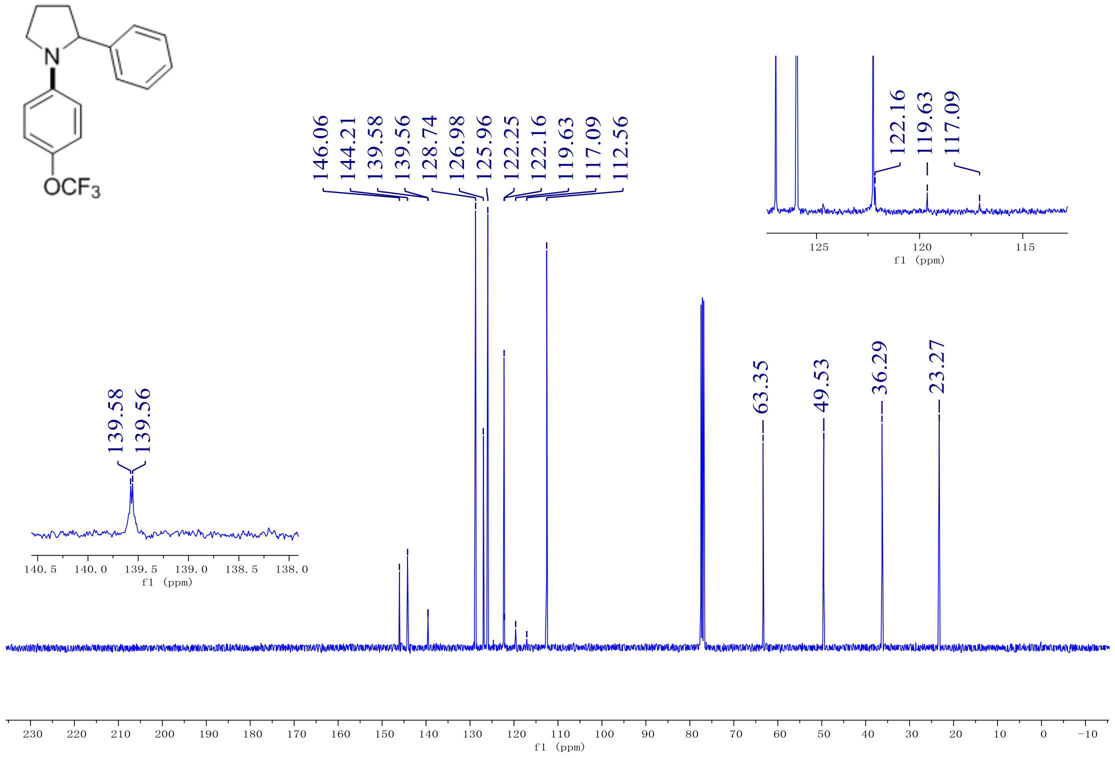
OCF3
146.06
144.21
139.58
139.56
128.74
126.98
125.96
122.25
122.16
119.63
117.09
112.56
63.35
49.53
36.29
23.27
f1 (ppm)

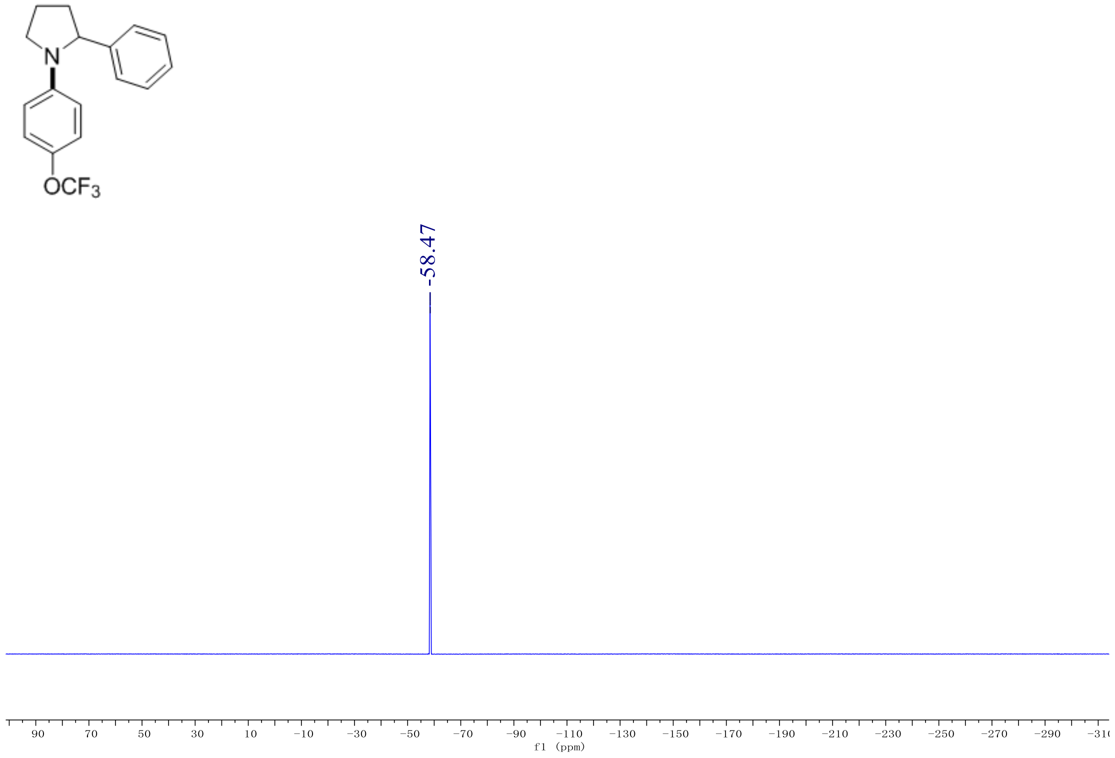
OCF3
-58.47
f1 (ppm)

4m

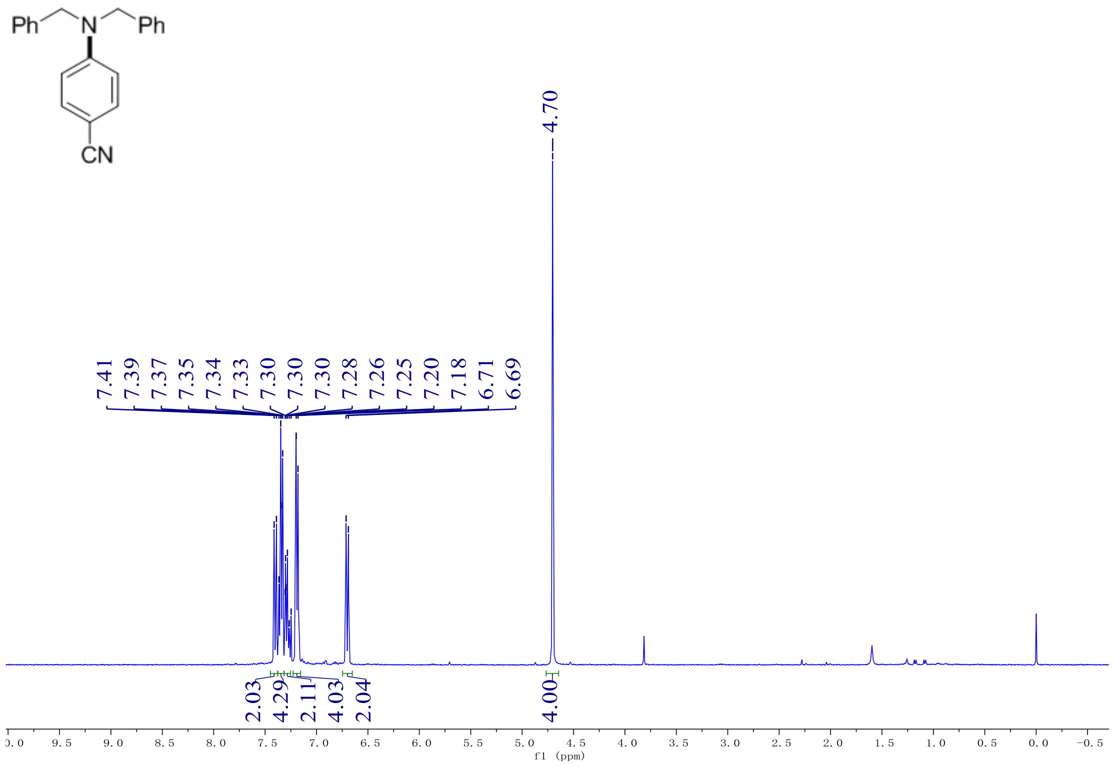
Ph
N
Ph
CN
7.41
7.39
7.37
7.35
7.34
7.33
7.30
7.30
7.30
7.28
7.26
7.25
7.20
7.18
6.71
6.69
4.70
2.03
4.29
2.11
4.03
2.04
4.00
f1 (ppm)

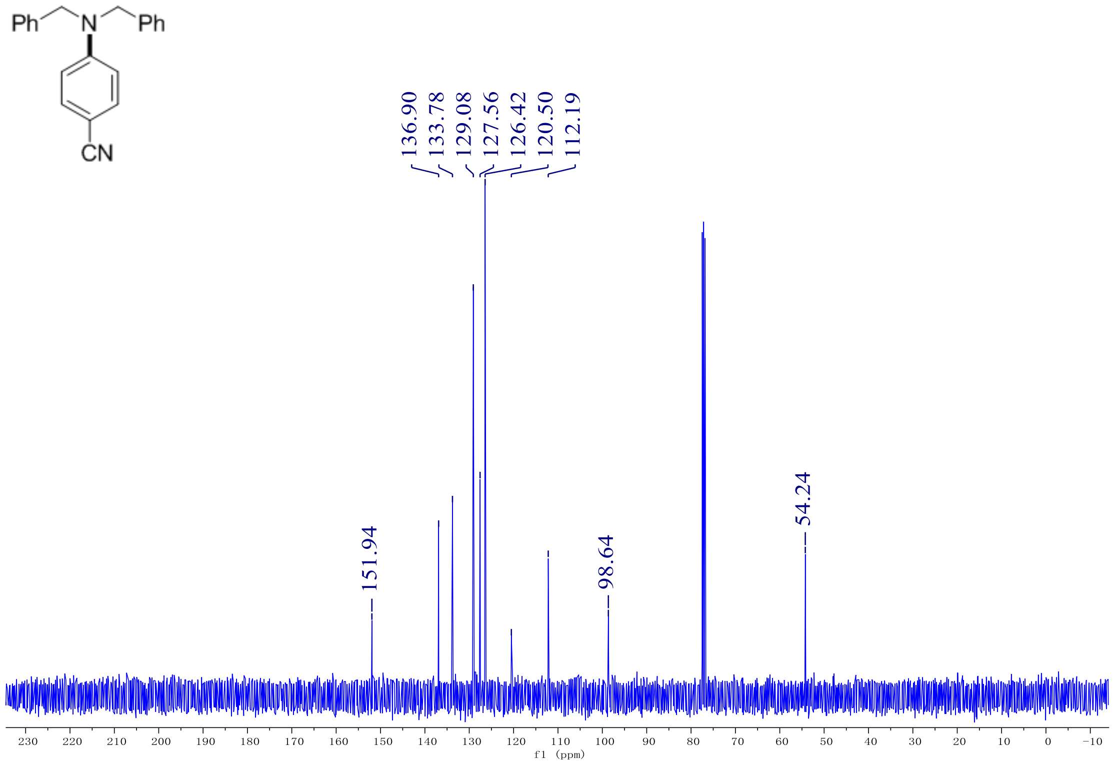
Ph
N
Ph
CN
136.90
133.78
129.08
127.56
126.42
120.50
112.19
151.94
98.64
54.24
f1 (ppm)

4n

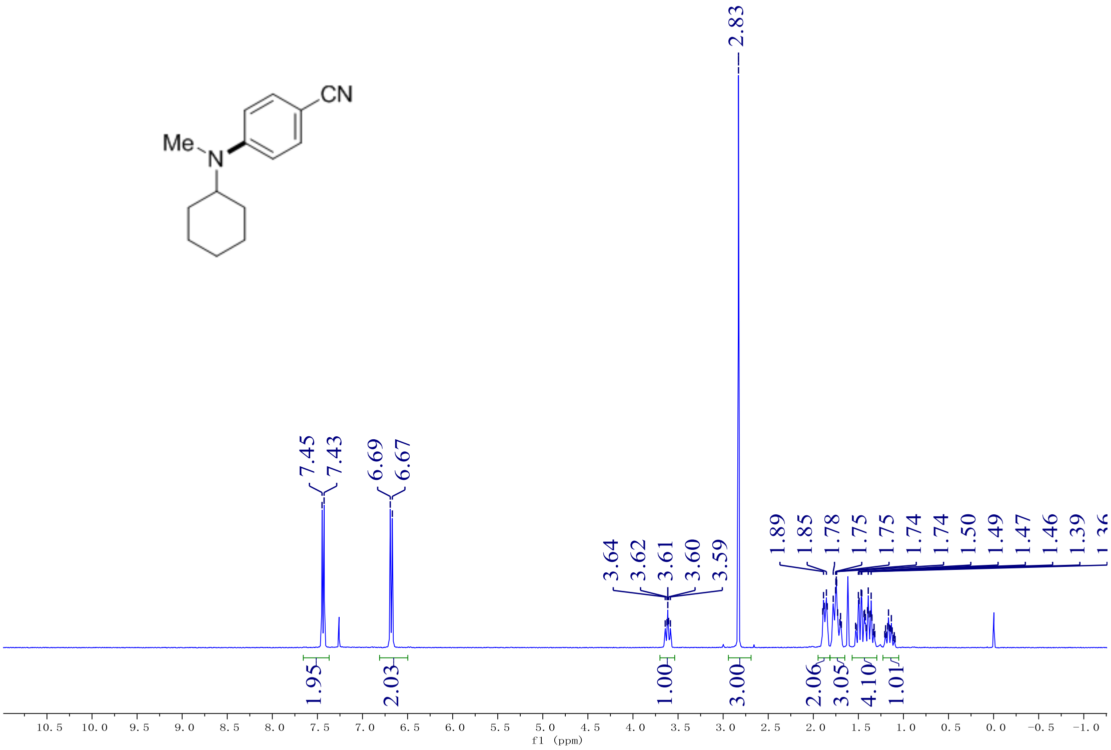
CN
Me
N
2.83
7.45
7.43
6.69
6.67
3.64
3.62
3.61
3.60
3.59
1.89
1.85
1.78
1.75
1.75
1.74
1.74
1.50
1.49
1.47
1.46
1.39
1.95
2.03
1.00
3.00
2.06
3.05
4.10
1.01
f1 (ppm)

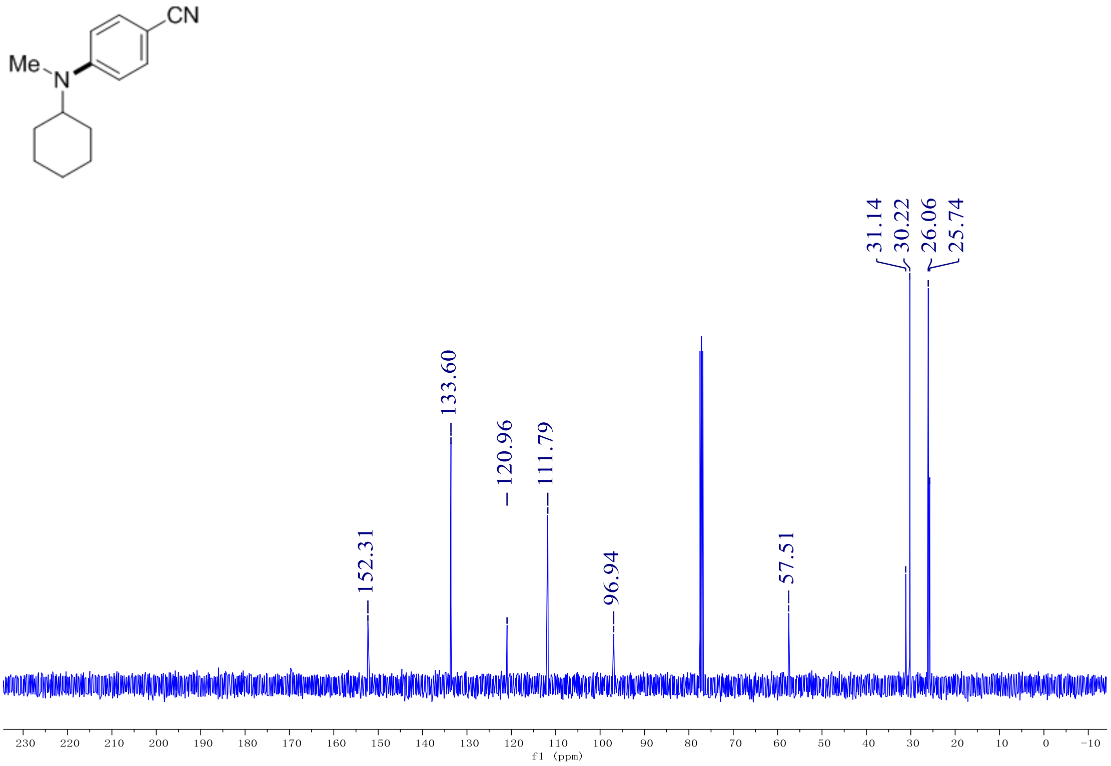
CN
Me
N
31.14
30.22
26.06
25.74
133.60
120.96
111.79
152.31
96.94
57.51
f1 (ppm)

4o

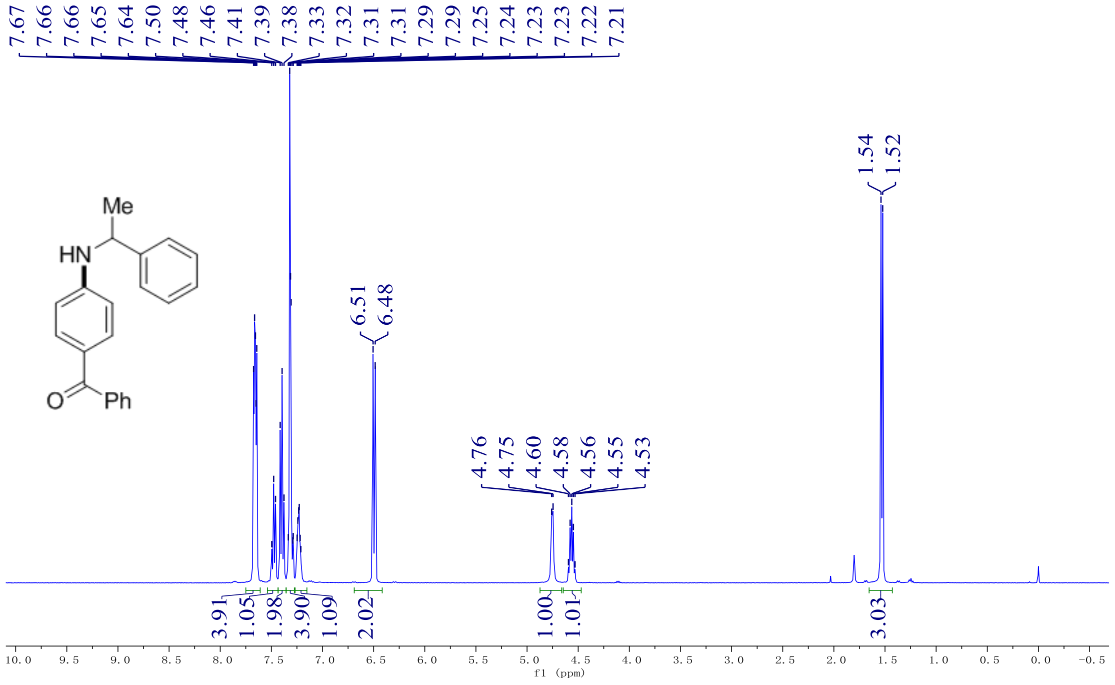
7.67
7.66
7.66
7.65
7.64
7.50
7.48
7.46
7.41
7.39
7.38
7.33
7.32
7.31
7.31
7.29
7.29
7.25
7.24
7.23
7.23
7.22
7.21
1.54
1.52
6.51
6.48
4.76
4.75
4.60
4.58
4.56
4.55
4.53
Me
HN
O
Ph
3.91
1.05
1.98
3.90
1.09
2.02
1.00
1.01
3.03
f1 (ppm)

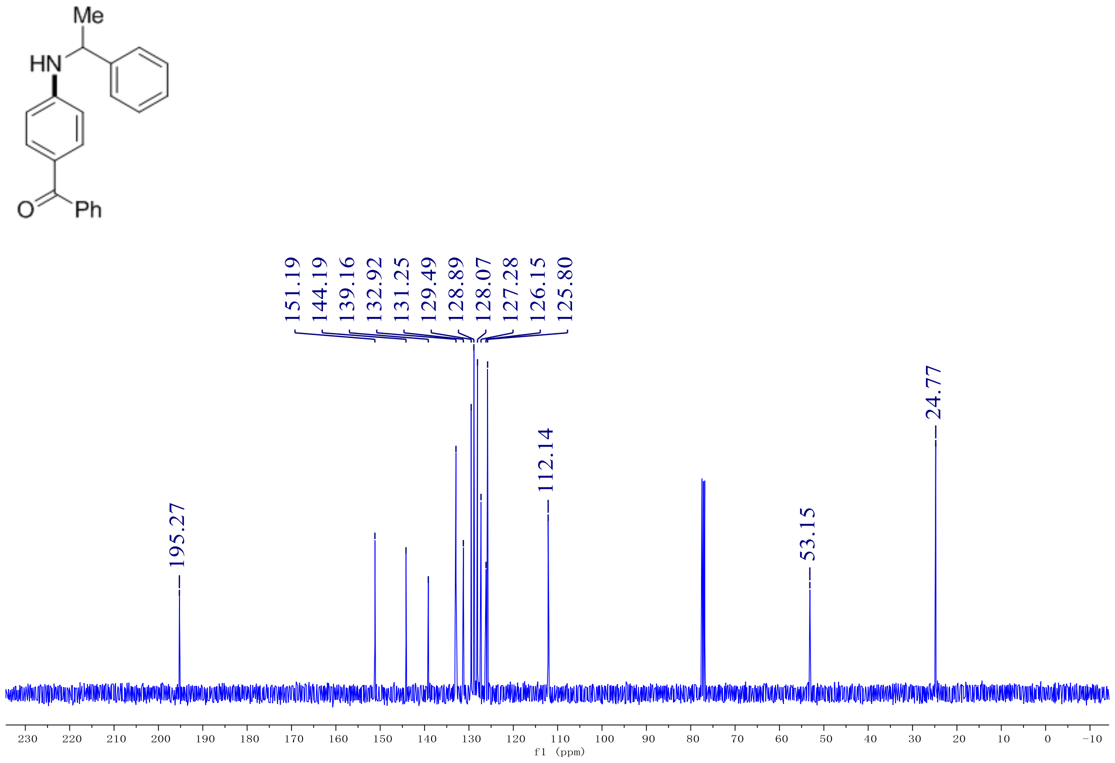
Me
HN
O
Ph
151.19
144.19
139.16
132.92
131.25
129.49
128.89
128.07
127.28
126.15
125.80
24.77
112.14
195.27
53.15
f1 (ppm)

4p

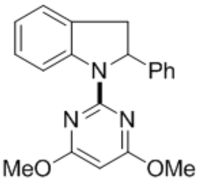

N
Ph
N
N
MeO
OMe

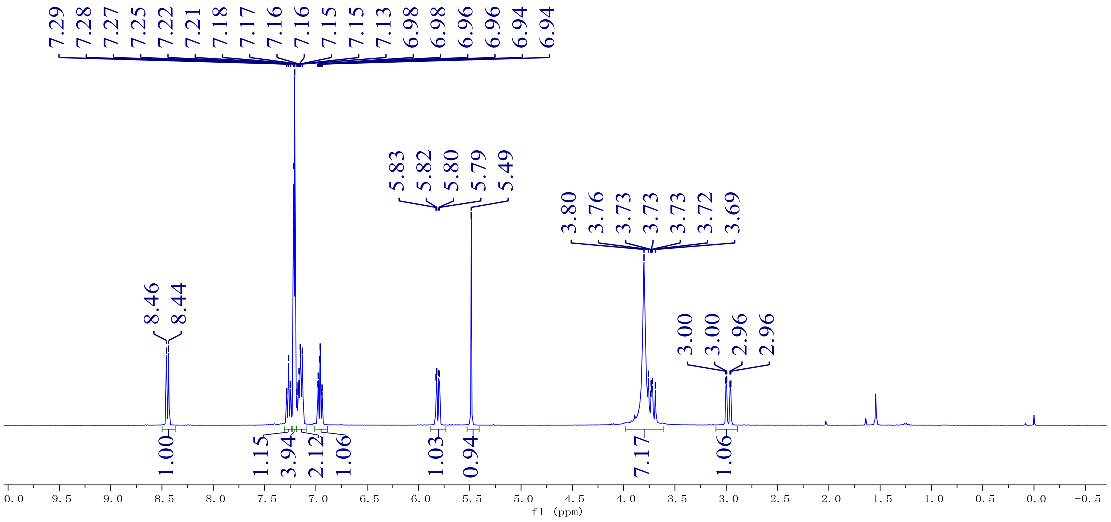

7.29 7.28 7.27 7.25 7.22 7.21 7.18 7.17 7.16 7.16 7.15 7.15 7.13 6.98 6.98 6.96 6.96 6.94 6.94
5.83 5.82 5.80 5.79 5.49
3.80 3.76 3.73 3.73 3.73 3.72 3.69
8.46 8.44
3.00 3.00 2.96 2.96
1.00 1.15 3.94 2.12 1.06 1.03 0.94 7.17 1.06
f1 (ppm)

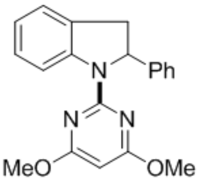

N
Ph
N
N
MeO
OMe

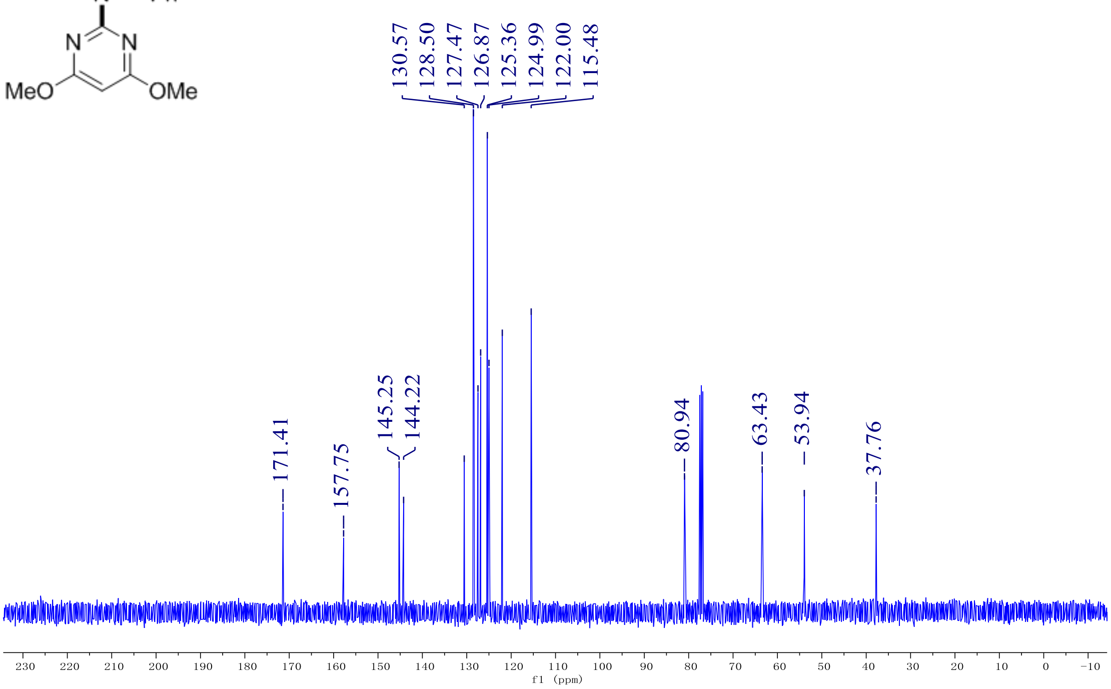

130.57 128.50 127.47 126.87 125.36 124.99 122.00 115.48
171.41
157.75
145.25 144.22
80.94
63.43
53.94
37.76
f1 (ppm)

4q

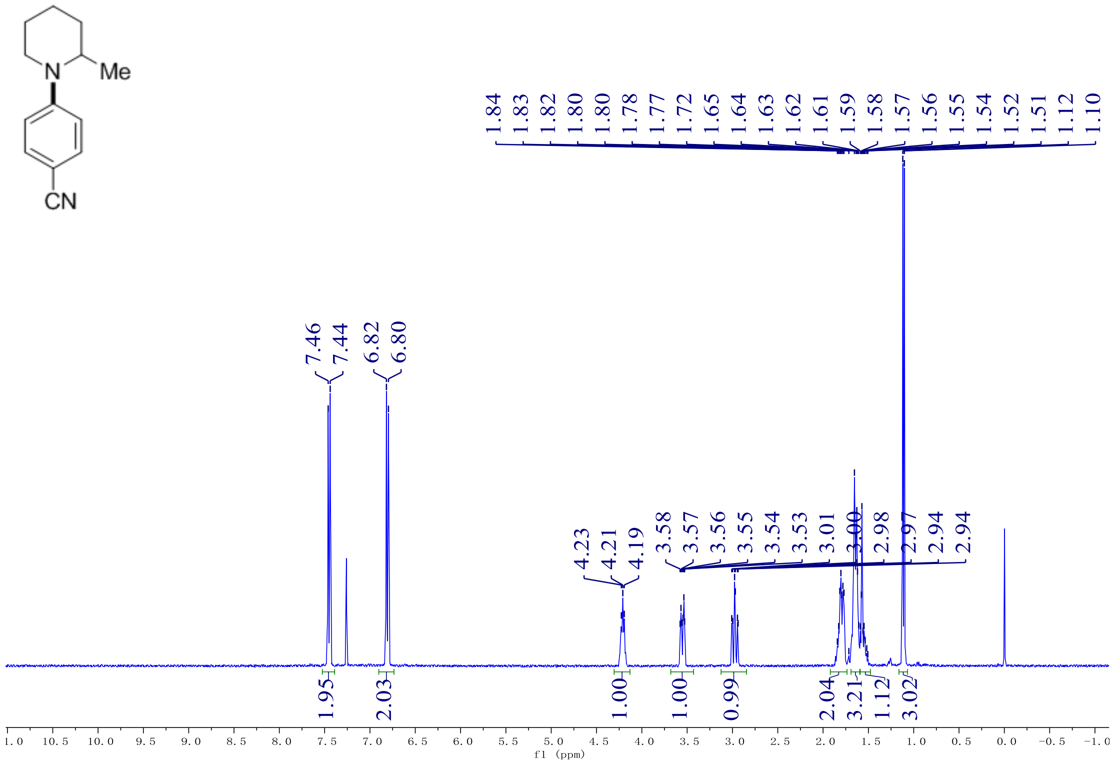
Me
N
CN
1.84
1.83
1.82
1.80
1.80
1.78
1.77
1.72
1.65
1.64
1.63
1.62
1.61
1.59
1.58
1.57
1.56
1.55
1.54
1.52
1.51
1.12
1.10
7.46
7.44
6.82
6.80
4.23
4.21
4.19
3.58
3.57
3.56
3.55
3.54
3.53
3.01
3.00
2.98
2.97
2.94
2.94
1.95
2.03
1.00
1.00
0.99
2.04
3.21
1.12
3.02
f1 (ppm)

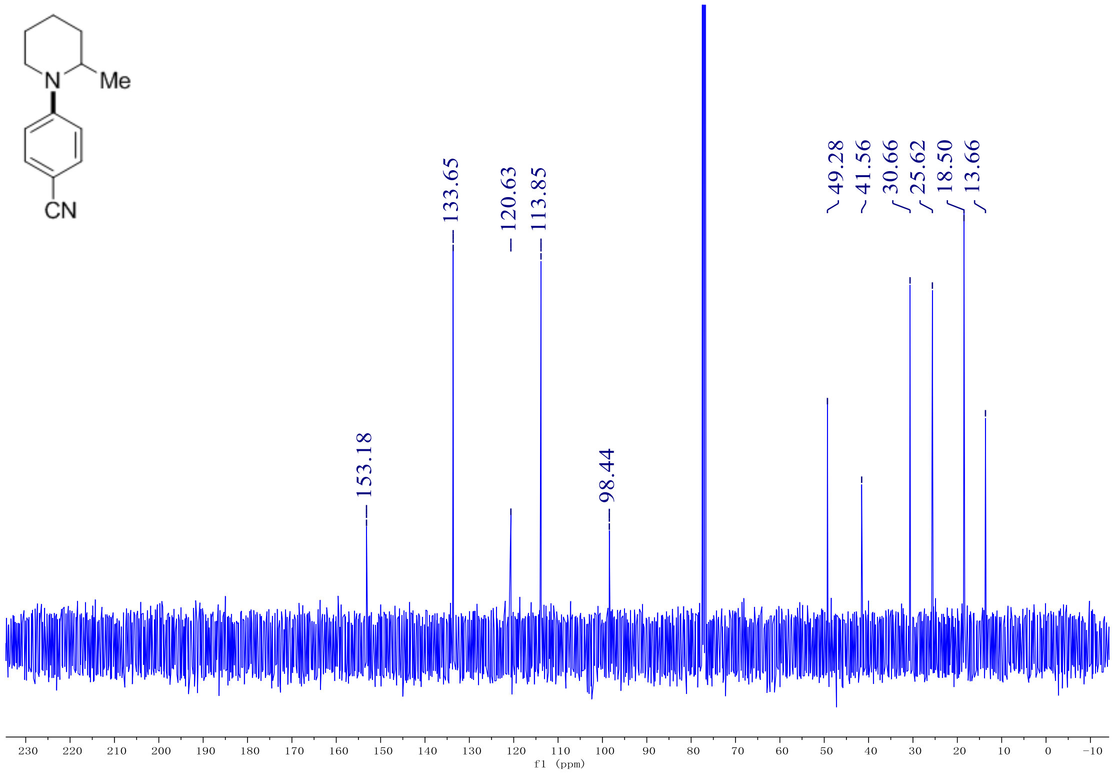
Me
N
CN
133.65
120.63
113.85
153.18
98.44
49.28
41.56
30.66
25.62
18.50
13.66
f1 (ppm)

4r

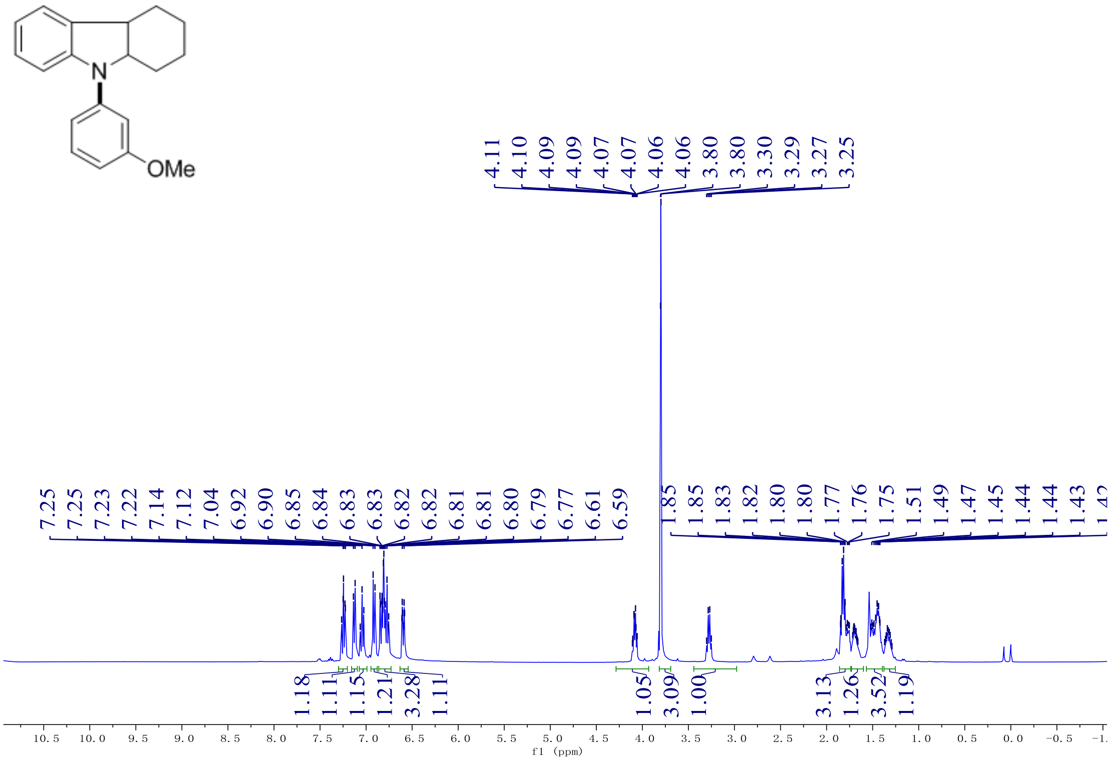
OMe
4.11
4.10
4.09
4.09
4.07
4.07
4.06
4.06
3.80
3.80
3.30
3.29
3.27
3.25
7.25
7.25
7.23
7.22
7.14
7.12
7.04
6.92
6.90
6.85
6.84
6.83
6.83
6.82
6.82
6.81
6.81
6.80
6.79
6.77
6.61
6.59
1.85
1.85
1.83
1.82
1.80
1.80
1.77
1.76
1.75
1.51
1.49
1.47
1.45
1.44
1.44
1.43
1.18
1.11
1.15
1.21
3.28
1.11
1.05
3.09
1.00
3.13
1.26
3.52
1.19
f1 (ppm)

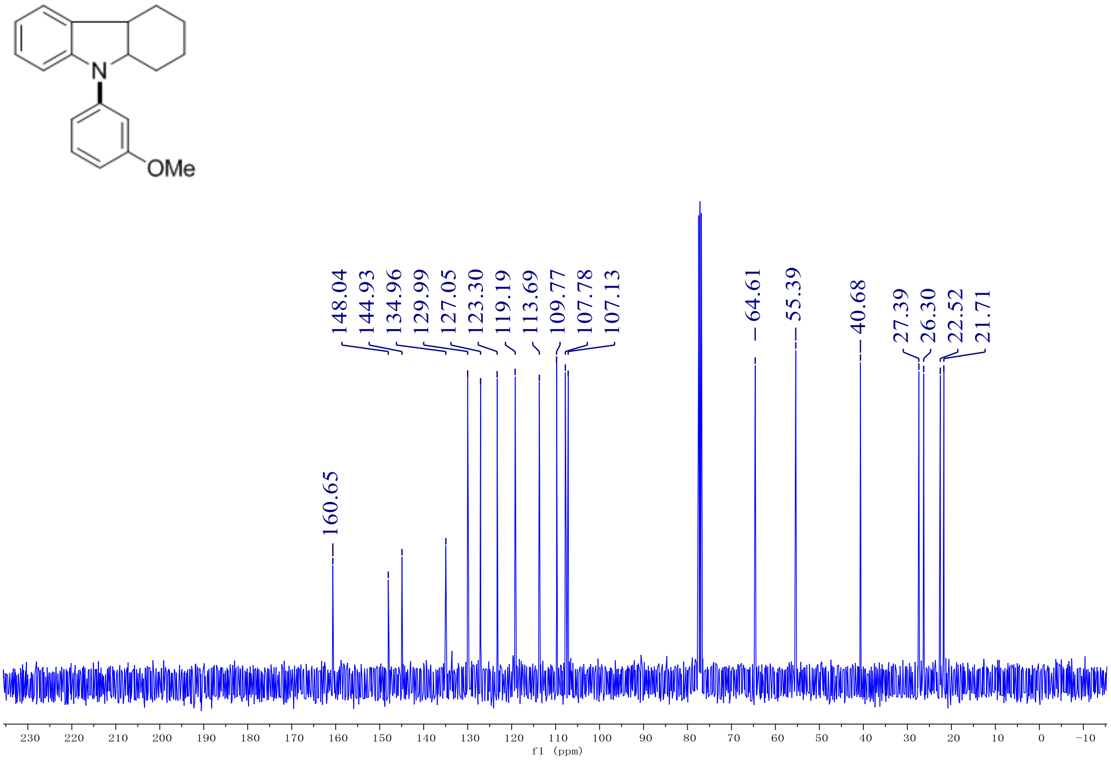
OMe
148.04
144.93
134.96
129.99
127.05
123.30
119.19
113.69
109.77
107.78
107.13
64.61
55.39
40.68
27.39
26.30
22.52
21.71
160.65
f1 (ppm)

4s

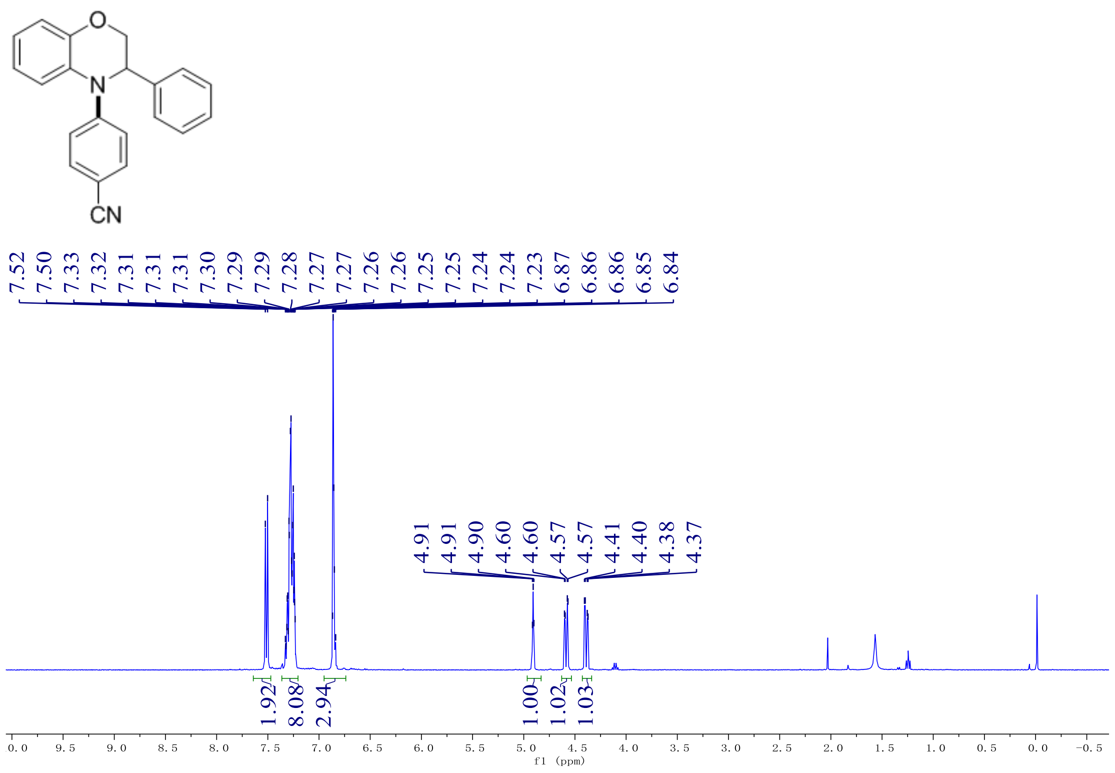
O
N
CN
7.52
7.50
7.33
7.32
7.31
7.31
7.31
7.30
7.29
7.29
7.28
7.27
7.27
7.26
7.26
7.25
7.25
7.24
7.24
7.23
6.87
6.86
6.86
6.85
6.84
4.91
4.91
4.90
4.60
4.60
4.57
4.57
4.41
4.40
4.38
4.37
1.92
8.08
2.94
1.00
1.02
1.03
f1 (ppm)

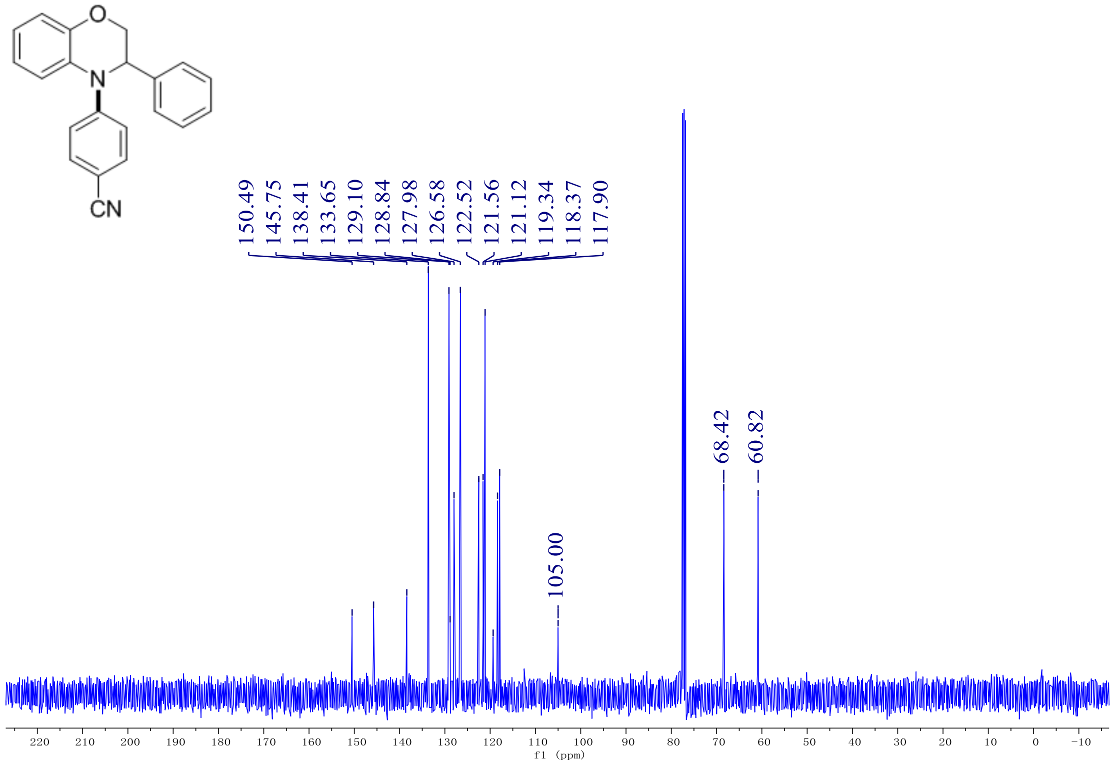
O
N
CN
150.49
145.75
138.41
133.65
129.10
128.84
127.98
126.58
122.52
121.56
121.12
119.34
118.37
117.90
105.00
68.42
60.82
f1 (ppm)

4t

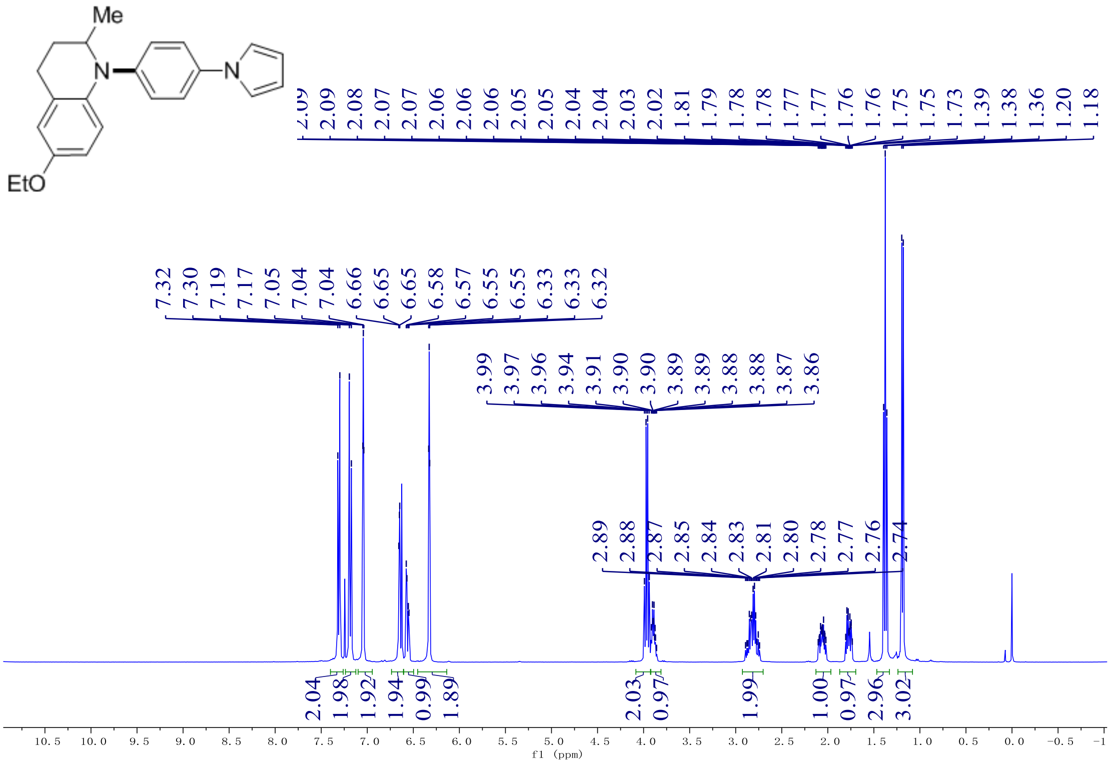
Me
N
N
EtO
2.09 2.09 2.08 2.07 2.07 2.06 2.06 2.06 2.05 2.05 2.04 2.04 2.03 2.02 1.81 1.79 1.78 1.78 1.77 1.77 1.76 1.76 1.75 1.75 1.73 1.39 1.38 1.36 1.20 1.18
7.32 7.30 7.19 7.17 7.05 7.04 7.04 6.66 6.65 6.65 6.58 6.57 6.55 6.55 6.33 6.33 6.32
3.99 3.97 3.96 3.94 3.91 3.90 3.90 3.89 3.89 3.88 3.88 3.87 3.86
2.89 2.88 2.87 2.85 2.84 2.83 2.81 2.80 2.78 2.77 2.76 2.74
2.04 1.98 1.92 1.94 0.99 1.89 2.03 0.97 1.99 1.00 0.97 2.96 3.02
10.5 10.0 9.5 9.0 8.5 8.0 7.5 7.0 6.5 6.0 5.5 5.0 4.5 4.0 3.5 3.0 2.5 2.0 1.5 1.0 0.5 0.0 -0.5 -1
f1 (ppm)

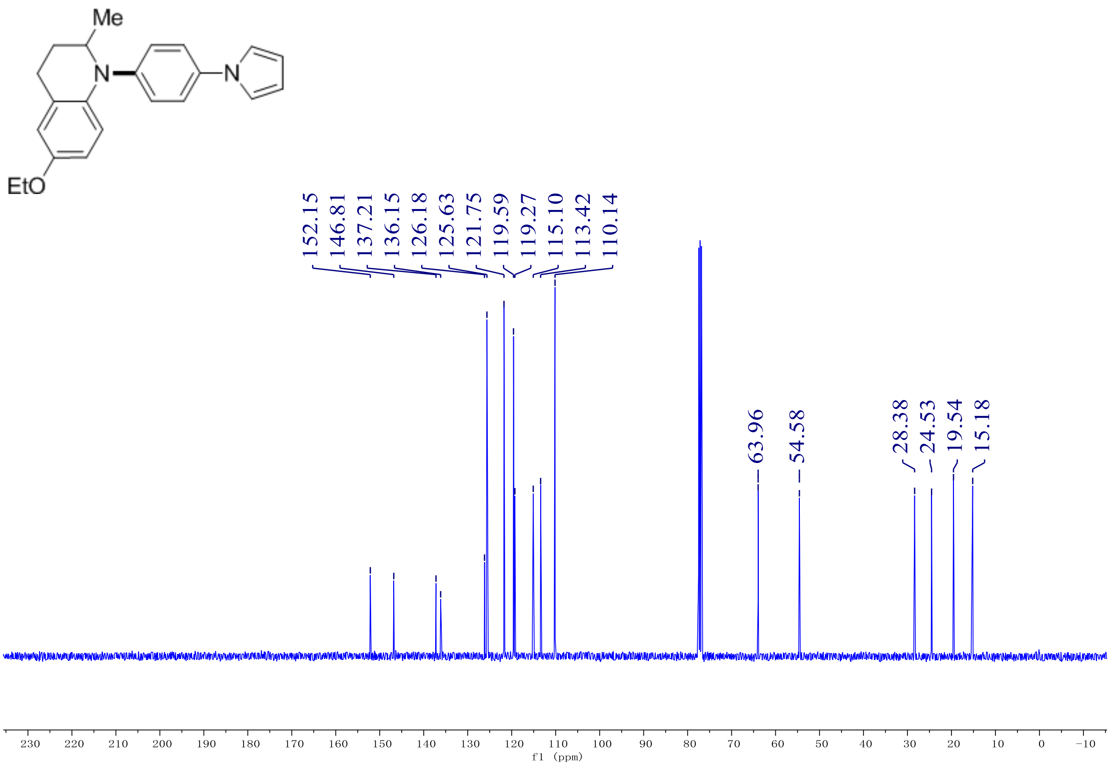
Me
N
N
EtO
152.15 146.81 137.21 136.15 126.18 125.63 121.75 119.59 119.27 115.10 113.42 110.14
63.96
54.58
28.38 24.53 19.54 15.18
230 220 210 200 190 180 170 160 150 140 130 120 110 100 90 80 70 60 50 40 30 20 10 0 -10
f1 (ppm)

5a

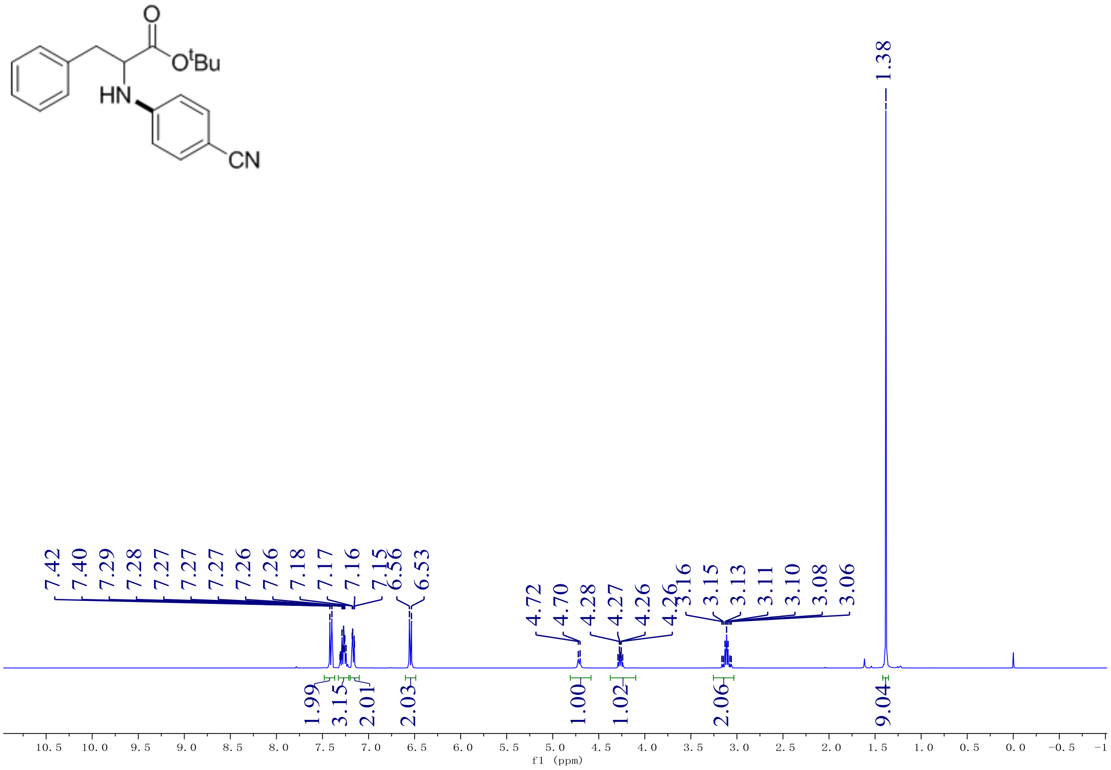
O
O^tBu
HN
CN
1.38
7.42
7.40
7.29
7.28
7.27
7.27
7.27
7.26
7.26
7.18
7.17
7.16
7.15
6.56
6.53
4.72
4.70
4.28
4.27
4.26
4.26
3.16
3.15
3.13
3.11
3.10
3.08
3.06
1.99
3.15
2.01
2.03
1.00
1.02
2.06
9.04
f1 (ppm)

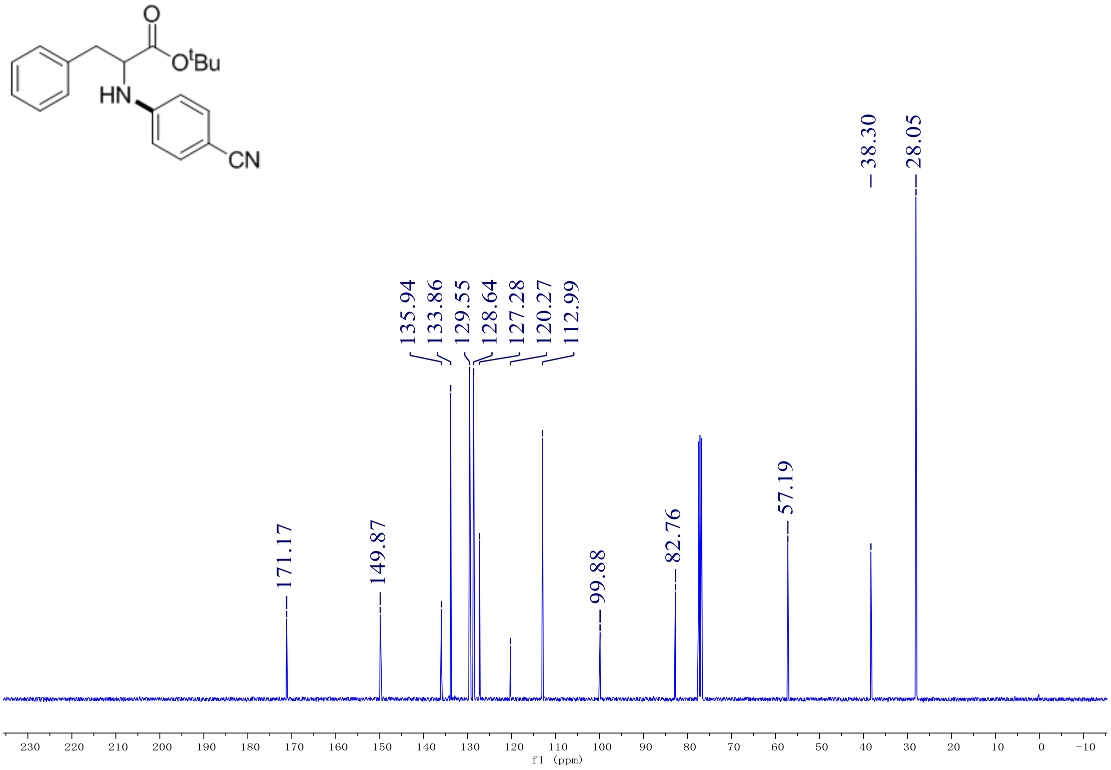
O
O^tBu
HN
CN
38.30
28.05
135.94
133.86
129.55
128.64
127.28
120.27
112.99
171.17
149.87
99.88
82.76
57.19
f1 (ppm)

5b

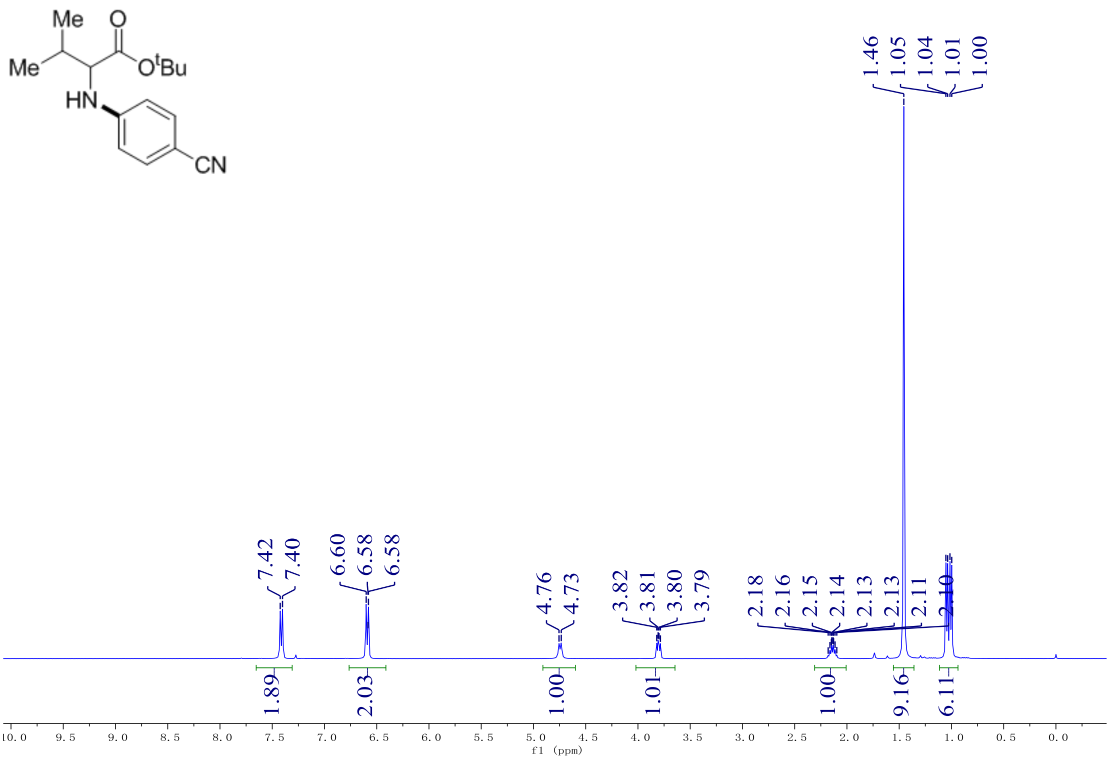

Me
O
Me
O^{t}Bu
HN
CN
1.46
1.05
1.04
1.01
1.00
7.42
7.40
6.60
6.58
6.58
4.76
4.73
3.82
3.81
3.80
3.79
2.18
2.16
2.15
2.14
2.13
2.13
2.11
1.89
2.03
1.00
1.01
1.00
9.16
6.11
f1 (ppm)

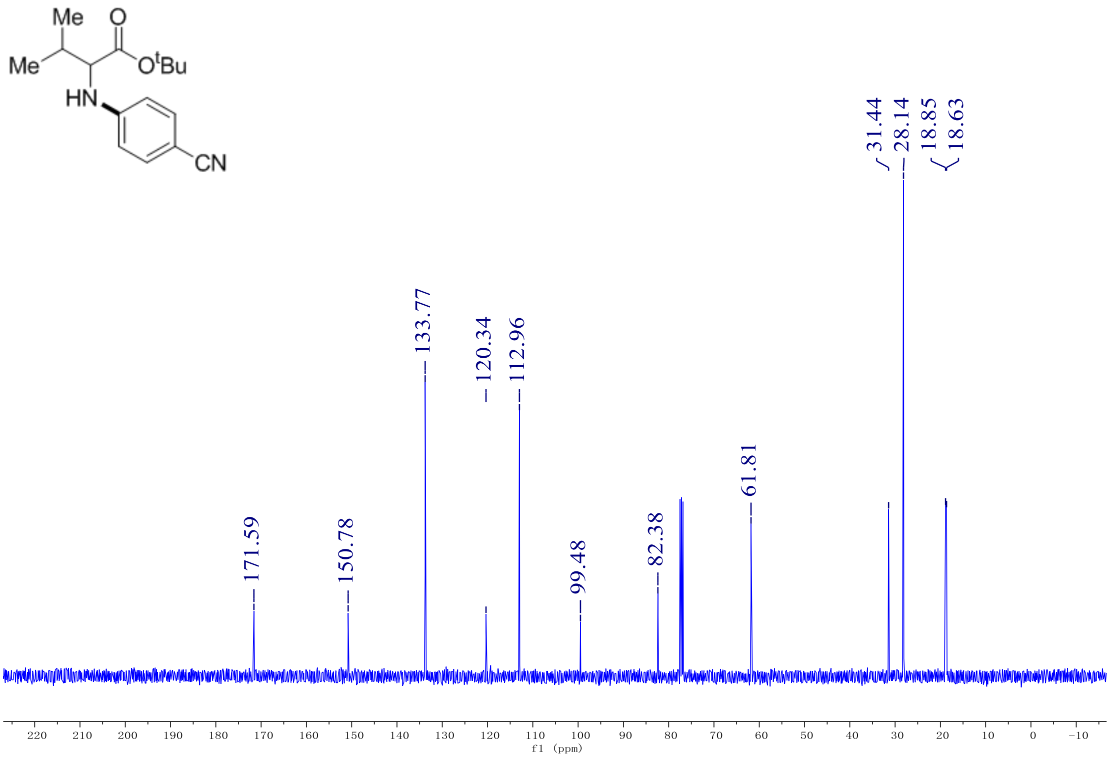

Me
O
Me
O^{t}Bu
HN
CN
31.44
28.14
18.85
18.63
133.77
120.34
112.96
61.81
171.59
150.78
99.48
82.38
f1 (ppm)

5c

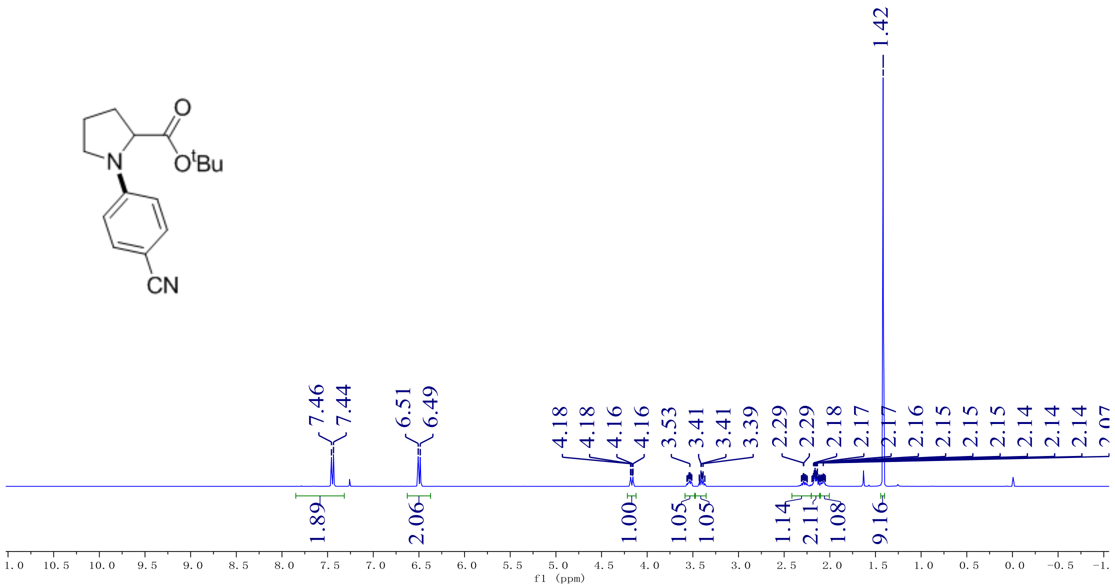
O
O^{t}Bu
N
CN
7.46
7.44
6.51
6.49
4.18
4.18
4.16
4.16
3.53
3.41
3.41
3.39
2.29
2.29
2.18
2.17
2.17
2.16
2.15
2.15
2.15
2.14
2.14
2.14
1.42
1.89
2.06
1.00
1.05
1.05
1.14
2.11
1.08
9.16
f1 (ppm)

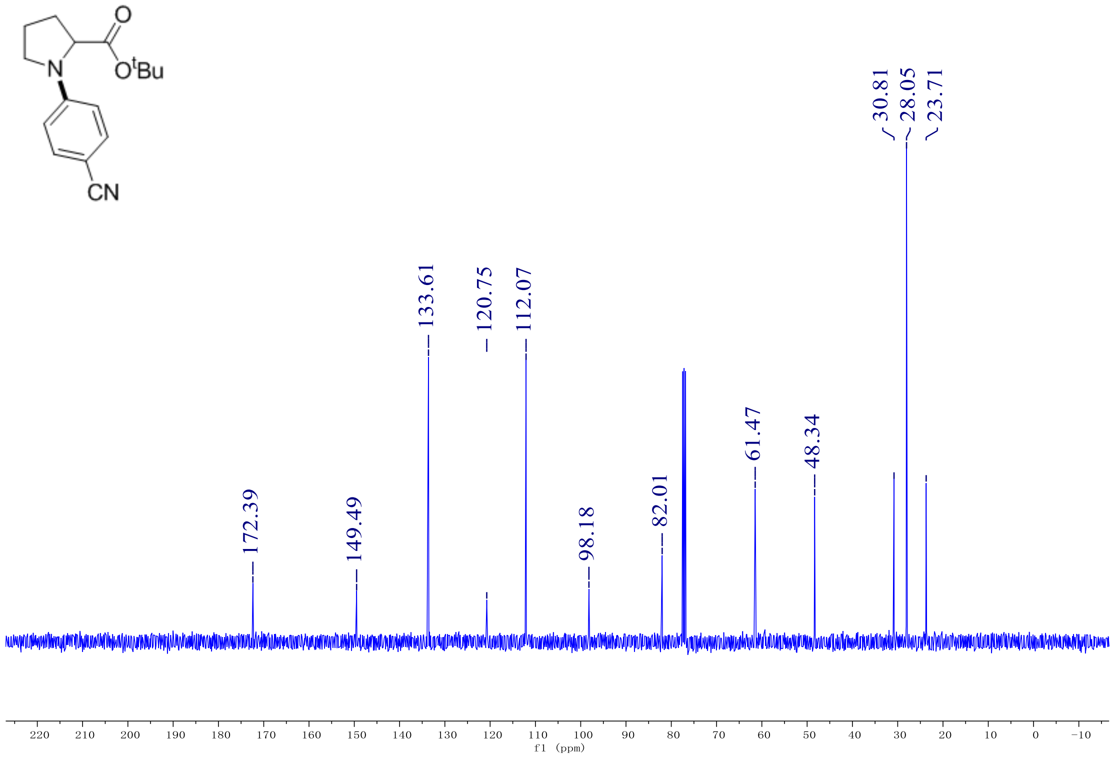
O
O^{t}Bu
N
CN
172.39
149.49
133.61
120.75
112.07
98.18
82.01
61.47
48.34
30.81
28.05
23.71
f1 (ppm)

5d

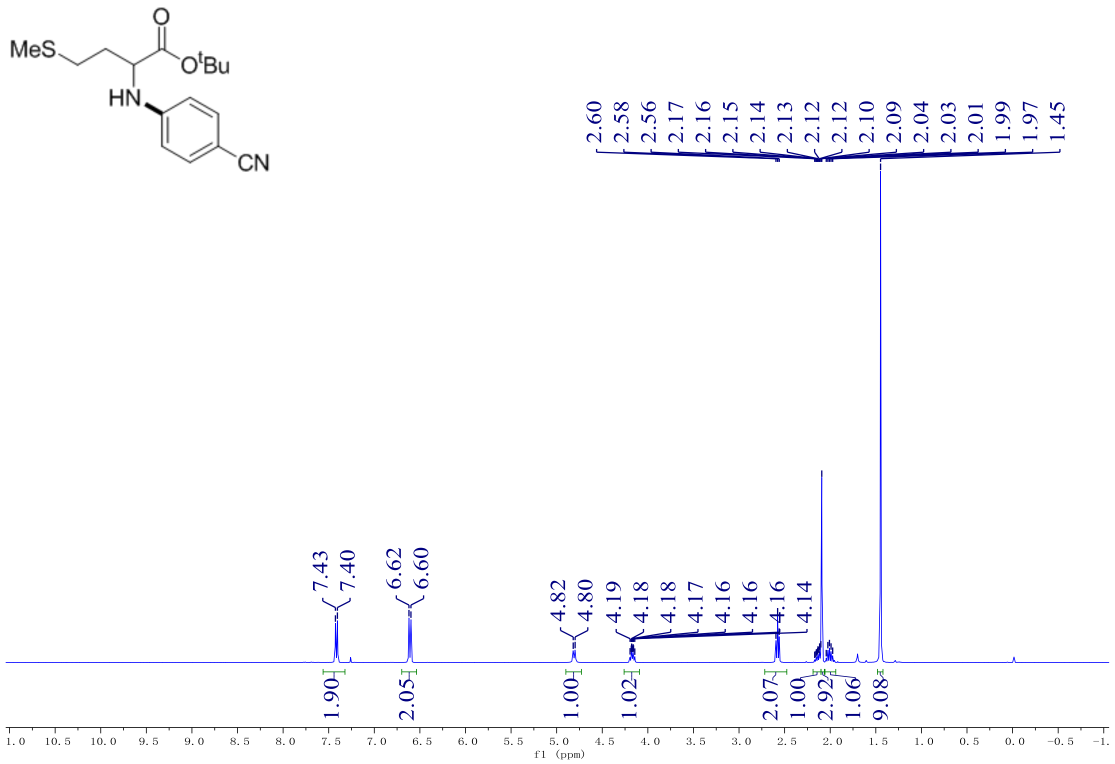
MeS
O
O^tBu
HN
CN
2.60
2.58
2.56
2.17
2.16
2.15
2.14
2.13
2.12
2.12
2.10
2.09
2.04
2.03
2.01
1.99
1.97
1.45
7.43
7.40
6.62
6.60
4.82
4.80
4.19
4.18
4.18
4.17
4.16
4.16
4.16
4.14
1.90
2.05
1.00
1.02
2.07
1.00
2.92
1.06
9.08
f1 (ppm)

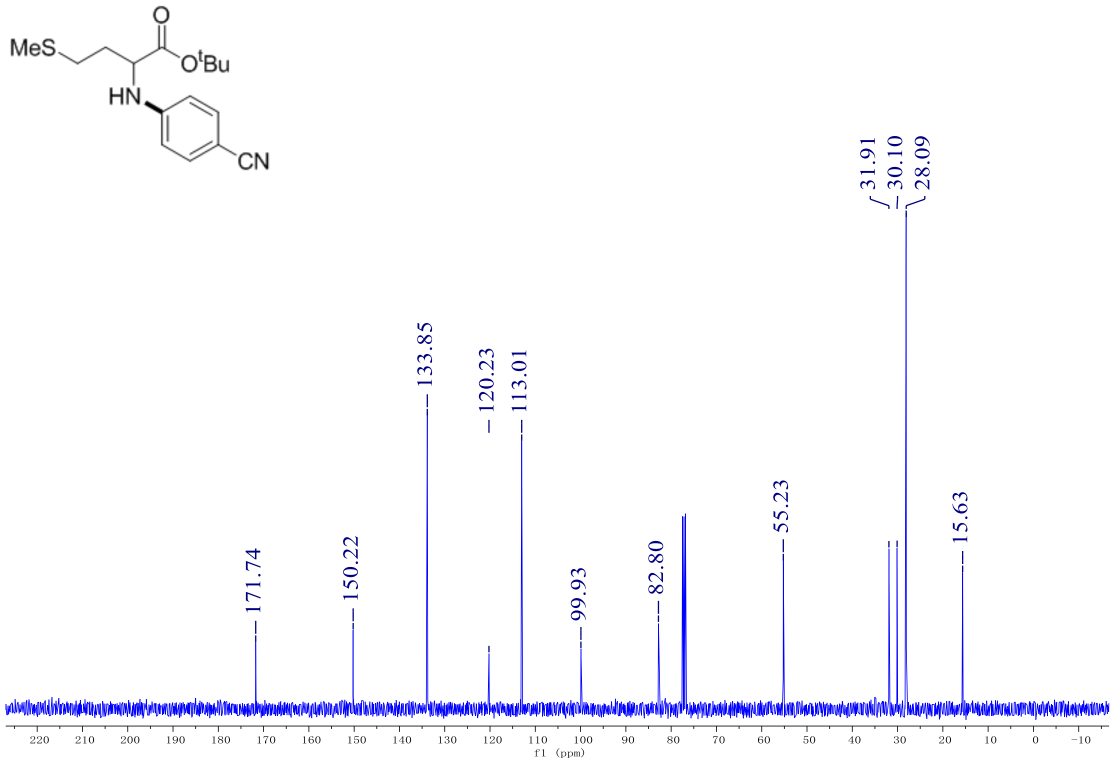
MeS
O
O^tBu
HN
CN
31.91
30.10
28.09
133.85
120.23
113.01
55.23
15.63
171.74
150.22
99.93
82.80
f1 (ppm)

5e

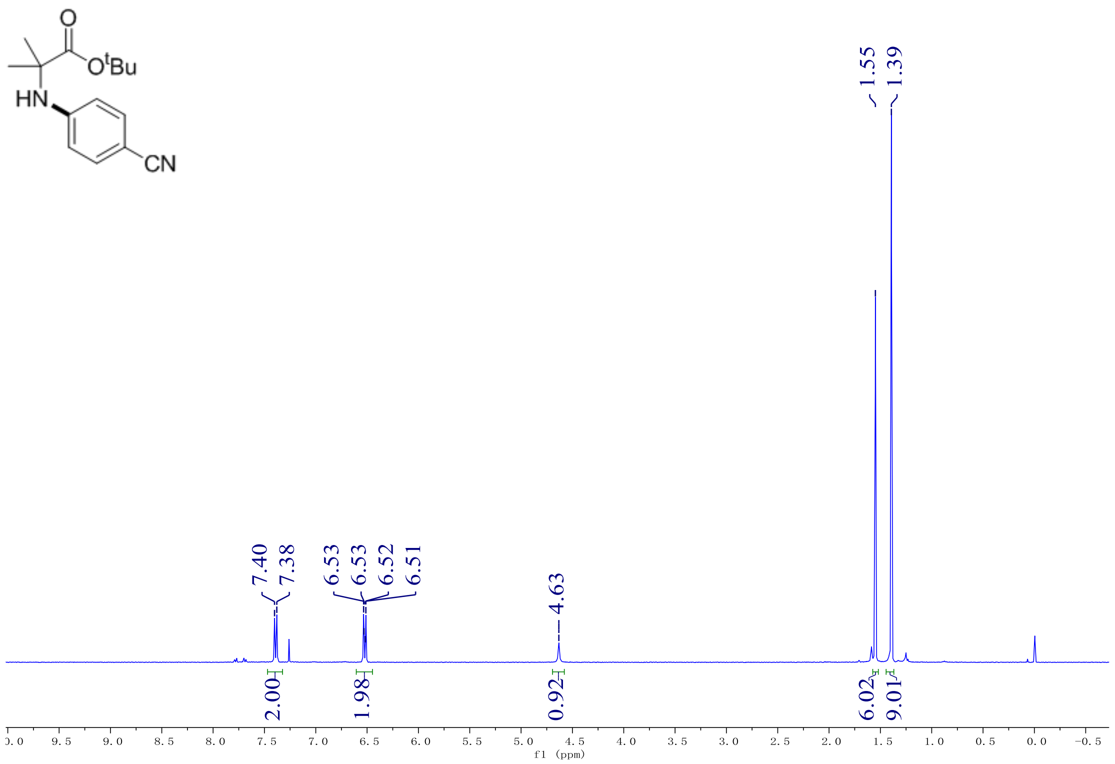
O
O^tBu
HN
CN
1.55
1.39
7.40
7.38
6.53
6.53
6.52
6.51
4.63
2.00
1.98
0.92
6.02
9.01
f1 (ppm)

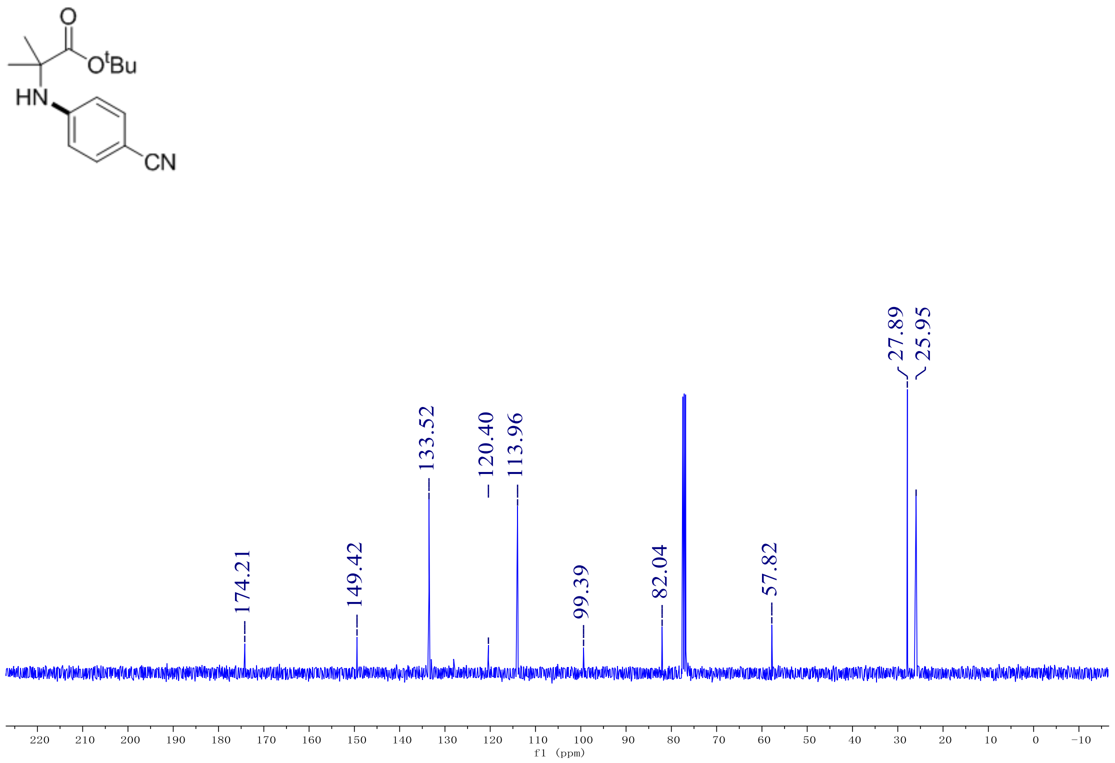
O
O^tBu
HN
CN
174.21
149.42
133.52
120.40
113.96
99.39
82.04
57.82
27.89
25.95
f1 (ppm)

6a

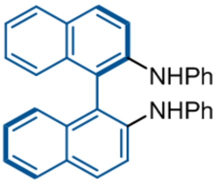
NHPh
NHPh

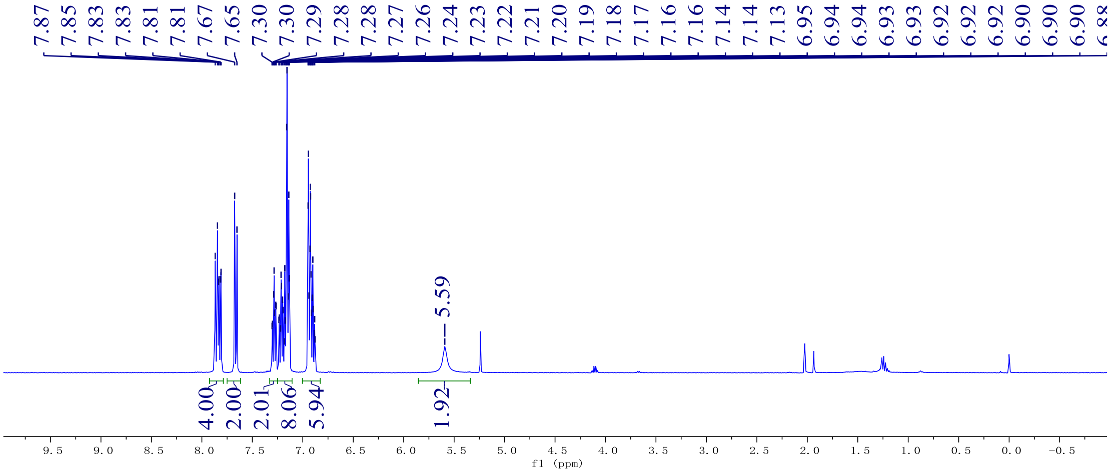
7.87 7.85 7.83 7.83 7.81 7.81 7.67 7.65 7.30 7.30 7.29 7.28 7.28 7.27 7.26 7.24 7.23 7.22 7.21 7.20 7.19 7.18 7.17 7.16 7.16 7.14 7.14 7.13 6.95 6.94 6.94 6.93 6.93 6.92 6.92 6.92 6.90 6.90 6.90
5.59
4.00 2.00 2.01 8.06 5.94 1.92
f1 (ppm)

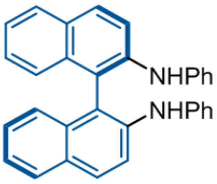
NHPh
NHPh

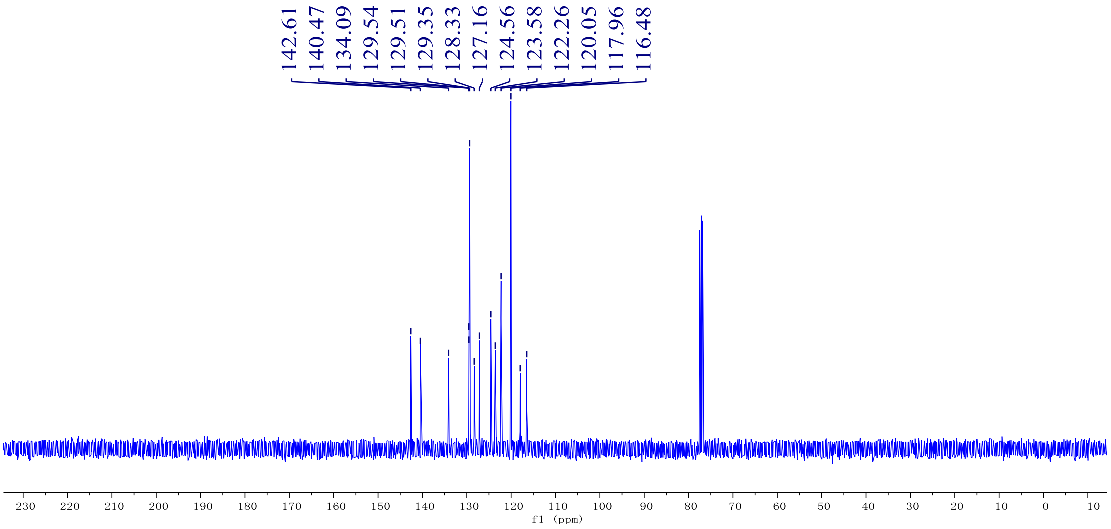
142.61 140.47 134.09 129.54 129.51 129.35 128.33 127.16 124.56 123.58 122.26 120.05 117.96 116.48
f1 (ppm)

6b

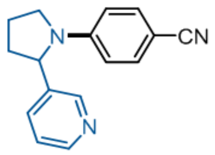
N
CN
N

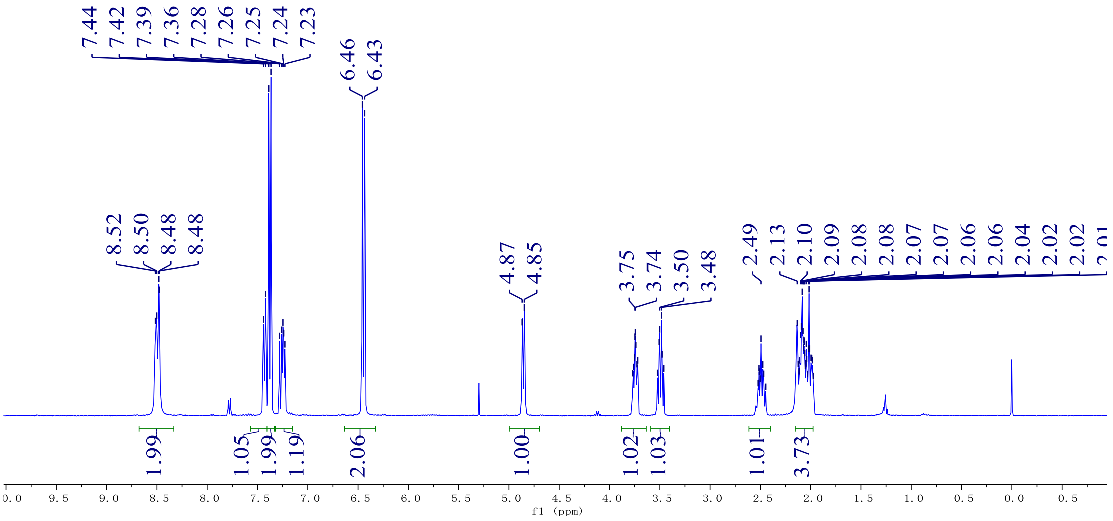
7.44
7.42
7.39
7.36
7.28
7.26
7.25
7.24
7.23
6.46
6.43
8.52
8.50
8.48
8.48
4.87
4.85
3.75
3.74
3.50
3.48
2.49
2.13
2.10
2.09
2.08
2.08
2.07
2.07
2.06
2.06
2.04
2.02
2.02
1.99
1.05
1.99
1.19
2.06
1.00
1.02
1.03
1.01
3.73
f1 (ppm)

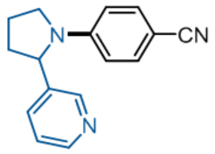
N
CN
N

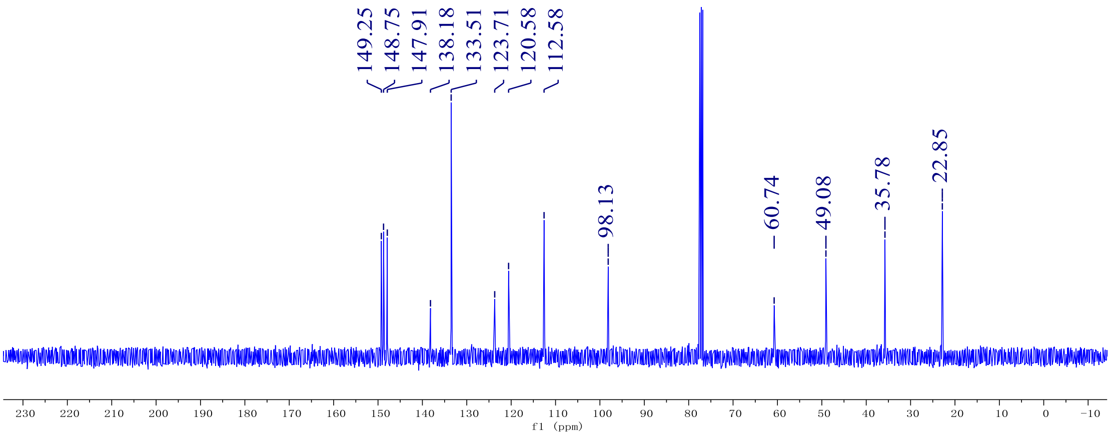
149.25
148.75
147.91
138.18
133.51
123.71
120.58
112.58
98.13
60.74
49.08
35.78
22.85
f1 (ppm)

6c

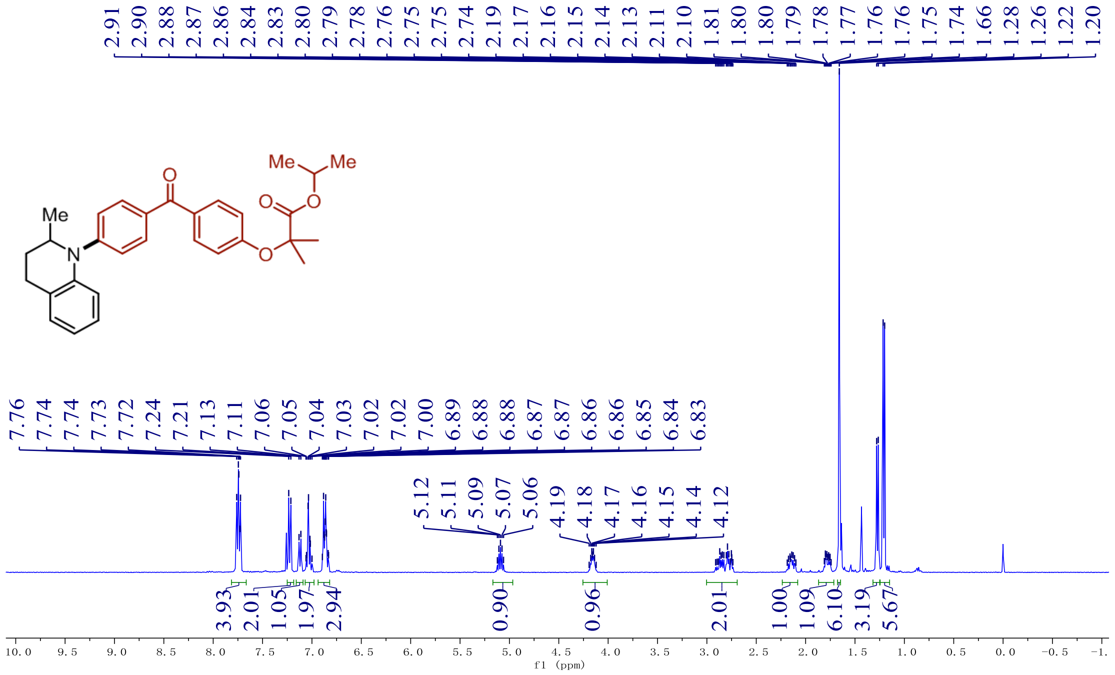
2.91 2.90 2.88 2.87 2.86 2.84 2.83 2.80 2.79 2.78 2.76 2.75 2.75 2.74 2.19 2.17 2.16 2.15 2.14 2.13 2.11 2.10 1.81 1.80 1.80 1.79 1.78 1.77 1.76 1.76 1.75 1.74 1.66 1.28 1.26 1.22 1.20
Me
O
Me
Me
O
O
N
O
7.76 7.74 7.74 7.73 7.72 7.24 7.21 7.13 7.11 7.06 7.05 7.04 7.03 7.02 7.02 7.00 6.89 6.88 6.88 6.87 6.87 6.86 6.86 6.85 6.84 6.83
5.12 5.11 5.09 5.07 5.06
4.19 4.18 4.17 4.16 4.15 4.14 4.12
3.93 2.01 1.05 1.97 2.94
0.90
0.96
2.01
1.00 1.09 6.10 3.19 5.67
10.0 9.5 9.0 8.5 8.0 7.5 7.0 6.5 6.0 5.5 5.0 4.5 4.0 3.5 3.0 2.5 2.0 1.5 1.0 0.5 0.0 -0.5 -1.
f1 (ppm)

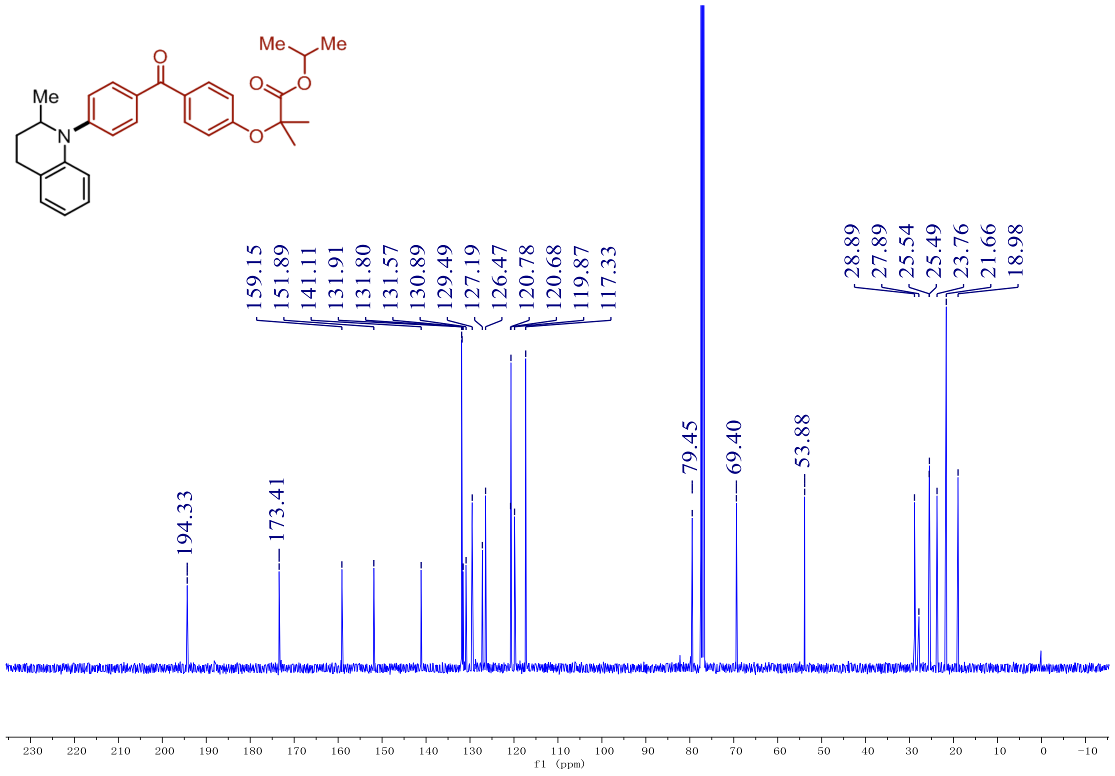
Me
O
Me
Me
O
O
N
O
159.15 151.89 141.11 131.91 131.80 131.57 130.89 129.49 127.19 126.47 120.78 120.68 119.87 117.33
28.89 27.89 25.54 25.49 23.76 21.66 18.98
194.33
173.41
79.45
69.40
53.88
230 220 210 200 190 180 170 160 150 140 130 120 110 100 90 80 70 60 50 40 30 20 10 0 -10
f1 (ppm)

6d

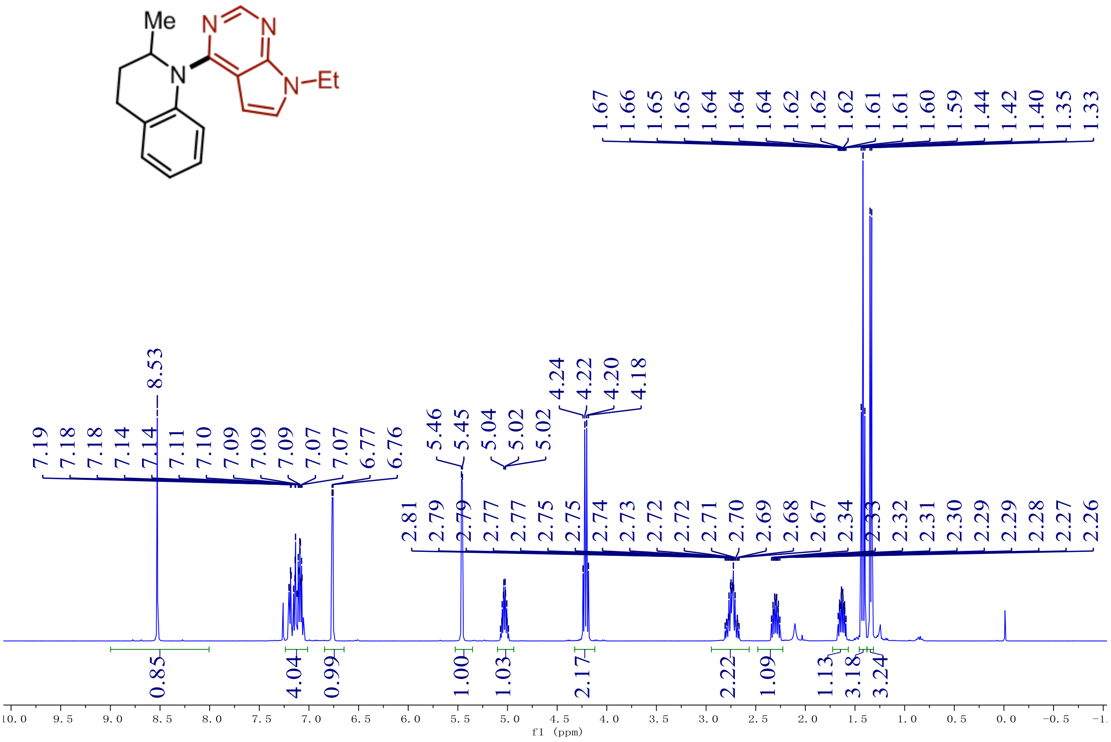
Me
N
N
N
N–Et
1.67 1.66 1.65 1.65 1.64 1.64 1.64 1.62 1.62 1.62 1.61 1.61 1.60 1.59 1.44 1.42 1.40 1.35 1.33
8.53
7.19 7.18 7.18 7.14 7.14 7.11 7.10 7.09 7.09 7.09 7.07 7.07 6.77 6.76
5.46 5.45
5.04 5.02 5.02
4.24 4.22 4.20 4.18
2.81 2.79 2.79 2.77 2.77 2.75 2.75 2.74 2.73 2.72 2.72 2.71 2.70 2.69 2.68 2.67 2.34 2.33 2.32 2.31 2.30 2.29 2.29 2.28 2.27 2.26
0.85
4.04
0.99
1.00
1.03
2.17
2.22
1.09
1.13
3.18
3.24
f1 (ppm)

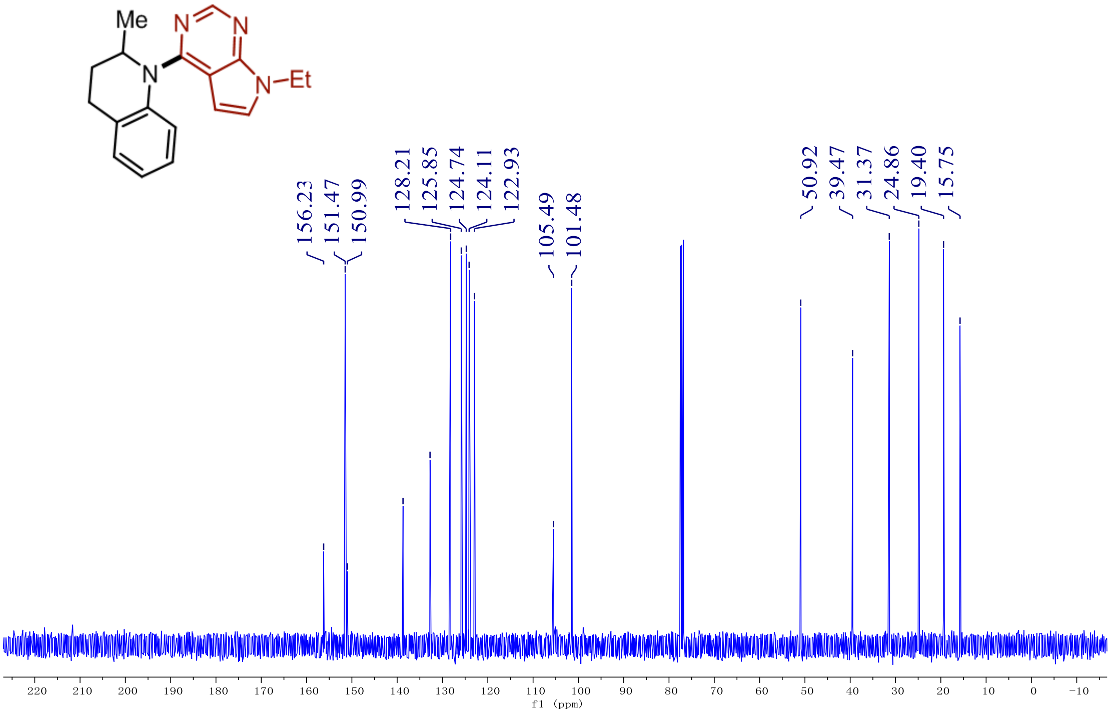
Me
N
N
N
N–Et
156.23 151.47 150.99
128.21 125.85 124.74 124.11 122.93
105.49 101.48
50.92 39.47 31.37 24.86 19.40 15.75
f1 (ppm)

7

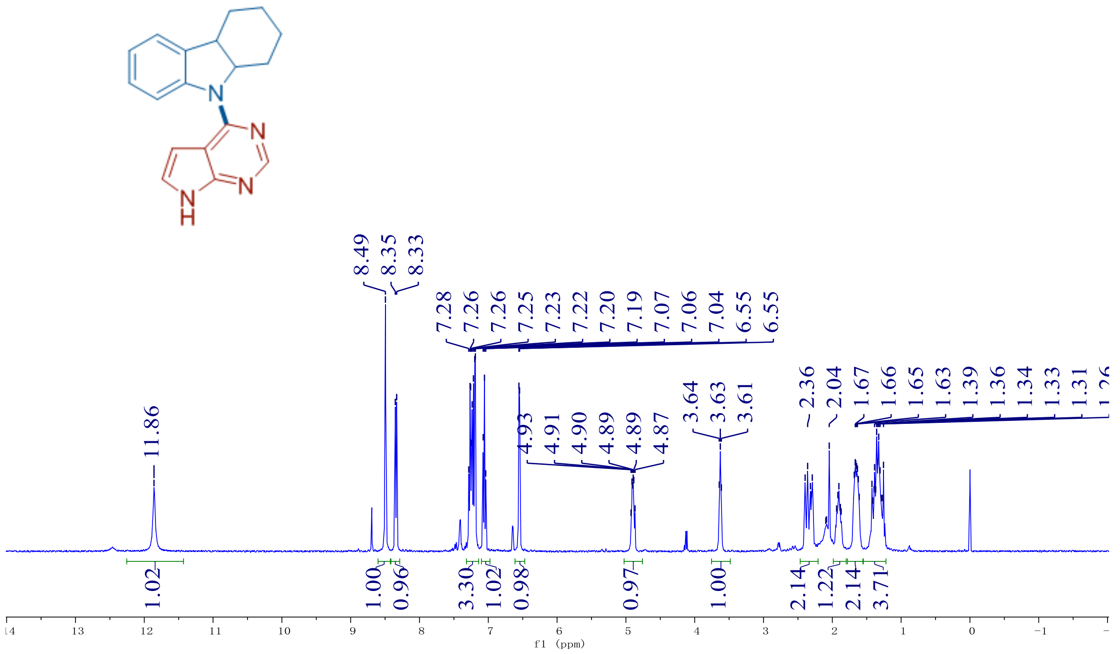
N
N
N
NH
11.86
8.49
8.35
8.33
7.28
7.26
7.26
7.25
7.23
7.22
7.20
7.19
7.07
7.06
7.04
6.55
6.55
4.93
4.91
4.90
4.89
4.89
4.87
3.64
3.63
3.61
2.36
2.04
1.67
1.66
1.65
1.63
1.39
1.36
1.34
1.33
1.31
1.02
1.00
0.96
3.30
1.02
0.98
0.97
1.00
2.14
1.22
2.14
3.71
f1 (ppm)

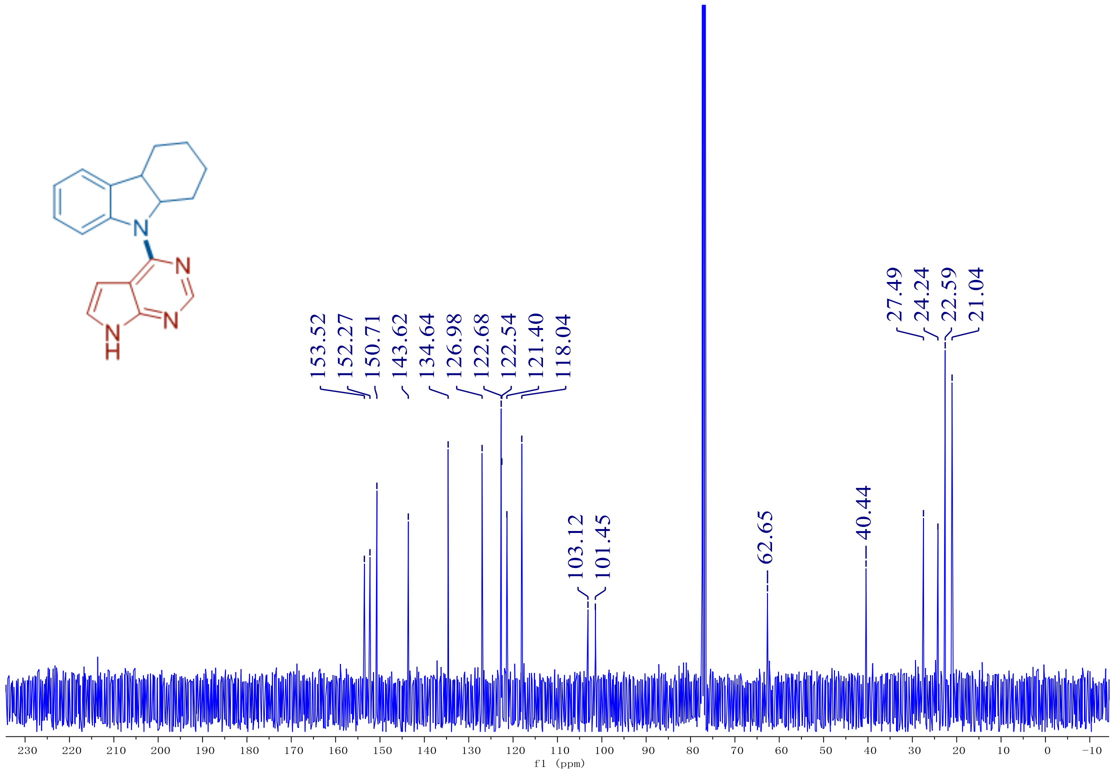
N
N
N
NH
153.52
152.27
150.71
143.62
134.64
126.98
122.68
122.54
121.40
118.04
103.12
101.45
62.65
40.44
27.49
24.24
22.59
21.04
f1 (ppm)

8

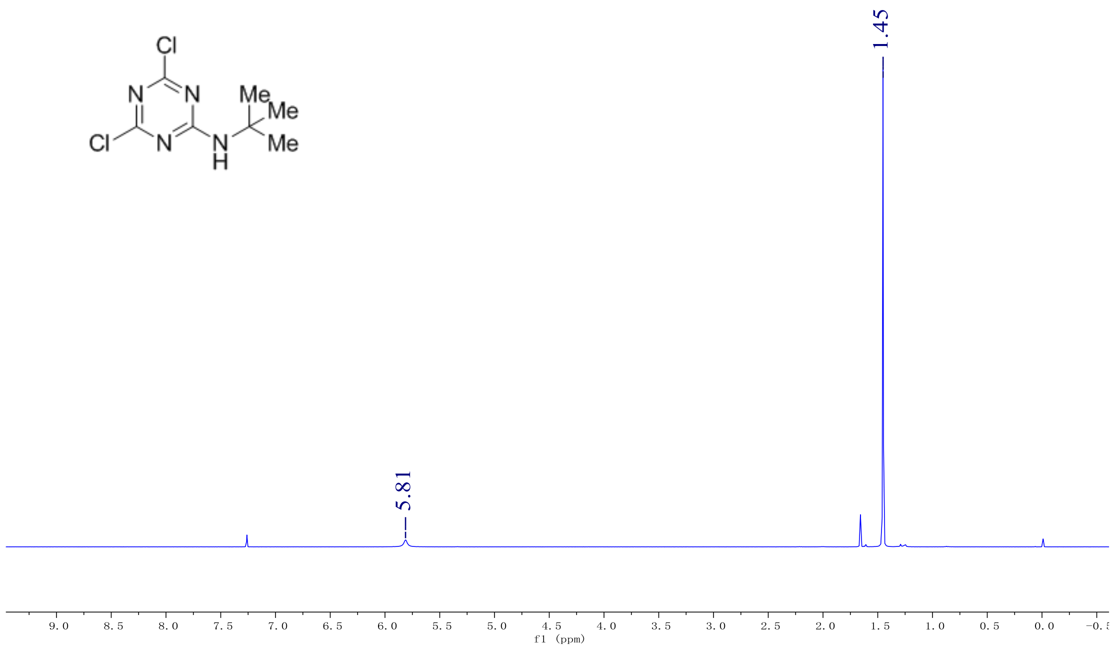

Cl
N
N
Me
Me
Cl
N
N
H
Me
1.45
5.81
9.0 8.5 8.0 7.5 7.0 6.5 6.0 5.5 5.0 4.5 4.0 3.5 3.0 2.5 2.0 1.5 1.0 0.5 0.0
f1 (ppm)

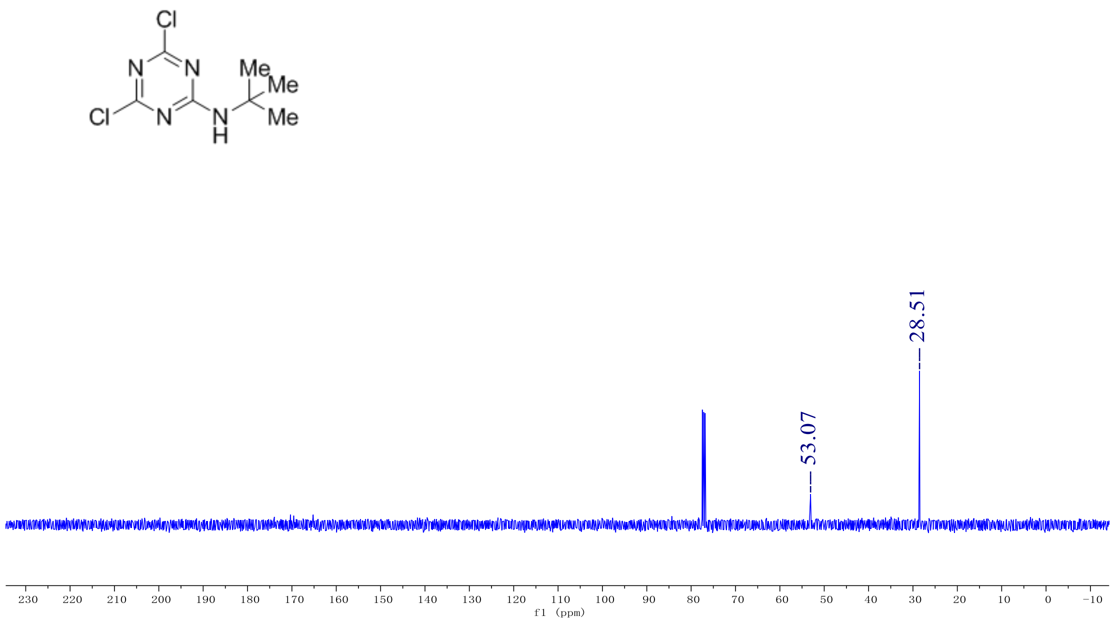

Cl
N
N
Me
Me
Cl
N
N
H
Me
28.51
53.07
230 220 210 200 190 180 170 160 150 140 130 120 110 100 90 80 70 60 50 40 30 20 10 0 -10
f1 (ppm)

9

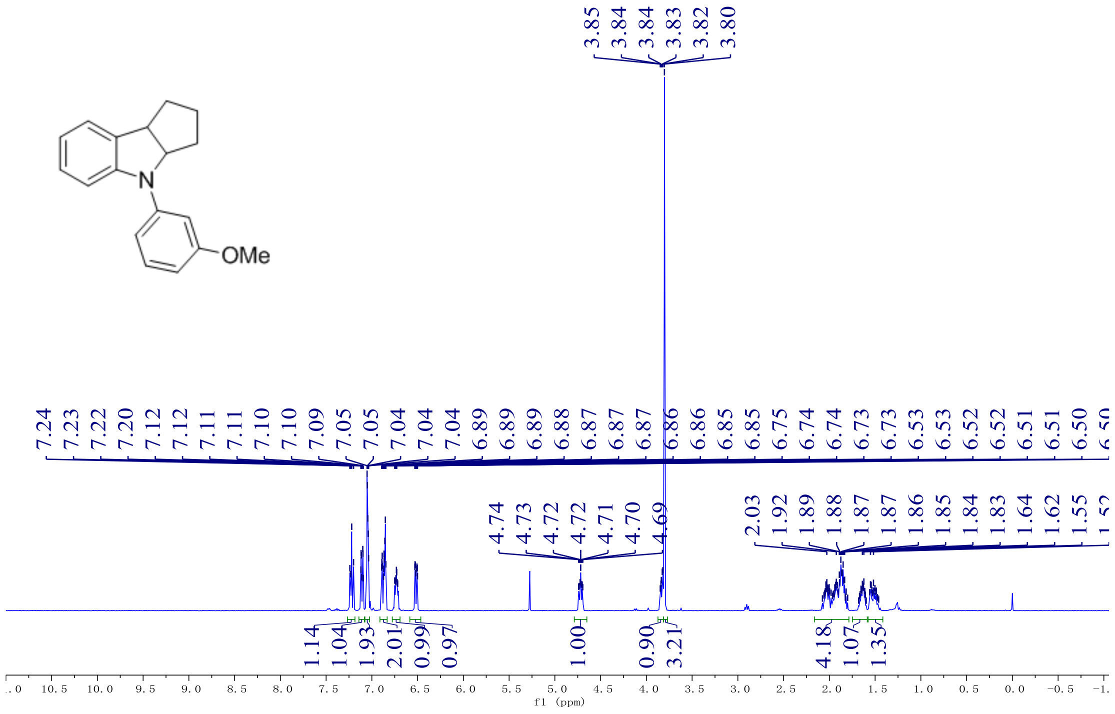
OMe
N
3.85
3.84
3.84
3.83
3.82
3.80
7.24
7.23
7.22
7.20
7.12
7.12
7.11
7.11
7.10
7.10
7.09
7.05
7.05
7.04
7.04
7.04
6.89
6.89
6.89
6.88
6.87
6.87
6.87
6.86
6.86
6.85
6.85
6.75
6.74
6.74
6.73
6.73
6.53
6.53
6.52
6.52
6.51
6.51
6.50
4.74
4.73
4.72
4.72
4.71
4.70
4.69
2.03
1.92
1.89
1.88
1.87
1.87
1.86
1.85
1.84
1.83
1.64
1.62
1.55
1.14
1.04
1.93
2.01
0.99
0.97
1.00
0.90
3.21
4.18
1.07
1.35
f1 (ppm)

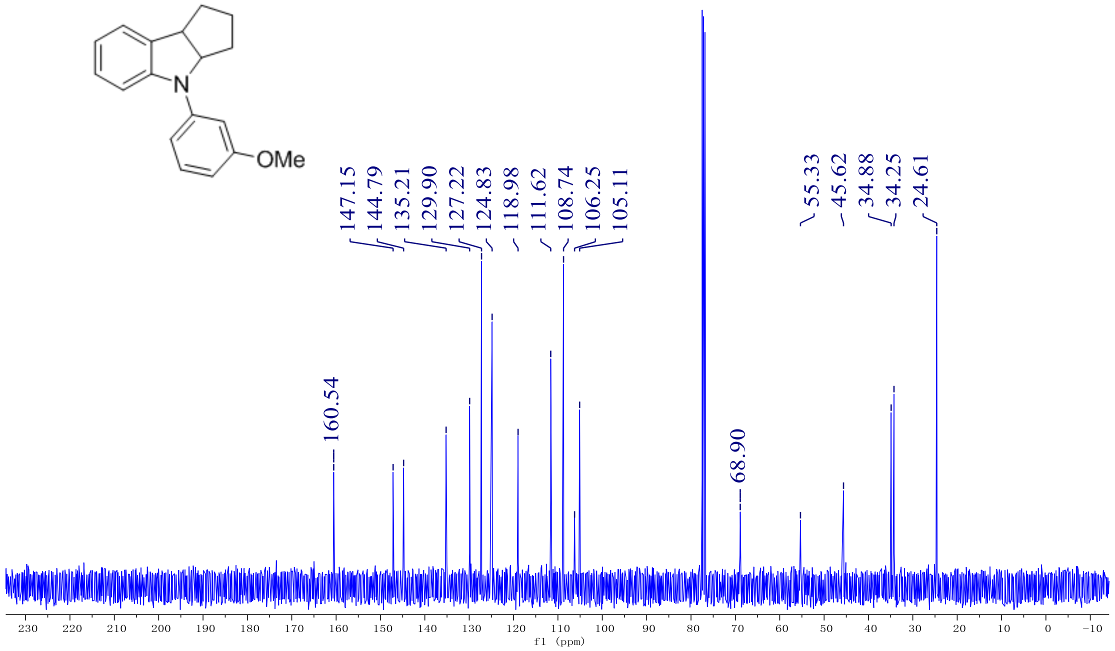
N
OMe
147.15
144.79
135.21
129.90
127.22
124.83
118.98
111.62
108.74
106.25
105.11
55.33
45.62
34.88
34.25
24.61
160.54
68.90
f1 (ppm)
